# Supplementary material for: Phosphorylation controls spatial and temporal activities of motor‐PRC1 complexes to complete mitosis
Source: EMBO J. 2023 Aug 18;42(21):e113647. doi: 10.15252/embj.2023113647 (PMC10620760; doi:10.15252/embj.2023113647)
Supplement: Supplementary file 1 — Expanded View Figures PDF [file EMBJ-42-e113647-s004.pdf]

Expanded View Figures

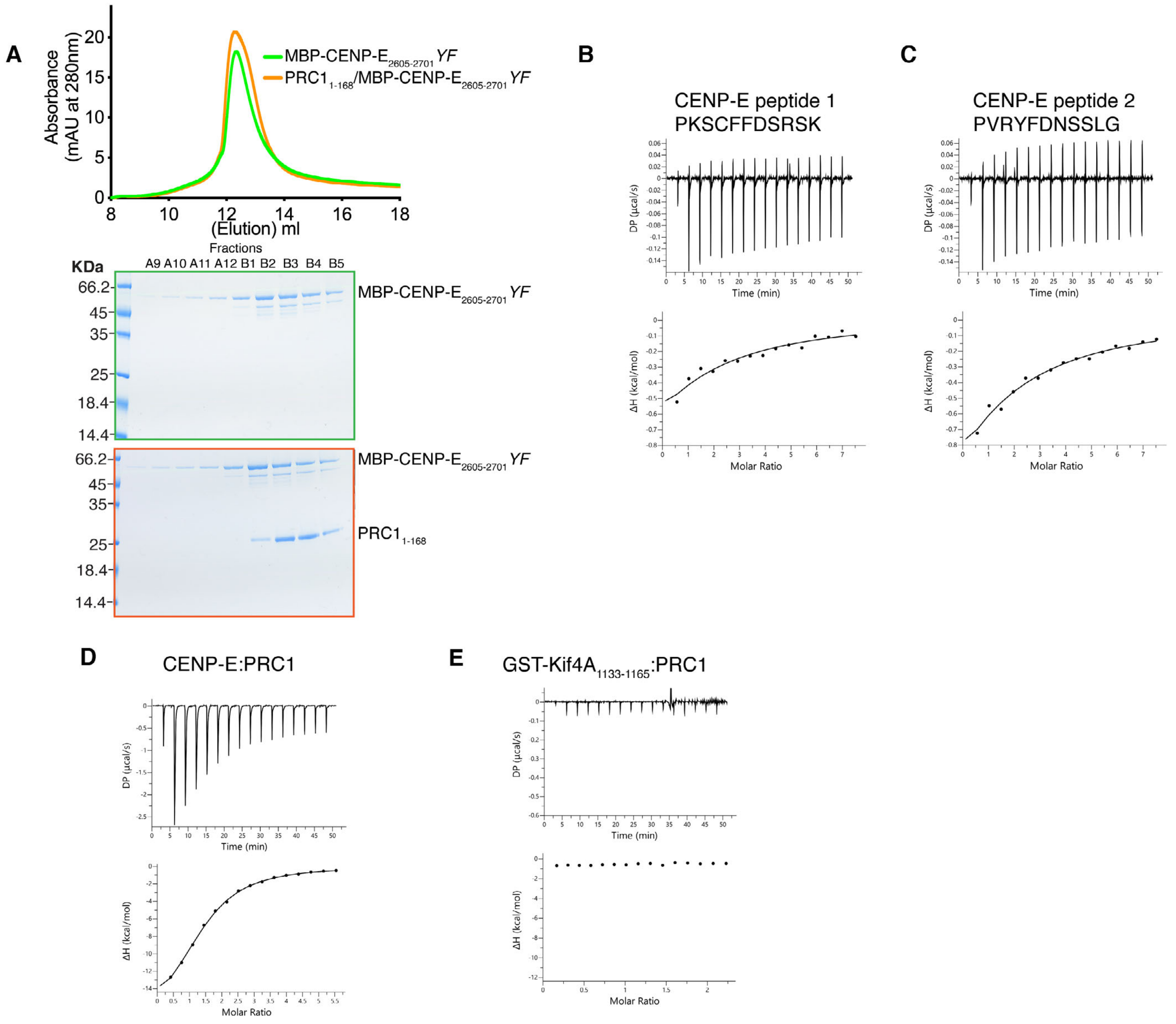

Figure EV1. Characterization of the PRC1/CENP-E interaction.

A Top. Size-exclusion chromatography elution profile of MBP-CENP-$E_{2605–2701}$*YF* (green) and MBP-CENP-$E_{2605–2701}$ *YF*/$PRC1_{1–168}$ (orange). Bottom, Coomassie-stained gel showing the size-exclusion chromatography profile of MBP-CENP-$E_{2605–2701}$ *YF* (green) and MBP-CENP-$E_{2605–2701}$ *YF*/$PRC1_{1–168}$ (orange). No shift in the elution profile was observed.

B–E Characterization by isothermal titration calorimetry of binding between $PRC1_{1–168}$ and CENP-E peptides containing 1 motif, CENP-$E_{2605–2701}$, and GST-$Kif4A_{1133–1165}$. The *y*-axis indicates kcal/mole of injectant.

© 2023 The Authors

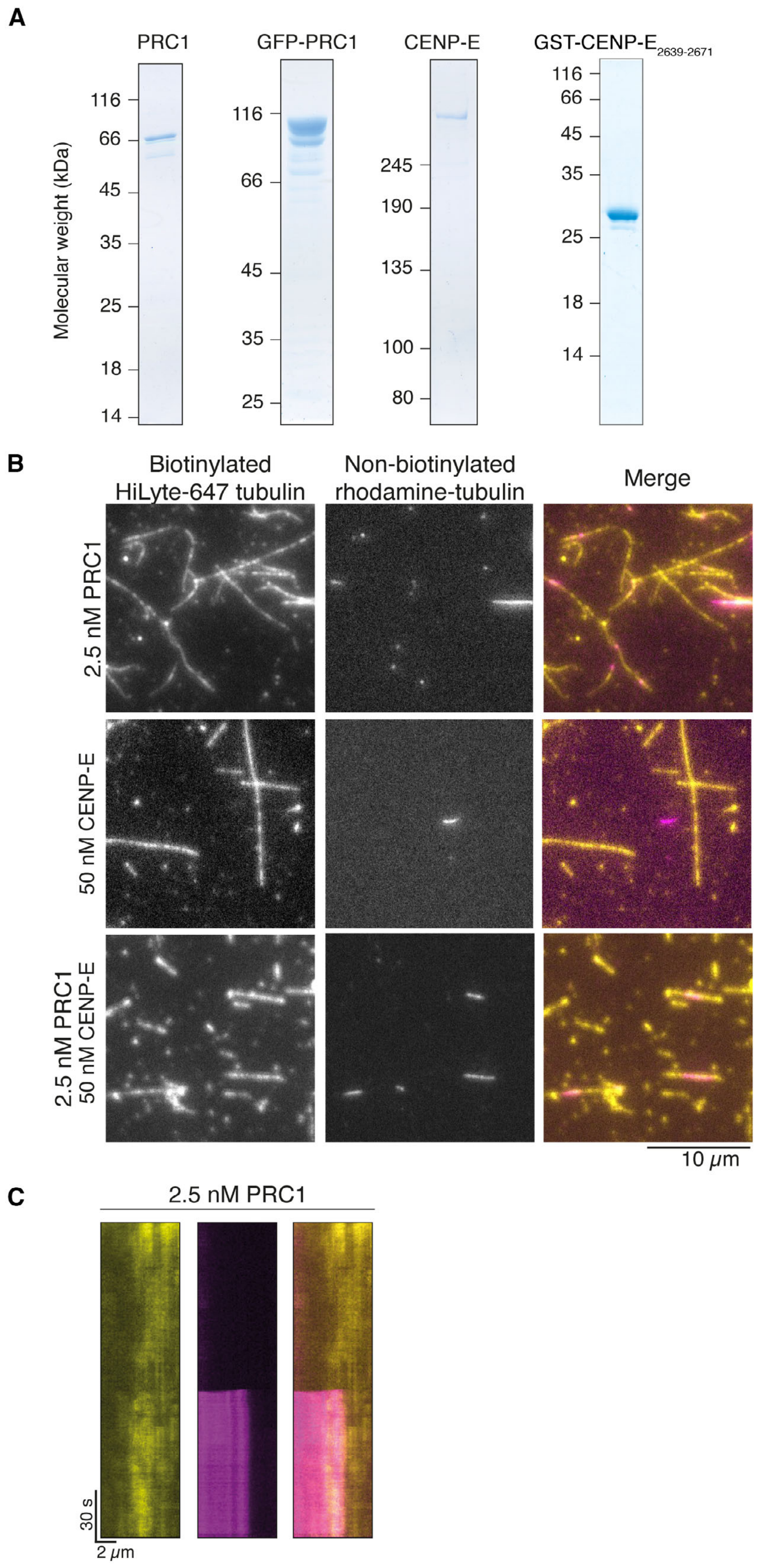

Figure EV2. Human CENP-E_{FL} does not bundle microtubules in the presence of ATP.

A Coomassie-stained gel showing purified His-PRC1, His-GFP-PFR1, full-length CENP-E and GST-CENP-$E_{2639–2671}$. (B) Bundling of microtubules by 2.5 nM PRC1 *in vitro*. Non-biotinylated rhodamine tubulin (pink) and biotinylated HiLyte-647 tubulin (yellow), scalebar 5 μm.

B Crosslinking of microtubules by 2.5 nM PRC1, 50 nM CENP-E_{FL} or both *in vitro*. In the absence of PRC1, rhodamine-labelled microtubules in solution (pink) are not crosslinked to biotinylated surface-bound Hilyte-647-labeled microtubules (yellow). Scalebar: 10 μm.

C Kymograph showing cross-linking of a free microtubule to an immobilized microtubule after approximately 15 s of imaging.

 © 2023 The Authors
