# Supplementary material for: Phosphorylation controls spatial and temporal activities of motor‐PRC1 complexes to complete mitosis
Source: EMBO J. 2023 Aug 18;42(21):e113647. doi: 10.15252/embj.2023113647 (PMC10620760; doi:10.15252/embj.2023113647)
Supplement: Supplementary file 2 — Movie EV1 [file EMBJ-42-e113647-s006.zip › movie EV1/Figure legend movie EMBO J.docx]

**Movie EV1:** **Microtubule sliding in the presence of full-length CENP-E and PRC1.**

Free microtubules (magenta) slide past surface-immobilized microtubules (yellow) in the presence of 2.5 nM PRC1 and 50 nM full-length CENP-E.
