# Supplementary material for: Phosphorylation controls spatial and temporal activities of motor‐PRC1 complexes to complete mitosis
Source: EMBO J. 2023 Aug 18;42(21):e113647. doi: 10.15252/embj.2023113647 (PMC10620760; doi:10.15252/embj.2023113647)
Supplement: Supplementary file 3 — PDF+ [file EMBJ-42-e113647-s001.pdf]

Article

SOURCE DATA TRANSPARENT PROCESS OPEN ACCESS

THE EMBO JOURNAL

Phosphorylation controls spatial and temporal activities of motor-PRC1 complexes to complete mitosis

Agata Gluszek-Kustusz[1], Benjamin Craske[1], Thibault Legal[1,2], Toni McHugh[1] & Julie PI Welburn[1,*]

Abstract

During mitosis, spindle architecture alters as chromosomes segregate into daughter cells. The microtubule crosslinker protein regulator of cytokinesis 1 (PRC1) is essential for spindle stability, chromosome segregation and completion of cytokinesis, but how it recruits motors to the central spindle to coordinate the segregation of chromosomes is unknown. Here, we combine structural and cell biology approaches to show that the human CENP-E motor, which is essential for chromosome capture and alignment by microtubules, binds to PRC1 through a conserved hydrophobic motif. This binding mechanism is also used by Kinesin-4 Kif4A: PRC1. Using *in vitro* reconstitution, we demonstrate that CENP-E slides antiparallel PRC1-crosslinked microtubules. We find that the regulation of CENP-E -PRC1 interaction is spatially and temporally coupled with relocalization to overlapping microtubules in anaphase. Finally, we demonstrate that the PRC1–microtubule motor interaction is essential in anaphase to control chromosome partitioning, retain central spindle integrity and ensure cytokinesis. Taken together our findings reveal the molecular basis for the cell cycle regulation of motor-PRC1 complexes to couple chromosome segregation and cytokinesis.

Keywords kinesin; microtubule; mitosis; phosphorylation; spindle
Subject Categories Cell Adhesion, Polarity & Cytoskeleton; Cell Cycle; Post-translational Modifications & Proteolysis
DOI 10.15252/embj.2023113647 | Received 31 January 2023 | Revised 30 June 2023 | Accepted 25 July 2023
The EMBO Journal (2023) e113647

Introduction

In cell division, the spindle has a crucial role in ensuring chromosomes are correctly partitioned into daughter cells. Antiparallel microtubules are essential for bipolar spindle stability during mitosis. In anaphase, sister chromatids are pulled to opposite poles. During that stage, re-modelling and elongation of the spindle reduces the risk of DNA damage to lagging chromosomes and aneuploidy, by moving chromosomes away from the cleavage plane. They are physically separated by the central spindle, or midzone, which is a stable structure of antiparallel microtubules that specifies the plane of division.

At the start of mitosis, CENP-E localizes to unattached kinetochores, where it associates with BubR1 and the outer corona (a fibrous expanded structure) of chromosomes (Yen *et al*, 1991; Cooke *et al*, 1997; Ciossani *et al*, 2018; Legal *et al*, 2020). Kinetochore-bound CENP-E enables kinetochore capture and lateral attachment to microtubules. CENP-E moves chromosomes along the spindle to the metaphase plate before kinetochore biorientation (Fig 1A, reviewed in Craske *et al*, 2022). The CENP-E motor relocalizes from kinetochores to the central spindle, at the metaphase to anaphase transition (Yao *et al*, 1997; Kurasawa *et al*, 2004). This relocalization is dependent on PRC1 (protein regulator of cytokinesis: 1) a non-motor microtubule-binding protein essential for central spindle assembly. CENP-E has a substantial role in chromosome alignment in early mitosis, but its PRC1-dependent recruitment from kinetochores to the central spindle, during the metaphase-to-anaphase transition, is less well understood. Depletion or inhibition of CENP-E results in accumulation of misattached polar chromosomes, and spindle checkpoint arrest in metaphase, making studies on CENP-E function in anaphase challenging (Schaar *et al*, 1997; Chan *et al*, 1998, 1999; Qian *et al*, 2010). Small molecule inhibition of CENP-E in anaphase and telophase results in spreading and delocalization of PRC1 on the central spindle. This observation led to a proposed role for CENP-E in organizing overlapping microtubules and the central spindle (Liu *et al*, 2020).

PRC1 is a dimeric, non-motor, microtubule-binding protein present on bundled spindle microtubules (Jiang *et al*, 1998; Kajtez *et al*, 2016). It then is enriched on the central spindle in anaphase and the midbody in telophase and is essential for the organization of the central spindle (Jiang *et al*, 1998; Mollinari *et al*, 2002; Verbrugghe & White, 2004). PRC1 preferentially binds to antiparallel overlapping microtubules (Bieling *et al*, 2010; Subramanian *et al*, 2010). Antiparallel microtubules compact to form a central spindle, facilitate chromosome separation and specify the division plane. The timing of PRC1 recruitment coincides with the rapid dephosphorylation of the proteome during the

1 Wellcome Centre for Cell Biology, School of Biological Sciences, University of Edinburgh, Edinburgh, UK
2 McGill University, Montreal, QC, Canada
*Corresponding author. Tel: +0131 650 7778; E-mail: julie.welburn@ed.ac.uk

© 2023 The Authors. Published under the terms of the CC BY 4.0 license.

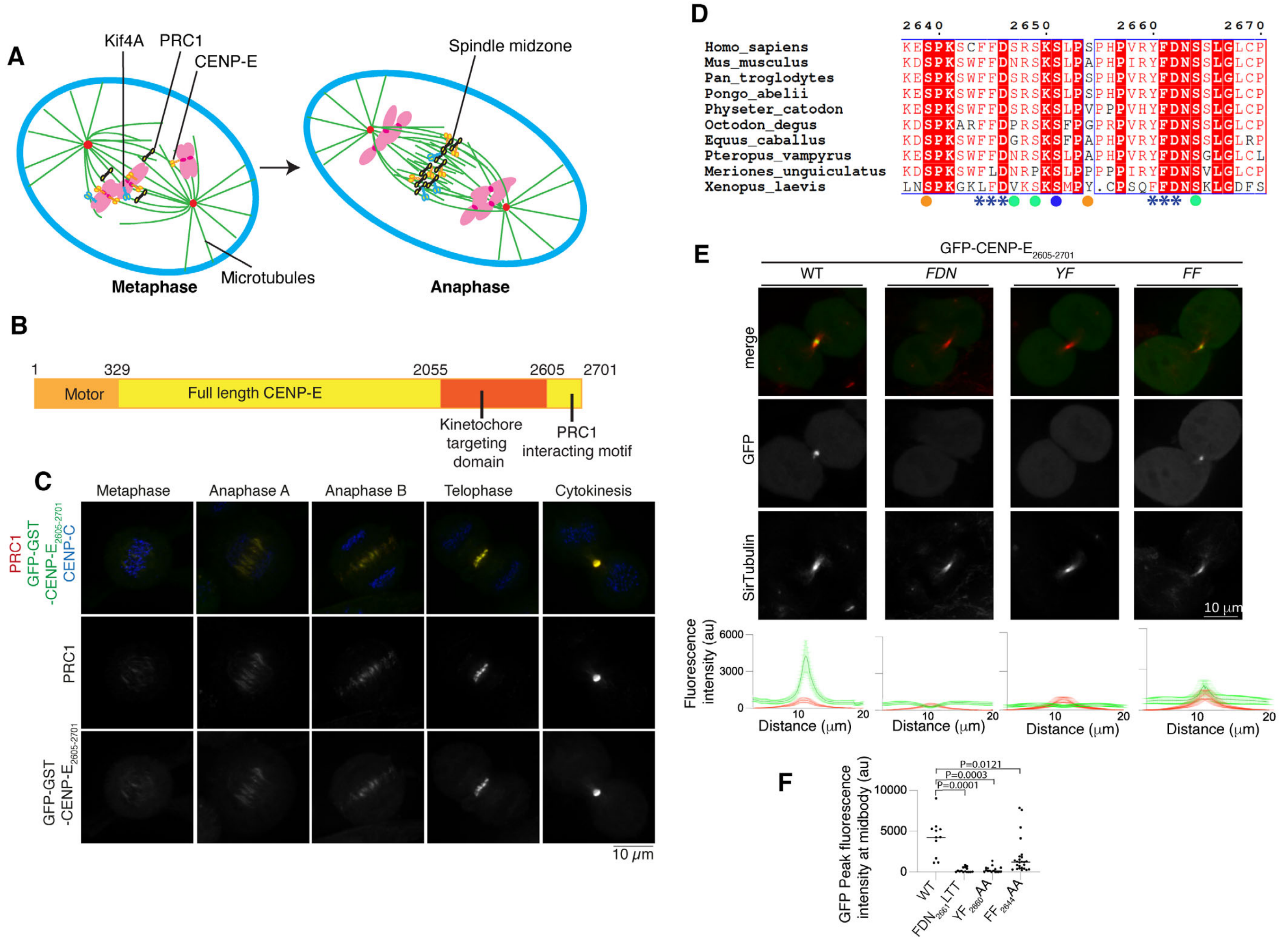

Figure 1. A hydrophobic motif is essential for recruitment of CENP-E to overlapping microtubules in mitosis.

A Schematic diagram showing the metaphase to anaphase transition, during which kinesin motors Kif4A (blue) and CENP-E (orange) relocalize from chromosomes and kinetochores (pink) to PRC1 (black) on crosslinked microtubules.

B Schematic diagram showing the different functional domains of full-length CENP-E, including the C terminus of CENP-E used in this study.

C Representative images of HeLa cells transiently transfected with GST-GFP-CENP-$E_{2605–2701}$ and immunostained with PRC1 and CENP-C, scale bar 10 μm (n = 4).

D Sequence alignment of the C terminus of human CENP-E with eight mammalian and *Xenopus laevis* CENP-E sequences. Amino acid numbering is relative to the human CENP-E sequence. The two PRC1 putative motifs ΦΦ are highlighted with asterisks (***). The following negatively charged amino acid is also highlighted. Phosphorylated residues following the CDK and Aurora kinase consensus sites are marked with an orange and blue circle respectively. Green circles represent sites that are phosphorylated but do not fit a kinase consensus site. The sequences were aligned using the program Clustal Omega (EBI) and formatted with ESPRIPT (Gouet *et al*, 1999).

E Top, Representative images of live HeLa cells in cytokinesis transiently transfected with GFP-CENP-$E_{2605–2701}$ and mutants (green), incubated with SiR-tubulin (red). Scale bar, 10 μm. Bottom, linescans showing the fluorescence intensity average and standard error of the mean (SEM) for the GFP-CENP-$E_{2605–2701}$ and mutants and tubulin across the cell midbody. For GFP-CENP-$E_{2605–2701}$, n is the number of cells. n = 11 and for the GFP-CENP-$E_{2605–2701}$ mutants, $FDN_{2661}LTT$, $YF_{2660}AA$ and $FF_{2644}AA$, n = 15, 16 and 25 respectively. Biological independent replicates were, respectively, 2, 3, 2 and 1.

F Graph showing the quantification of GFP peak fluorescence intensity for cells transfected with GFP-CENP-$E_{2605–2701}$ constructs and quantified in (E). Mean and peak intensities for each cell are represented for each mutant, ordinary one-way ANOVA test was performed to test significance.

Source data are available online for this figure.

metaphase-to-anaphase transition and is controlled by mitotic phosphorylation and dephosphorylation (Mollinari *et al*, 2002; Hu *et al*, 2012). Several motors—the Kinesin-4 Kif4A, Kif14A, MKLP1/Kif23 and CENP-E—are recruited to overlapping microtubules in a PRC1-dependent manner in anaphase, and interact with PRC1 in cell extracts (Kurasawa *et al*, 2004; Gruneberg *et al*, 2006; Glotzer, 2009; Douglas & Mishima, 2010; Hornick *et al*, 2010).

Association of PRC1 with motor proteins controls the organization and the length of microtubule overlaps in the central spindle (Kurasawa *et al*, 2004; Zhu & Jiang, 2005; Gruneberg *et al*, 2006; Lee *et al*, 2015; Poser *et al*, 2019). Interestingly, Kif4A is bound to chromatin in early mitosis. It relocalizes in anaphase to the PRC1-marked central spindle (Wang & Adler, 1995; Kurasawa *et al*, 2004). *In vitro*, PRC1 and Kif4A organize microtubules into bundles that resemble

© 2023 The Authors

overlapping microtubule arrays in the central spindle. Kif4A enables motorized sliding of microtubules past each other in the presence of PRC1 (Bieling *et al*, 2010; Subramanian *et al*, 2013; Hannabuss *et al*, 2019).

In order to understand how PRC1 recruits and interacts with motors to assemble the central spindle and enable the timely completion of mitosis, we combine cell biology and structural approaches to dissect the molecular mechanism of the PRC1-motor interaction, focusing on how CENP-E is recruited to PRC1 in anaphase, for which little is known. We show that human CENP-E interacts directly with PRC1 using a bipartite ΦΦ motif at their C terminus, and this mechanism is also used by Kif4A to bind PRC1. We applied AlphaFold 2 to identify a region of PRC1 that is predicted to bind to CENP-E and validated our predictions using site-directed mutagenesis. We used *in vitro* reconstitution and TIRF microscopy to show that full-length CENP-E slides PRC1-crosslinked microtubules past each other. We showed that the CENP-E:PRC1 interaction is spatially and temporally regulated by a phosphoswitch, which we propose, enables rapid relocalization of CENP-E from kinetochores to the central spindle during the metaphase-to-anaphase transition. Finally, we reveal a key role of motor-PRC1 interactions in the completion of mitosis.

Our findings provide a framework for understanding how phosphorylation controls the spatial and temporal activities of kinesin motors using CENP-E as a paradigm to enable completion of mitosis.

Results

The C terminus of CENP-E co-localizes with PRC1 throughout mitosis

CENP-E localizes to the central spindle and midbody in anaphase and telophase (Kurasawa *et al*, 2004). Previous work indicated that this process requires the C terminus of CENP-E and is dependent on PRC1 (Fig 1B; Ohashi *et al*, 2016). We hypothesized that the function of CENP-E in anaphase is independent of its kinetochore function. We have previously mapped the kinetochore-targeting domain (a.a. 2055–2608; Chan *et al*, 1998; Legal *et al*, 2020). Thus, we hypothesized the region of CENP-E that targets the central spindle and PRC1 would be the region C-terminal to the kinetochore-targeting domain.

In order to mimic full-length CENP-E which is homodimeric, we dimerized CENP-$E_{2605–2701}$ by fusing a GST-tag N-terminal to CENP-$E_{2605–2701}$, as previously reported (Legal *et al*, 2020). To test whether the C-terminal fragment of CENP-$E_{2605–2701}$ localized to overlapping microtubules in the central spindle during anaphase, and to the midbody during telophase when PRC1 is present, we imaged GST-GFP-CENP-$E_{2605–2701}$ in mitosis. We found GST-GFP-CENP-$E_{2605–2701}$ weakly co-localized with PRC1 to the centre of the metaphase spindle, and more strongly localized to PRC1-crosslinked microtubule bundles on the central spindle and midbody (Fig 1C). This is similar to the localization of full-length CENP-E in anaphase and telophase (Kurasawa *et al*, 2004).

A conserved CENP-E motif is required for PRC-1 binding

An alignment of full-length CENP-E from nine mammalian species and *Xenopus laevis* revealed high levels of sequence conservation in the last 100 amino acids of CENP-E, C-terminal to the kinetochore-targeting domain (Fig 1B and D). This C-terminal domain contains the sequence motif RYFDNSSL (amino acids 2659–2666), which was previously reported to be essential for localization of CENP-E to the midbody. The localization of the CENP-E C terminus is also dependent on PRC1 (Ohashi *et al*, 2016).

We mutated the strongly conserved residues FDN (F2661, D2662, N2663) or YF (Y2660, F2661) to LTT and AA respectively. We then imaged cells in mitosis transiently expressing wild-type or mutant GFP-CENP-$E_{2605–2701}$ using live-cell imaging to preserve dynamic interactions. GFP-CENP-$E_{2605–2701}$ did not display any dominant negative phenotype, unlike the CENP-E kinetochore-targeting domain (Legal *et al*, 2020). Microtubules were stained with the SiR-tubulin dye, well suited for live-cell imaging. Both GFP-CENP-$E_{2605–2701}$ mutants failed to localize to the central spindle (Fig 1E). Interestingly, we also observed a similar, highly conserved (FFD) motif upstream, at positions 2644–2646 (Fig 1D). To test the contribution of these two motifs to CENP-E recruitment to the central spindle, we generated a series of mutations in CENP-E that had altered motifs (either one motif, or the other, or both were mutagenized—alone or in tandem, Table 1). Mutation $_{2644}FF_{2645}$ to AA caused a significant reduction in the localization of CENP-E to the midbody, whereas mutation of $_{2660}YF_{2661}$ to AA completely abolished CENP-E localization to the midbody (Fig 1E and F). We concluded that the second motif present in CENP-E, $_{2660}YF_{2661}$, is essential for midbody localization, and that the upstream $_{2644}FF_{2645}$ motif strongly contributes to targeting to the midbody. Taken together, our data reveal that the hydrophobic motif, which we describe as ΦΦ motif, is essential for CENP-E recruitment to overlapping microtubules, and to PRC1 *in vivo*.

CENP-E binds PRC1 *in vitro*

Next, we tested whether CENP-E and PRC1 interact *in vitro*. We expressed the C terminus of CENP-E as a monomeric MBP fusion, named MBP-CENP-$E_{2605–2701}$ and a $PRC1_{1–168}$ fragment, previously reported to bind to Kif4A (Subramanian *et al*, 2013; Table 1). After mixing them together in an equimolar ratio and carrying out size-exclusion chromatography and SDS–PAGE analysis, we observed co-migration, indicating that these proteins assemble into a complex in solution (Fig 2A). We hypothesized that disruption of the second motif, which is essential for CENP-E recruitment to the central spindle, might abrogate the interaction of CENP-E with PRC1. In order to test this, we purified MBP-CENP-$E_{2605–2701}$*FDN and* MBP-CENP-$E_{2605–2701}$*YF*. Size-exclusion chromatography of MBP-CENP-$E_{2605–2701}$*FDN* mixed with $PRC1_{1–168}$ in a 1:1 molar ratio followed by SDS–PAGE analysis revealed that the two proteins no longer interacted (Fig 2B). We observed the same results with size-exclusion chromatography of $PRC1_{1–168}$ and MBP-CENP-$E_{2605–2701}$*YF* (Fig EV1A). This confirms that the region flanking the second ΦΦ motif is essential for PRC1-CENP-E to interact *in vitro* (Figs 2B and EV1A).

PRC1 binds dimeric the CENP-E C terminus with high affinity

In cell extracts, PRC1 interacts with Kif4A, Kif14, MKLP1 and CENP-E (Kurasawa *et al*, 2004; Gruneberg *et al*, 2006). The PRC1-Kif4A interaction has been reconstituted *in vitro* (Bieling *et al*, 2010; Subramanian *et al*, 2013) but the mechanism underlying the

© 2023 The Authors

Table 1. Constructs generated in this study.

		Construct	Vector	Expression
pAG77	GFP-CENP-E$_{2605-2701}$	GFP-CENP-E 2605–2701	pBabe	Human
pAG78	MBP-CENP-E$_{2605-2701}$	MBP-CENP-E 2605–2701	pMALC2X	Bacteria
pAG84	MBP-CENP-E$_{2605-2701}$ *FDN*	MBP-CENP-E 2605–2701 F2661L, D2662T, N2663T	pMALC2X	Bacteria
pAG85	GFP-CENP-E$_{2605-2701}$ 2SD	GFP-CENP-E 2605–2701 S2639D S2654D	pBabe	Human
pAG86	GFP-CENP-E$_{2605-2701}$ *FDN*	GFP-CENP-E 2605–2701 F2661L, D2662T, N2663T	pBabe	Human
pAG87	GFP-CENP-E$_{2605-2701}$ 2SA	GFP-CENP-E 2605–2701 S2639A S2654A	pBabe	Human
pAG92	GFP-CENP-E$_{2605-2701}$ *YF*	GFP-CENP-E 2605–2701 Y2660A F2661A	pBabe	Human
pAG94	GFP-CENP-E$_{2605-2701}$ *FF*	GFP-CENP-E 2605–2701 F2644A F2645A	pBabe	Human
pAG95	GST-CENP-E$_{2605-2701}$	GST-CENP-E 2605–2701	pGEX6p1	Bacteria
pAG96	GST-CENP-E$_{2605-2701}$ 2SD	GST-CENP-E 2605–2701 S2639D S2654D	pGEX6p1	Bacteria
pAG100	GFP-GST-CENP-E$_{2605-2701}$	GFP-GST-CENP-E 2605–2701	pBabe	Human
pAG101	GFP-GST-CENP-E$_{2605-2701}$ 2SD	GFP-GST-CENP-E 2605–2701 S2639D S2654D	pBabe	Human
pAG102	GFP-GST-CENP-E$_{2605-2701}$ 2SA	GFP-GST-CENP-E 2605–2701 S2639A S2654A	pBabe	Human
pAG116	GST-CENP-E$_{2605-2701}$ 6SD	GST-CENP-E 2605–2701 S2639D S2647D S2469D S2651D S2654D S26646D	pGEX6p1	Bacteria
pAG118	GST-CENP-E$_{2639-2671}$	GST-CENP-E 2639–2671	pGEX6p1	Bacteria
pAG119	GFP-GST-CENP-E$_{2605-2701}$ 6SD	GFP-GST-CENP-E 2605–2701 S2639D S2647D S2469D S2651D S2654D S26646D	pBabe	Human
pAG120	GFP-GST-CENP-E$_{2605-2701}$ 6SA	GFP-GST-CENP-E 2605–2701 S2639A S2647A S2469A S2651A S2654A S2664A	pBabe	Human
pAG121	GST-Kif4A$_{1133-1165}$	GST-Kif4A 1133–1165	pGEX6p1	Bacteria
pAG127	GST-Kif4A$_{1133-1232}$	GST-Kif4A 1133–1232	pGEX6p1	Bacteria
pAG122	GFP-GST-Kif4A$_{1133-1165}$	GFP-GST-Kif4A 1133–1165	pBabe	Human
pAG128	GFP-GST-Kif4A$_{1133-1232}$	GFP-GST-Kif4A 1133–1232	pBabe	Human
pAG129	GFP-PRC1 WT	GFP-PRC1 sgRNA resistant	pBabe	Human
pAG132	GFP-PRC1 *MEE*	GFP-PRC1 M54A E57A E58A sgRNA resistant	pBabe	Human
	His-PRC1 1–168 *MEE*	His-PRC1 *MEE* 1–168 M54A E57A E58A	pET Duet 1	Bacteria
	His-PRC1 *MEE*	His-SNAP-TEV-PRC1 *MEE* M54A E57A E58A	pET Duet 1	Bacteria

interaction of PRC1 with kinesin motors has not been reported. Hence, we sought to delineate the mechanism by which PRC1 binds to CENP-E by quantifying the affinity of CENP-E for PRC1$_{1-168}$ (Fig 2A). First, we measured the affinity of the CENP-E peptides containing the two ΦΦ motifs for PRC1$_{1-168}$ separately to understand the contributions of these two sites. Isothermal calorimetry (ITC) measurement of CENP-E peptide 1, containing the first ΦΦ motif (PKSC$_{2644}$FF$_{2645}$DSRSK), and peptide 2, containing the second ΦΦ motif (PVR$_{2660}$YF$_{2661}$DNSSLG) affinity for PRC1$_{1-168}$ revealed a very weak affinity of each peptide for PRC1 (Figs 2C, and EV1B and C). Next, we determined the affinity of the 98 amino acid C-terminal fragment of CENP-E (CENP-E$_{2605-2701}$), which contains both ΦΦ motifs in tandem in their native CENP-E sequence (Fig EV1D) with PRC1$_{1-168}$. We measured an affinity of 19.3 μM (Fig 2C). The C-terminal domain of CENP-E is predicted to be disordered using AlphaFold2 (Jumper *et al*, 2021), so binding to PRC1 would likely involve a large entropic penalty associated with a reduction in conformational flexibility in the protein upon binding to PRC1. Overall, this interaction is relatively weak. However, CENP-E and PRC1 are both dimers *in vivo*. So, in order to more closely represent the

Figure 2. CENP-E interacts with PRC1 through a kinesin ΦΦ motif.

A Top. Size-exclusion chromatography elution profile of MBP-CENP-E$_{2605-2701}$ (green), PRC1$_{1-168}$ (pink) and MBP-CENP-E$_{2605-2701}$/PRC1$_{1-168}$ (orange). Bottom, Coomassie-stained gel showing the size-exclusion chromatography profile of PRC1$_{1-168}$ (pink), MBP-CENP-E$_{2605-2701}$ (green) and MBP-CENP-E$_{2605-2701}$/PRC1$_{1-168}$ (orange). A shift in the elution volume was only seen in the presence of both CENP-E and PRC1.

B Top. Size-exclusion chromatography elution profile of MBP-CENP-E$_{2605-2701}$*FDN* (green) and MBP-CENP-E$_{2605-2701}$ *FDN*/PRC1$_{1-168}$ (orange). Bottom, Coomassie-stained gel showing the size-exclusion chromatography profile of MBP-CENP-E$_{2605-2701}$*FDN* (green) and MBP-CENP-E$_{2605-2701}$ *FDN*/PRC1$_{1-168}$ (orange). No shift in the elution profile was observed.

C Table summarizing the isothermal titration calorimetry (ITC) results measuring the dissociation constant Kd for the PRC1$_{1-168}$/CENP-E C-terminal interaction, using various C-terminal peptides.

D Characterization by ITC of the PRC1$_{1-168}$/GST-CENP-E$_{2605-2701}$ interaction. Bottom. Top DP is the differential power and ΔH is the enthalpy.

Source data are available online for this figure.

 © 2023 The Authors

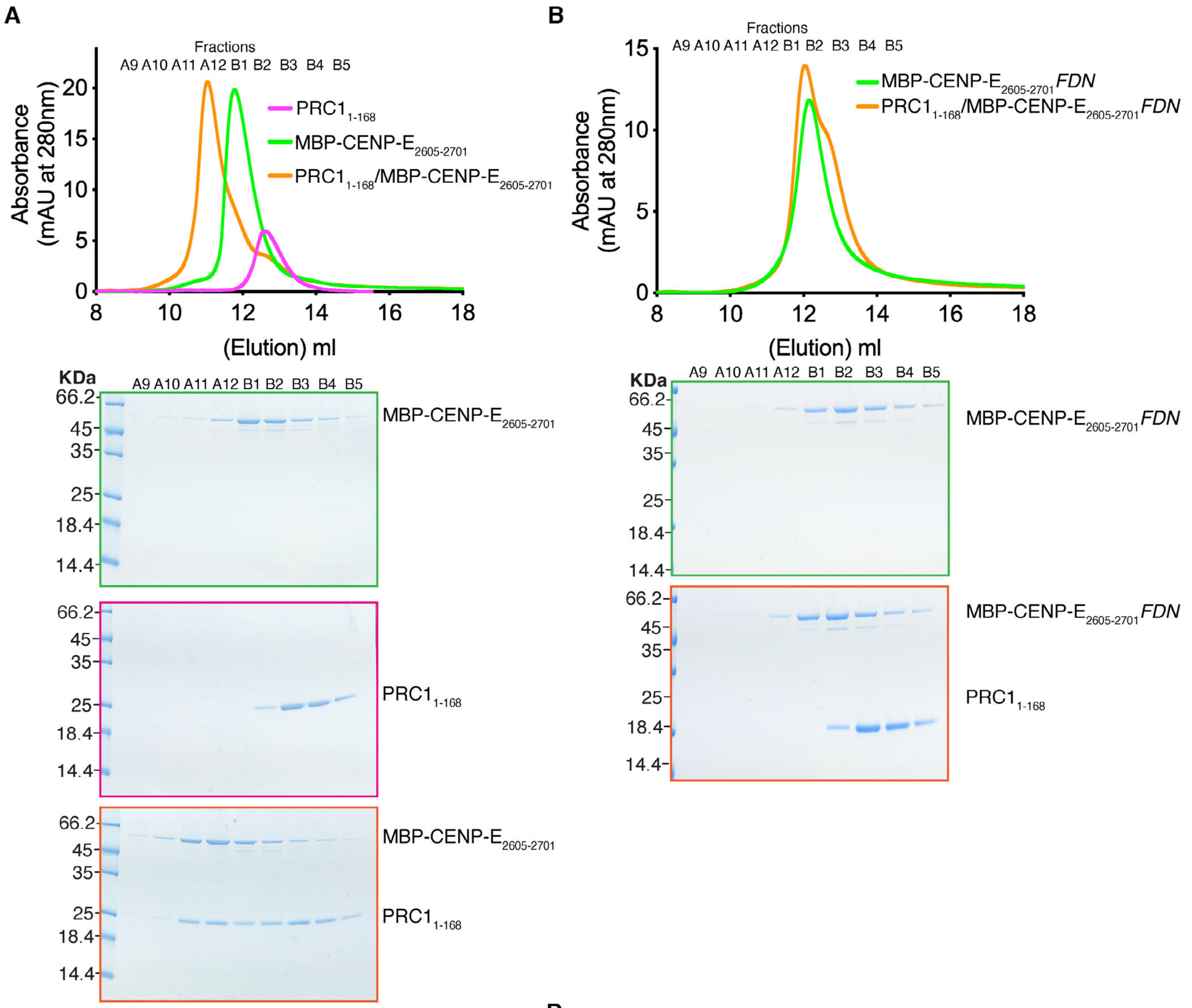

C

Construct	Kd (μM)
CENP-E peptide 1: PKSCFFDSRSK	41.1±21
CENP-E peptide 2: PVRYFDNSSLG	64.0±16
CENP-E$_{2605\text{-}2701}$	19.3±1.8
GST-CENP-E$_{2605\text{-}2701}$	1.5±0.05

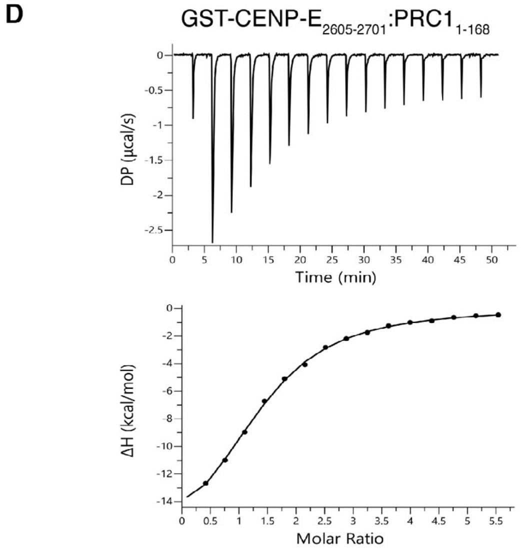

Figure 2.

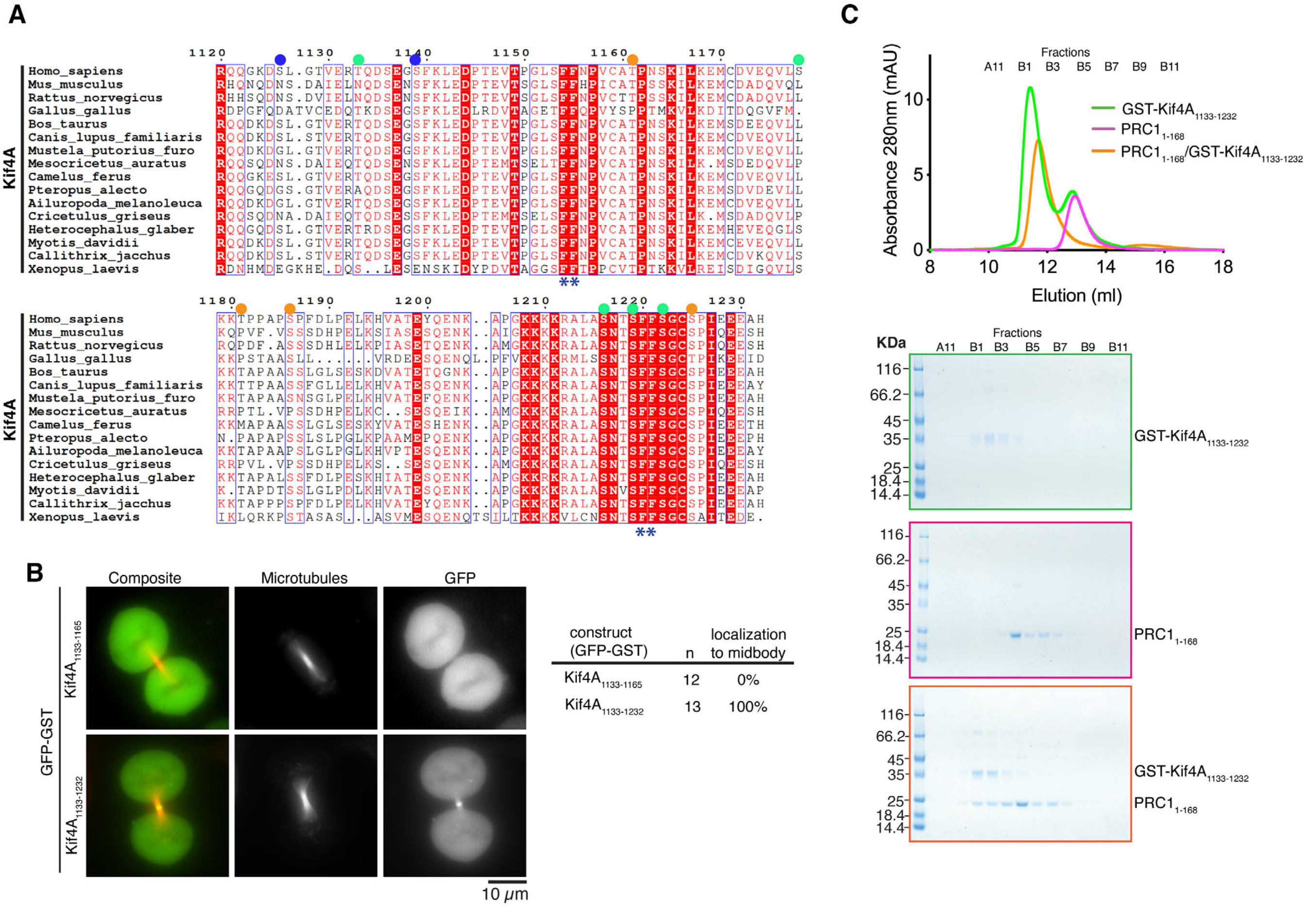

Figure 3. Kif4A binds to PRC1 using a bipartite ΦΦ motif.

A Sequence alignment of the C terminus of human Kif4A with Kif4A of other metazoans. Amino acid numbering is relative to the human Kif4A sequence. The two PRC1 putative motifs ΦΦ are highlighted with asterisks (*). Published phosphorylated residues are marked. Those following the CDK and Aurora kinase consensus sites are marked with an orange and blue circle respectively. Green circles represent sites that are phosphorylated but do not fit a kinase consensus site. The sequences were aligned using the program Clustal Omega (EBI) and formatted with ESPRIPT (Gouet *et al*, 1999).

B Representative images of live HeLa cells in mitosis transiently transfected with either GFP-Kif4A$_{1133–1165}$ or GFP-Kif4A$_{1133–1232}$ incubated with SiR-Tubulin. Scale bar, 10 μm. Quantification of cells with GFP-Kif4A$_{1133–1165}$ (n = 12) or GFP-Kif4A$_{1133–1232}$ (n = 13) localization to the central spindle. Data are represented from two independent experiments.

C A shift in the elution volume was only seen in the presence of both Kif4A and PRC1. Size-exclusion chromatography elution profile of GST-Kif4A$_{1133–1232}$ (green) and PRC1$_{1–168}$ (pink), or together (orange). Bottom, Coomassie-stained gel showing the size-exclusion chromatography profile of GST-Kif4A$_{1133–1232}$ (green), PRC1$_{1–168}$ (pink) and GST-Kif4A$_{1133–1232}$/PRC1$_{1–168}$ (orange).

Source data are available online for this figure.

in vivo interaction, we purified GST-CENP-E$_{2605–2701}$ which is dimeric and measured the affinity of GST-CENP-E$_{2605–2701}$ for PRC1$_{1–168}$ using ITC (Fig 2C and D). Dimerization led to a 12-fold increase in affinity between GST-CENP-E$_{2605–2701}$ for PRC1$_{1–168}$ to 1.5 μM, similar to the binding affinity reported for the Kif4A:PRC1 measured using binding assays (Subramanian *et al*, 2013). Overall, these data indicate multiple ΦΦ motifs increase the affinity of the CENP-E:PRC1 interaction through an avidity effect.

Kif4A requires a bipartite ΦΦ motif for PRC1 binding

Kif4A also contains a phenylalanine ΦΦ motif (F1154, F1155) essential for targeting the central spindle and PRC1 binding (Poser *et al*, 2019). It is reminiscent of the CENP-E motif (Fig 3A), although no aspartate follows the ΦΦ motif. We expressed the 32-amino-acid region of Kif4A containing the ΦΦ motif as a GST fusion (GST-Kif4A$_{1133–1165}$; Table 1). Unlike the C terminus of CENP-E, GST-Kif4A$_{1133–1165}$ did not have any affinity for PRC1, as measured by ITC (Fig EV1E). *In vivo*, GFP-GST-Kif4A$_{1133–1165}$ did not localize to overlapping microtubules (Fig 3B). We then searched for a second motif that could increase Kif4A binding to PRC1, similarly to CENP-E. There is a second ΦΦ motif in Kif4A (F1220, F1221) downstream of the published PRC1-binding region (F1154, F1155) (Fig 3A). We hypothesized that this second motif might contribute to the PRC1-Kif4A interaction, and that the ΦΦ motif (F1154, F1155) is essential but not sufficient for PRC1 binding, in common with CENP-E (Fig EV1). To test whether Kif4A and CENP-E bind PRC1 using a similar mechanism, we expressed a dimeric fragment of Kif4A that contains both

 © 2023 The Authors

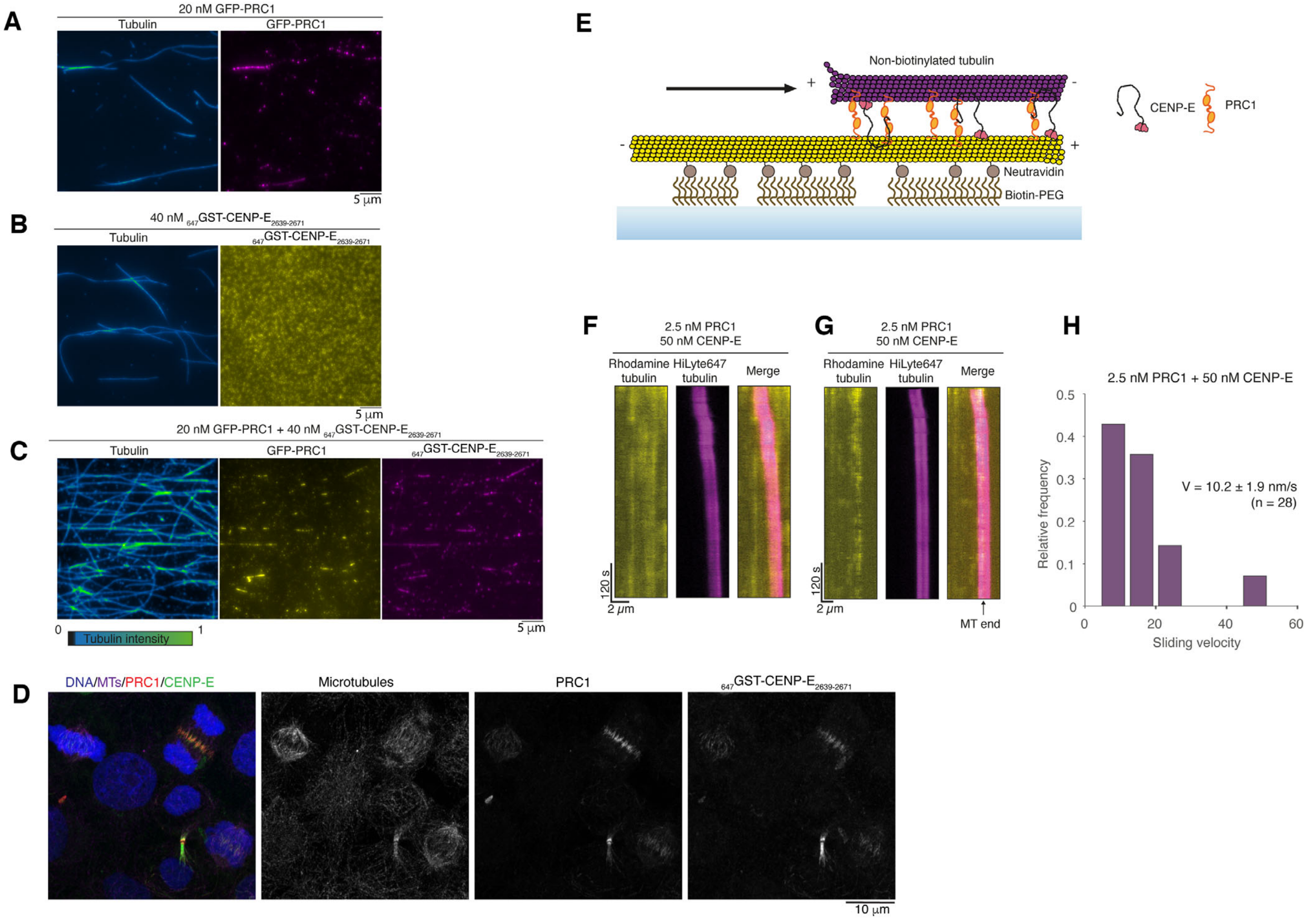

Figure 4. CENP-E slides anti-parallel microtubules in the presence of PRC1.

- A Representative images of GFP-PRC1 (magenta) mixed with rhodamine microtubules. Fire blue-green intensity LUT is used to show tubulin intensity and microtubule overlaps.
- B Representative images of $_{647}$GST-CENP-E$_{2639–2671}$ (yellow) mixed with rhodamine microtubules.
- C Representative images of GFP-PRC1 and GST-CENP-E$_{2639–2671}$ mixed with rhodamine microtubules.
- D Representative immunofluorescence images of HeLa cells stained for DNA, microtubules, PRC1 and with Alexa647-labeled GST-CENP-E$_{2639–2671}$ showing it also recognizes PRC1 in cells. Experiments were repeated > 3 times.
- E Schematic representation of a microtubule sliding assay in a reconstituted system. The biotinylated microtubule, immobilized to the surface, is represented in yellow. The free microtubule is represented in magenta.
- F Representative kymograph showing microtubule–microtubule sliding in the presence of 2.5 nM PRC1 and 50 nM full-length CENP-E.
- G Example kymograph showing a free microtubule sliding until reaching the end of the immobilized microtubule where it slows down to a stall.
- H Graph showing the quantification of microtubule–microtubule sliding velocity exhibited by free microtubules transported in the presence of 2.5 nM PRC1 and 50 nM full-length CENP-E, n = 28, with median velocity and standard error reported. Data are represented from two independent experiments.

Source data are available online for this figure.

ΦΦ motifs, GFP-GST-Kif4A$_{1133–1232}$ and showed that it localizes to overlapping microtubules (Fig 3B). *In vitro*, GST-Kif4A$_{1133–1232}$ and PRC1 co-eluted as a complex using SEC (Fig 3C). Together, these data indicate that while the previously reported Kif4A ΦΦ motif is necessary to bind PRC1 (Poser *et al*, 2019), it is not sufficient. Similar to CENP-E, Kif4A uses a bipartite motif to stably bind PRC1.

A CENP-E-PRC1 complex slides microtubules

CENP-E has been proposed to slide microtubules in mitosis using its non-motor microtubule=binding domain (Steblyanko *et al*, 2020). It is also possible that CENP-E slides antiparallel microtubules that are crosslinked by PRC1, similar to Kif4A (Bieling *et al*, 2010; Subramanian *et al*, 2013). To distinguish between these two models, we carried out an *in vitro* reconstitution experiment (Fig 4). We previously reconstituted motility of both truncated and full-length CENP-E *in vitro* (Craske *et al*, 2022). The challenge in analysing the contribution of CENP-E to microtubule sliding is that about 10% purified full-length CENP-E is motile, with the long coiled-coil stalk interfering with its activity (Craske *et al*, 2022). In order to determine whether CENP-E slides microtubules alone, or only slides those crosslinked by PRC1, we first analysed whether CENP-E was

© 2023 The Authors

recruited to microtubules crosslinked with PRC1, or to PRC1 directly (Fig EV2A). As full-length CENP-E is challenging to work with owing to its instability, we purified a minimal PRC1-binding CENP-E construct, GST-CENP-$E_{2639–2671}$ (Table 1 and Fig EV2A) and chemically labelled this protein with an Alexa Fluor-647 dye. To test if PRC1 is able to recruit the CENP-E C terminus to microtubules, polymerized GMPCPP-stabilized rhodamine microtubules were mixed with $_{647}$GST-CENP-$E_{2639–2671}$ alone, GFP-PRC1 alone or both $_{647}$GST-CENP-$E_{2639–2671}$ and GFP-PRC1. These samples were then added to silanized coverslips that were coated with anti-tubulin antibodies (Fig 4A–C). When we added GFP-PRC1- to GMPCCP-stabilized rhodamine microtubules in a flow chamber, GFP-PRC1 decorated the length of the microtubule but was preferentially recruited to overlapping regions between two or more microtubules (Fig 4B). In the presence of GFP-PRC1, $_{647}$GST-CENP-$E_{2639–2671}$ bound specifically to PRC1 at overlapping microtubules. These results indicate that the C terminus of CENP-E specifically binds to PRC1 rather than to microtubules (Fig 4C). $_{647}$GST-CENP-$E_{2639–2671}$ also recognizes and stains endogenous PRC1 in cells (Fig 4D).

Next, we analysed whether full-length human CENP-E could slide microtubules apart in the presence of PRC1 *in vitro*. We incubated surface-immobilized microtubules with full-length human PRC1 to allow coating of the microtubule lattice with PRC1, then added GMPCPP-stabilized rhodamine microtubules, which led to microtubule bundling (Fig 4E). When 2.5 nM PRC1 alone was added, pairs of overlapping microtubules formed (Fig EV2B). Overlaps remained constant over time, and no sliding of microtubules was observed throughout the experiment lasting 20 min (Fig EV2C). In contrast, we did not observe overlapping microtubule pairs when we added 50 nM full-length CENP-E, ATP and microtubules (Fig EV2B). When we added 50 nM CENP-E and 2.5 nM PRC1, free microtubules were crosslinked and transported unidirectionally along the coverslip-immobilized microtubules (Figs 4F and G, and EV2B, Movie EV1). CENP-E-driven microtubule sliding was slow, with an average velocity of 10.4 ± 2.5 nm/s (Fig 4H). This sliding velocity is comparable to that of Kif4A, around 11 nm/s in the presence of 1 nM PRC1 (Wijeratne & Subramanian, 2018). Slowing down of microtubule sliding over time was observed (Fig 4F and G).

Together, these data suggest that a CENP-E-PRC1 complex is able to slide antiparallel microtubules relative to each other. The sliding velocity may be regulated by frictional forces that are either generated by the accumulation over time of PRC1 on cross-linked microtubules, similar to Kif4A/PRC1 sliding (Wijeratne & Subramanian, 2018), or by the fraction of inactive or paused microtubule-bound CENP-E motors that can still bind to PRC1 (Craske *et al*, 2022).

Phosphorylation of CENP-E controls its PRC1 microtubule-binding activity

CENP-E is strongly recruited to overlapping antiparallel microtubules, crosslinked by PRC1 in anaphase. Before anaphase, CENP-E is primarily localized to unattached kinetochores in prometaphase and remains localized to kinetochores in smaller amounts in metaphase. We hypothesized that the interaction between PRC1 and CENP-E might be regulated by post-translational modifications to enable rapid temporal and spatial relocalization of CENP-E from kinetochores to PRC1-bound microtubules in the central spindle. Of note, mitotic kinase activity is high in prometaphase, contributed by CDK, Aurora, Mps1 and Plk1 kinases. In particular, CDK activity drops dramatically during the metaphase-to-anaphase transition. Multiple phosphoproteomic studies have previously reported that the C terminus of CENP-E is phosphorylated in mitosis and identified the phosphorylated residues *in vivo* (Nousiainen *et al*, 2006; Dephoure *et al*, 2008; Malik *et al*, 2009; Kettenbach *et al*, 2011; Santamaria *et al*, 2011; Sharma *et al*, 2014). We noted six of these phosphorylated residues were close to the PRC1-binding motif (Fig 1D). Two serines phosphorylated at positions 2639 and 2654 fit the CDK consensus site (S/T–P) and a serine 2651 phosphorylated by the Aurora kinases close to the FF motifs was reported multiple times (Nousiainen *et al*, 2006; Kettenbach *et al*, 2011; Santamaria *et al*, 2011; Sharma *et al*, 2014; Fig 1D). S2647, S2649 and S2664 were also reported as phosphosites (Sharma *et al*, 2014).

In order to test whether phosphorylation of the C terminus of CENP-E affects its interaction with PRC1, we generated phosphomimetic (amino acid substitutions that mimic a phosphorylated version of the amino acid) mutants of GST-CENP-$E_{2605–2701}$. We mutated S2639 and S2654 to generate GST-CENP-$E_{2605–2701}$ 2SD (mimicking two phosphorylated amino acids), and S2639, S2647, S2649, S2651, S2654 and S2664 for GST-CENP-$E_{2605–2701}$ 6SD (mimicking six phosphorylated amino acids), and measured their affinity for PRC1 using ITC. There was a small decrease in affinity of GST-CENP-$E_{2605–2701}$ 2SD for $PRC1_{1–168}$. The Kd increased from 1.5 μM for control versus 2.4 μM for 2SD (Fig 5A). GST-CENP-$E_{2605–2701}$ 6SD displayed no binding to PRC1 (Fig 5B and C). Further phosphorylation of CENP-E could also contribute to reducing the PRC1: CENP-E interaction *in vivo*.

Next, we analysed how phosphorylation of the C terminus of CENP-E affected association with PRC1 at overlapping microtubules in cells. We examined the localization of the CENP-E C terminus in metaphase (Fig 5D). GFP-CENP-$E_{2605–2701}$ was mostly cytoplasmic, but dimeric GFP-GST-CENP-$E_{2605–2701}$ (to mimic full-length CENP-E, which functions as a dimer *in vivo* and would be representative of CENP-E), and was enriched on interpolar overlapping microtubules, close to the chromosomes. GST-CENP-$E_{2605–2701}$ was not observed uniformly on microtubules, indicating it is unlikely to bind microtubules directly (Fig 5D). We observed GFP-GST-CENP-$E_{2605–2701}$ 2SD localized weakly to interpolar microtubules, but we did not observe association of GFP-CENP-$E_{2605–2701}$ 2SD and GFP-GST-CENP-$E_{2605–2701}$ 6SD with microtubules. We frequently observed GFP-CENP-$E_{2605–2701}$ 2SA, albeit in small amounts (weak fluorescence) on interpolar microtubules in metaphase. GFP-GST-CENP-$E_{2605–2701}$ 2SA, which is dimeric, was observed on interpolar microtubules, and GFP-GST-CENP-$E_{2605–2701}$ 6SA was also enriched there (Fig 5D).

Next, we examined the localization of GFP-GST-CENP-$E_{2605–2701}$ at the midbody in telophase. GFP-GST-CENP-$E_{2605–2701}$ and GFP-GST-CENP-$E_{2605–2701}$ 6SA were present at the midbody but GFP-GST-CENP-$E_{2605–2701}$ 6SD did not associate with the midbody, similar to our observations with the GFP-CENP-$E_{2605–2701}$ *YF* (Fig 5E–G).

We surmise that phosphorylation of the CENP-E C terminus prevents association with PRC1. Taken together these data reveal that phosphorylation of the C terminus of CENP-E inhibits recruitment to PRC1 at overlapping microtubules in early mitosis, by reducing the affinity of CENP-E for PRC1. The phosphorylation state of CENP-E during mitosis therefore regulates its interactions, both spatially and temporally, to enable CENP-E to associate with the outer corona of kinetochores in early mitosis, where it mediates chromosome

© 2023 The Authors

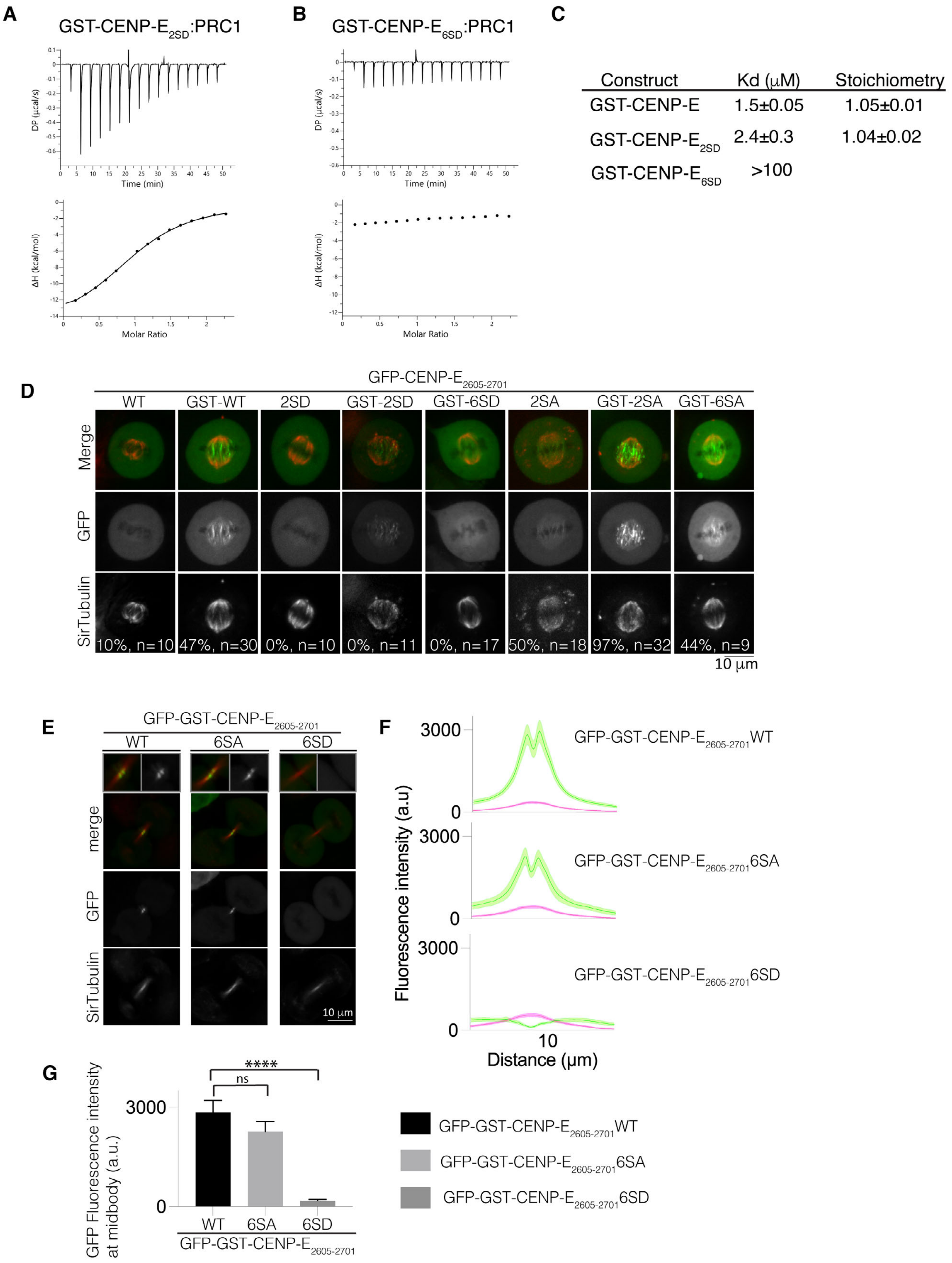
A
GST-CENP-E2SD:PRC1
DP (μcal/s)
Time (min)
ΔH (kcal/mol)
Molar Ratio
B
GST-CENP-E6SD:PRC1
C
Construct Kd (μM) Stoichiometry
GST-CENP-E 1.5±0.05 1.05±0.01
GST-CENP-E2SD 2.4±0.3 1.04±0.02
GST-CENP-E6SD >100
D
GFP-CENP-E2605-2701
WT GST-WT 2SD GST-2SD GST-6SD 2SA GST-2SA GST-6SA
Merge
GFP
SirTubulin
10%, n=10 47%, n=30 0%, n=10 0%, n=11 0%, n=17 50%, n=18 97%, n=32 44%, n=9
10 μm
E
GFP-GST-CENP-E2605-2701
WT 6SA 6SD
merge
GFP
SirTubulin
10 μm
F
Fluorescence intensity (a.u)
GFP-GST-CENP-E2605-2701WT
GFP-GST-CENP-E2605-2701 6SA
GFP-GST-CENP-E2605-2701 6SD
Distance (μm)
G
GFP Fluorescence intensity at midbody (a.u.)
****
ns
WT 6SA 6SD
GFP-GST-CENP-E2605-2701

Figure 5.

Figure 5. Regulation by phosphorylation of the PRC1-CENP-E interaction.

A, B Characterization by isothermal titration calorimetry (ITC) of binding between PRC1$_{1–168}$ and GST-CENP-E$_{2605–2701}$ 2SD (A) and GST-CENP-E$_{2605–2701}$ 6SD (B). The *y*-axis indicates kcal/mole of injectant.

C Table summarizing the affinity of the CENP-E constructs for PRC1$_{1–168}$.

D Live-cell imaging of metaphase spindles in HeLa cells transiently transfected wild-type, phosphomimetic and non-phosphorylatable mutants of GFP-CENP-E$_{2605–2701}$ (monomeric) and GFP-GST-CENP-E$_{2605–2701}$ (dimeric) and stained for tubulin using SiR-Tubulin. The fraction of cells localizing to the overlapping microtubules is represented as a percentage. Scalebar, 10 μm.

E Live-cell imaging of the midbody in HeLa cells transfected with wild-type, phosphomimetic and non-phosphorylatable mutants of GFP-GST-CENP-E$_{2605–2701}$ and stained for tubulin. Scalebar, 10 μm. Data represented from three independent experiments.

F Linescans showing the mean fluorescence intensity and standard error (SEM) for the GFP-CENP-E$_{2605–2701}$ wild-type constructs and SA and SD mutants and tubulin across the cell midbody. *n* (cells) = 27, 21 and 20 respectively. Two to three biological replicates were collected.

G Bar graph showing mean and standard error for GFP fluorescence intensity at peak fluorescence for GFP-GST-CENP-E$_{2605–2701}$ and mutants at 9.7 μm, quantified in (F). Asterisks indicate ordinary one-way ANOVA test significance value. ****$P < 0.0001$.

Source data are available online for this figure.

capture and alignment, and then to associate with PRC1 later in mitosis.

Structural features of CENP-E-PRC1 interactions

We used AlphaFold2 to predict how CENP-E might interact with PRC1, using CENP-E$_{2605–2701}$ and PRC1$_{1–168}$ dimers as inputs for our analysis (Jumper *et al*, 2021; Mirdita *et al*, 2022). AlphaFold2 predicted that CENP-E could interact with the rod and dimerization interface of PRC1 with high confidence, and identified $_{2660}$YFD$_{2661}$ in CENP-E as important for that interaction (Fig 6A). PRC1 is dimeric, with the dimerization domains and the rod fold organized around a twofold symmetry, antiparallel to each other (Subramanian *et al*, 2013). The ΦΦ-binding sites on PRC1 are in close proximity to each other. This could explain why two ΦΦ motifs from the same peptide can bind to PRC1 to increase motor affinity, such as CENP-E or KIF4A, for PRC1. Based on AlphaFold2 predicted structures of the CENP-E$_{2605–2701}$:PRC1$_{1–168}$ complex, we could identify several amino acids that might be involved in coordinating the ΦΦ motif: I25, W26, M54, E57 and E58. In order to test these structural predictions, we generated a PRC1$_{1–168}$ in which M54, E57 and E58 were all mutated to A, named PRC1$_{1–168}$ *MEE* (Table 1). PRC1$_{1–168}$ *MEE* was purified and soluble, behaving similarly to PRC1$_{1–168}$ in size-exclusion chromatography (Fig 6B and C). To check the oligomerization status of PRC1$_{1–168}$ *MEE*, we performed size-exclusion chromatography coupled with multi-angle light scattering (SEC-MALS). Both have a measured molecular weight of 52 ± 2.5 kDa, close to the predicted molecular weight for PRC1$_{1–168}$ dimer 47.1 kDa (Fig 6B and C). These measurements indicate both PRC1$_{1–168}$ and PRC1$_{1–168}$ *MEE* are dimeric, around a twofold symmetry axis that supports the antiparallel crosslinking of microtubules and that the inserted mutations do not disrupt the PRC1 dimer interface. We then test that full-length PRC1 *MEE* could also crosslink microtubules similarly to full-length PRC1 in solution (Fig 6D). Next, we tested whether PRC1$_{1–168}$ *MEE* interacted with CENP-E by size-exclusion chromatography. PRC1$_{1–168}$ *MEE* and MBP-CENP-E$_{2605–2701}$ did not co-elute, indicating PRC1$_{1–168}$ *MEE* did not bind to MBP-CENP-E$_{2605–2701}$ (Fig 6E).

Overall, these data indicate that kinesin motors bind the dimerization rod domain of PRC1 using their bipartite ΦΦ motifs.

PRC1-motor interactions are essential for cytokinesis

PRC1 has dual-molecular functions: it crosslinks microtubules, and it associates with kinesin motors, such as CENP-E and Kif4A. These functions allow assembly of the central spindle and ensure the final steps of mitosis. In order to distinguish the contribution(s) of PRC1 to central spindle formation, which could occur either by crosslinking microtubules or by recruiting kinesin motors, we engineered cell lines expressing GFP-PRC1-WT or GFP-PRC1-*MEE* (which does not bind the ΦΦ motif in kinesin motors; Fig 7A). Both cell lines were stable, indicating that these constructs did not have a dominant effect. Both cell lines also expressed constitutively a guide RNA that targets endogenous PRC1, with Cas9 expressed using an inducible promoter, so that we could induce PRC1 knockout by addition of doxycycline (McKinley & Cheeseman, 2017), as an alternative to siRNA knockdown (Fig 7A).

We observed that GFP-PRC1-WT localizes to overlapping microtubules in the metaphase spindle, the central spindle and the midbody, as previously reported (Fig 7A and B; Subramanian *et al*, 2013; Kajtez *et al*, 2016; Pamula *et al*, 2019). We found that GFP-PRC1-WT localization was slightly reduced when endogenous PRC1 was knocked down, most likely due to the N-terminal GFP-tagging of PRC1, which places the tag close to the dimerization interface and the motor-binding interface. However, we observed that endogenous CENP-E was present at overlapping structures

Figure 6. Molecular basis for the PRC1-CENP-E interaction.

A AlphaFold2 prediction of the CENP-E/PRC1 interaction identifies the PRC1 residues important in CENP-E binding.

B, C Elution profile from a size-exclusion chromatography (SEC, black line, left *y*-axis) run with subsequent multi-angle light scattering (MALS, right *y*-axis) analysis for PRC1$_{1-168}$ and PRC1$_{1-168}$ *MEE*. Outcome of the MALS analysis for the peak is presented in pink (molecular weight, right *y*-axis).

D Fire purple–yellow intensity LUT used to show tubulin intensity and microtubule overlaps. Microtubules are shown alone or incubated with 2.5 nM full-length PRC1 WT or PRC1 *MEE*. Scalebar, 10 μm. Experiment was replicated two times.

E SEC elution profile of PRC1$_{1–168}$ *MEE* (pink), MBP-CENP-E$_{2605–2701}$ (green) and MBP-CENP-E$_{2605–2701}$ /PRC1$_{1–168}$ *MEE* (orange) (black line, left *y*-axis).

Source data are available online for this figure.

 © 2023 The Authors

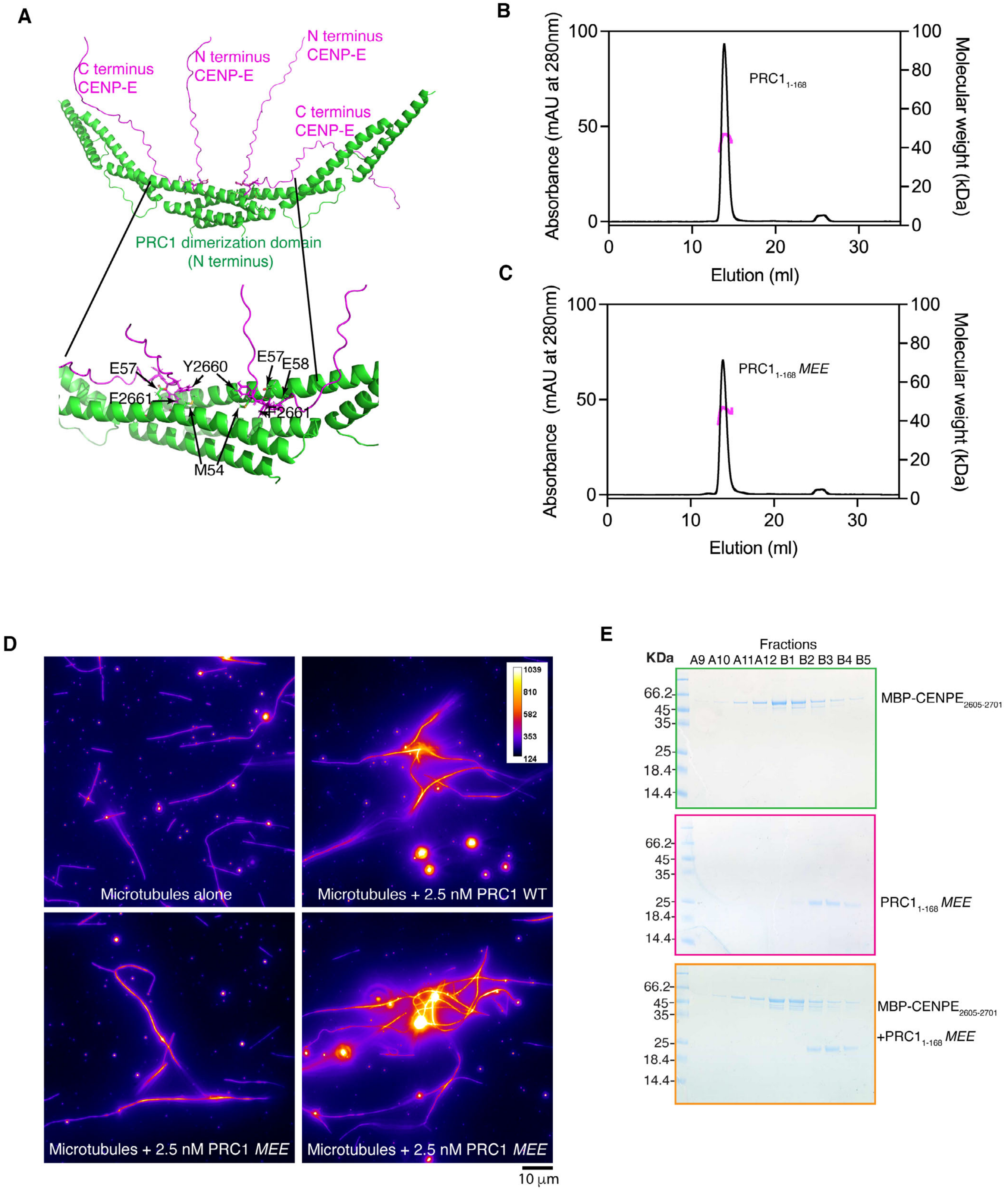

Figure 6.

bound by GFP-PRC1-WT, indicating that GFP-PRC1-WT was able to interact with the ΦΦ motif of CENP-E and recruit CENP-E (Fig 7A and C). When endogenous PRC1 was knocked down, cells expressing GFP-PRC1-*MEE* still progressed through mitosis and chromosome segregated in anaphase, meaning that checkpoint silencing must have taken place. In anaphase, the amount of GFP-

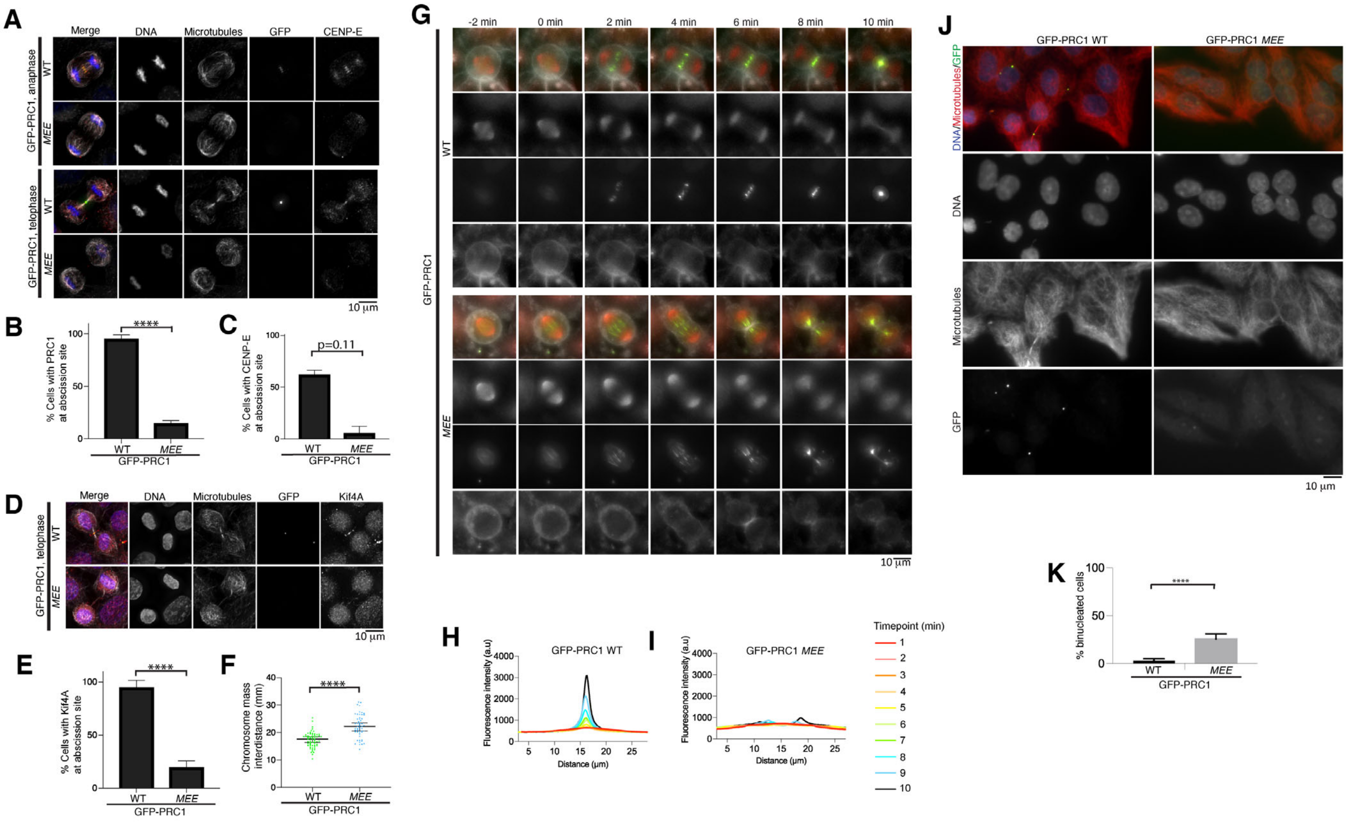

Figure 7. The interaction of PRC1 with motors is critical for the integrity of the central spindle.

A Representative immunofluorescence images of HeLa cells in late anaphase and telophase, expressing GFP-PRC1-WT or -*MEE* and depleted for endogenous PRC1 using a PRC1 siRNA and a PRC1 sgRNA after doxycycline-induced Cas 9 expression. Microtubules, GFP and CENP-E are in white, green and red respectively. DNA is in blue.

B Graph showing the percentage of cells with a PRC1 localized at the site of abscission for cells treated in (A). Mean and standard deviation are presented, $n = 156$ and 141 for cells expressing PRC1-WT and -MEE respectively. Data are from four biological replicates and one technical replicate. Asterisks indicate a *T*-test significance value. ****$P < 0.0001$.

C Graph showing the percentage of cells with CENP-E localized at the abscission site for cells treated in (A), mean and standard deviation are presented, $n = 58$ and 66 for cells expressing PRC1-WT and -MEE respectively. Data are from two biological replicates. *P*-value ($P = 0.11$) calculated for an unpaired *T*-test.

D Representative immunofluorescence images of HeLa cells in telophase, expressing GFP-PRC1-WT or -*MEE* and depleted for endogenous PRC1 using a PRC1 siRNA and a PRC1 sgRNA after doxycycline-induced Cas 9 expression. Microtubules, GFP and Kif4A are in white, green and red respectively. DNA is in blue.

E Graph showing the percentage of cells with Kif4A localized at the abscission site for cells treated in (D). Mean and standard deviation are presented, $n = 90$ and 78 for cells expressing PRC1-WT and -MEE respectively. Asterisks indicate a *T*-test significance value for an unpaired *T*-test. ****$P < 0.0001$. Data from two biological replicates and one technical replicate.

F Interchromosome distance in telophase in cells expressing GFP-PRC1-WT and GFP-PRC1-*MEE* in the absence of endogenous PRC1 ($n = 60$ and $n = 53$, respectively). Median and 95% confidence intervals are presented. Data from two biological replicates. Asterisks indicate a *T*-test significance value for an unpaired *T*-test. ****$P < 0.0001$.

G Representative live-cell images of HeLa cells expressing GFP-PRC1-WT or -*MEE* and depleted for endogenous PRC1 using a PRC1 siRNA and a PRC1 sgRNA after doxycycline-induced Cas 9 expression. Microtubules (SiR-Tubulin) and DNA (SPY650) are shown in red. The cell membrane (CellMask) is highlighted in white and GFP-PRC1 is in green. Scalebar, 10 μm.

H, I Graph showing the change in fluorescence intensity for DNA and microtubules along the longitudinal axis of the spindle from metaphase (red) to anaphase and cytokinesis (dark blue) for cells expressing GFP-PRC1-WT (H, $n = 10$) or -*MEE* (I, $n = 10$) in the absence of endogenous PRC1.

J Representative immunofluorescence images of cells expressing GFP-PRC1-WT or -*MEE* after 72 h siRNA depletion and doxycycline-induced knockout of endogenous PRC1. DNA, microtubules and GFP-PRC1/GFP-PRC1-*MEE* are in blue, red and green respectively. Scalebar, 10 μm. Quantification was from two biological replicates but experiment was also repeated using in (G).

K Quantification of the number of cells mononucleated and binucleated from experiment in (J). $n = 633$ and 724 for cells expressing GFP-PRC1 WT and GFP-PRC1-*MEE* respectively. Data are from two independent experiments. Asterisks indicate a *T*-test significance value. ****$P < 0.0001$.

Source data are available online for this figure.

PRC1-*MEE* was reduced on the central part of the spindle compared with that observed for GFP-PRC1-WT (Fig 7A and B). The central spindle, which is usually marked by a high density of antiparallel microtubules, was absent or severely disrupted in cells expressing GFP-PRC1-*MEE* and lacking endogenous PRC1. Very few microtubules were observed between the two segregating half-spindles. CENP-E was not present on the central spindle in these cells (Fig 7A and C). We also observed Kif4A was recruited to GFP-PRC1-WT, but

 © 2023 The Authors

was largely absent from the abscission site in the presence of GFP-PRC1-*MEE* (Fig 7D and E). Under these conditions, we observed hypersegregation of chromosomes in the presence of GFP-PRC1-*MEE*, with the distance between the chromosome mass significantly greater than in cells expressing GFP-PRC1-WT (Fig 7A and F). This phenotype is similar to that seen in cells depleted for PRC1 (Pamula *et al*, 2019).

To better understand how PRC1-motor interactions affect chromosome segregation and cell division, we carried out time-lapse imaging on cells expressing GFP-PRC1-WT or GFP-PRC1-*MEE* in the absence of endogenous PRC1, using dyes to demarcate cell membrane, DNA and microtubules. We observed that the speed of the chromosome and spindle pole mass to the daughter cells, marked by DNA and tubulin dyes, respectively, was similar in cells expressing GFP-PRC1-*MEE* compared with those expressing GFP-PRC1-WT (Fig 7G). We measured the accumulation of GFP-PRC1-WT on the central spindle over time (Fig 7H). GFP-PRC1-*MEE* was weakly recruited to overlapping microtubules (Fig 7I), unlike GFP-PRC1-WT, which accumulated in the central spindle throughout anaphase and telophase. However, there were less antiparallel microtubules and the fibres appear thicker, indicating an abnormal regulation of microtubule bundling in the presence of GFP-PRC1-*MEE* (Fig 7G). The staining of GFP-PRC1-*MEE* was more diffuse than for GFP-PRC1-WT, and the distribution of GFP-PRC1-*MEE* was not constrained to the central spindle (Fig 7G and I). Additionally, GFP-PRC1-marked bundles seemed to move away from each other and make thicker bundles, which were easier to distinguish during spindle elongation in anaphase. It is also possible that the PRC1-*MEE*-marked bundles were severed or broken at the site of furrow ingression (Fig 7G).

PRC1 is essential for cytokinesis (Jiang *et al*, 1998; Mollinari *et al*, 2005). Next, we analysed whether the motor recruitment properties of PRC1 contribute to cytokinesis. We depleted endogenous PRC1 in cells expressing GFP-PRC1-WT or GFP-PRC1-*MEE* and imaged them after 72 h. In cells expressing GFP-PRC1-*MEE*, there was a significant increase in binucleated cells, indicating they had failed cytokinesis and were tetraploid (Fig 7J and K). Overall, these results indicate the motor recruitment properties of PRC1 are critical to ensure the completion of cytokinesis and support successful cell division. Future work will be needed to determine the contributions of individual PRC1-motor interactions to anaphase and cytokinesis.

Discussion

In this manuscript, we report the mechanistic basis of PRC1-microtubule motor interactions, and recapitulate microtubule sliding by a CENP-E:PRC1 complex. We reveal the functional contribution of PRC1-interacting microtubule motors to spindle elongation in anaphase and the completion of chromosome segregation. We identify the temporal and spatial features of these regulated interactions, and show that these features are required for correct timing of cytokinesis.

In early mitosis, CENP-E is present at unattached kinetochores, and moves laterally attached kinetochores along microtubules. The kinetochore-bound CENP-E slides microtubules by pushing microtubules relative to kinetochores and promoting spindle flux (Steblyanko *et al*, 2020). CENP-E has also been proposed to slide spindle microtubules past each other (Risteski *et al*, 2021). Some motors, such as the Kinesin-14 dimeric motor HSET, use both its motor and non-motor microtubule-binding domains to slide microtubules past each other (Cai *et al*, 2009; Braun *et al*, 2017). We demonstrate that CENP-E, unlike Kinesin-14 HSET, does not slide antiparallel microtubules on its own *in vitro*. Instead, CENP-E promotes microtubule sliding in the presence of PRC1 *in vitro*. This suggests that it may function in a similar manner to Kif4A in anaphase (Bieling *et al*, 2010; Subramanian *et al*, 2013; Figs 2 and 4).

Previous work on the CENP-E C-terminal tail included part of the kinetochore targeting domain (Gudimchuk *et al*, 2013). *In vitro* and *in vivo*, we did not observe any binding of the CENP-E C-terminal domain to microtubules in the absence of CENP-E interaction with PRC1 (Fig 4A). The unstructured C terminus of CENP-E is phosphorylated in metaphase (Dephoure *et al*, 2008). Therefore, its affinity for microtubules in the context of the full-length motor is likely to be weak and non-specific. The data we present in this manuscript rule out microtubule-sliding activity of CENP-E via its C-terminal tail.

The timing of the PRC1–motor interaction is important because Kif4A and CENP-E are involved in chromosome organization and alignment in early mitosis (reviewed in Samejima *et al*, 2012; Craske *et al*, 2022). PRC1 is phosphorylated by CDK1/cyclin B on T470 and T481, in the region important for microtubule binding, at the junction between the unstructured microtubule-binding tail and the spectrin domain (Jiang *et al*, 1998). The microtubule bundling activity of PRC1 has been proposed to be downregulated by CDK1 and Plk1 phosphorylation (Mollinari *et al*, 2002; Hu *et al*, 2012). We show here that mitotic phosphorylation of CENP-E also reduces the strength of the PRC1–CENP-E interaction. It ensures that CENP-E associates with kinetochores to promote their alignment in early mitosis and does not associate with PRC1 (Jagric *et al*, 2021). At the metaphase to anaphase, cyclin B degradation leads to a decrease in mitotic kinase activity. As chromosomes segregate, we conclude CENP-E is dephosphorylated, which increases its affinity for PRC1 and facilitates its recruitment to the central spindle. Interestingly, the two ΦΦ motifs in the Kif4A motor are also flanked by threonines and serines (Nousiainen *et al*, 2006; Huttlin *et al*, 2010; Olsen *et al*, 2010; Kettenbach *et al*, 2011; Fig 3A). Thus, mitotic phosphorylation of the C terminus of Kif4A may regulate the Kif4A:PRC1 interaction temporally and spatially.

The motor recruiting function of PRC1, via the ΦΦ binding site, is crucial to the correct completion of cytokinesis and chromosome segregation (Fig 7). When this interaction is abrogated, the chromosomes separate further. Ultimately, cells fail cytokinesis and become multinucleated (Fig 7). It is possible that the other kinesins such as Kif14, MKLP2 and MKLP1 use an ΦΦ motif for PRC1 recruitment to the central spindle. However, we could not identify a PRC1 binding site from their primary sequence with confidence. MKLP1 forms a heterotetramer with the RhoGAP Cyk4/MgcRacGAP, which has a hydrophobic motif important for PRC1 binding *Caenorhabditis elegans* and is highly conserved (Lee *et al*, 2015). If the MKLP2-PRC1 interaction is disrupted, MKLP2-dependent transport of the CPC (chromosomal passenger complex) to the central spindle would be compromised and prevent cytokinesis completion (Gruneberg *et al*, 2004; Adriaans *et al*, 2020; Serena *et al*, 2020).

© 2023 The Authors

During anaphase, we observe that the central spindle starts to assemble, but the antiparallel microtubule bundles, marked by GFP-PRC1-*MEE*, are reduced and no longer concentrated at the central spindle. GFP-PRC1-*MEE* still has a strong preference for antiparallel microtubule fibres and crosslinks microtubules within the spindle overlap. However, GFP-PRC1-*MEE* is not concentrated at the plus end of overlaps, highlighting that motors are essential for concentrating PRC1 and marking the central spindle and midbody (Subramanian *et al*, 2013; Wijeratne & Subramanian, 2018; Hannabuss *et al*, 2019). The PRC1-marked bundles also lose their coherent behaviour within the spindle (Fig 7). Recent work proposes that microtubule bundles in the central spindle are connected to each other (Carlini *et al*, 2022). Our data reveal that PRC1-interacting proteins that bind to the dimerization domain contribute to interbundle stability by crosslinking different sets of microtubule bundles. This may reinforce their stiffness.

The central spindle is proposed to act as a break counteracting forces that drive spindle elongation (Janson *et al*, 2007; Lee *et al*, 2015). The forces generated by single PRC1 molecules on microtubules are low, in the 0.1 pN range (Forth *et al*, 2014). At higher density, PRC1-crosslinked microtubules can produce significant resistance during microtubule sliding that scale with velocity of microtubule sliding, in the range 5–20 pN for sliding velocities of 25–200 nm/s (Gaska *et al*, 2020). Our results suggest that the brake forces produced by the microtubule-crosslinking activity of PRC1 do not fully oppose forces that drive chromosome separation because chromosomes hypersegregate in the presence of PRC1 which does not bind motors (Fig 7). This has also been reported for cells lacking PRC1 in which two half spindles became disconnected and were pulled apart (Pamula *et al*, 2019; Vukusic *et al*, 2021). Because controlled microtubule sliding does not occur when PRC1 is not bound to the kinesin motors, the outwards spindle and cortical-generated forces are likely to dominate the system. We propose that PRC1 acts both as a break and signalling adaptor. Kinesin motors associate with PRC1 via the conserved ΦΦ motifs and either generate breaking forces on the spindle or recruit other signalling molecules to regulate cytokinesis (Neef *et al*, 2007). Kif4A, CENP-E, MKLP1 and MKLP2 all interact with PRC1 across species. Future work will address how these PRC1-interacting motors work collectively to complete cell division.

Failed cytokinesis is a hallmark of cancer cells, leading to chromosome instability. Fast-growing polyploid cancer cells are particularly vulnerable to cytokinesis failure (McKenzie & D'Avino, 2016). Our work may open up opportunities to interfere with cytokinesis completion and induce cytokinesis failure in cancer cells as a therapeutic cancer target to increase chromosome instability and cell death (Lens & Medema, 2019).

Materials and Methods

Cloning

To assay the localization in cell culture of CENP-E subdomains, various constructs were generated from CENP-E transcript variant 1 (NM_001813.2) and cloned into pBABE-blasticidin containing an N-terminal GFP tag and using restriction enzymes (Cheeseman & Desai, 2005). PRC1 was also cloned into pBABE-blasticidin containing an N-terminal GFP tag. MBP-CENP-E was cloned into pMal-C2X (NEB). Bacterially expressed constructs of GST-CENP-E were cloned in pET-3aTr (Tan, 2001). Mutagenesis was performed according to Quickchange mutagenesis protocols (Agilent). Mutants for CENP-$E_{2605–2701}$ and $PRC1_{1–168}$ were synthesized using G-Blocks (IDT).

Protein expression, purification and assays

All constructs for bacterial expression were transformed in *Escherichia coli* BL21-CodonPlus (DE3)-RIL. Cultures were induced with 0.5 mM IPTG when OD_{600} = 0.6 for 4 h at 25°C or overnight at 18°C for 18–20 h. Cells expressing his_6-proteins ($PRC1_{1–168}$, PRC1 full-length) were re-suspended in lysis buffer (50 mM HEPES pH 7.5, 500 mM NaCl, 40 mM imidazole and 5 mM β-mercaptoethanol) supplemented with 1 mM PMSF and cOmplete EDTA-free protease inhibitor cocktail (Roche) and lysed by sonication. The lysate was cleared by centrifugation (50 min, 58,440 *g*) in a JA 25.50 rotor (Beckman Coulter), filtered and loaded onto a HisTrap HP column (Cytiva). His_6-tagged proteins (from Sf9 cells and bacteria) were eluted in elution buffer (lysis buffer containing 250 mM imidazole). Constructs containing a 3C protease cleavage site were incubated overnight in dialysis buffer (25 mM HEPES pH 7.5, 300 mM NaCl, 10 mM imidazole, 1 mM EDTA and 5 mM β-mercaptoethanol) with 3C protease and then loaded onto a HisTrap HP column (Cytiva). MBP-CENP-$E_{2605–2701}$ was purified using the same lysis buffer without imidazole and an MBP-Trap HP column (Cytiva). For ITC, the MPB tag was cleaved overnight using Factor Xa (NEB) in dialysis buffer and loaded again on an MBP-Trap HP column. GST proteins were purified as previously described (Legal *et al*, 2020). Recombinant proteins were then concentrated and loaded on a Superdex 200 Increase 10/300 GL (Cytiva) pre-equilibrated in size-exclusion chromatography buffer: 20 mM HEPES pH 7.5, 300 mM NaCl, 1 mM EDTA and 1 mM DTT. Labelling of 647His_6-GST-CENP-E was performed according to the manufacturer's instructions with the AlexaFluor647 (A20173A, Invitrogen). Degree of labelling was estimated as 1:3.

Full-length CENP-E was expressed in Sf9 insect cells and purified as previously published (Craske *et al*, 2022). Freshly purified CENP-E was used for *in vitro* sliding assays in Fig 4 due to deterioration in activity after freezing. Porcine brain tubulin was purified as described (Castoldi & Popov, 2003) and stored in liquid nitrogen long term.

Isothermal titration calorimetry

ITC experiments were carried out to determine the affinity and stoichiometry of PRC1:CENP-E constructs and Kif4A, known to bind PRC1. CENP-E peptides were synthesized by Lifetein, LLC. $PRC1_{1–168}$, CENP-E constructs and GST-$Kif4A_{1133–1165}$ were extensively dialysed into ITC buffer (20 mM HEPES pH 7.5, 150 mM NaCl, 0.005% Tween-20 and 0.5 mM TCEP); prior to the experiment to minimize heats of dilution upon titration. Peptides were directly diluted into ITC buffer. Protein concentrations were determined by absorption at 280 nm; extinction coefficients ε were as follows: $PRC1_{1–168}$: 8,480 M^{-1} cm^{-1}, CENP-$E_{2605–2701}$: 6,990 M^{-1} cm^{-1} and GST-CENP-$E_{2605–2701}$: 49,850 M^{-1} cm^{-1}. Peptide concentrations were determined by absorption at 214 nm; extinction coefficients ε

© 2023 The Authors

were 22,904 M^{-1} cm^{-1} for peptide 1 and 22,983 M^{-1} cm^{-1} for peptide 2. For protein–protein ITC experiments, 1,140, 224 and 224 μM $PRC1_{1–168}$ were titrated into 56.1, 18.6 and 20.7 μM CENP-$E_{2605–2701}$, GST-CENP-$E_{2605–2701}$ and GST-CENP-$E_{2605–2701\ 2SD}$, respectively, at 25°C in 16 aliquots: 1 of 0.5 μl followed by 15 × 2.5 μl. The concentration was calculated for the monomeric CENP-E constructs. For protein–peptide ITC experiments, 557 μM of $PRC1_{1–168}$ (calculated for monomeric PRC1) was titrated into 15 μM peptide 1 or peptide 2 at 25°C in 16 aliquots: 1 of 0.5 μl followed by 15 × 2.5 μl. The reference power was set to 3 μcal/s. The enthalpy of binding was analysed with correction for heat of dilution using the software package provided by the instrument manufacturer (Auto-iTC200 microcalorimeter; Malvern Instruments). Data were fit to a simple binding model with one set of sites.

SEC-MALS

Size-exclusion chromatography coupled to UV, static light scattering and refractive index detection (Viscotec SEC-MALS 20 and Viscotek RI Detector VE3580; Malvern Instruments) was used to determine the absolute molecular mass of $PRC1_{1–168}$ and $PRC1_{1–168}$ *MEE* in solution. Injections of 100 μl of 3.6 and 2.0 mg/ml of $PRC1_{1–168}$ and $PRC1_{1–168}$ *MEE* (152 and 84 μM), respectively, were run on a calibrated Superdex-200 Increase 10/300 GL (Cytiva) size-exclusion column pre-equilibrated in 20 mM HEPES, pH 7.4; 300 mM NaCl; and 1 mM EDTA at 22°C with a flow rate of 1.0 ml/min. Light scattering, refractive index (RI) and A_{280nm} were analysed by a homopolymer model (OmniSEC software, v5.02; Malvern Instruments) using the following parameters: $\partial A_{280nm}/\partial c = 0.43$ AU ml/mg, $\partial n/\partial c = 0.185$ ml/g and buffer RI value of 1.338.

Cell culture, immunofluorescence and microscopy

Stable clonal HeLa cell lines expressing GFP-PRC1 WT and GFP-PRC1 *MEE* (siRNA resistant and Cas9 resistant) were generated as described previously using a retroviral system (Cheeseman & Desai, 2005). The HeLa cells also expressed constitutively a guide RNAi targeting PRC1 and Cas9 under an inducible promoter (McKinley & Cheeseman, 2017). To knockout PRC1 inducibly, cells were treated with 1 μg/ml doxycycline for 48–72 h. We observed the knockout was partial and hence we also used siRNA to deplete PRC1. To deplete PRC1, we treated the cells for 48–72 h with an siRNA which targets the 3′UTR of PRC1 (A-019491-15-0020, Horizon Discovery) previously characterized (Jagric *et al*, 2021). To quantify binucleation and cytokinesis failure, we treated the cells with doxycycline and PRC1 siRNA for 72 h.

HeLa cells (93021013, Sigma Aldrich) were used and maintained in DMEM (Gibco) supplemented with 10% Tet-free FBS (A4736401, ThermoFisher), 5% penicillin/streptomycin (Gibco) and 2.5 mM L-glutamine at 37°C in a humidified atmosphere with 5% CO_2. Cells are monthly checked for mycoplasma contamination (MycoAlert detection kit, Lonza). Transient transfections were conducted using Effectene reagent (Qiagen) or lipofectamine 3000 (Invitrogen) according to the manufacturer's guidelines. Cells were washed in PBS and fixed in ice-cold methanol or alternatively 3.8% formaldehyde in PHEM buffer (60 mM Pipes, 25 mM HEPES, 10 mM EGTA and 2 mM $MgSO_4$, pH 7.0) for 10 min. For immunofluorescence, cells were incubated 5 min in pre-extraction buffer containing 22.6 nM $_{647}$GST-CENP-$E_{2639–2671}$ and fixed with 10 min cold methanol followed by 1 min acetone treatment. Immunofluorescence in human cells was conducted as previously described using antibodies against tubulin (1:1,000 anti-beta tubulin, mouse, T7816, Sigma OR 1:2,000/1:1,000 anti-alpha tubulin, rabbit, ab18251, Abcam) and PRC1 (sc-376983, Santa Cruz, 1:200 mouse; McHugh *et al*, 2018). Anti-CENP-E (Abcam, Ab5093; 1:1,000 or 1:200), mouse anti-Kif4 (sc-365144, Santa Cruz; 1:100), guinea pig anti-CENP-C (pAb; MBL PD030; 1:2,000), secondary anti-rabbit Cy3 (1:400, Invitrogen) and anti-mouse Cy2 (1:800; Invitrogen) were used for immunofluorescence. Hoechst 33342 (Thermo Fisher Scientific; H3570) was used to stain DNA. Images were obtained from a widefield Eclipse Ti2 (Nikon) microscope equipped with a Prime 95B Scientific CMOS camera (Photometrics) using a 100× objective (CFI Plan Apochromat Lambda, 1.49 N.A). A total of 10–20 z-sections were acquired at 0.2–0.5 μm and presented as maximum-intensity projections. For live-cell imaging, cells were transferred into a 35 mm glass-bottom viewing chamber (MatTek). Prior to imaging, cells were incubated for 5 min with CellMask orange (1:40,000, Thermo Fisher Scientific) and 5 min with SPY650 (1:1,000, Spirochrome). Cells were washed multiple times in L15 Leibowitz media (Gibco) supplemented with 10% FBS and 2.5 mM L-glutamine, prior to imaging on the widefield Eclipse Ti2 (Nikon) microscope equipped with a Prime 95B Scientific CMOS camera (Photometrics), using a 60× oil objective (CFI Plan Apochromat Lambda, Nikon, 1.3 N.A) and a heated chamber with CO_2. Data were acquired for the three channels at 1 min interval with an optical spacing of 1.25 μm.

Sample preparation for TIRF microscopy and TIRF microscopy imaging

For PRC1 and CENP-E, microtubule-binding assays, 0.2 mg/ml GMPCPP (Jena Biosciences) microtubule seeds containing 7% rhodamine-tubulin (Cytoskeleton Inc., TL590M-B) were polymerized in BRB80 (80 mM PIPES pH 6.9, 1 mM EGTA and 1 mM $MgCl_2$) for 1 h at 37°C, followed by centrifugation at 17,000 *g* for 10 min and then resuspended in BRB80. Anti-tubulin antibodies (Sigma, T7816) at a 1:10 dilution in BRB80 were first introduced to the chamber. Next, 40 μl of 1% Pluronic F-127 (Sigma Aldrich) in BRB80 was washed through the chamber and incubated for 5 min. Chambers were then washed with 40 μl of BRB80, then 40 μl 1 mg/ml casein (Sigma Aldrich) before adding the final mixture of GMP-CPP microtubules and PRC1 and/or CENP-E in final assay mix at indicated concentrations (80 mM PIPES pH 6.9, 5 mM $MgCl_2$, 1 mM DTT and an oxygen scavenger mix: 0.2 mg/ml glucose oxidase, 0.035 mg/ml catalase, 4.5 mg/ml glucose and 0.1% β-mercaptoethanol).

Sliding assays were carried out in flow chambers consisting of functionalized glass coverslips coated with PEG-biotin. Firstly, chambers were washed with BRB80. Next, 40 μl of 50 μg/ml Neutravidin was washed through the chamber and left to incubate for 5 min. GMPCPP polymerized biotinylated tubulin (HiLyte647 labelled) was washed into the chamber and left for 5 min. Next, 50 μl of purified full-length PRC1 at 2.5 nM was added to coat the microtubules. This was left for 10 min. Chambers were then washed with BRB80, followed by flowing through with a final assay mix containing GMPCPP non-biotinylated microtubules, 2 mM ATP, 0.5 mg/ml casein, oxygen scavenger and CENP-E motor at indicated

© 2023 The Authors

concentration (or buffer as a control). Microscopy was carried out immediately following this step. For microtubule sliding assays, images of free rhodamine microtubules and immobilized HiLyte 647-biotinylated microtubules using the red and far-red channels, respectively, were taken every 2 s for a total of 20 min. Imaging was performed on a Zeiss Axio Observer Z1 TIRF microscope using a Zeiss 100 × NA 1.46 objective and either a Photometrics Evolve Delta electron-multiplying charge-coupled device camera or a Photometrics Prime 95B sCMOS camera controlled by Zeiss Zen Blue software.

Image analysis

Quantification was done in Omero (OME, or ImageJ; National Institutes of Health; Allan *et al*, 2012; Schneider *et al*, 2012). Linescans for measurement of intensity across the central spindle were generated for cells at a stage of cell division defined by taking the width of the spindle of around 10–12 pixels, visualized by tubulin staining with SiR dye (1:40,000 at a final concentration of 25 nM, for 90–120 min, Spirochrome). For the measurement of the chromosome separation in telophase, the maximum distance between chromosome masses along the spindle axis was measured after maximum-intensity projection of images. Cell stages were assessed by DNA morphology; telophase was distinguished from cytokinesis by the state of chromosome condensation and the shape of the cell; analysis was done in late anaphase and telophase cells, excluding cytokinesis. For quantification of midbody integrity, cells in cytokinesis were identified morphologically with a bundle of microtubules between neighbouring cells, marked by GFP-PRC1. For cells expressing PRC1-*MEE* in the absence of endogenous CENP-E, the bundle was generally partially or fully missing, or distorted, and GFP-PRC1 was absent or on the remnant midbody, but chromosomes and microtubules could be seen between the two cells. Midbody and abscission site were defined as a distinct PRC1 signal between the future daughter cells.

Statistics and reproducibility

Statistical analyses were performed using GraphPad Prism 9.0. Data normality was checked. No statistical method was used to predetermine sample size. No data were excluded and no blinding was done.

AlphaFold analysis

The PRC1:CENP-E dimeric complex structure was predicted using AlphaFold2 in the multimer version.

Data availability

This study includes no data deposited in external repositories.

Expanded View for this article is available online.

Acknowledgements
His-GFP-PRC1 and His-PRC1 were kind gifts from Tarun Kapoor (Rockfeller University). We are grateful to Iain Cheeseman (Whitehead institute for Biomedical Research, Cambridge, Massachusetts, USA) for sharing the human HeLa-inducible CRISPR/Cas9/PRC1-G2.2 knock-out (KO) cell line. We thank Owen Davies for help with AlphaFold2 and Liz Blackburn for help with ITC. We thank Martin Wear for help with SEC-MALS, and COIL for imaging support. JPIW received a JEDI award from the Life Science Editors Foundation, which provided editorial feedback on an early draft of the manuscript. JPIW is supported by a Wellcome Senior Research Fellowship (207430). BC was supported by the Biotechnology and Biological Sciences Research Council (BBSRC) (grant number BB/M010996/1). This project was also supported by a COVID-reboot grant from the Royal Society of Edinburgh. The Wellcome Centre for Cell Biology is supported by core funding from the Wellcome Trust (203149). A Multi-User Equipment grant for the Edinburgh Protein Production Facility is also funded by the Wellcome Trust (101527).

Author contributions
Agata Gluszek-Kustusz: Formal analysis; investigation; writing – review and editing. **Benjamin Craske:** Formal analysis; investigation. **Thibault Legal:** Formal analysis; investigation; writing – review and editing. **Toni McHugh:** Formal analysis; investigation; writing – review and editing. **Julie PI Welburn:** Conceptualization; supervision; funding acquisition; investigation; methodology; writing – original draft; project administration; writing – review and editing.

Disclosure and competing interests statement
The authors declare that they have no conflict of interest.

References

Adriaans IE, Hooikaas PJ, Aher A, Vromans MJM, van Es RM, Grigoriev I, Akhmanova A, Lens SMA (2020) MKLP2 is a motile kinesin that transports the chromosomal passenger complex during anaphase. *Curr Biol* 30: 2628 – 2637 e2629

Allan C, Burel JM, Moore J, Blackburn C, Linkert M, Loynton S, Macdonald D, Moore WJ, Neves C, Patterson A *et al* (2012) OMERO: flexible, model-driven data management for experimental biology. *Nat Methods* 9: 245 – 253

Bieling P, Telley IA, Surrey T (2010) A minimal midzone protein module controls formation and length of antiparallel microtubule overlaps. *Cell* 142: 420 – 432

Braun M, Lansky Z, Szuba A, Schwarz FW, Mitra A, Gao M, Ludecke A, Ten Wolde PR, Diez S (2017) Changes in microtubule overlap length regulate kinesin-14-driven microtubule sliding. *Nat Chem Biol* 13: 1245 – 1252

Cai S, Weaver LN, Ems-McClung SC, Walczak CE (2009) Kinesin-14 family proteins HSET/XCTK2 control spindle length by cross-linking and sliding microtubules. *Mol Biol Cell* 20: 1348 – 1359

Carlini L, Renda F, Pamula MC, Khodjakov A, Kapoor TM (2022) Coupling of microtubule bundles isolates them from local disruptions to set the structural stability of the anaphase spindle. *Proc Natl Acad Sci USA* 119: e2204068119

Castoldi M, Popov AV (2003) Purification of brain tubulin through two cycles of polymerization-depolymerization in a high-molarity buffer. *Protein Expr Purif* 32: 83 – 88

Chan GK, Schaar BT, Yen TJ (1998) Characterization of the kinetochore binding domain of CENP-E reveals interactions with the kinetochore proteins CENP-F and hBUBR1. *J Cell Biol* 143: 49 – 63

Chan GK, Jablonski SA, Sudakin V, Hittle JC, Yen TJ (1999) Human BUBR1 is a mitotic checkpoint kinase that monitors CENP-E functions at kinetochores and binds the cyclosome/APC. *J Cell Biol* 146: 941 – 954

 © 2023 The Authors

Cheeseman IM, Desai A (2005) A combined approach for the localization and tandem affinity purification of protein complexes from metazoans. *Sci STKE* 2005: pl1

Ciossani G, Overlack K, Petrovic A, Huis In 't Veld PJ, Koerner C, Wohlgemuth S, Maffini S, Musacchio A (2018) The kinetochore proteins CENP-E and CENP-F directly and specifically interact with distinct BUB mitotic checkpoint Ser/Thr kinases. *J Biol Chem* 293: 10084 – 10101

Cooke CA, Schaar B, Yen TJ, Earnshaw WC (1997) Localization of CENP-E in the fibrous corona and outer plate of mammalian kinetochores from prometaphase through anaphase. *Chromosoma* 106: 446 – 455

Craske B, Legal T, Welburn JPI (2022) Reconstitution of an active human CENP-E motor. *Open Biol* 12: 210389

Dephoure N, Zhou C, Villen J, Beausoleil SA, Bakalarski CE, Elledge SJ, Gygi SP (2008) A quantitative atlas of mitotic phosphorylation. *Proc Natl Acad Sci USA* 105: 10762 – 10767

Douglas ME, Mishima M (2010) Still entangled: assembly of the central spindle by multiple microtubule modulators. *Semin Cell Dev Biol* 21: 899 – 908

Forth S, Hsia KC, Shimamoto Y, Kapoor TM (2014) Asymmetric friction of nonmotor MAPs can lead to their directional motion in active microtubule networks. *Cell* 157: 420 – 432

Gaska I, Armstrong ME, Alfieri A, Forth S (2020) The mitotic crosslinking protein PRC1 acts like a mechanical dashpot to resist microtubule sliding. *Dev Cell* 54: 367 – 378

Glotzer M (2009) The 3Ms of central spindle assembly: microtubules, motors and MAPs. *Nat Rev Mol Cell Biol* 10: 9 – 20

Gouet P, Courcelle E, Stuart DI, Metoz F (1999) ESPript: analysis of multiple sequence alignments in PostScript. *Bioinformatics* 15: 305 – 308

Gruneberg U, Neef R, Honda R, Nigg EA, Barr FA (2004) Relocation of Aurora B from centromeres to the central spindle at the metaphase to anaphase transition requires MKlp2. *J Cell Biol* 166: 167 – 172

Gruneberg U, Neef R, Li X, Chan EH, Chalamalasetty RB, Nigg EA, Barr FA (2006) KIF14 and citron kinase act together to promote efficient cytokinesis. *J Cell Biol* 172: 363 – 372

Gudimchuk N, Vitre B, Kim Y, Kiyatkin A, Cleveland DW, Ataullakhanov FI, Grishchuk EL (2013) Kinetochore kinesin CENP-E is a processive bi-directional tracker of dynamic microtubule tips. *Nat Cell Biol* 15: 1079 – 1088

Hannabuss J, Lera-Ramirez M, Cade NI, Fourniol FJ, Nedelec F, Surrey T (2019) Self-organization of minimal anaphase spindle midzone bundles. *Curr Biol* 29: 2120 – 2130

Hornick JE, Karanjeet K, Collins ES, Hinchcliffe EH (2010) Kinesins to the core: the role of microtubule-based motor proteins in building the mitotic spindle midzone. *Semin Cell Dev Biol* 21: 290 – 299

Hu CK, Ozlu N, Coughlin M, Steen JJ, Mitchison TJ (2012) Plk1 negatively regulates PRC1 to prevent premature midzone formation before cytokinesis. *Mol Biol Cell* 23: 2702 – 2711

Huttlin EL, Jedrychowski MP, Elias JE, Goswami T, Rad R, Beausoleil SA, Villen J, Haas W, Sowa ME, Gygi SP (2010) A tissue-specific atlas of mouse protein phosphorylation and expression. *Cell* 143: 1174 – 1189

Jagric M, Risteski P, Martincic J, Milas A, Tolic IM (2021) Optogenetic control of PRC1 reveals its role in chromosome alignment on the spindle by overlap length-dependent forces. *Elife* 10: e61170

Janson ME, Loughlin R, Loiodice I, Fu C, Brunner D, Nedelec FJ, Tran PT (2007) Crosslinkers and motors organize dynamic microtubules to form stable bipolar arrays in fission yeast. *Cell* 128: 357 – 368

Jiang W, Jimenez G, Wells NJ, Hope TJ, Wahl GM, Hunter T, Fukunaga R (1998) PRC1: a human mitotic spindle-associated CDK substrate protein required for cytokinesis. *Mol Cell* 2: 877 – 885

Jumper J, Evans R, Pritzel A, Green T, Figurnov M, Ronneberger O, Tunyasuvunakool K, Bates R, Zidek A, Potapenko A *et al* (2021) Highly accurate protein structure prediction with AlphaFold. *Nature* 596: 583 – 589

Kajtez J, Solomatina A, Novak M, Polak B, Vukusic K, Rudiger J, Cojoc G, Milas A, Sumanovac Sestak I, Risteski P *et al* (2016) Overlap microtubules link sister k-fibres and balance the forces on bi-oriented kinetochores. *Nat Commun* 7: 10298

Kettenbach AN, Schweppe DK, Faherty BK, Pechenick D, Pletnev AA, Gerber SA (2011) Quantitative phosphoproteomics identifies substrates and functional modules of Aurora and Polo-like kinase activities in mitotic cells. *Sci Signal* 4: rs5

Kurasawa Y, Earnshaw WC, Mochizuki Y, Dohmae N, Todokoro K (2004) Essential roles of KIF4 and its binding partner PRC1 in organized central spindle midzone formation. *EMBO J* 23: 3237 – 3248

Lee KY, Esmaeili B, Zealley B, Mishima M (2015) Direct interaction between centralspindlin and PRC1 reinforces mechanical resilience of the central spindle. *Nat Commun* 6: 7290

Legal T, Hayward D, Gluszek-Kustusz A, Blackburn EA, Spanos C, Rappsilber J, Gruneberg U, Welburn JPI (2020) The C-terminal helix of BubR1 is essential for CENP-E-dependent chromosome alignment. *J Cell Sci* 133: jcs246025

Lens SMA, Medema RH (2019) Cytokinesis defects and cancer. *Nat Rev Cancer* 19: 32 – 45

Liu X, Xu L, Li J, Yao PY, Wang W, Ismail H, Wang H, Liao B, Yang Z, Ward T *et al* (2020) Mitotic motor CENP-E cooperates with PRC1 in temporal control of central spindle assembly. *J Mol Cell Biol* 12: 654 – 665

Malik R, Lenobel R, Santamaria A, Ries A, Nigg EA, Korner R (2009) Quantitative analysis of the human spindle phosphoproteome at distinct mitotic stages. *J Proteome Res* 8: 4553 – 4563

McHugh T, Gluszek AA, Welburn JPI (2018) Microtubule end tethering of a processive kinesin-8 motor Kif18b is required for spindle positioning. *J Cell Biol* 217: 2403 – 2416

McKenzie C, D'Avino PP (2016) Investigating cytokinesis failure as a strategy in cancer therapy. *Oncotarget* 7: 87323 – 87341

McKinley KL, Cheeseman IM (2017) Large-scale analysis of CRISPR/Cas9 cell-cycle knockouts reveals the diversity of p53-dependent responses to cell-cycle defects. *Dev Cell* 40: 405 – 420

Mirdita M, Schutze K, Moriwaki Y, Heo L, Ovchinnikov S, Steinegger M (2022) ColabFold: making protein folding accessible to all. *Nat Methods* 19: 679 – 682

Mollinari C, Kleman JP, Jiang W, Schoehn G, Hunter T, Margolis RL (2002) PRC1 is a microtubule binding and bundling protein essential to maintain the mitotic spindle midzone. *J Cell Biol* 157: 1175 – 1186

Mollinari C, Kleman JP, Saoudi Y, Jablonski SA, Perard J, Yen TJ, Margolis RL (2005) Ablation of PRC1 by small interfering RNA demonstrates that cytokinetic abscission requires a central spindle bundle in mammalian cells, whereas completion of furrowing does not. *Mol Biol Cell* 16: 1043 – 1055

Neef R, Gruneberg U, Kopajtich R, Li X, Nigg EA, Sillje H, Barr FA (2007) Choice of Plk1 docking partners during mitosis and cytokinesis is controlled by the activation state of Cdk1. *Nat Cell Biol* 9: 436 – 444

Nousiainen M, Sillje HH, Sauer G, Nigg EA, Korner R (2006) Phosphoproteome analysis of the human mitotic spindle. *Proc Natl Acad Sci USA* 103: 5391 – 5396

Ohashi A, Ohori M, Iwai K (2016) Motor activity of centromere-associated protein-E contributes to its localization at the center of the midbody to regulate cytokinetic abscission. *Oncotarget* 7: 79964 – 79980

Olsen JV, Vermeulen M, Santamaria A, Kumar C, Miller ML, Jensen LJ, Gnad F, Cox J, Jensen TS, Nigg EA *et al* (2010) Quantitative phosphoproteomics

© 2023 The Authors

reveals widespread full phosphorylation site occupancy during mitosis. *Sci Signal* 3: ra3

Pamula MC, Carlini L, Forth S, Verma P, Suresh S, Legant WR, Khodjakov A, Betzig E, Kapoor TM (2019) High-resolution imaging reveals how the spindle midzone impacts chromosome movement. *J Cell Biol* 218: 2529–2544

Poser E, Caous R, Gruneberg U, Barr FA (2019) Aurora A promotes chromosome congression by activating the condensin-dependent pool of KIF4A. *J Cell Biol* 219: e201905194

Qian X, McDonald A, Zhou HJ, Adams ND, Parrish CA, Duffy KJ, Fitch DM, Tedesco R, Ashcraft LW, Yao B *et al* (2010) Discovery of the first potent and selective inhibitor of centromere-associated protein E: GSK923295. *ACS Med Chem Lett* 1: 30–34

Risteski P, Jagric M, Pavin N, Tolic IM (2021) Biomechanics of chromosome alignment at the spindle midplane. *Curr Biol* 31: R574–R585

Samejima K, Samejima I, Vagnarelli P, Ogawa H, Vargiu G, Kelly DA, de Lima Alves F, Kerr A, Green LC, Hudson DF *et al* (2012) Mitotic chromosomes are compacted laterally by KIF4 and condensin and axially by topoisomerase II alpha. *J Cell Biol* 199: 755–770

Santamaria A, Wang B, Elowe S, Malik R, Zhang F, Bauer M, Schmidt A, Sillje HH, Korner R, Nigg EA (2011) The Plk1-dependent phosphoproteome of the early mitotic spindle. *Mol Cell Proteomics* 10: M110.004457

Schaar BT, Chan GK, Maddox P, Salmon ED, Yen TJ (1997) CENP-E function at kinetochores is essential for chromosome alignment. *J Cell Biol* 139: 1373–1382

Schneider CA, Rasband WS, Eliceiri KW (2012) NIH Image to ImageJ: 25 years of image analysis. *Nat Methods* 9: 671–675

Serena M, Bastos RN, Elliott PR, Barr FA (2020) Molecular basis of MKLP2-dependent Aurora B transport from chromatin to the anaphase central spindle. *J Cell Biol* 219: e201910059

Sharma K, D'Souza RC, Tyanova S, Schaab C, Wisniewski JR, Cox J, Mann M (2014) Ultradeep human phosphoproteome reveals a distinct regulatory nature of Tyr and Ser/Thr-based signaling. *Cell Rep* 8: 1583–1594

Steblyanko Y, Rajendraprasad G, Osswald M, Eibes S, Jacome A, Geley S, Pereira AJ, Maiato H, Barisic M (2020) Microtubule poleward flux in human cells is driven by the coordinated action of four kinesins. *EMBO J* 39: e105432

Subramanian R, Wilson-Kubalek EM, Arthur CP, Bick MJ, Campbell EA, Darst SA, Milligan RA, Kapoor TM (2010) Insights into antiparallel microtubule crosslinking by PRC1, a conserved nonmotor microtubule binding protein. *Cell* 142: 433–443

Subramanian R, Ti SC, Tan L, Darst SA, Kapoor TM (2013) Marking and measuring single microtubules by PRC1 and kinesin-4. *Cell* 154: 377–390

Tan S (2001) A modular polycistronic expression system for overexpressing protein complexes in *Escherichia coli*. *Protein Expr Purif* 21: 224–234

Verbrugghe KJ, White JG (2004) SPD-1 is required for the formation of the spindle midzone but is not essential for the completion of cytokinesis in *C. elegans* embryos. *Curr Biol* 14: 1755–1760

Vukusic K, Ponjavic I, Buda R, Risteski P, Tolic IM (2021) Microtubule-sliding modules based on kinesins EG5 and PRC1-dependent KIF4A drive human spindle elongation. *Dev Cell* 56: 1253–1267

Wang SZ, Adler R (1995) Chromokinesin: a DNA-binding, kinesin-like nuclear protein. *J Cell Biol* 128: 761–768

Wijeratne S, Subramanian R (2018) Geometry of antiparallel microtubule bundles regulates relative sliding and stalling by PRC1 and Kif4A. *Elife* 7: e32595

Yao X, Anderson KL, Cleveland DW (1997) The microtubule-dependent motor centromere-associated protein E (CENP-E) is an integral component of kinetochore corona fibers that link centromeres to spindle microtubules. *J Cell Biol* 139: 435–447

Yen TJ, Compton DA, Wise D, Zinkowski RP, Brinkley BR, Earnshaw WC, Cleveland DW (1991) CENP-E, a novel human centromere-associated protein required for progression from metaphase to anaphase. *EMBO J* 10: 1245–1254

Zhu C, Jiang W (2005) Cell cycle-dependent translocation of PRC1 on the spindle by Kif4 is essential for midzone formation and cytokinesis. *Proc Natl Acad Sci USA* 102: 343–348

License: This is an open access article under the terms of the Creative Commons Attribution License, which permits use, distribution and reproduction in any medium, provided the original work is properly cited.

 © 2023 The Authors

Expanded View Figures

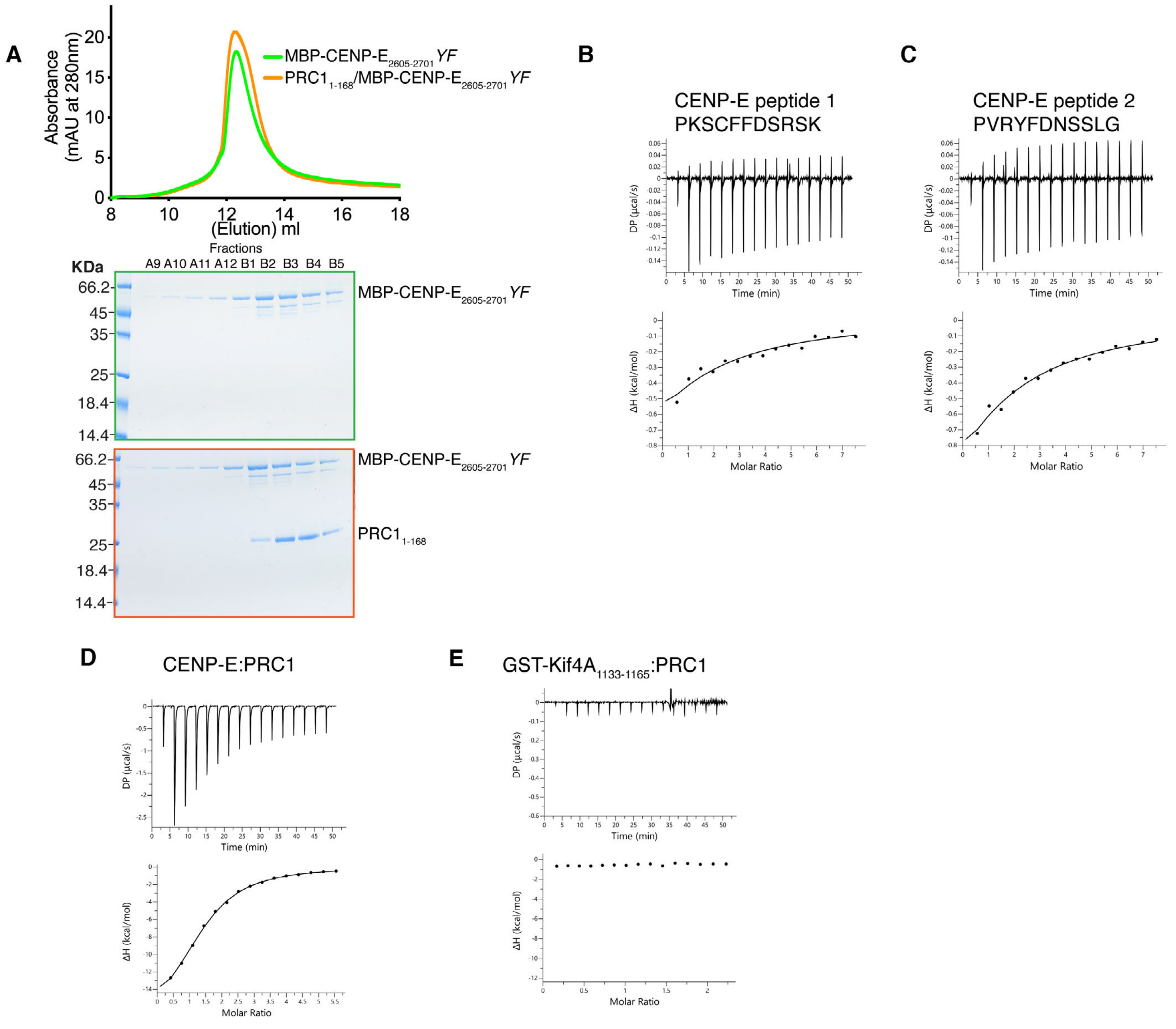

Figure EV1. Characterization of the PRC1/CENP-E interaction.

A Top. Size-exclusion chromatography elution profile of MBP-CENP-$E_{2605–2701}$*YF* (green) and MBP-CENP-$E_{2605–2701}$ *YF*/PRC$1_{1–168}$ (orange). Bottom, Coomassie-stained gel showing the size-exclusion chromatography profile of MBP-CENP-$E_{2605–2701}$ *YF* (green) and MBP-CENP-$E_{2605–2701}$ *YF*/PRC$1_{1–168}$ (orange). No shift in the elution profile was observed.

B–E Characterization by isothermal titration calorimetry of binding between PRC$1_{1–168}$ and CENP-E peptides containing 1 motif, CENP-$E_{2605–2701}$, and GST-Kif4$A_{1133–1165}$. The *y*-axis indicates kcal/mole of injectant.

© 2023 The Authors

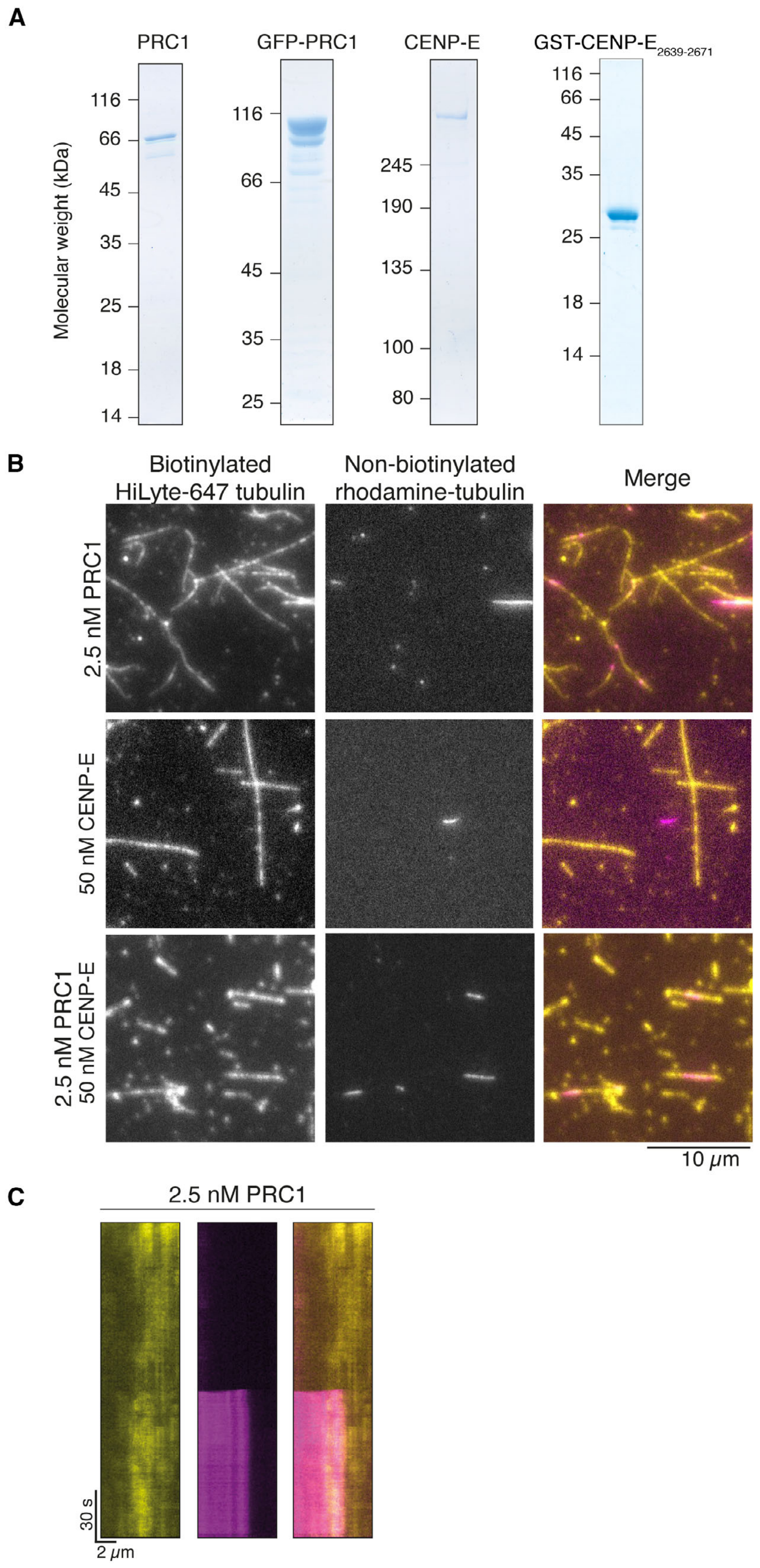

Figure EV2. Human CENP-E$_{FL}$ does not bundle microtubules in the presence of ATP.

A Coomassie-stained gel showing purified His-PRC1, His-GFP-PFR1, full-length CENP-E and GST-CENP-E$_{2639–2671}$. (B) Bundling of microtubules by 2.5 nM PRC1 *in vitro*. Non-biotinylated rhodamine tubulin (pink) and biotinylated HiLyte-647 tubulin (yellow), scalebar 5 μm.

B Crosslinking of microtubules by 2.5 nM PRC1, 50 nM CENP-E$_{FL}$ or both *in vitro*. In the absence of PRC1, rhodamine-labelled microtubules in solution (pink) are not crosslinked to biotinylated surface-bound Hilyte-647-labeled microtubules (yellow). Scalebar: 10 μm.

C Kymograph showing cross-linking of a free microtubule to an immobilized microtubule after approximately 15 s of imaging.

 © 2023 The Authors
