# Supplementary figures and images for: Phosphorylation controls spatial and temporal activities of motor‐PRC1 complexes to complete mitosis (part 1 of 2)
Source: EMBO J. 2023 Aug 18;42(21):e113647. doi: 10.15252/embj.2023113647 (PMC10620760; doi:10.15252/embj.2023113647)

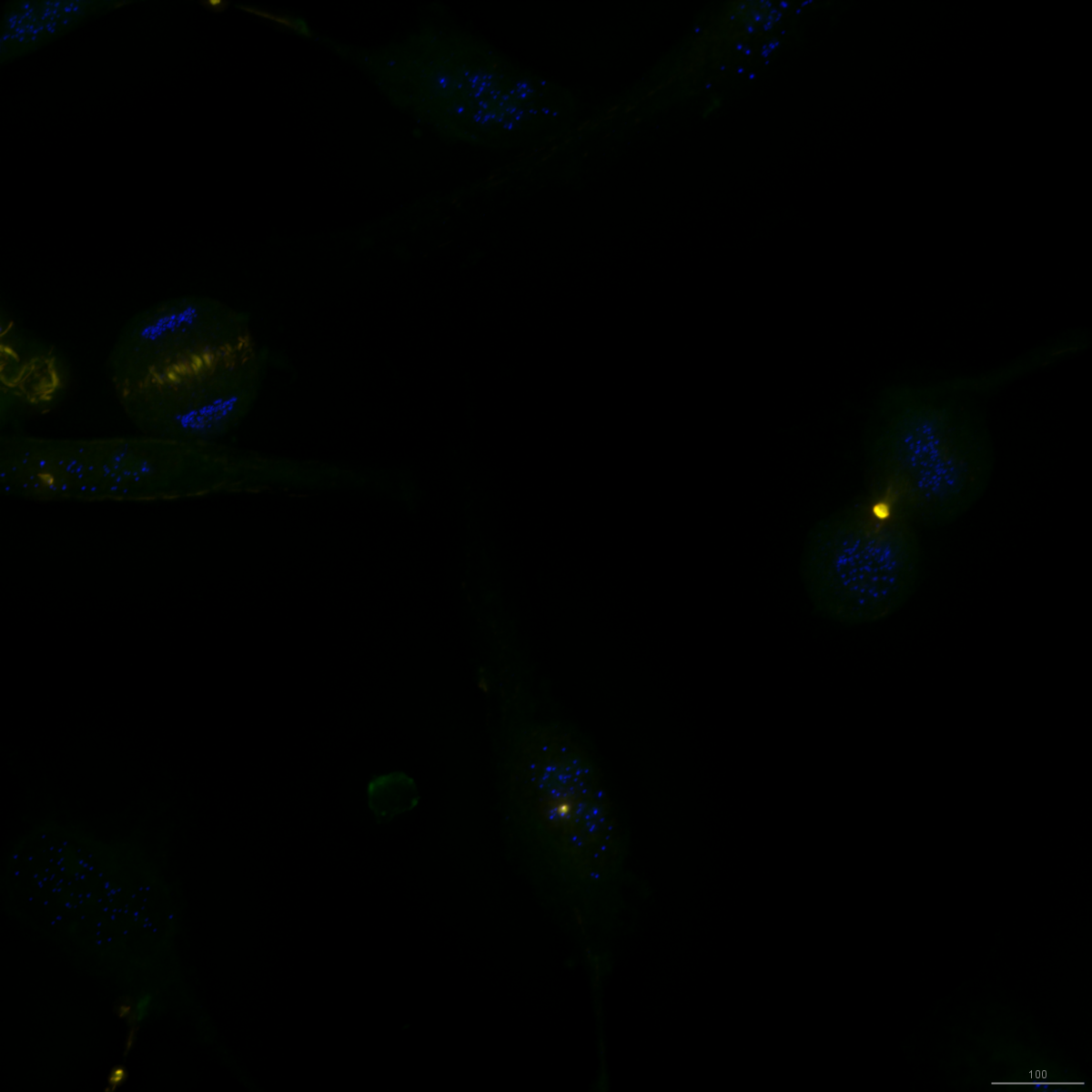

Supplement: Supplementary file 4 — Source Data for Figure 1 [file EMBJ-42-e113647-s010.zip › Figure 1/Figure 1C colocalisation of GST-GFP CENPE 2605-2701 with Prc1 /Figure 1C anaphase A and cytokinesis merged.tif]

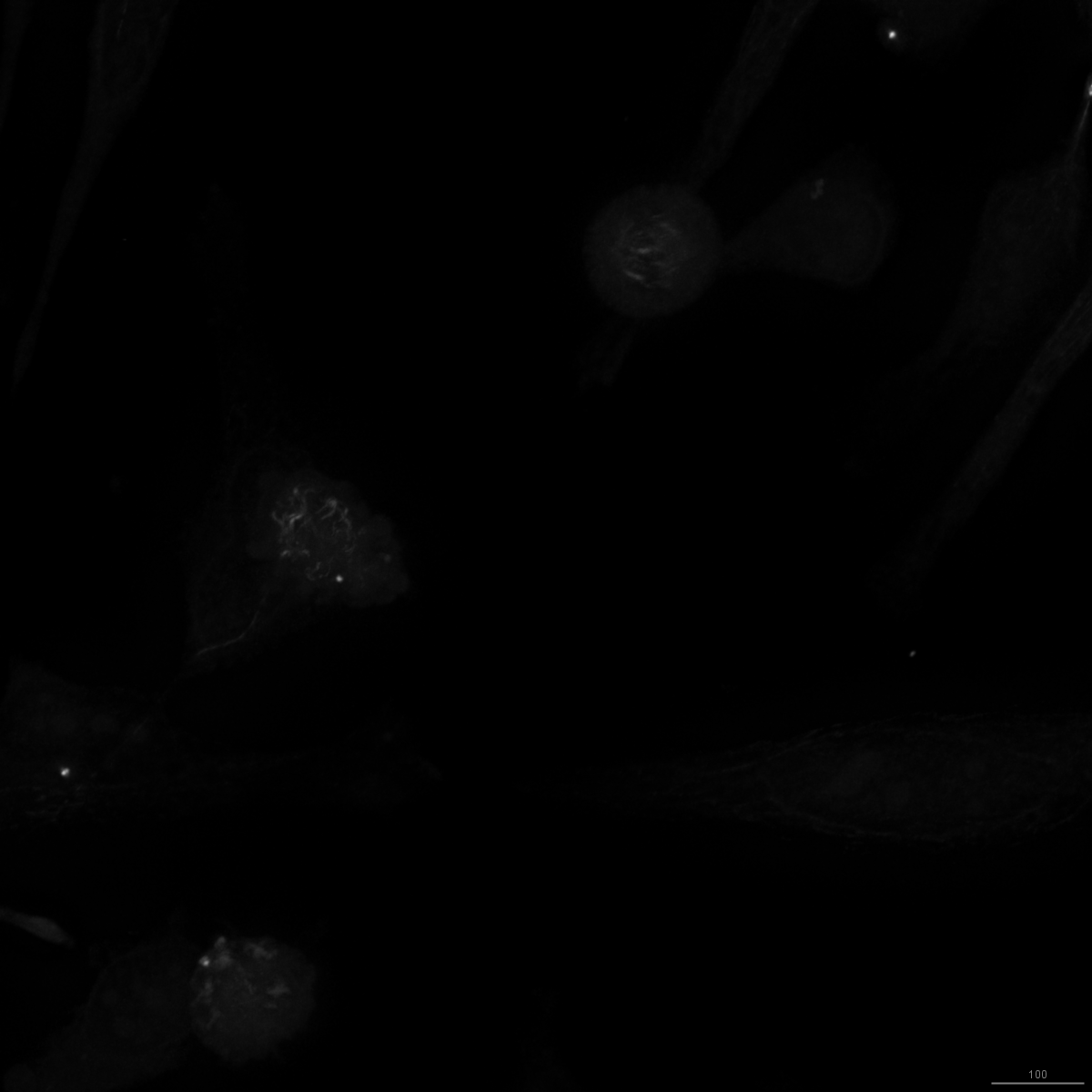

Supplement: Supplementary file 4 — Source Data for Figure 1 [file EMBJ-42-e113647-s010.zip › Figure 1/Figure 1C colocalisation of GST-GFP CENPE 2605-2701 with Prc1 /Figure 1C metaphase GFP.tif]

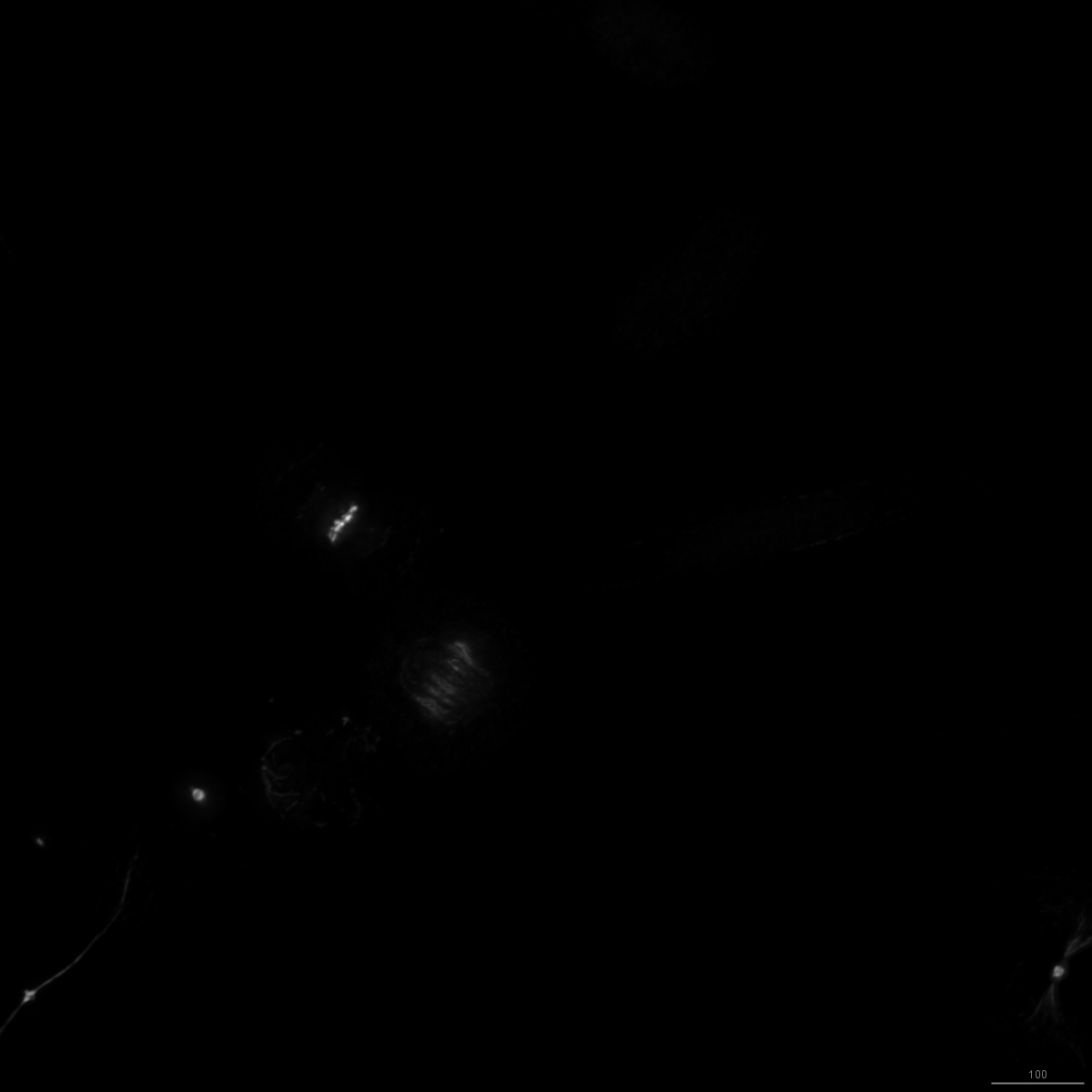

Supplement: Supplementary file 4 — Source Data for Figure 1 [file EMBJ-42-e113647-s010.zip › Figure 1/Figure 1C colocalisation of GST-GFP CENPE 2605-2701 with Prc1 /Figure 1C anaphase B and telophase PRC1.tif]

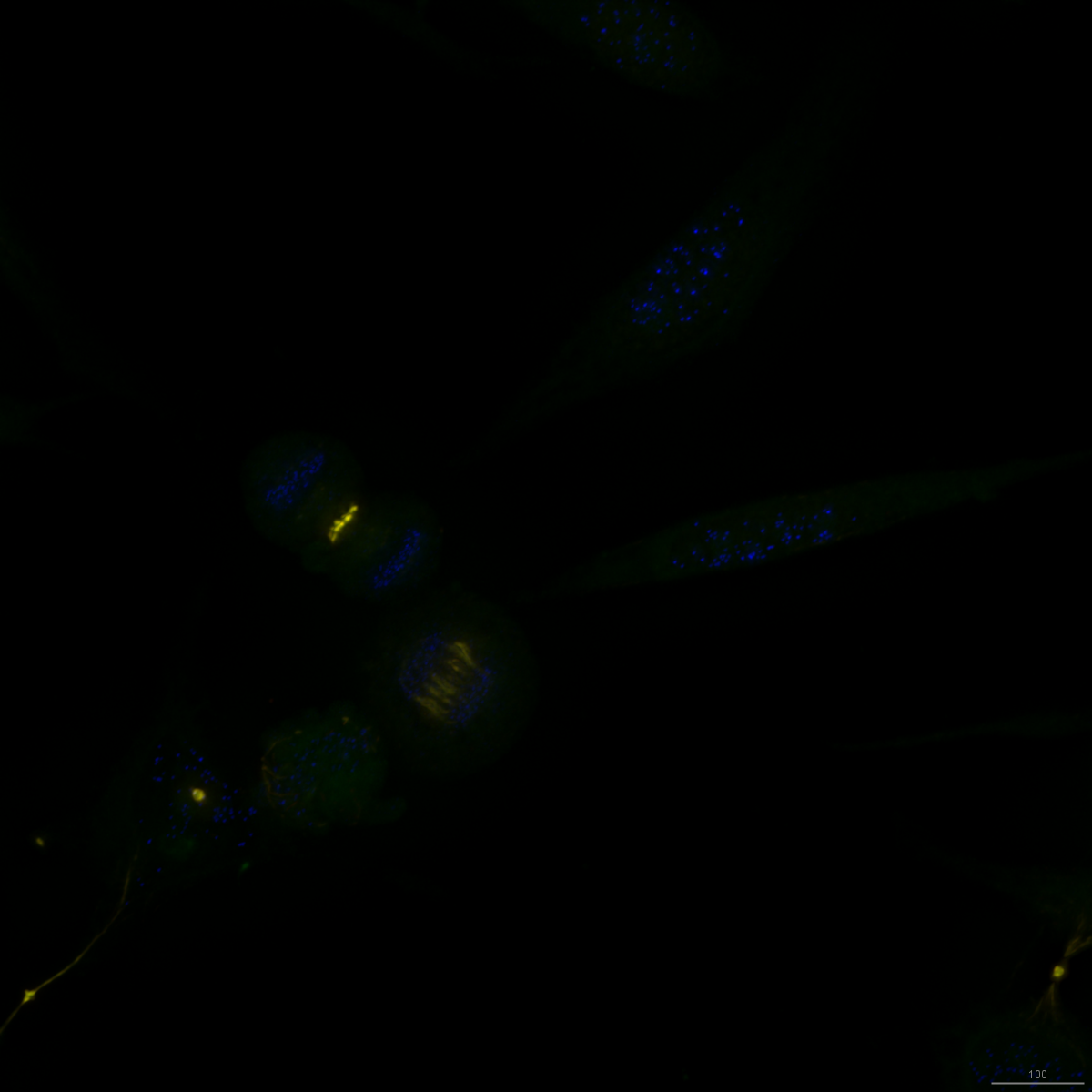

Supplement: Supplementary file 4 — Source Data for Figure 1 [file EMBJ-42-e113647-s010.zip › Figure 1/Figure 1C colocalisation of GST-GFP CENPE 2605-2701 with Prc1 /Figure 1C anaphase B and telophase merged.tif]

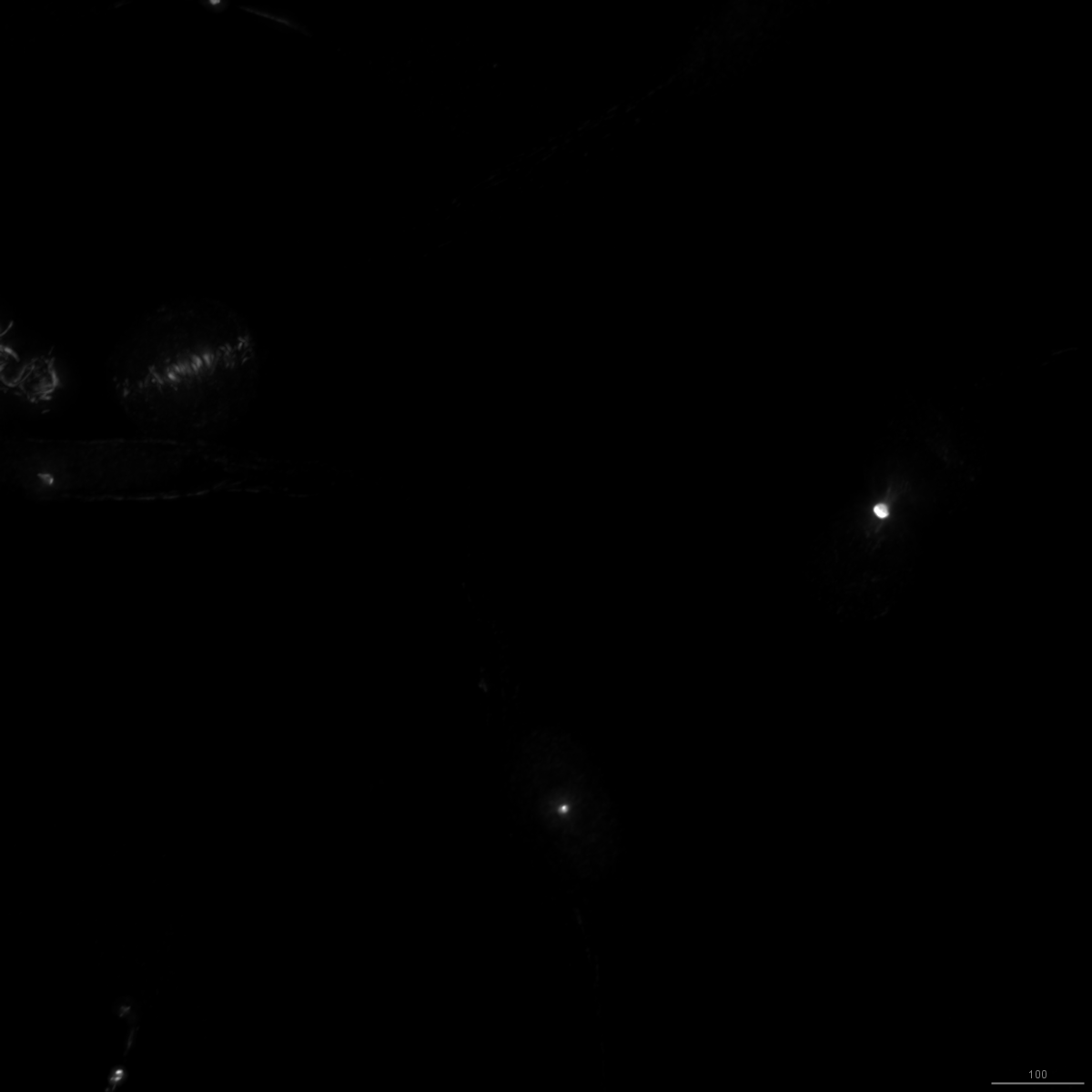

Supplement: Supplementary file 4 — Source Data for Figure 1 [file EMBJ-42-e113647-s010.zip › Figure 1/Figure 1C colocalisation of GST-GFP CENPE 2605-2701 with Prc1 /Figure 1C anaphase A and cytokinesis PRC1.tif]

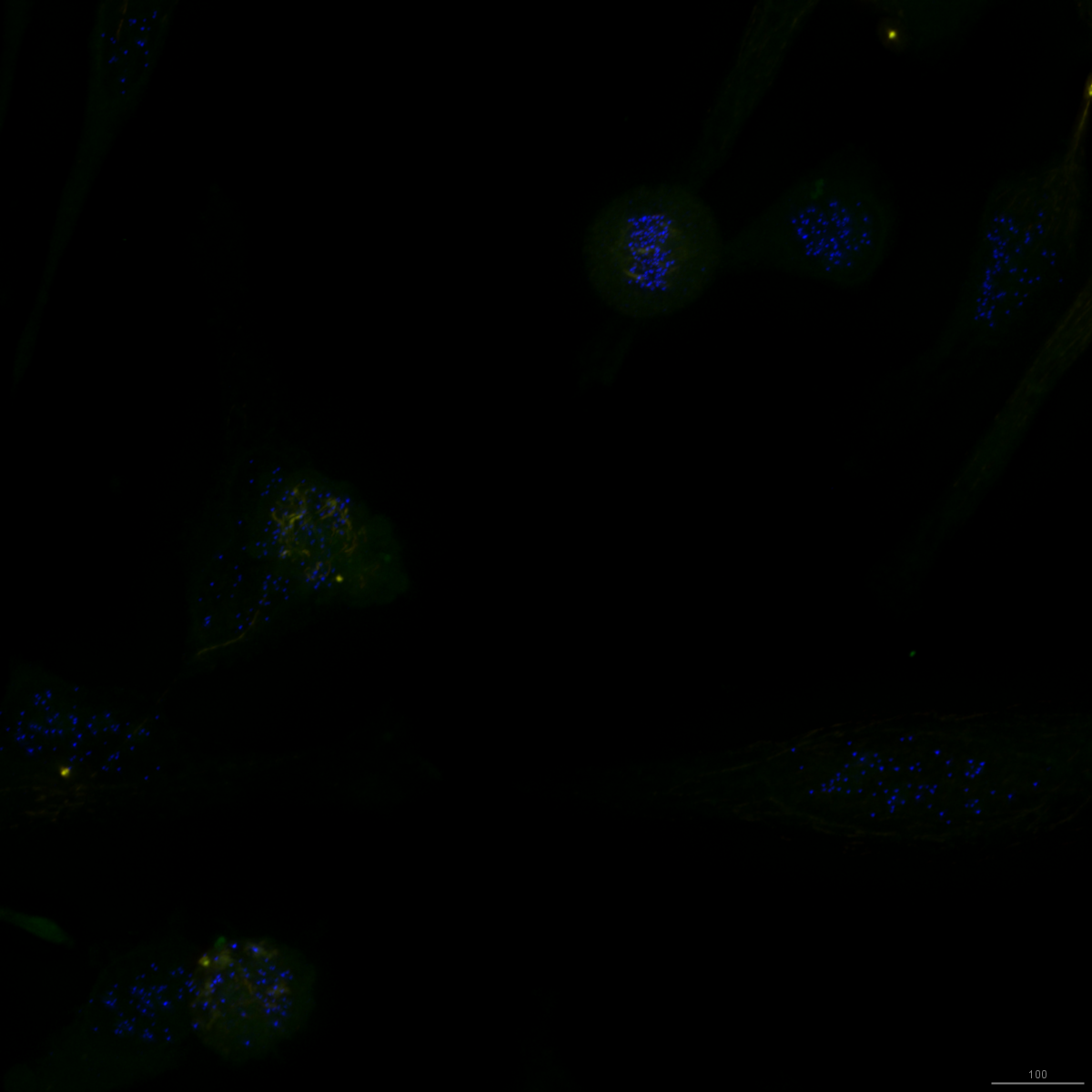

Supplement: Supplementary file 4 — Source Data for Figure 1 [file EMBJ-42-e113647-s010.zip › Figure 1/Figure 1C colocalisation of GST-GFP CENPE 2605-2701 with Prc1 /Figure 1C metaphase merged.tif]

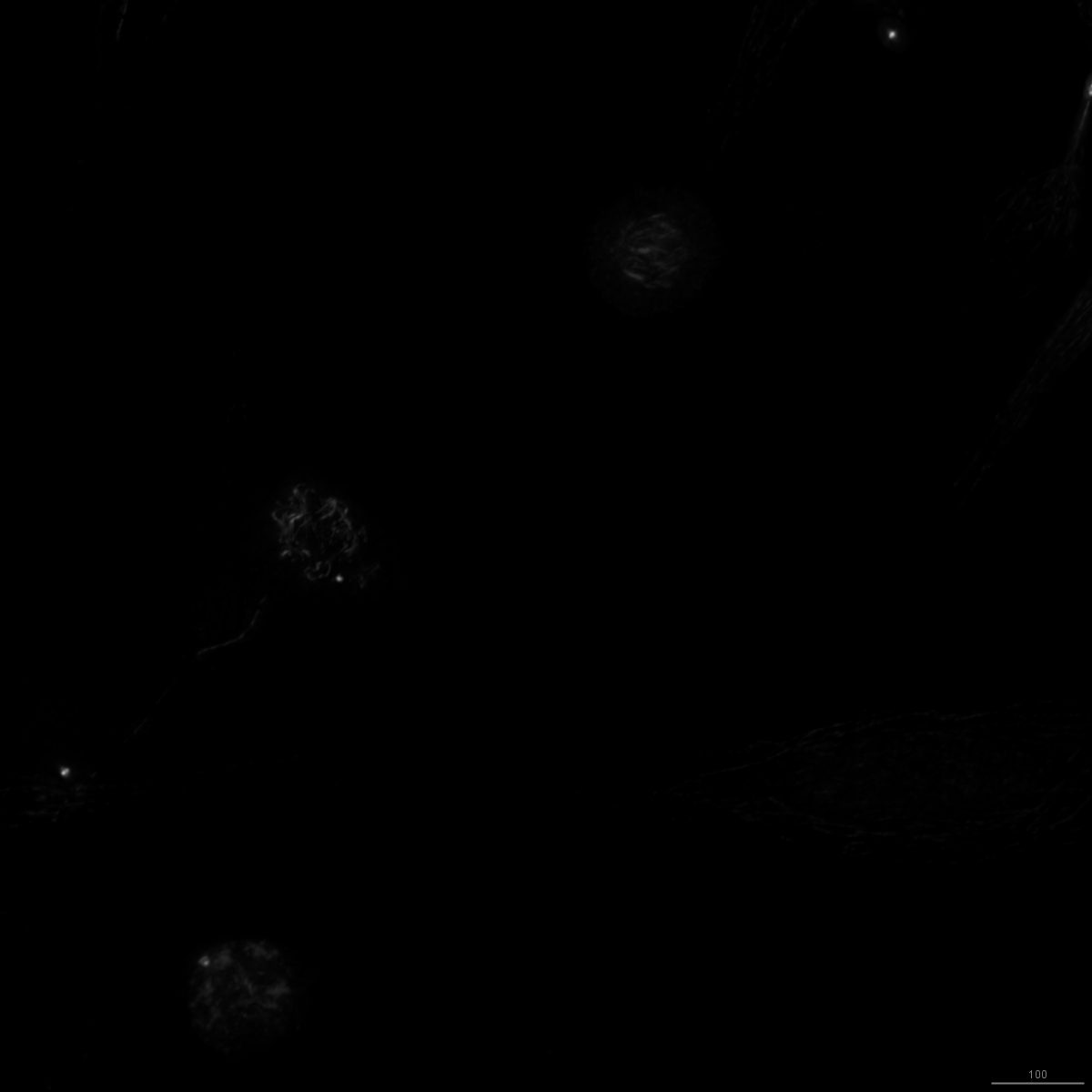

Supplement: Supplementary file 4 — Source Data for Figure 1 [file EMBJ-42-e113647-s010.zip › Figure 1/Figure 1C colocalisation of GST-GFP CENPE 2605-2701 with Prc1 /Figure 1C metaphase PRC1.tif]

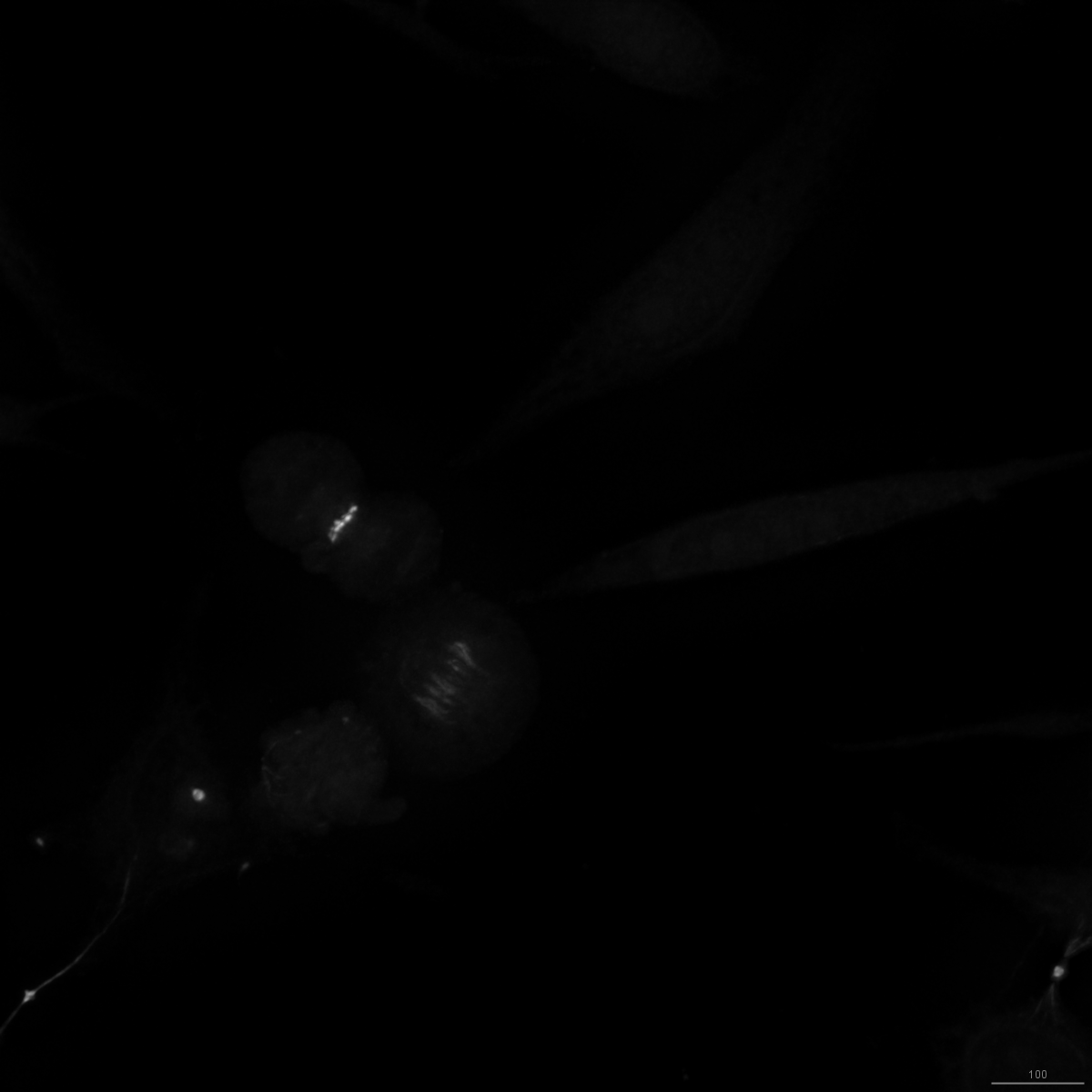

Supplement: Supplementary file 4 — Source Data for Figure 1 [file EMBJ-42-e113647-s010.zip › Figure 1/Figure 1C colocalisation of GST-GFP CENPE 2605-2701 with Prc1 /Figure 1C anaphase B and telophase GFP.tif]

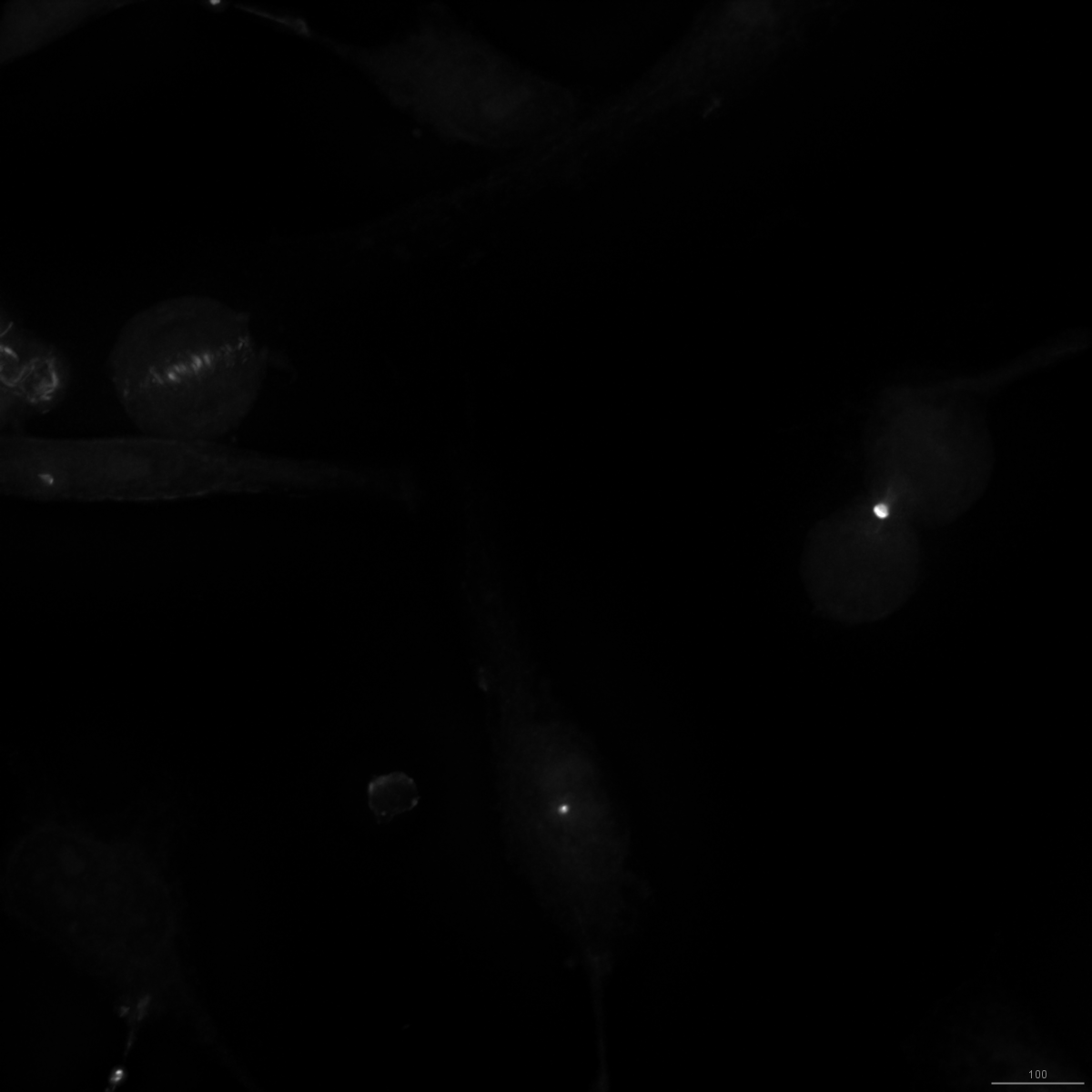

Supplement: Supplementary file 4 — Source Data for Figure 1 [file EMBJ-42-e113647-s010.zip › Figure 1/Figure 1C colocalisation of GST-GFP CENPE 2605-2701 with Prc1 /Figure 1C anaphase A and cytokinesis GFP.tif]

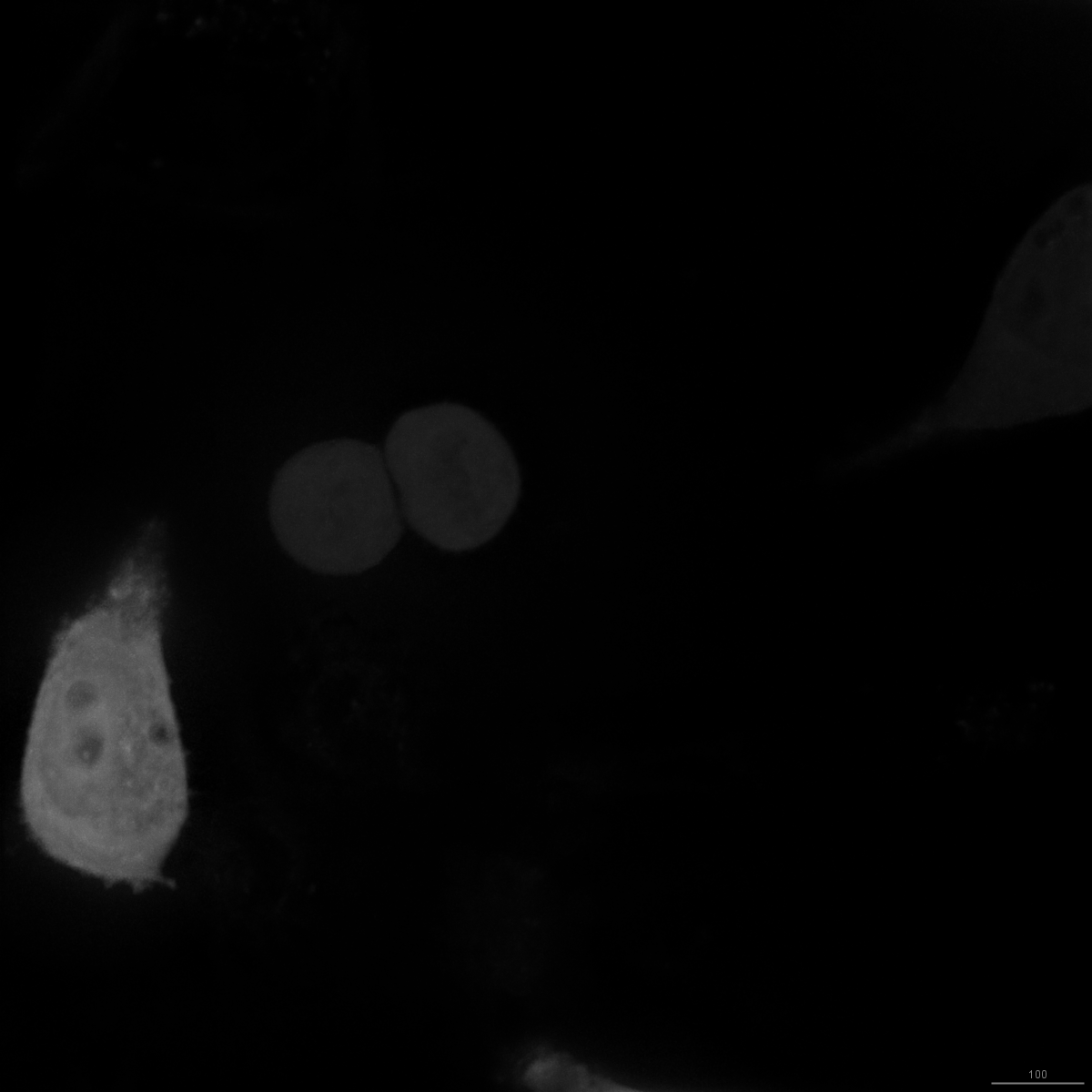

Supplement: Supplementary file 4 — Source Data for Figure 1 [file EMBJ-42-e113647-s010.zip › Figure 1/Figure 1E/YF mutant/GFPCENPE2605-2701 GFP.tif]

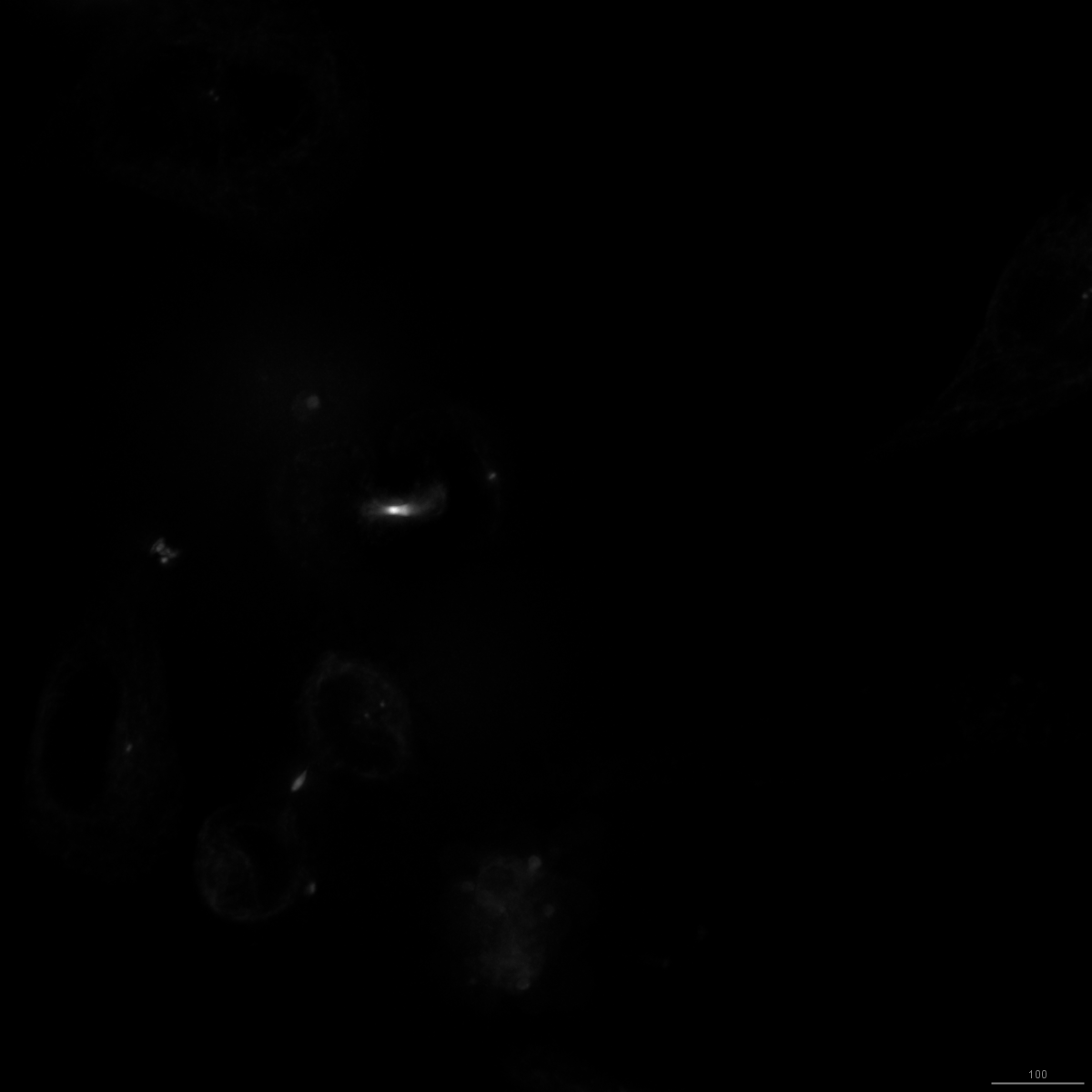

Supplement: Supplementary file 4 — Source Data for Figure 1 [file EMBJ-42-e113647-s010.zip › Figure 1/Figure 1E/YF mutant/GFPCENPE2605-2701 MT.tif]

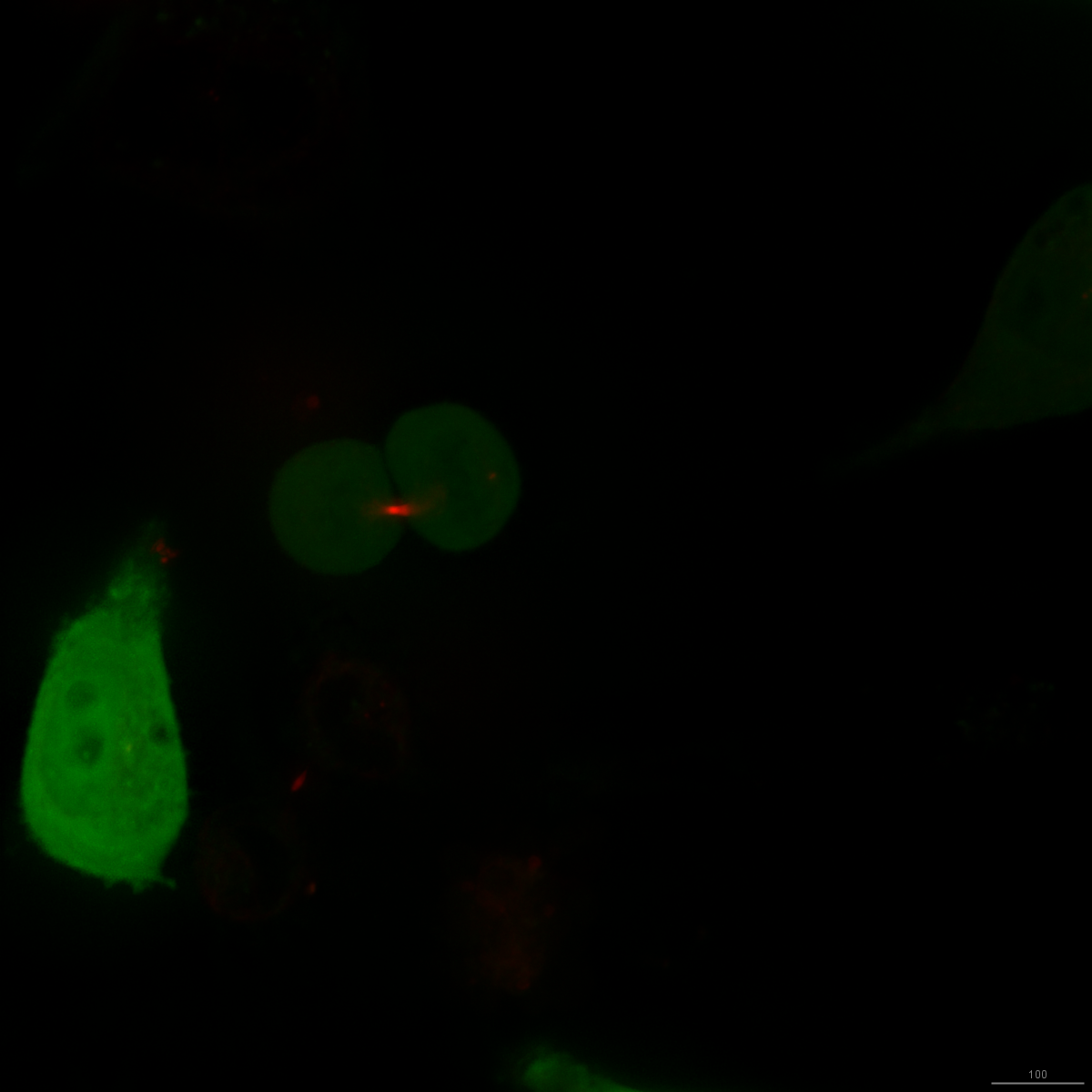

Supplement: Supplementary file 4 — Source Data for Figure 1 [file EMBJ-42-e113647-s010.zip › Figure 1/Figure 1E/YF mutant/GFPCENPE2605-2701 merge.tif]

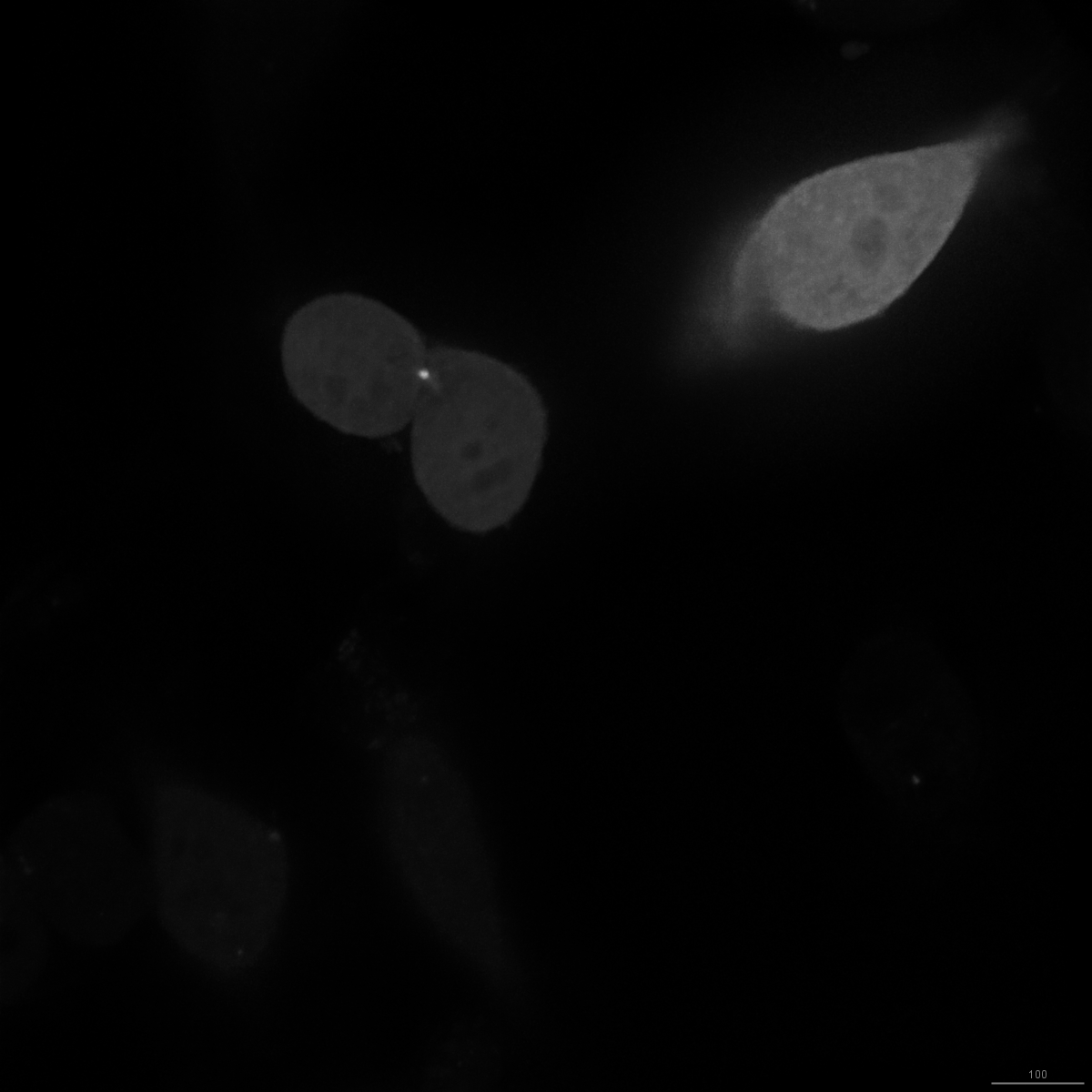

Supplement: Supplementary file 4 — Source Data for Figure 1 [file EMBJ-42-e113647-s010.zip › Figure 1/Figure 1E/FF mutant/GFPCENPE2605-2701 GFP.tif]

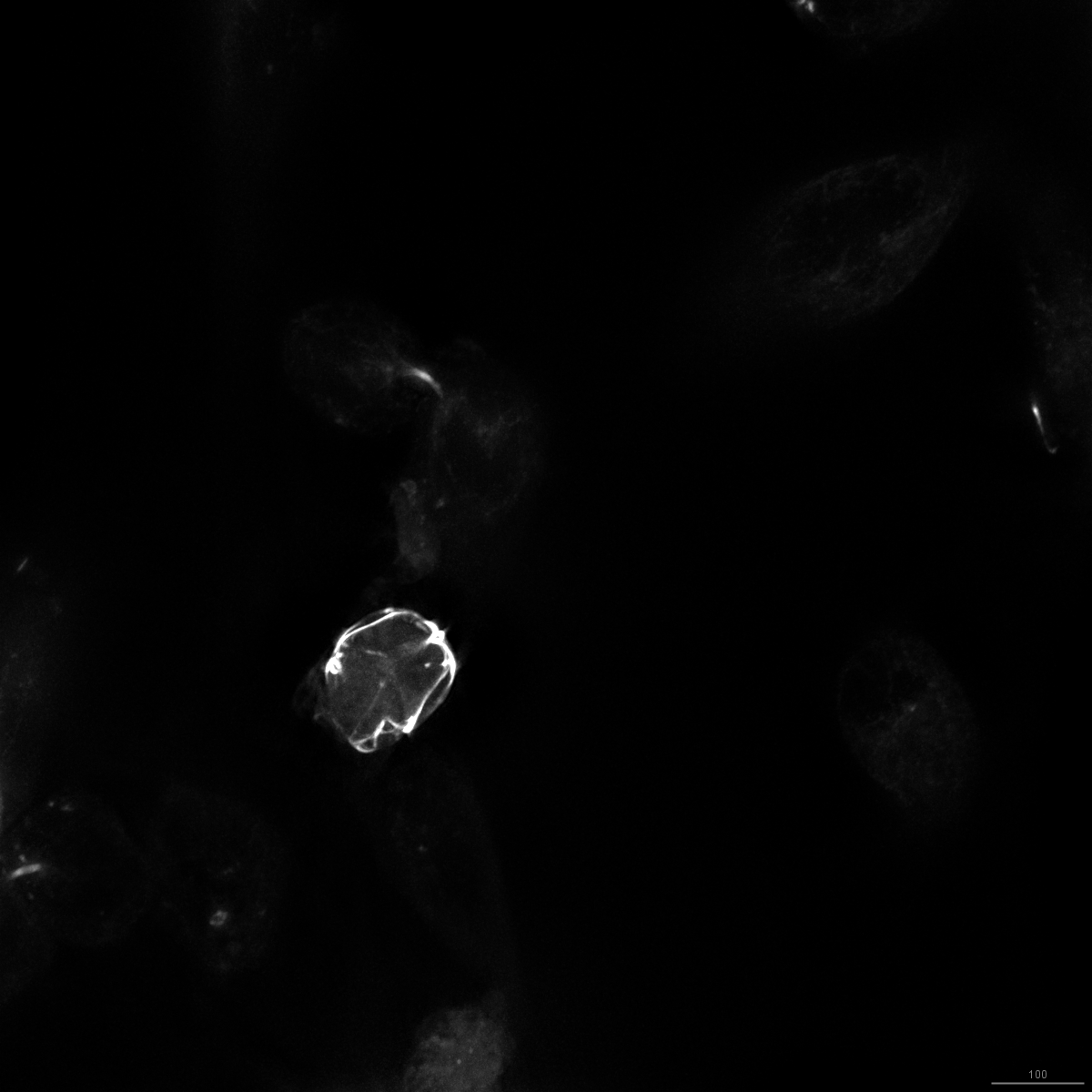

Supplement: Supplementary file 4 — Source Data for Figure 1 [file EMBJ-42-e113647-s010.zip › Figure 1/Figure 1E/FF mutant/GFPCENPE2605-2701 MT.tif]

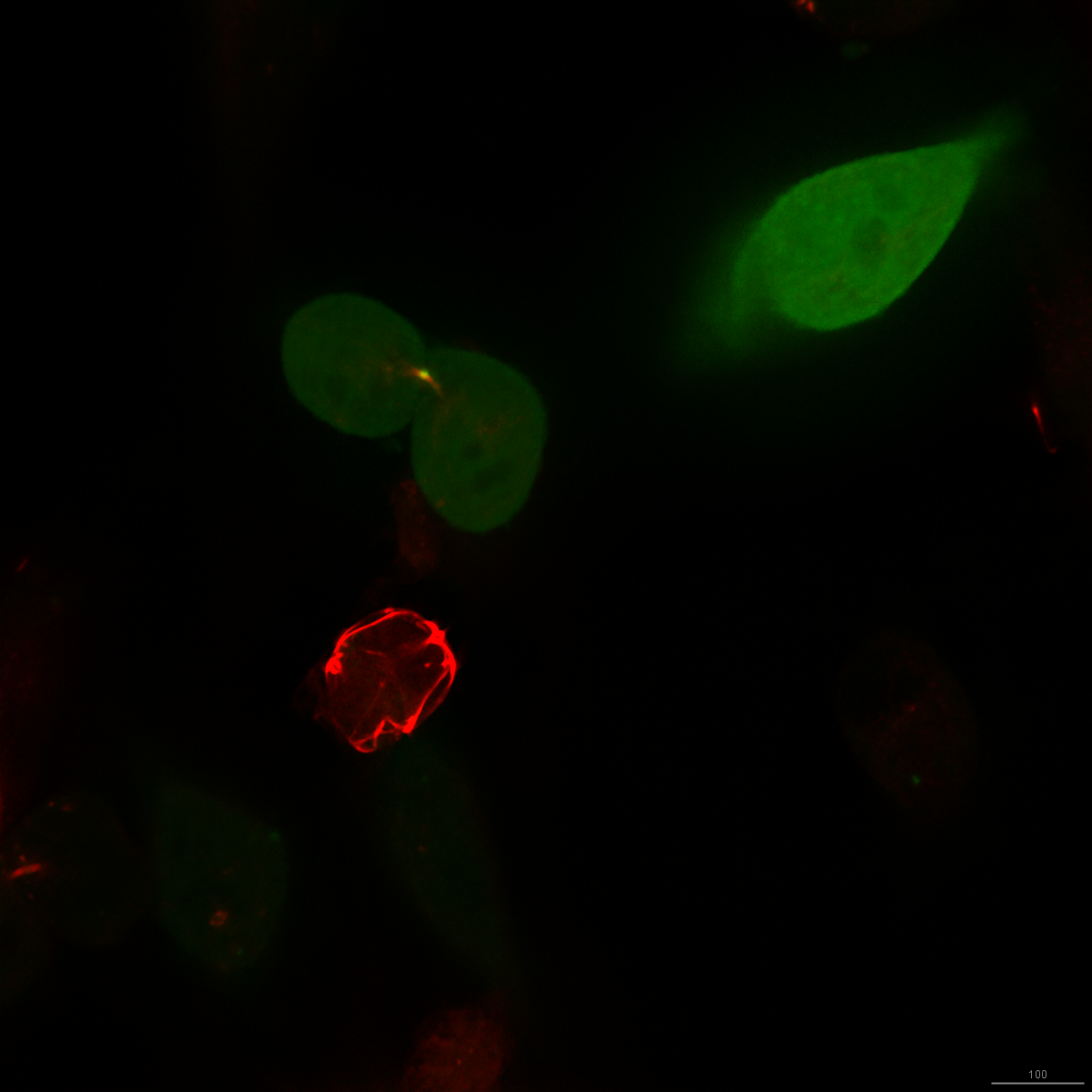

Supplement: Supplementary file 4 — Source Data for Figure 1 [file EMBJ-42-e113647-s010.zip › Figure 1/Figure 1E/FF mutant/GFPCENPE2605-2701 merge.tif]

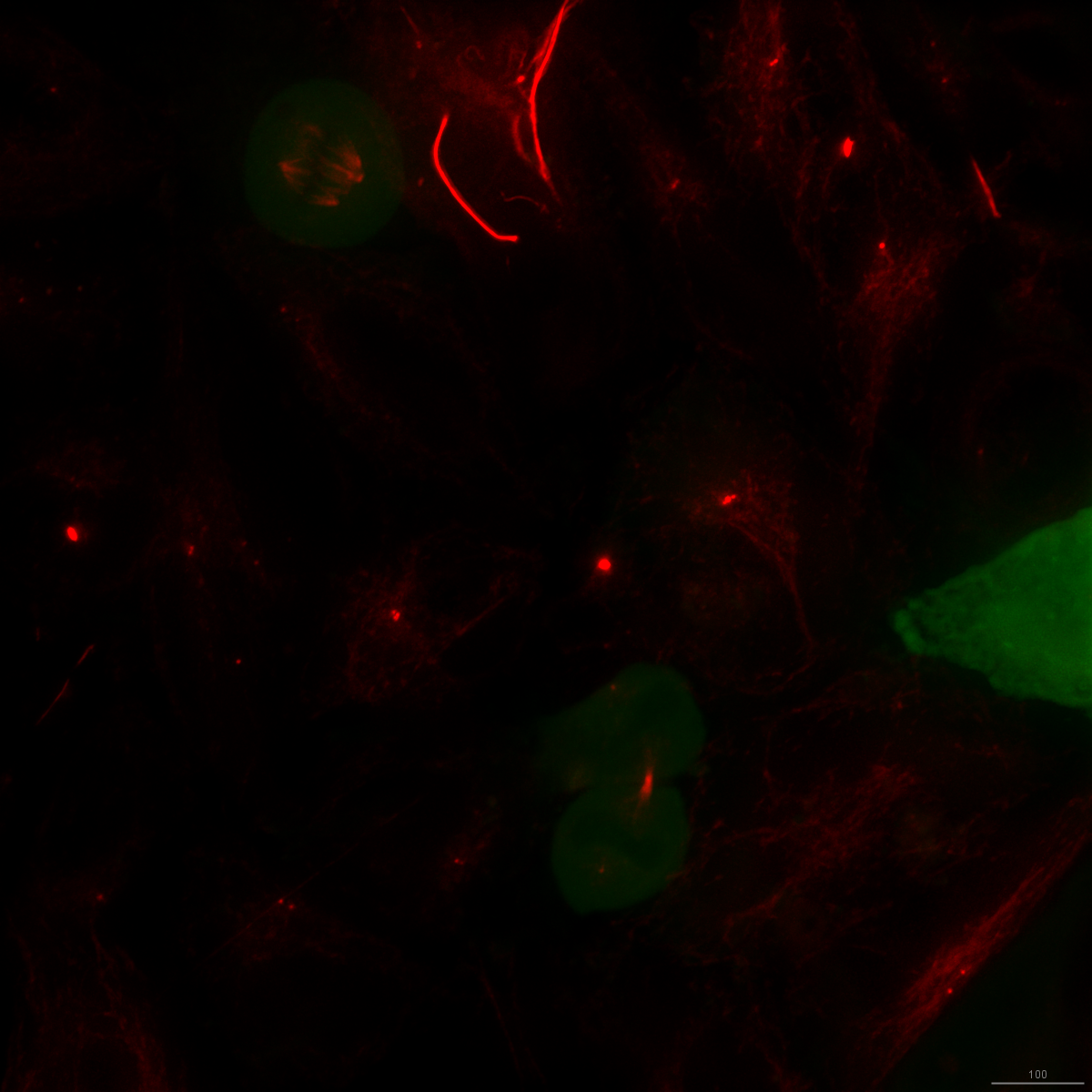

Supplement: Supplementary file 4 — Source Data for Figure 1 [file EMBJ-42-e113647-s010.zip › Figure 1/Figure 1E/FDN mutant/GFPCENPE2605-2701FDN_merge.tif]

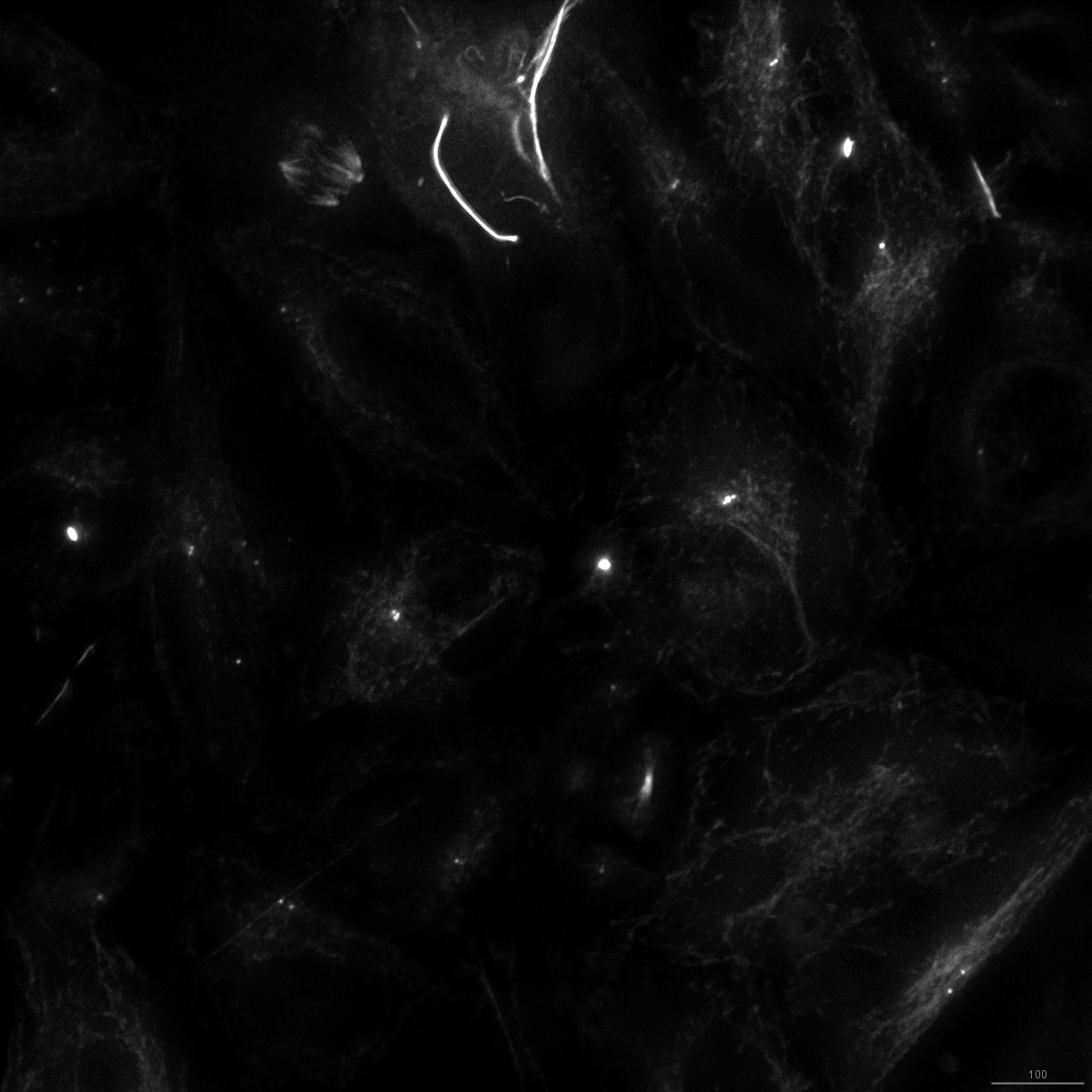

Supplement: Supplementary file 4 — Source Data for Figure 1 [file EMBJ-42-e113647-s010.zip › Figure 1/Figure 1E/FDN mutant/GFPCENPE2605-2701FDN_proj MT.tif]

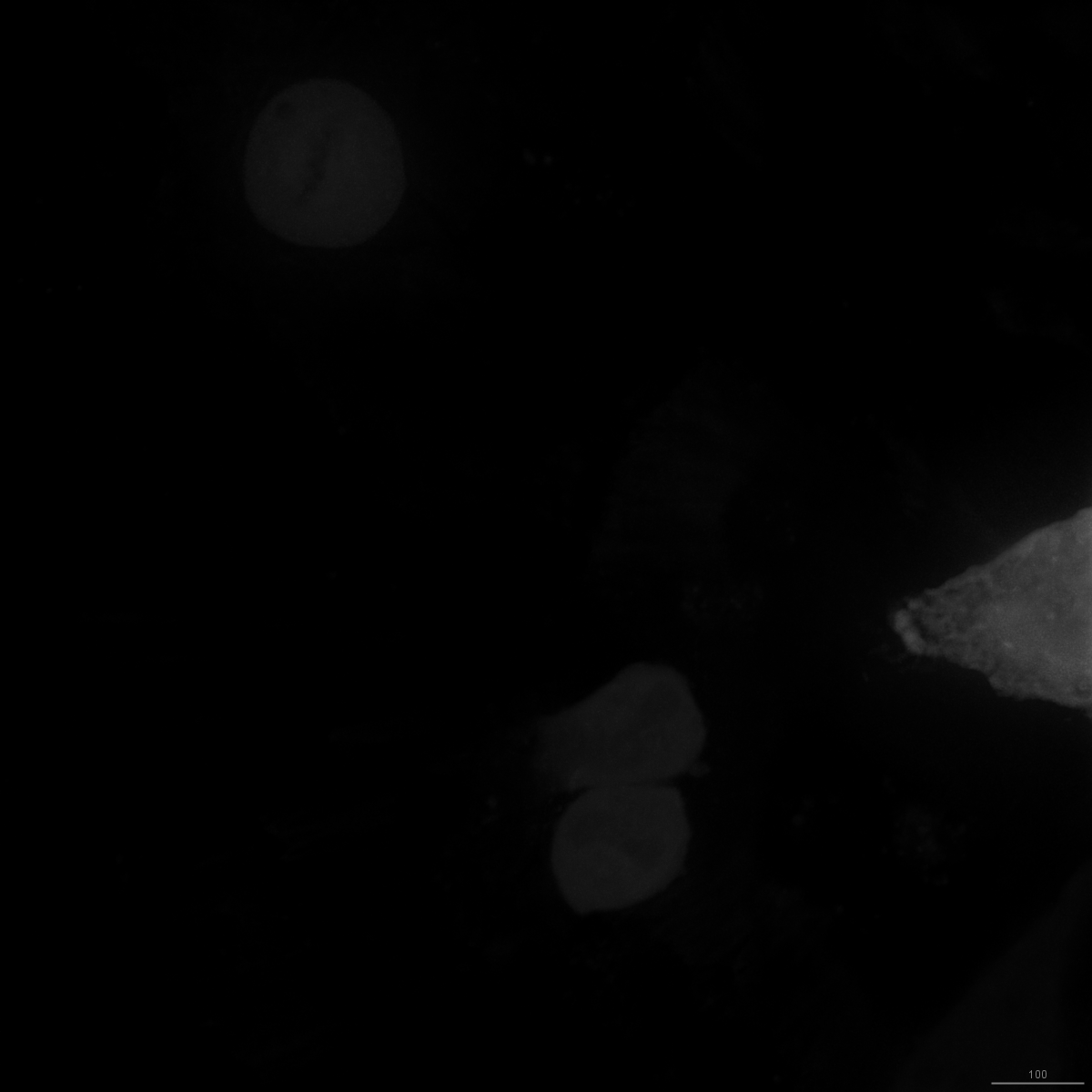

Supplement: Supplementary file 4 — Source Data for Figure 1 [file EMBJ-42-e113647-s010.zip › Figure 1/Figure 1E/FDN mutant/GFPCENPE2605-2701FDN_proj GFP.tif]

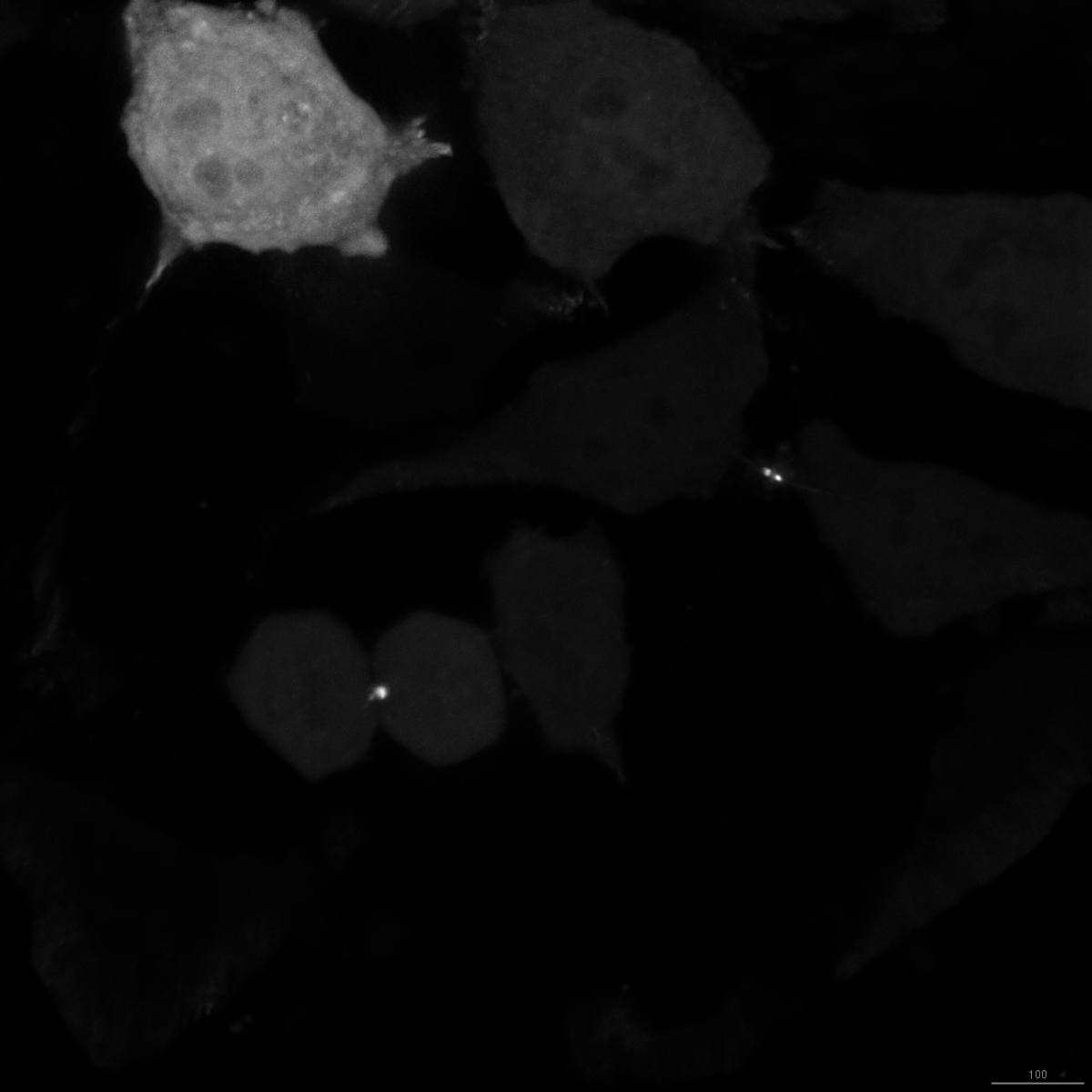

Supplement: Supplementary file 4 — Source Data for Figure 1 [file EMBJ-42-e113647-s010.zip › Figure 1/Figure 1E/WT/GFPCENPE2605-2701 GFP.tif]

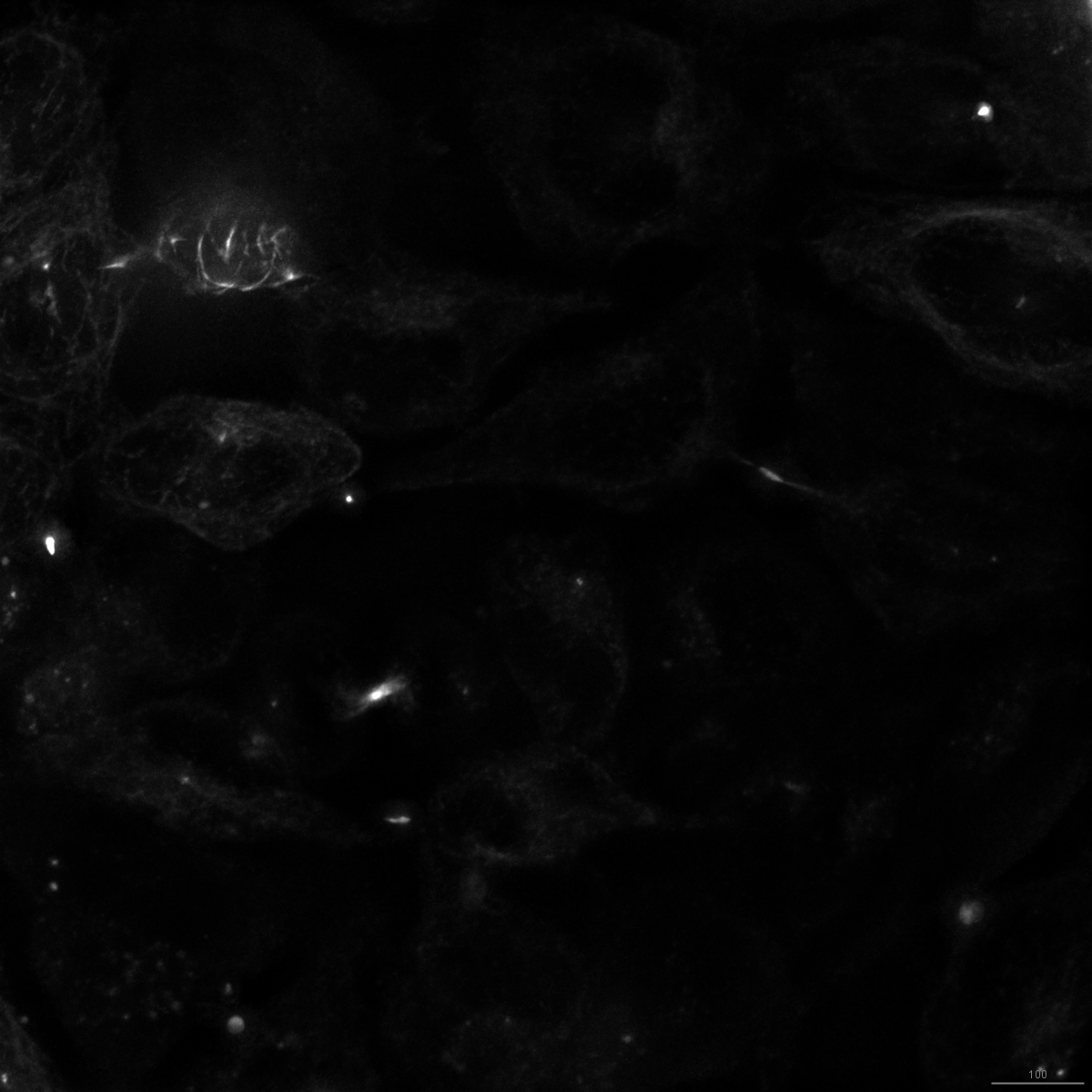

Supplement: Supplementary file 4 — Source Data for Figure 1 [file EMBJ-42-e113647-s010.zip › Figure 1/Figure 1E/WT/GFPCENPE2605-2701 MT.tif]

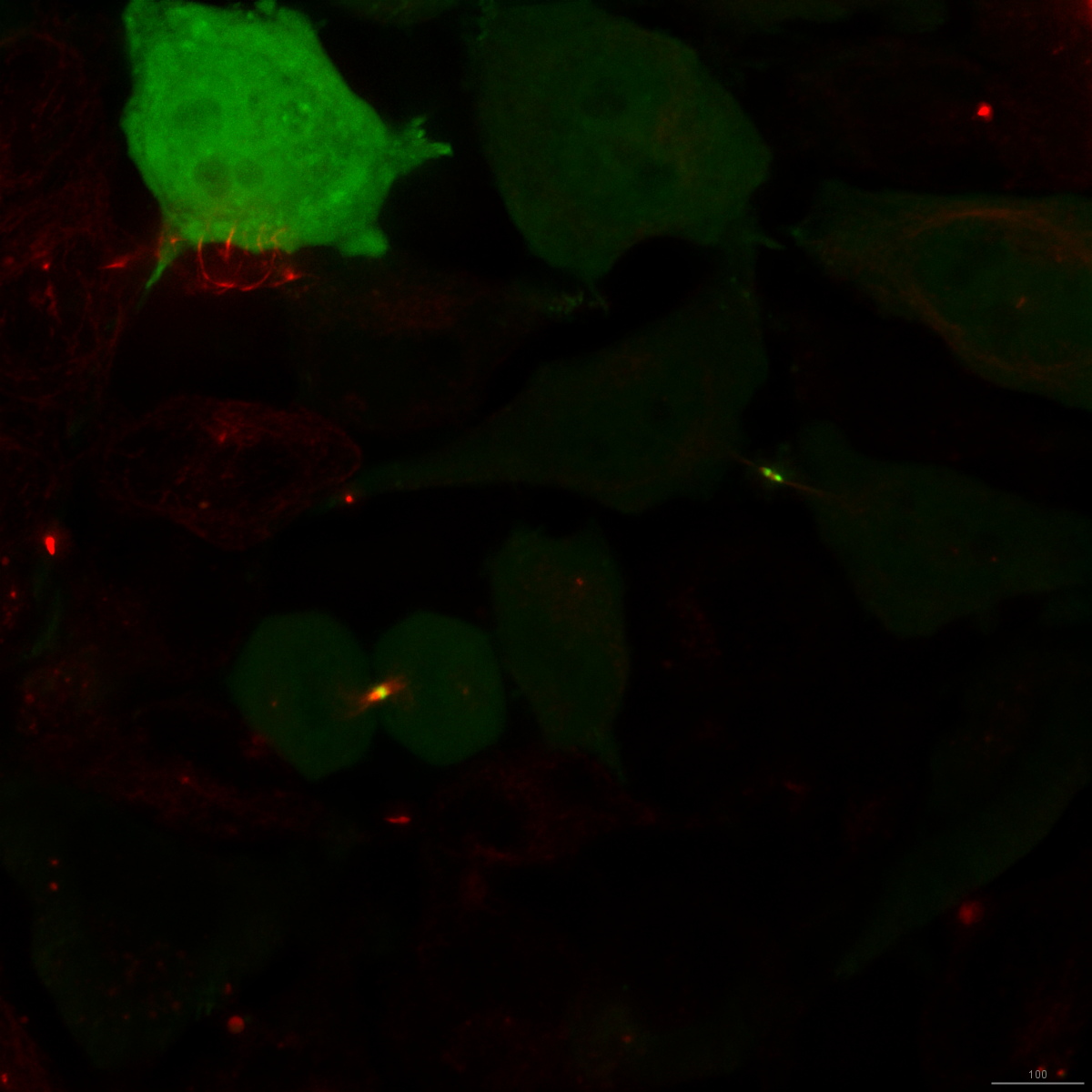

Supplement: Supplementary file 4 — Source Data for Figure 1 [file EMBJ-42-e113647-s010.zip › Figure 1/Figure 1E/WT/GFPCENPE2605-2701 merge.tif]

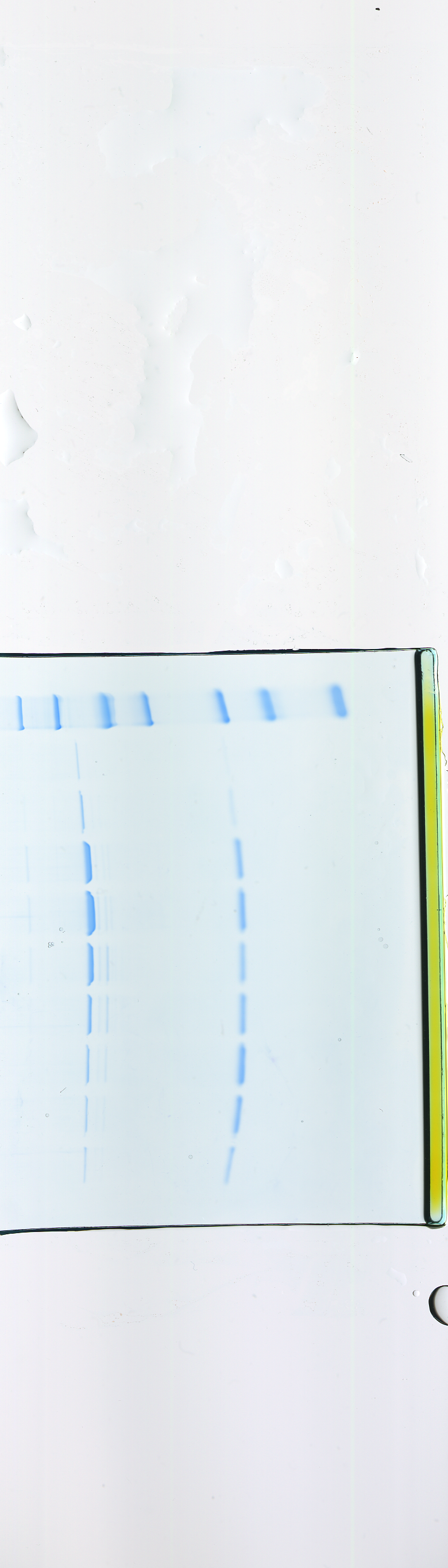

Supplement: Supplementary file 5 — Source Data for Figure 2 [file EMBJ-42-e113647-s007.zip › Figure 2/Figure 2A together binding300dpitif.tif]

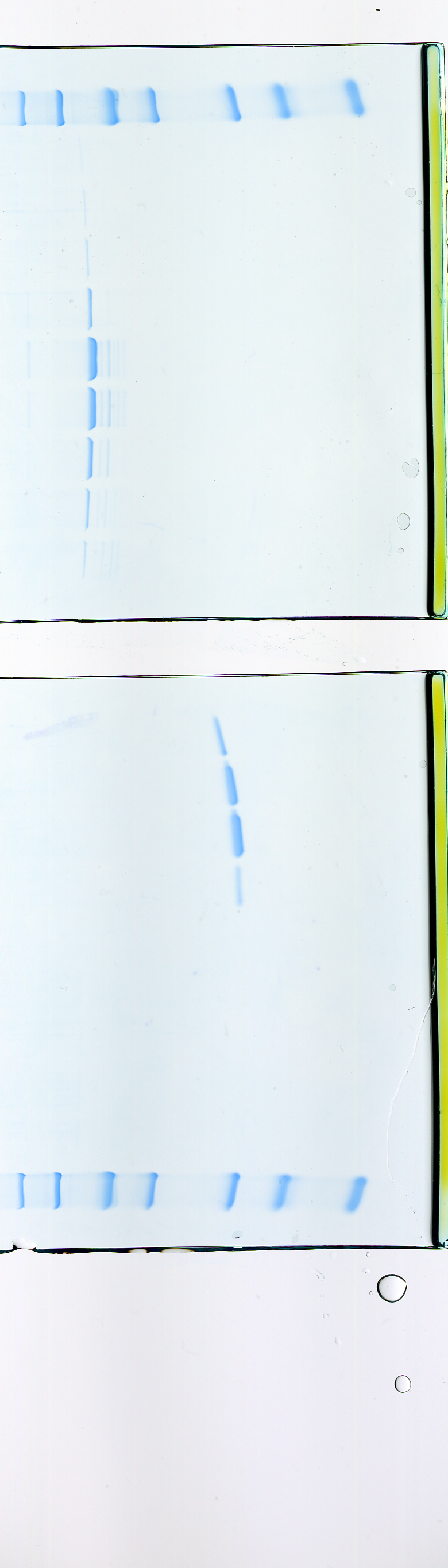

Supplement: Supplementary file 5 — Source Data for Figure 2 [file EMBJ-42-e113647-s007.zip › Figure 2/Figure 2A MBPCenpE2605-2701 (top) and Prc1 1-168 (bottom).tif]

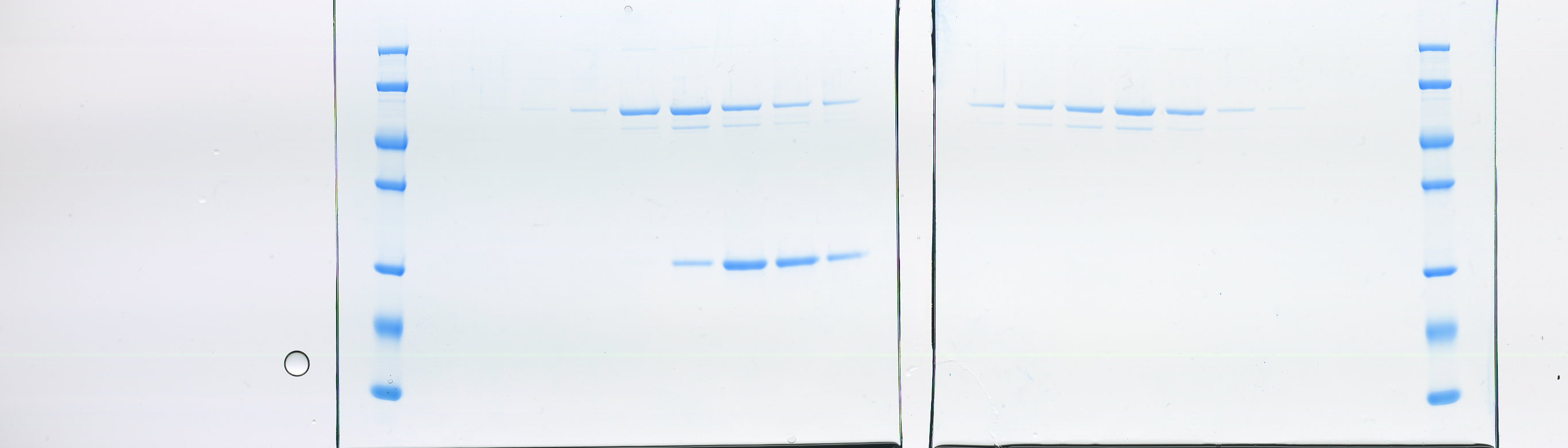

Supplement: Supplementary file 5 — Source Data for Figure 2 [file EMBJ-42-e113647-s007.zip › Figure 2/Figure 2B/S200 MBPCenpE2605-2701 FDN (top) and MBPCenpE2605-2701 FDN wirh PRC1 1-168 (bottom) 300dpi.tif]

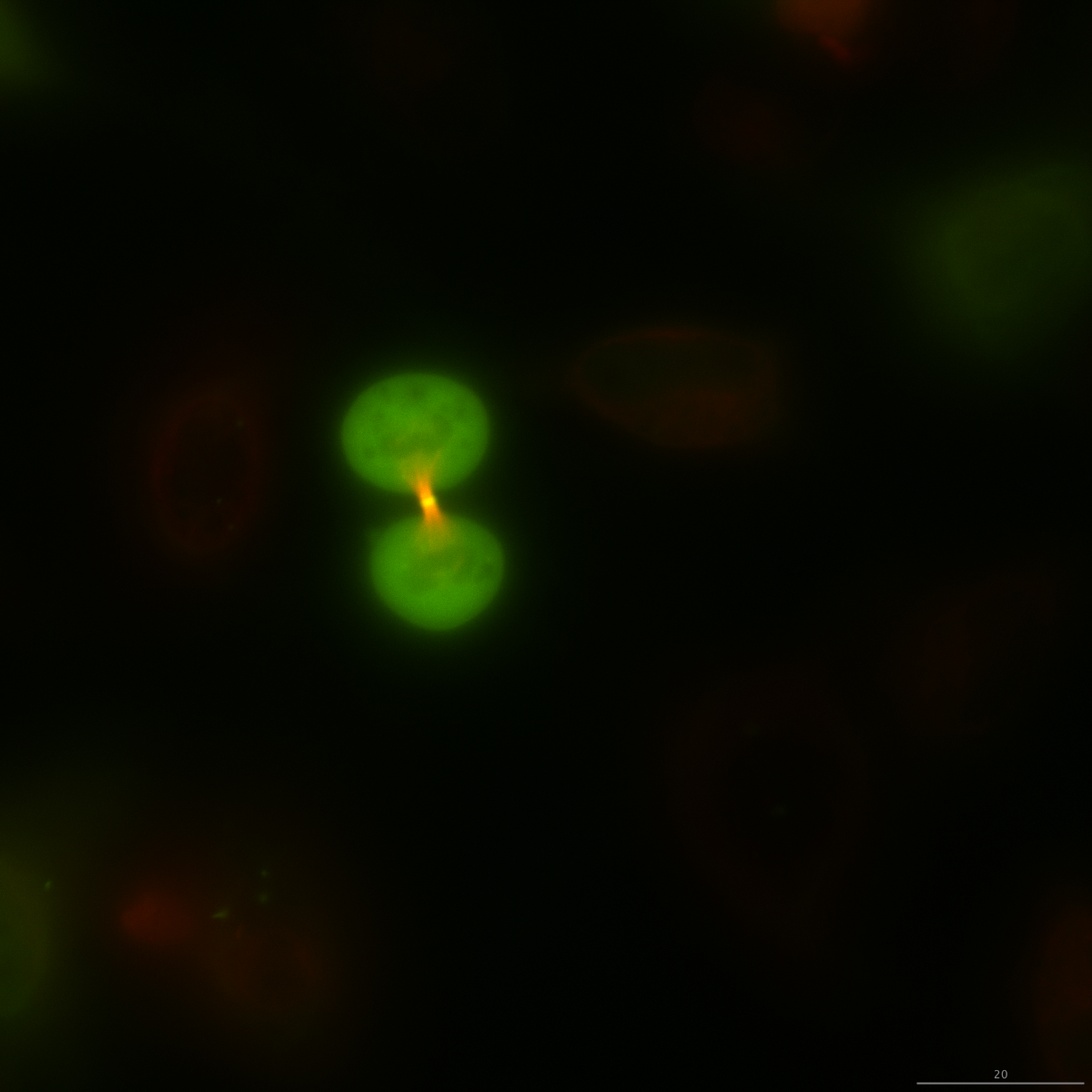

Supplement: Supplementary file 6 — Source Data for Figure 3 [file EMBJ-42-e113647-s011.zip › Figure 3/Figure 3b/ Figure 3b HeLA GFP-Kif4a1133-1232 SiR001composite.png]

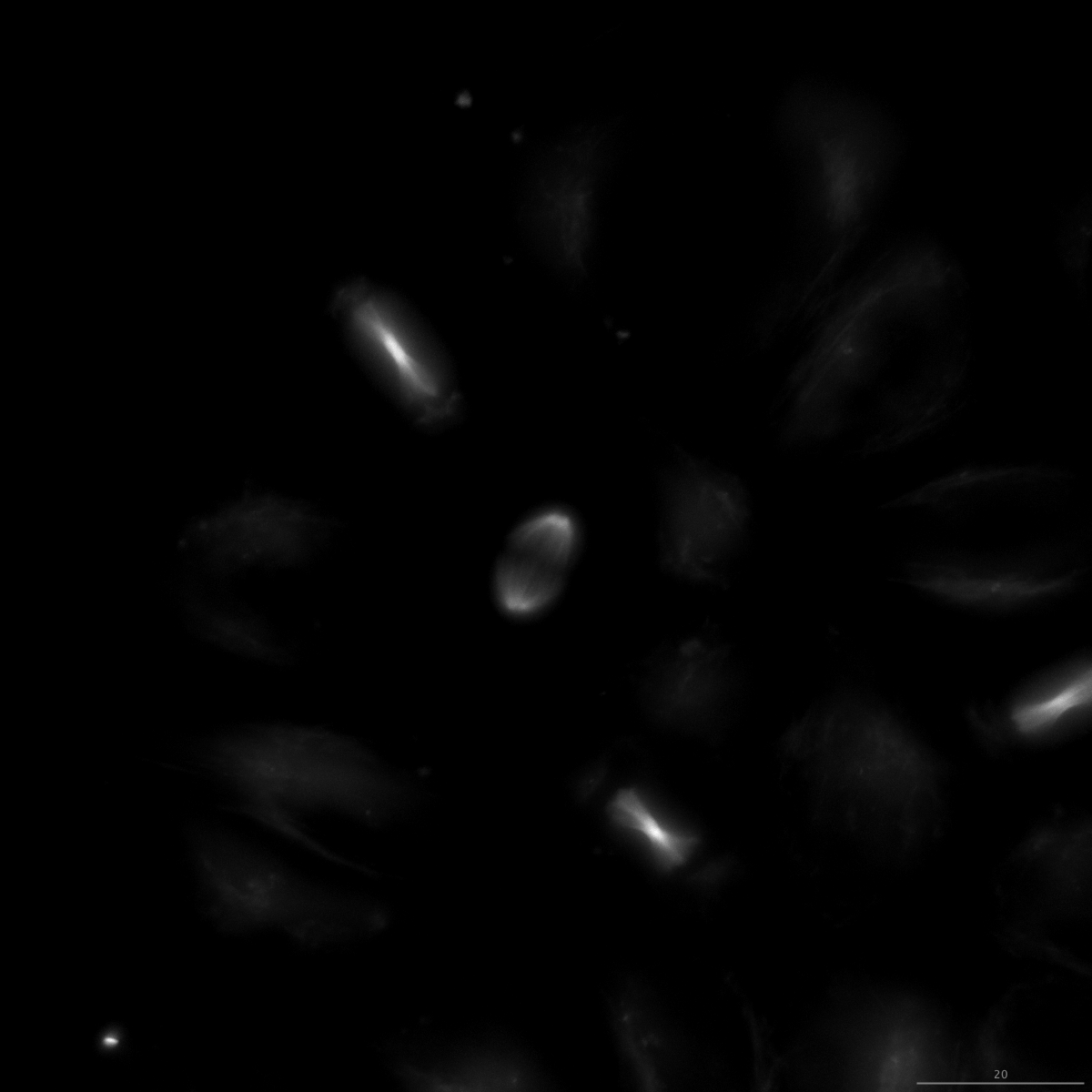

Supplement: Supplementary file 6 — Source Data for Figure 3 [file EMBJ-42-e113647-s011.zip › Figure 3/Figure 3b/CenpEcas9 GFP-Kif4a1133-1165 SiR005_projMT.png]

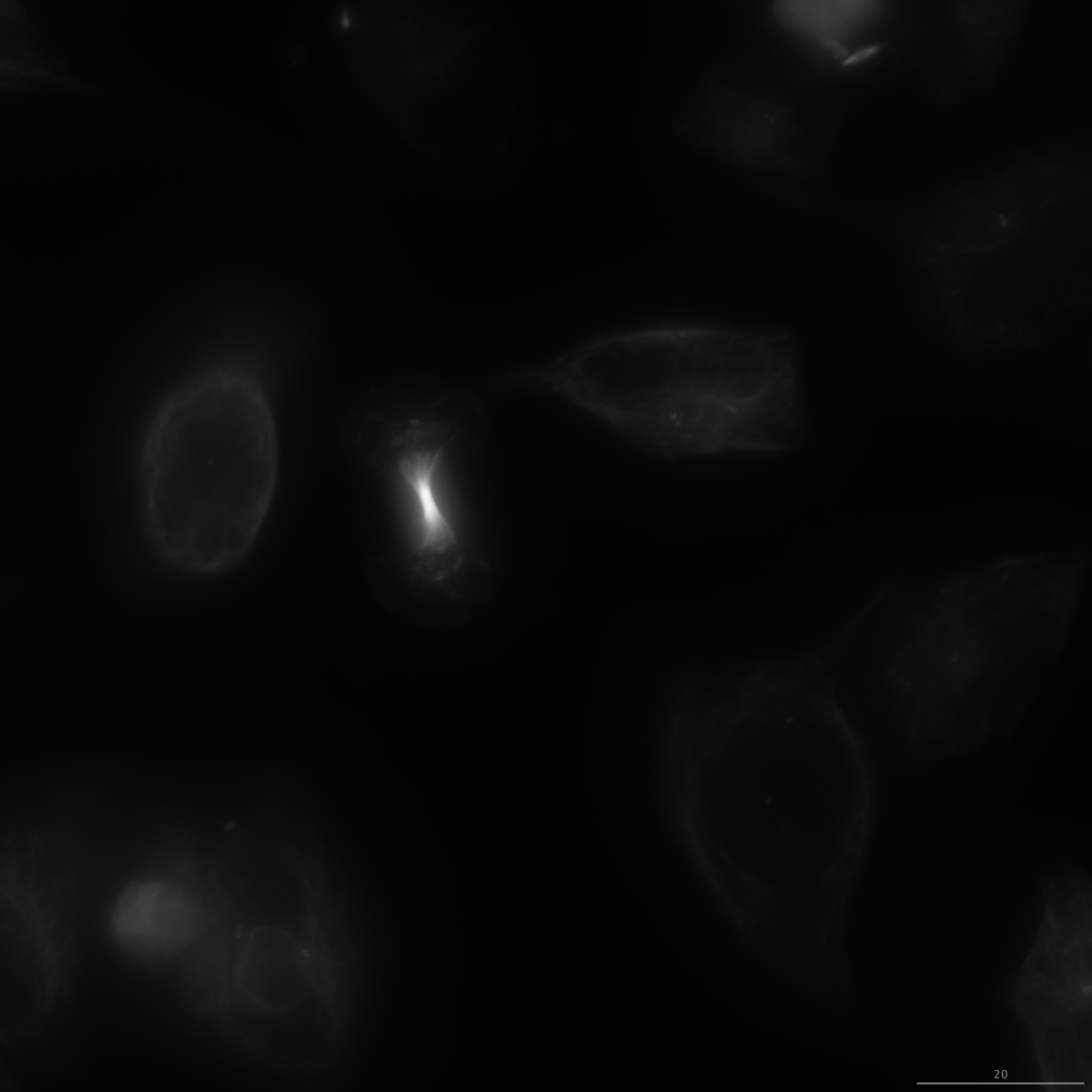

Supplement: Supplementary file 6 — Source Data for Figure 3 [file EMBJ-42-e113647-s011.zip › Figure 3/Figure 3b/Figure 3b HeLA GFP-Kif4a1133-1232 SiR001_MT.png]

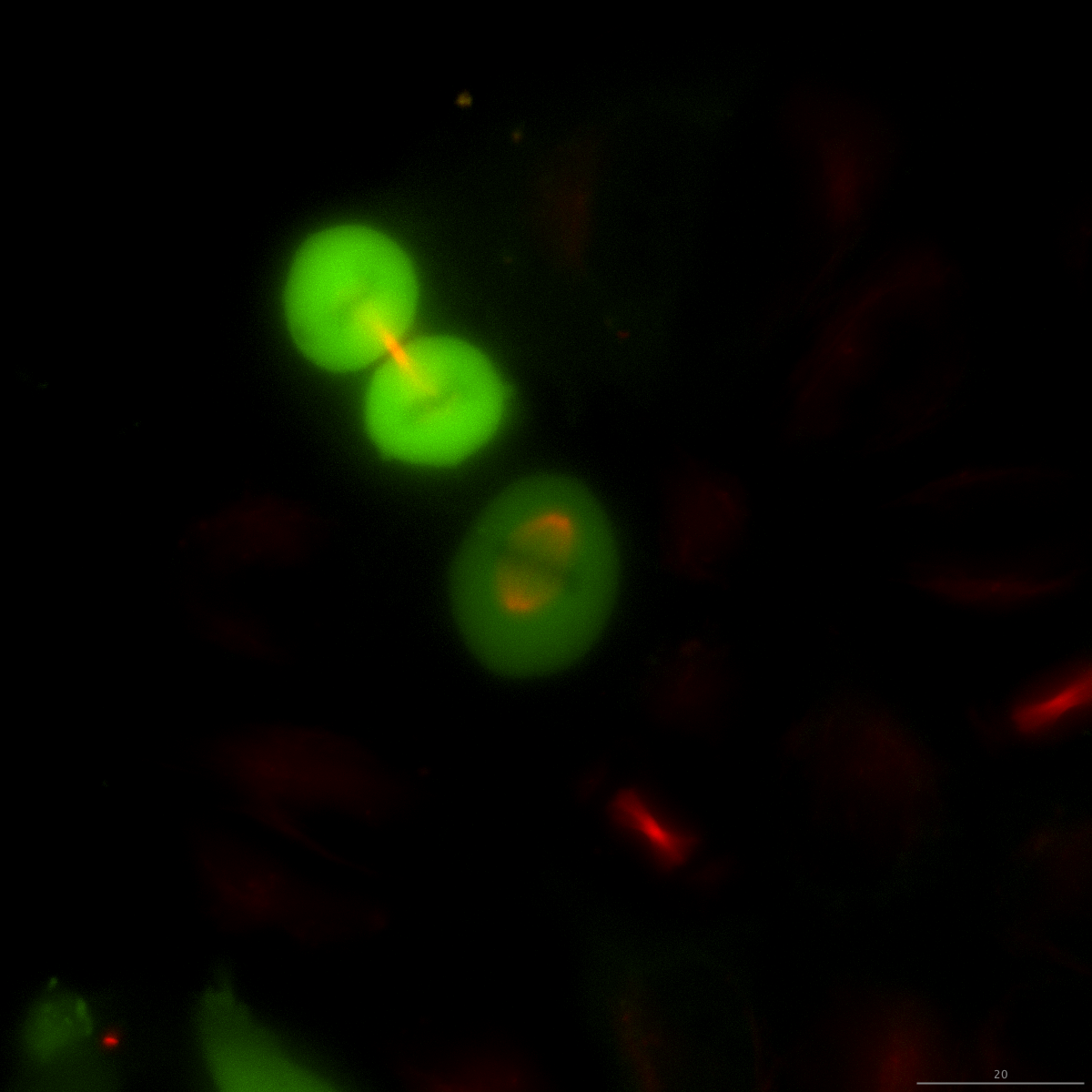

Supplement: Supplementary file 6 — Source Data for Figure 3 [file EMBJ-42-e113647-s011.zip › Figure 3/Figure 3b/CenpEcas9 GFP-Kif4a1133-1165 SiR005_projkif4ashortcomposite.png]

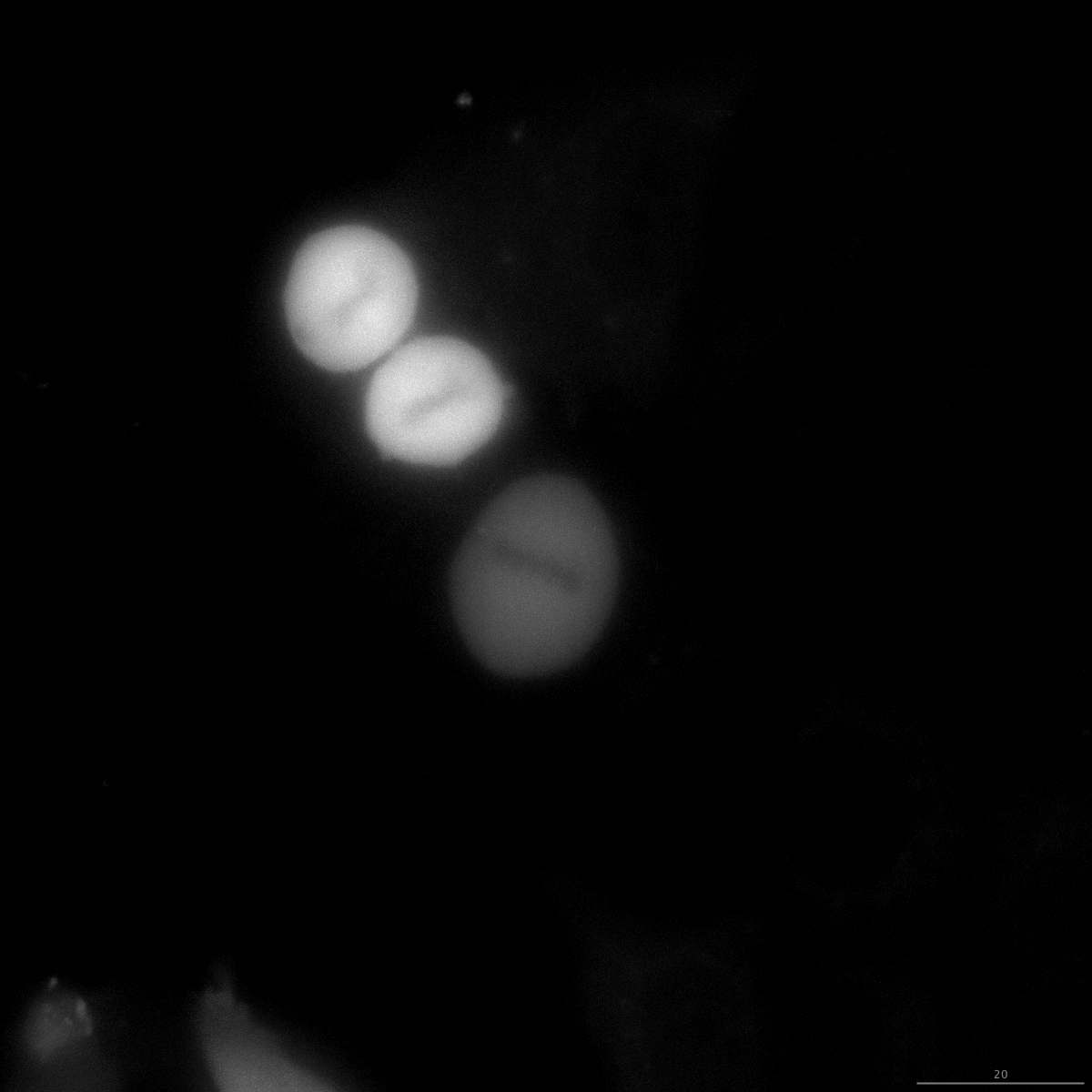

Supplement: Supplementary file 6 — Source Data for Figure 3 [file EMBJ-42-e113647-s011.zip › Figure 3/Figure 3b/CenpEcas9 GFP-Kif4a1133-1165 SiR005_GFP.png]

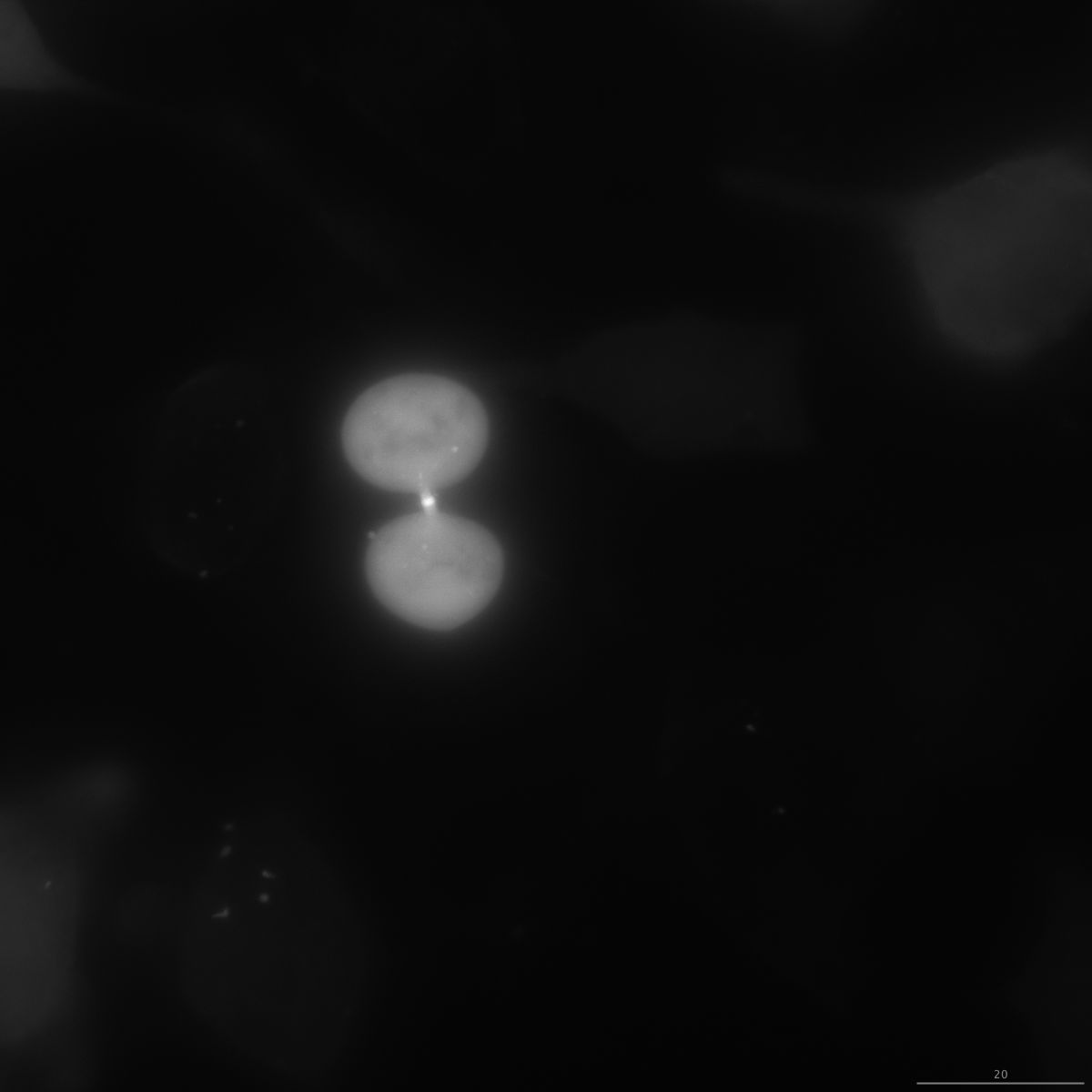

Supplement: Supplementary file 6 — Source Data for Figure 3 [file EMBJ-42-e113647-s011.zip › Figure 3/Figure 3b/ Figure 3b HeLAGFP-Kif4a1133-1232 SiR001_pgfp.png]

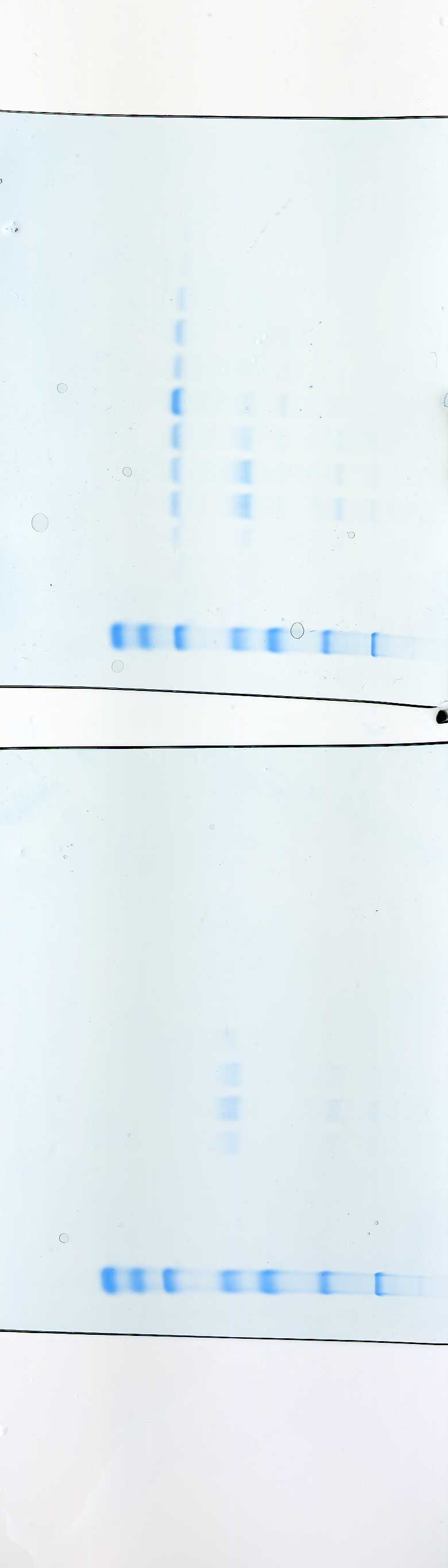

Supplement: Supplementary file 6 — Source Data for Figure 3 [file EMBJ-42-e113647-s011.zip › Figure 3/figure 3c/GFP-Kif4A 1133-1165 +PRC1 1-168 and PRC1 1-168 .jpg]

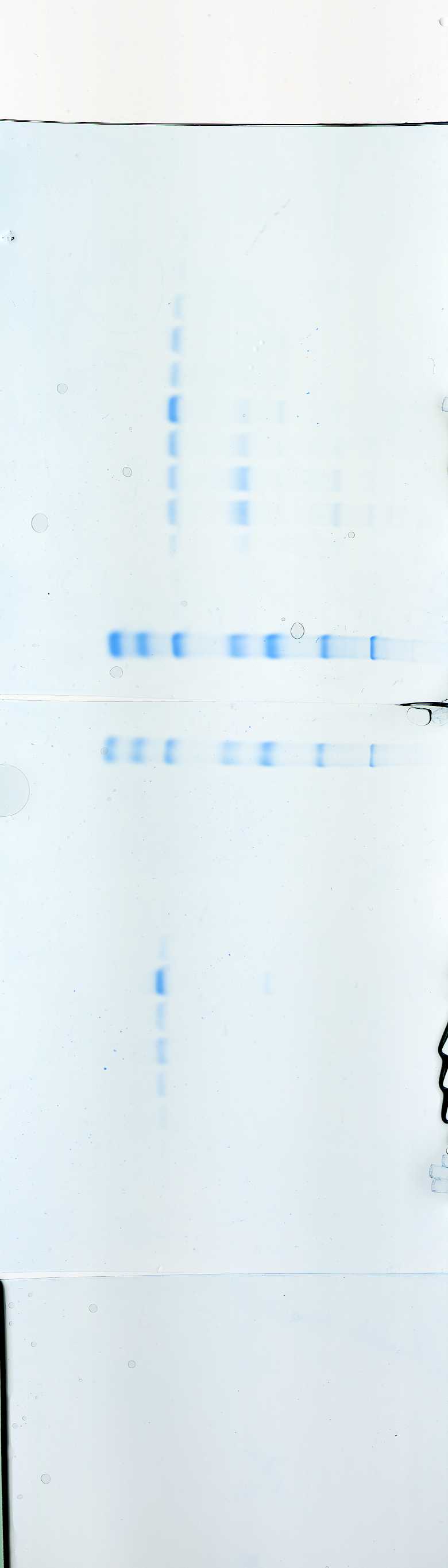

Supplement: Supplementary file 6 — Source Data for Figure 3 [file EMBJ-42-e113647-s011.zip › Figure 3/figure 3c/GFP-Kif4A 1133-1165.jpg]

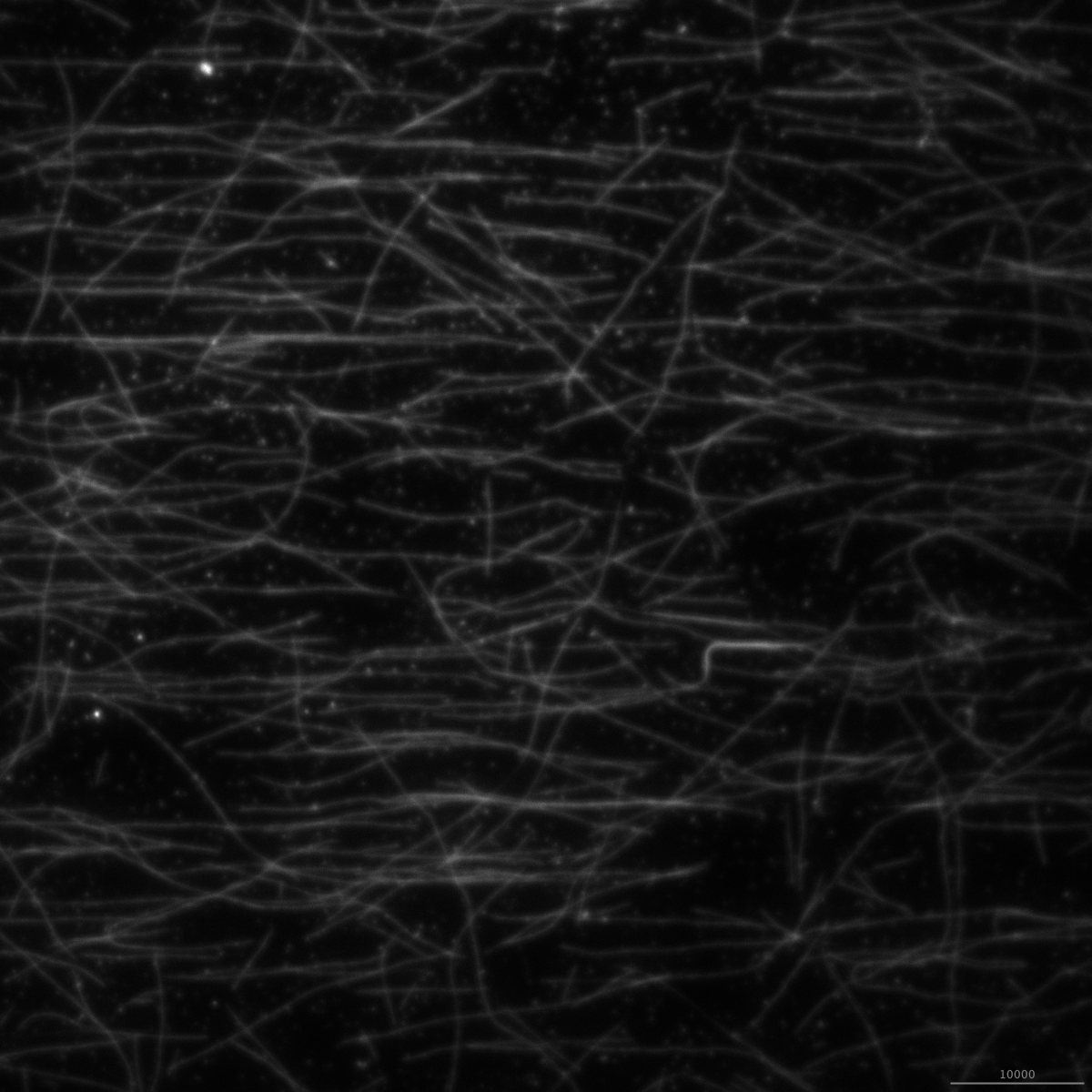

Supplement: Supplementary file 7 — Source Data for Figure 4 [file EMBJ-42-e113647-s008.zip › Figure 4/Fig 4C /Figure 4c_MT.tif]

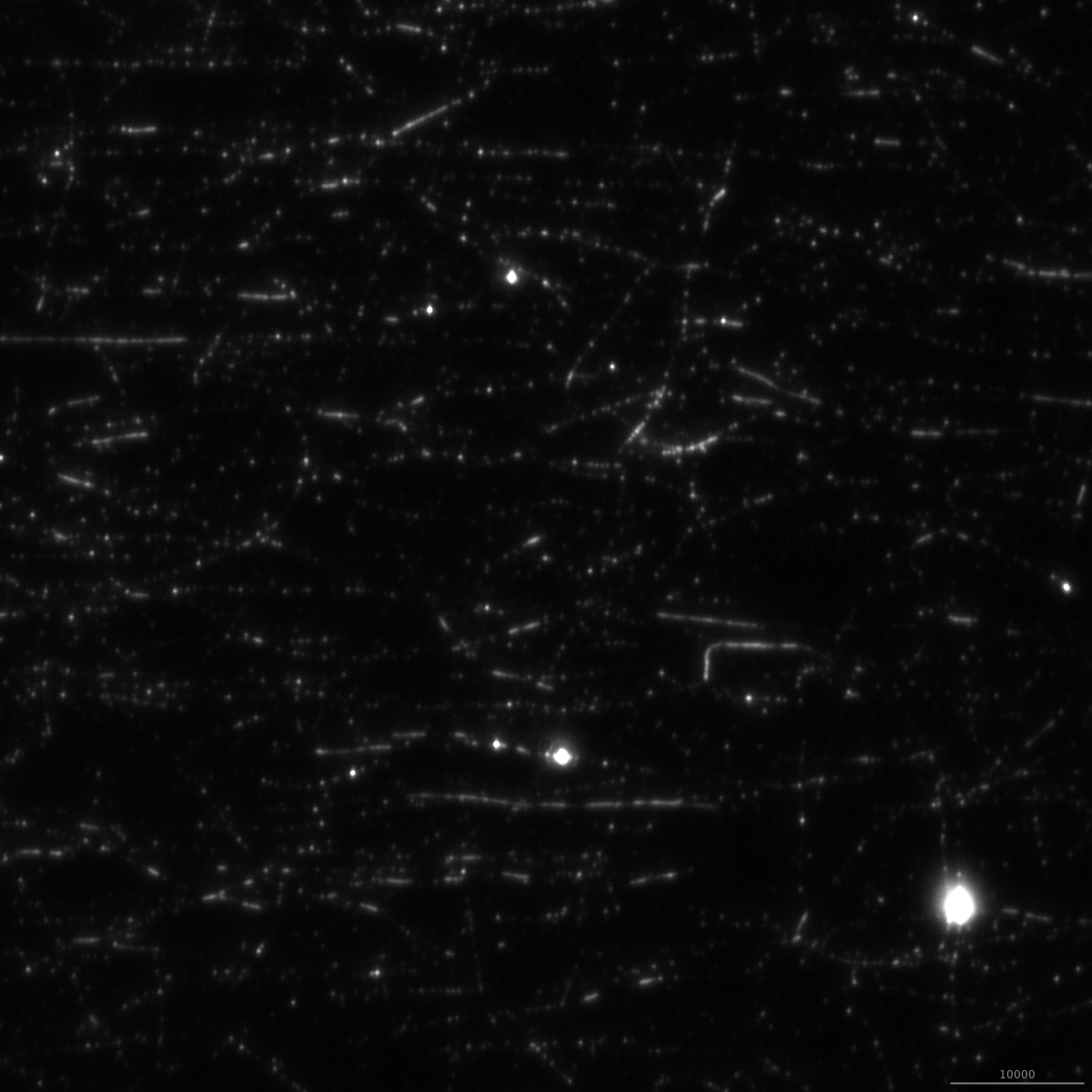

Supplement: Supplementary file 7 — Source Data for Figure 4 [file EMBJ-42-e113647-s008.zip › Figure 4/Fig 4C /Figure 4c_GFPCENPE.tif]

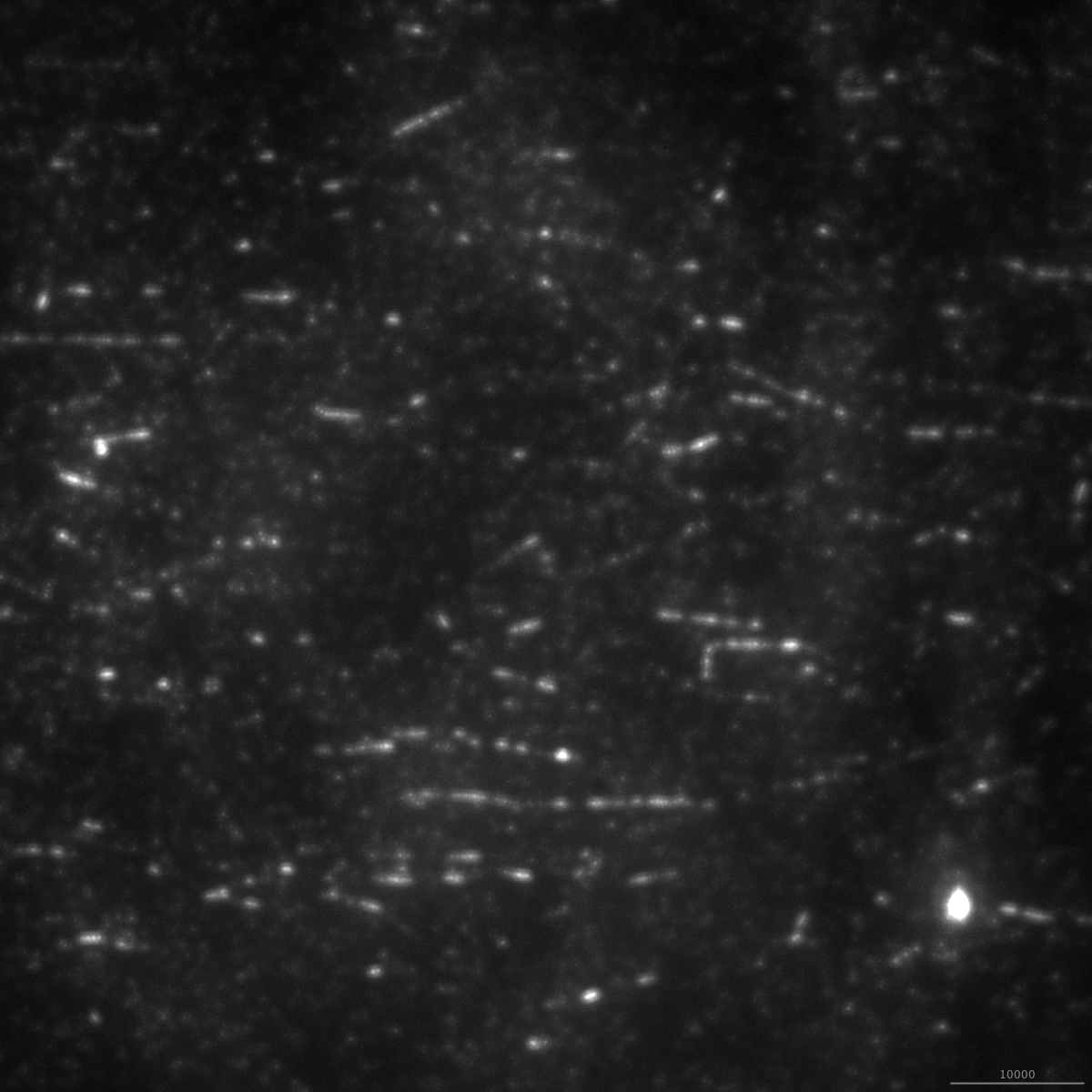

Supplement: Supplementary file 7 — Source Data for Figure 4 [file EMBJ-42-e113647-s008.zip › Figure 4/Fig 4C /figure 4c prc1.tif]

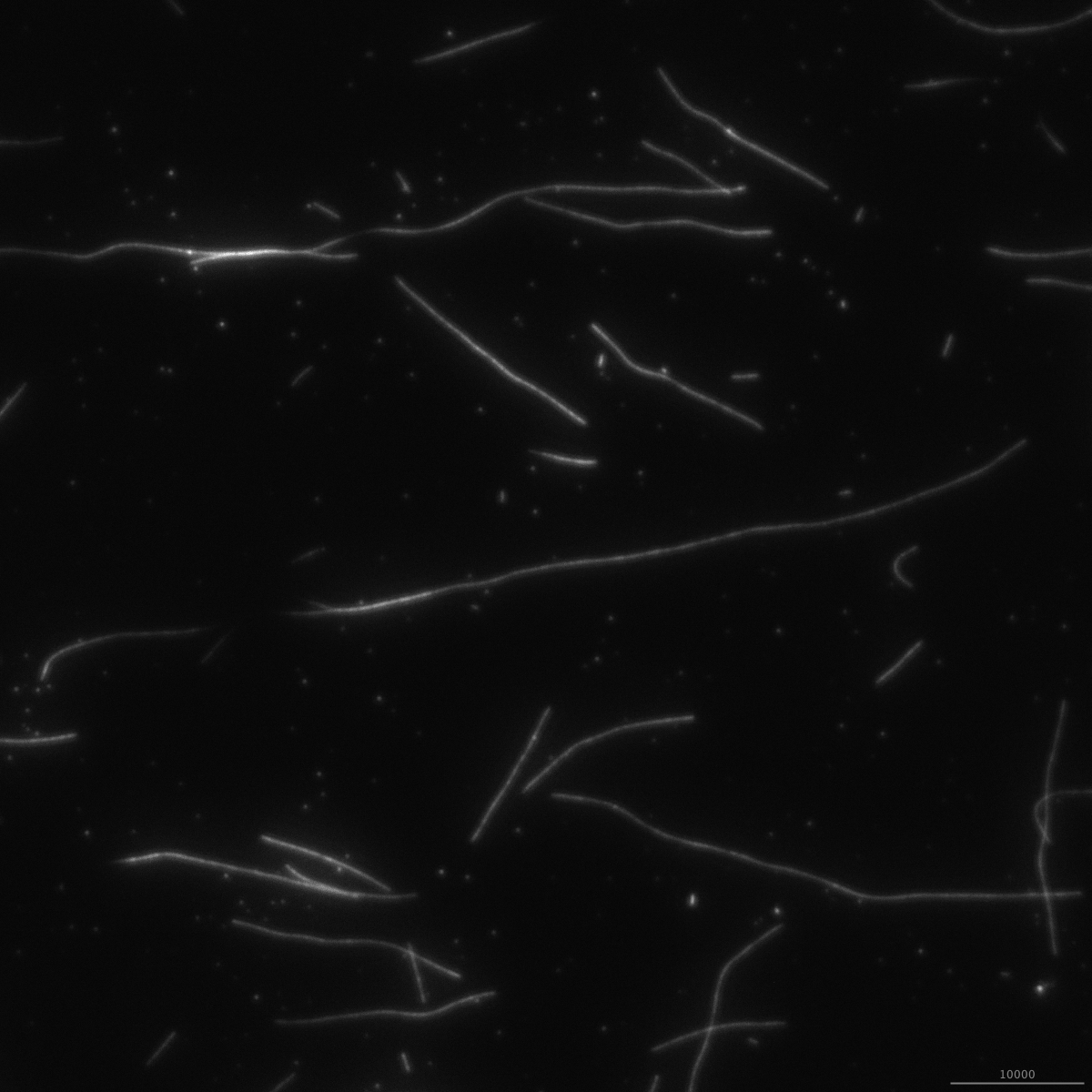

Supplement: Supplementary file 7 — Source Data for Figure 4 [file EMBJ-42-e113647-s008.zip › Figure 4/Fig 4A /Figure 4a_MT.tif]

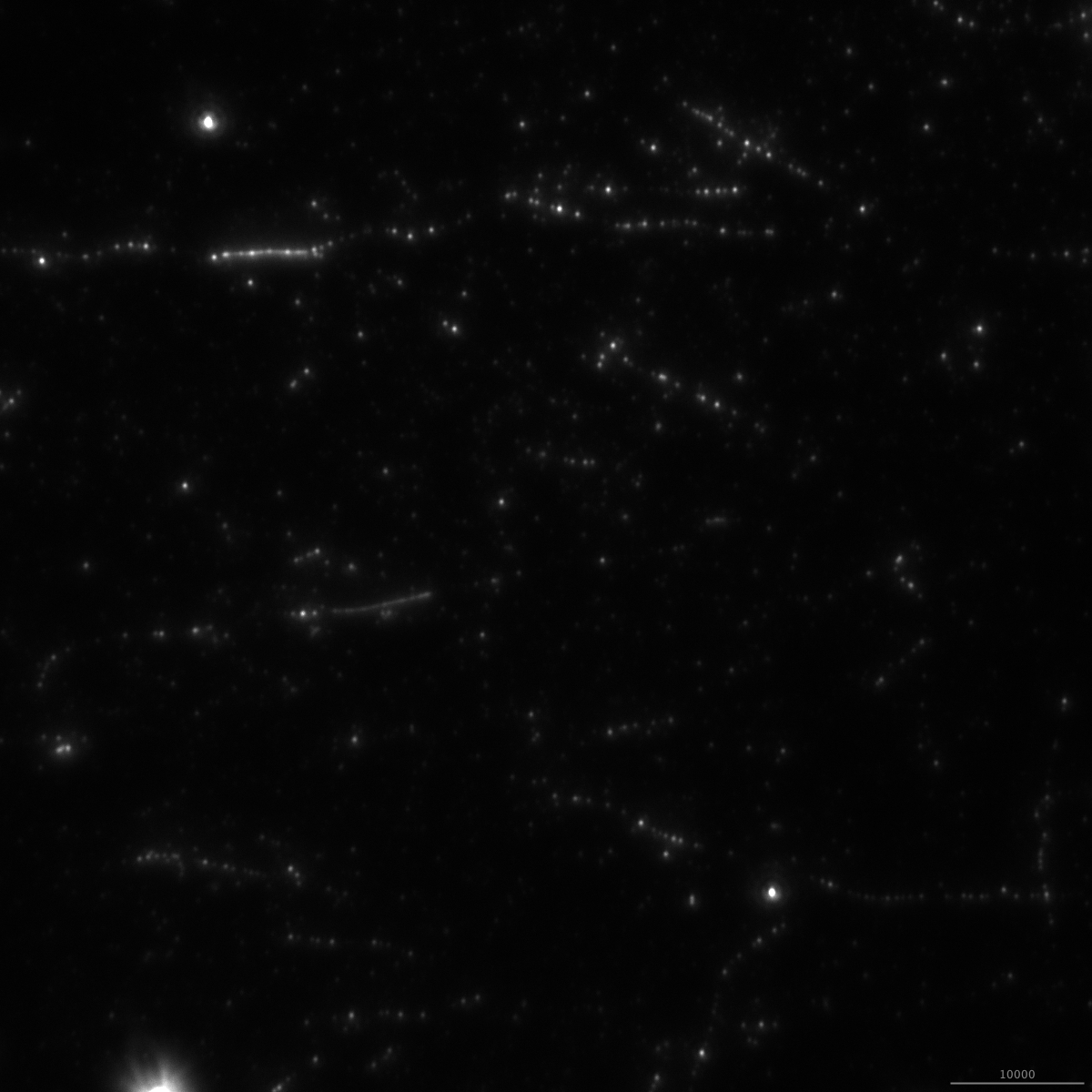

Supplement: Supplementary file 7 — Source Data for Figure 4 [file EMBJ-42-e113647-s008.zip › Figure 4/Fig 4A /Figure 4a_PRC1.tif]

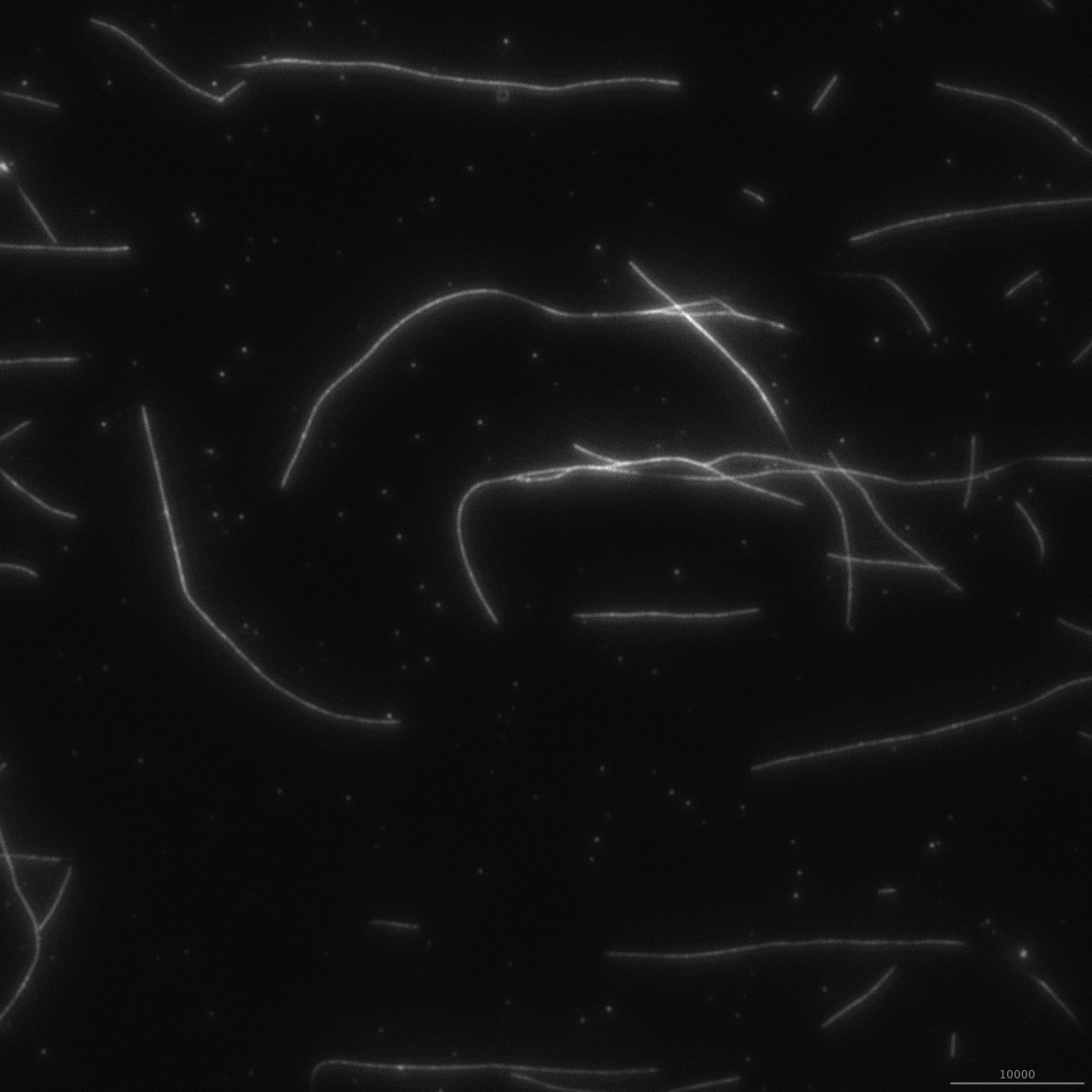

Supplement: Supplementary file 7 — Source Data for Figure 4 [file EMBJ-42-e113647-s008.zip › Figure 4/Fig 4B /Figure 4B-MT.tif]

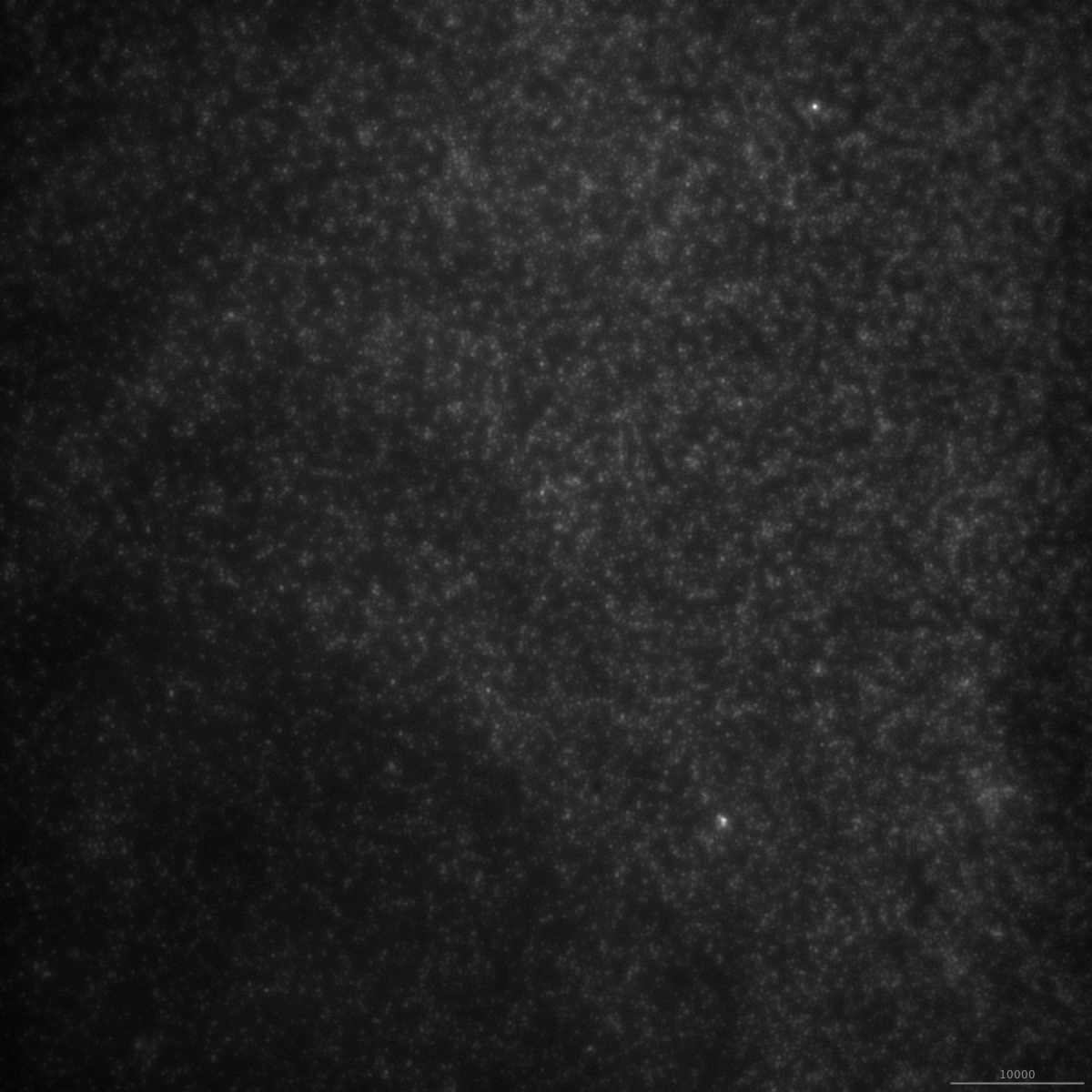

Supplement: Supplementary file 7 — Source Data for Figure 4 [file EMBJ-42-e113647-s008.zip › Figure 4/Fig 4B /Figure 4B_GFP CENPE.tif]

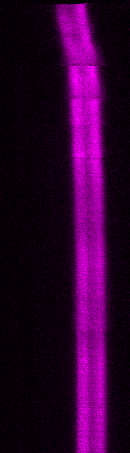

Supplement: Supplementary file 7 — Source Data for Figure 4 [file EMBJ-42-e113647-s008.zip › Figure 4/Fig 4F kymo/8.94 um 453 frames rho C1-0910-0623.tif]

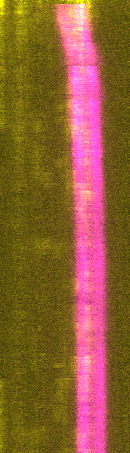

Supplement: Supplementary file 7 — Source Data for Figure 4 [file EMBJ-42-e113647-s008.zip › Figure 4/Fig 4F kymo/8.94 um 453 frames merge C1-0910-0623.tif]

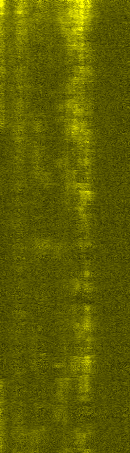

Supplement: Supplementary file 7 — Source Data for Figure 4 [file EMBJ-42-e113647-s008.zip › Figure 4/Fig 4F kymo/8.94 um 453 frames 647 C1-0910-0623.tif]

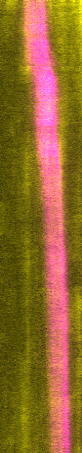

Supplement: Supplementary file 7 — Source Data for Figure 4 [file EMBJ-42-e113647-s008.zip › Figure 4/Fig 4G kymo/5.64 um 453 frames merge C1-0910-0623.tif]

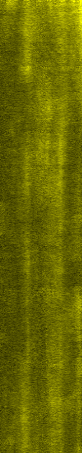

Supplement: Supplementary file 7 — Source Data for Figure 4 [file EMBJ-42-e113647-s008.zip › Figure 4/Fig 4G kymo/5.64 um 453 frames 647 C1-0910-0623.tif]

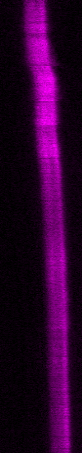

Supplement: Supplementary file 7 — Source Data for Figure 4 [file EMBJ-42-e113647-s008.zip › Figure 4/Fig 4G kymo/5.64 um 453 frames rho C1-0910-0623.tif]

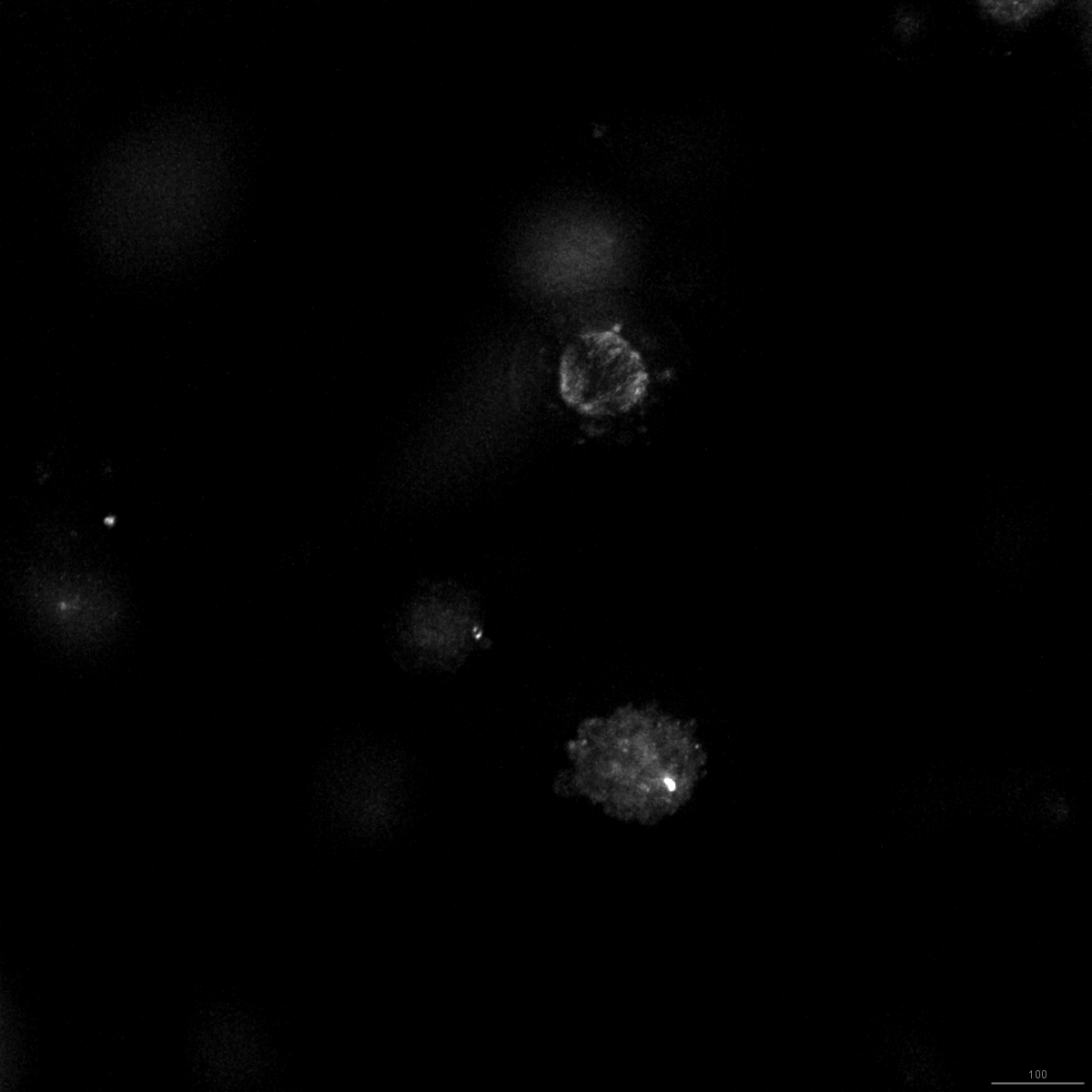

Supplement: Supplementary file 8 — Source Data for Figure 5 [file EMBJ-42-e113647-s003.zip › Figure 5/Figure 5D/GST-2SD/SiR Cy5.tif]

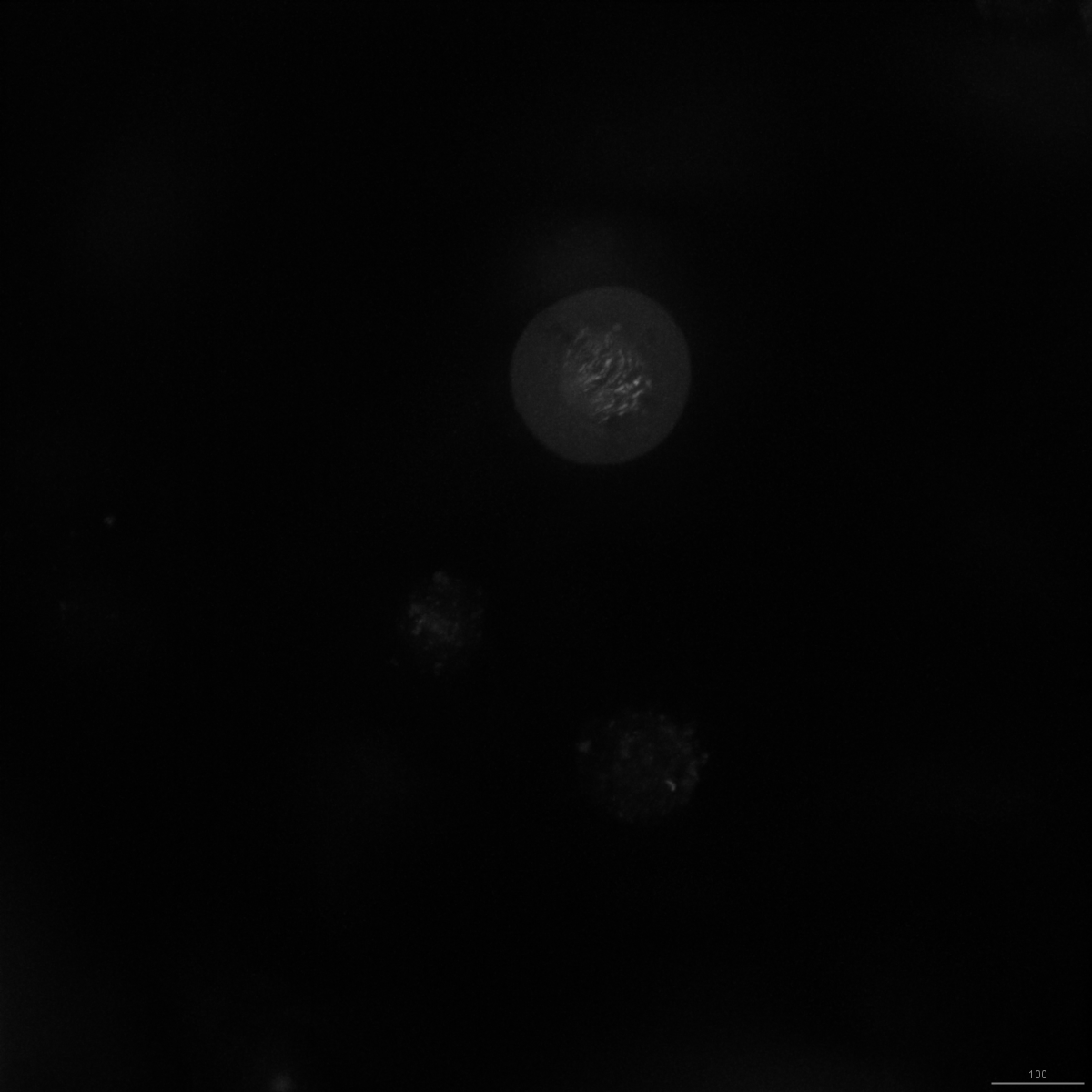

Supplement: Supplementary file 8 — Source Data for Figure 5 [file EMBJ-42-e113647-s003.zip › Figure 5/Figure 5D/GST-2SD/GFP.tif]

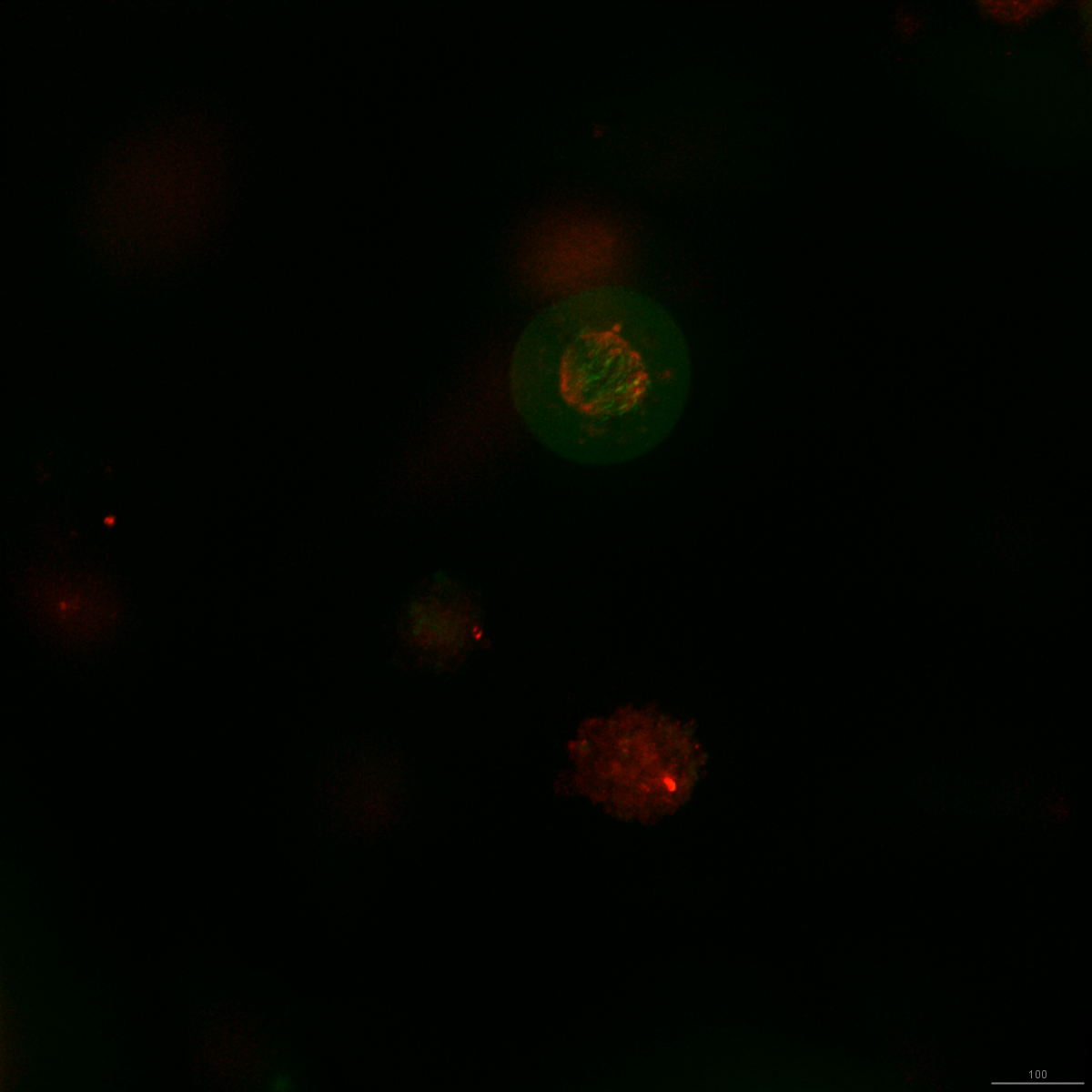

Supplement: Supplementary file 8 — Source Data for Figure 5 [file EMBJ-42-e113647-s003.zip › Figure 5/Figure 5D/GST-2SD/merge.tif]

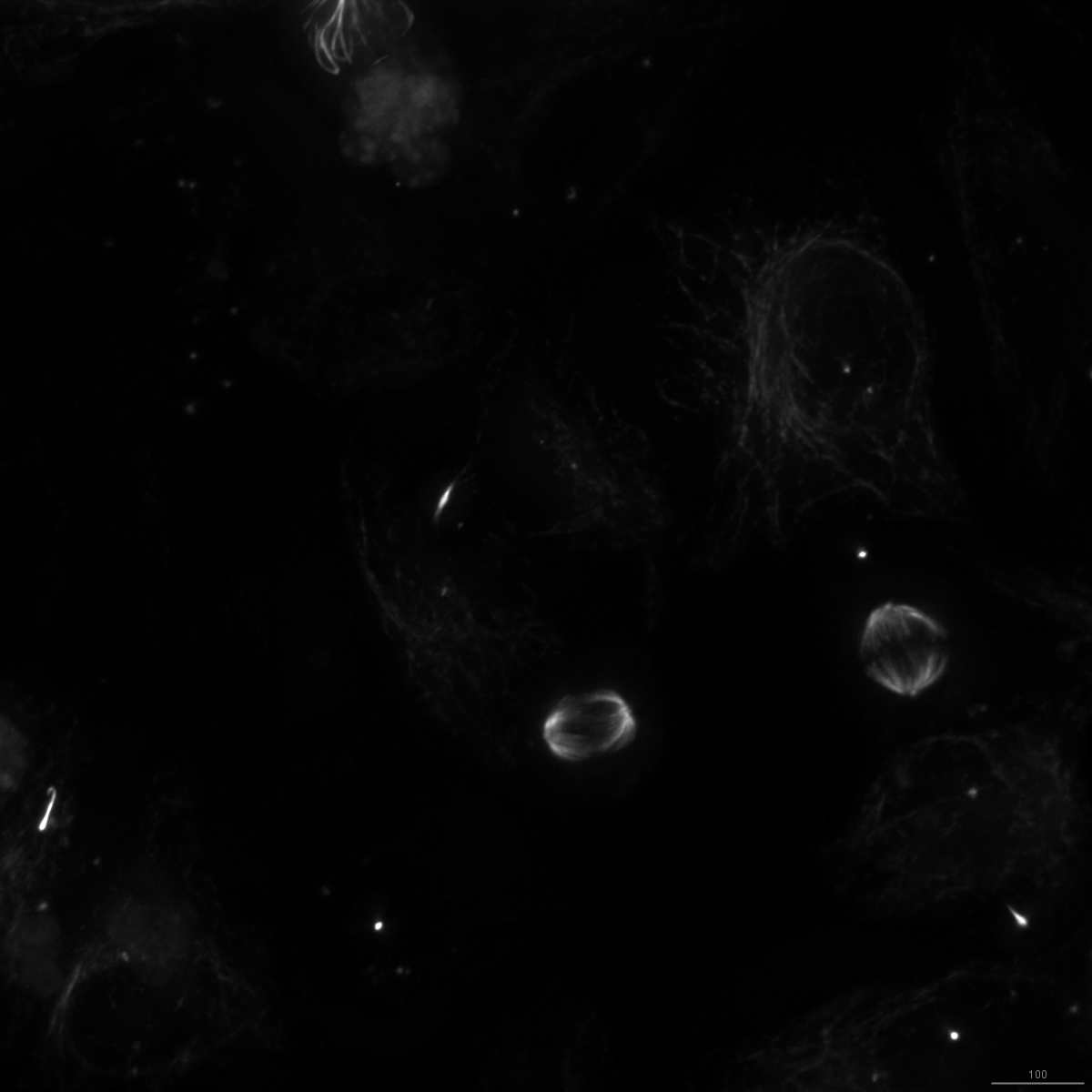

Supplement: Supplementary file 8 — Source Data for Figure 5 [file EMBJ-42-e113647-s003.zip › Figure 5/Figure 5D/GST-6SD/SiR Cy5.tif]

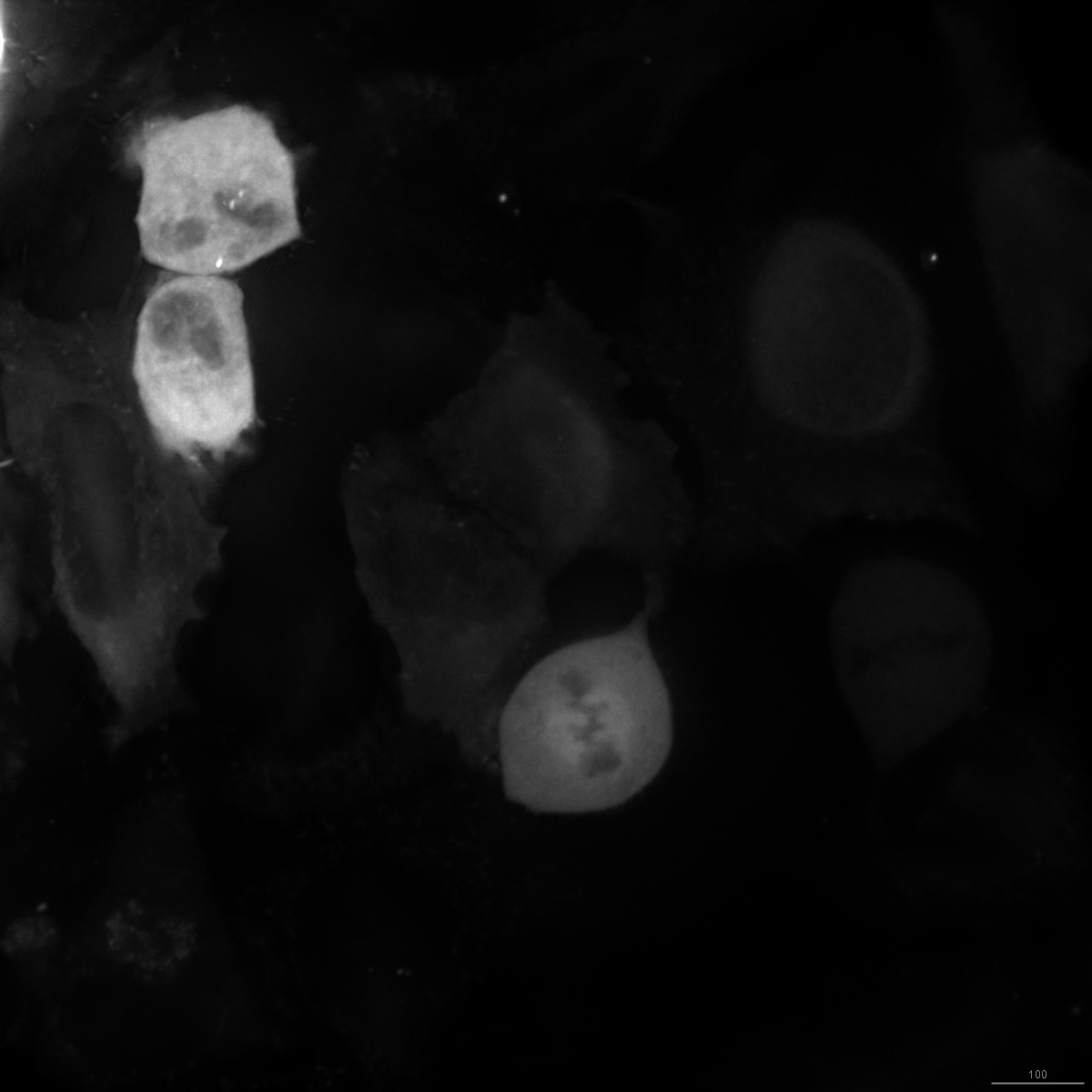

Supplement: Supplementary file 8 — Source Data for Figure 5 [file EMBJ-42-e113647-s003.zip › Figure 5/Figure 5D/GST-6SD/GFP.tif]

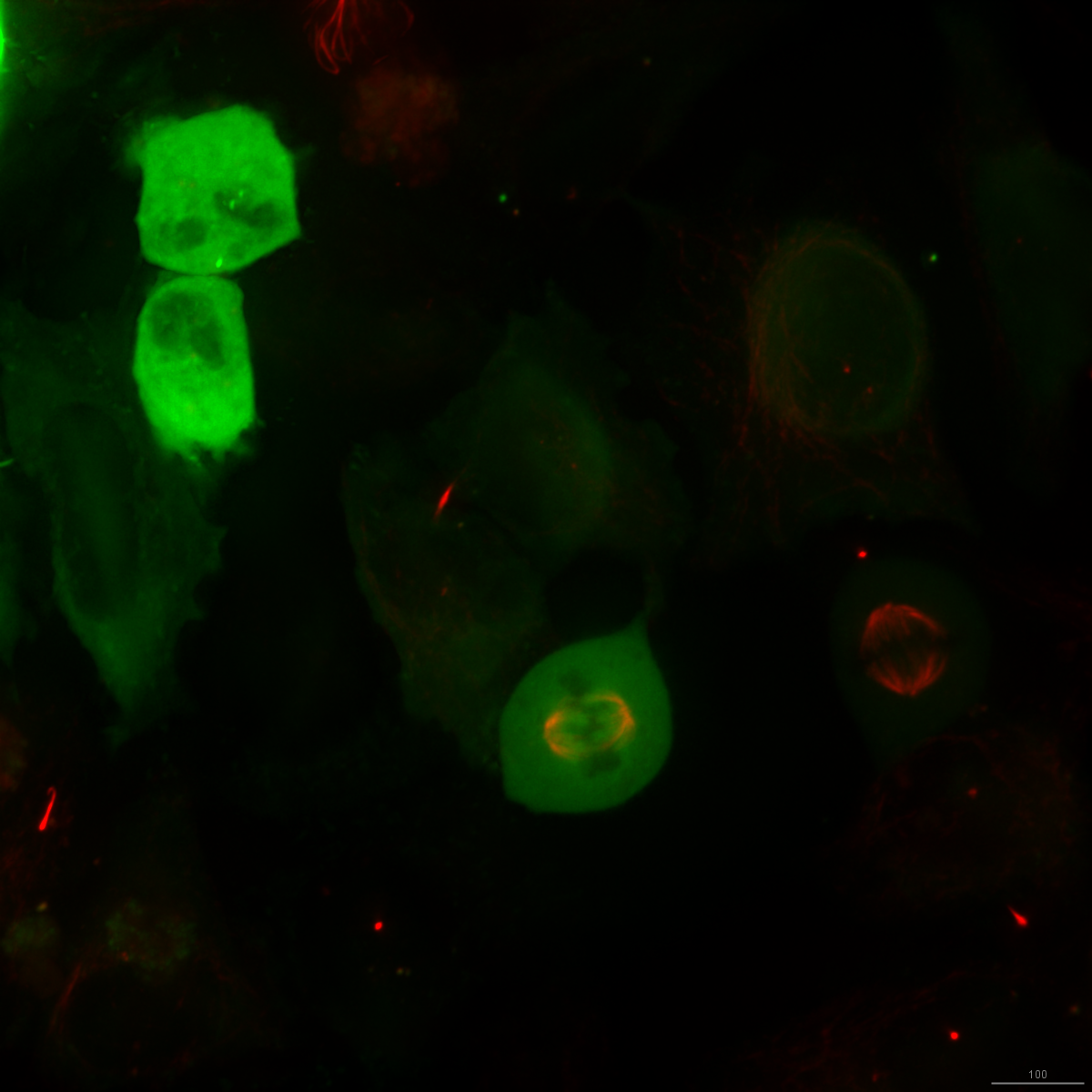

Supplement: Supplementary file 8 — Source Data for Figure 5 [file EMBJ-42-e113647-s003.zip › Figure 5/Figure 5D/GST-6SD/merge.tif]

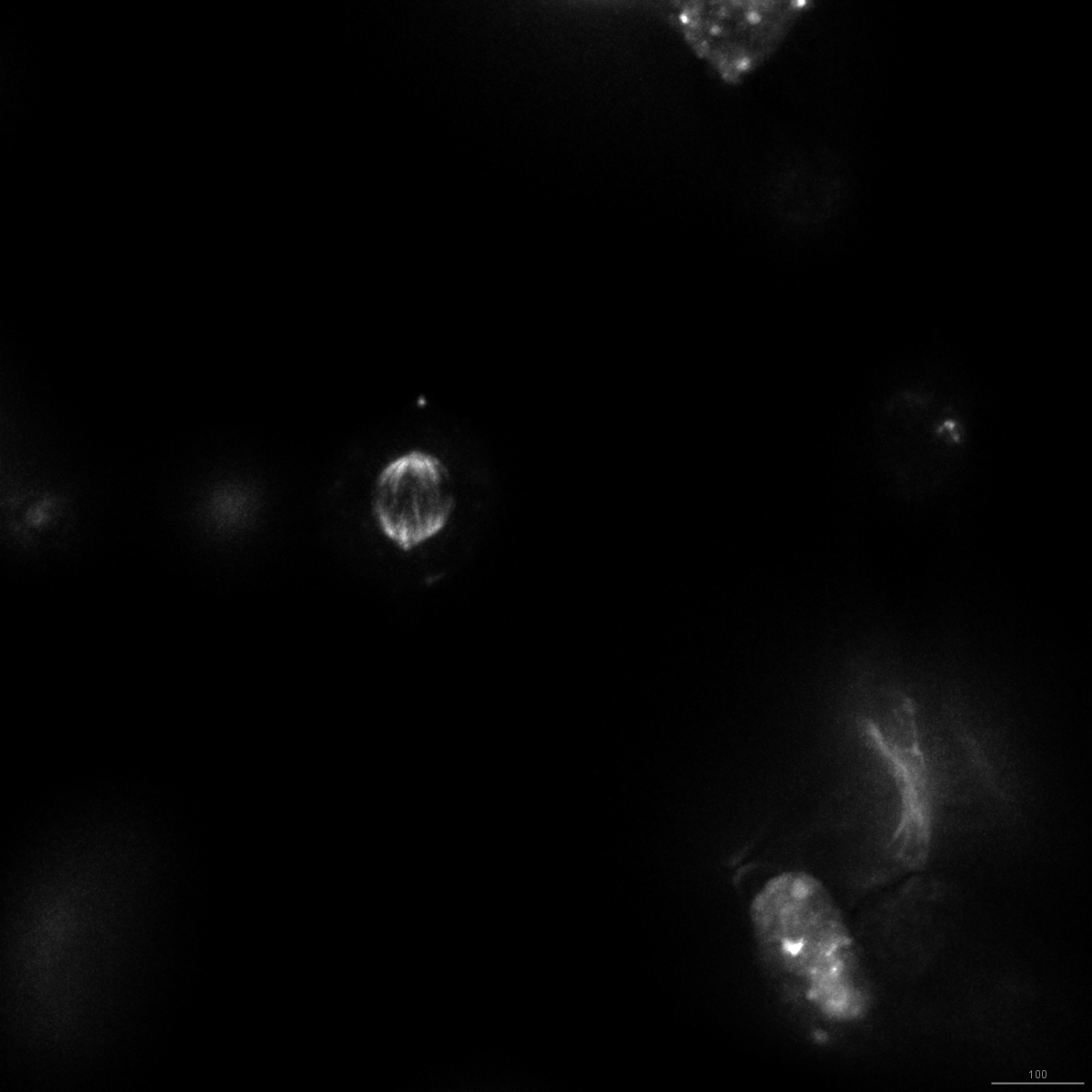

Supplement: Supplementary file 8 — Source Data for Figure 5 [file EMBJ-42-e113647-s003.zip › Figure 5/Figure 5D/GST-wt/SiR Cy5.tif]

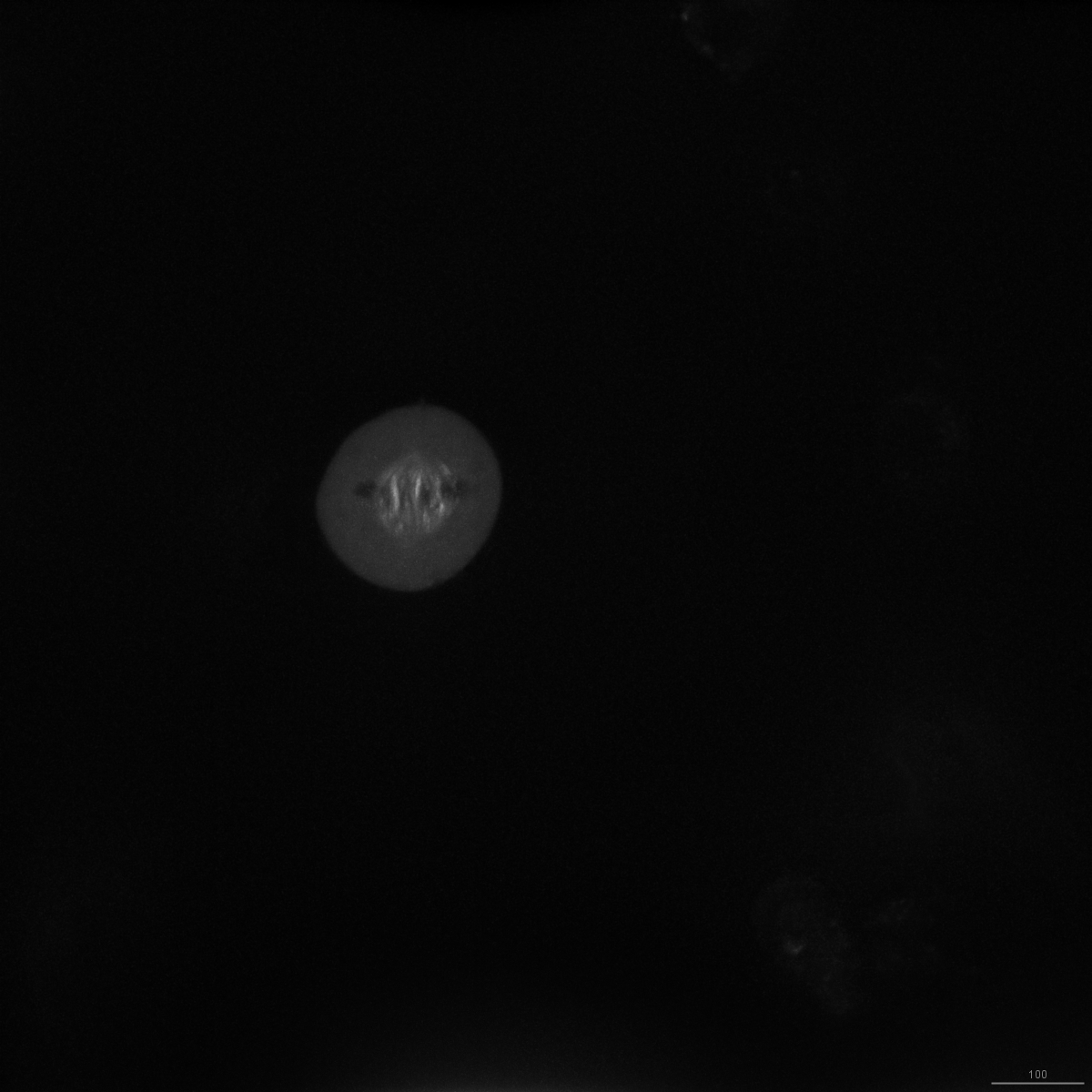

Supplement: Supplementary file 8 — Source Data for Figure 5 [file EMBJ-42-e113647-s003.zip › Figure 5/Figure 5D/GST-wt/GFP.tif]

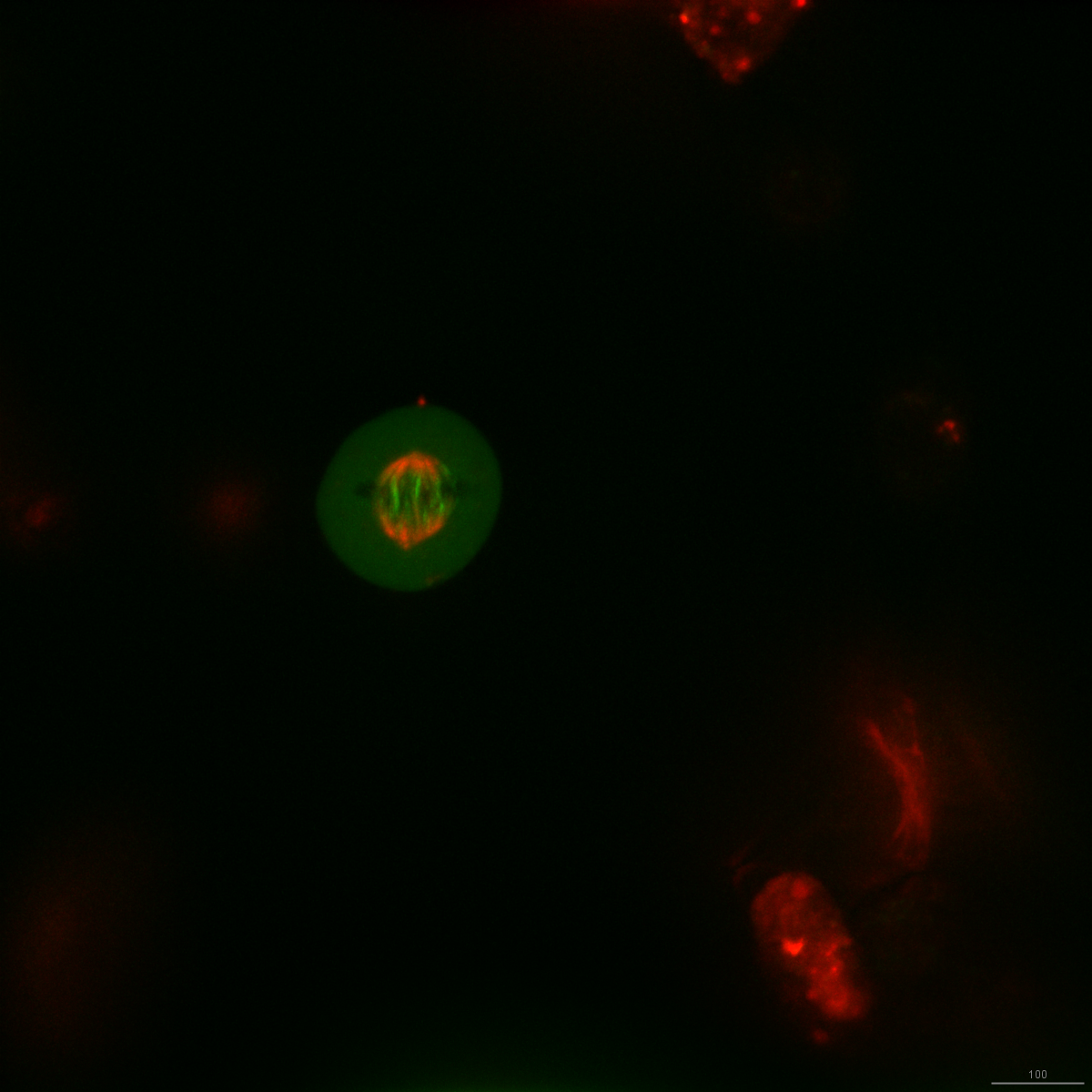

Supplement: Supplementary file 8 — Source Data for Figure 5 [file EMBJ-42-e113647-s003.zip › Figure 5/Figure 5D/GST-wt/merge.tif]

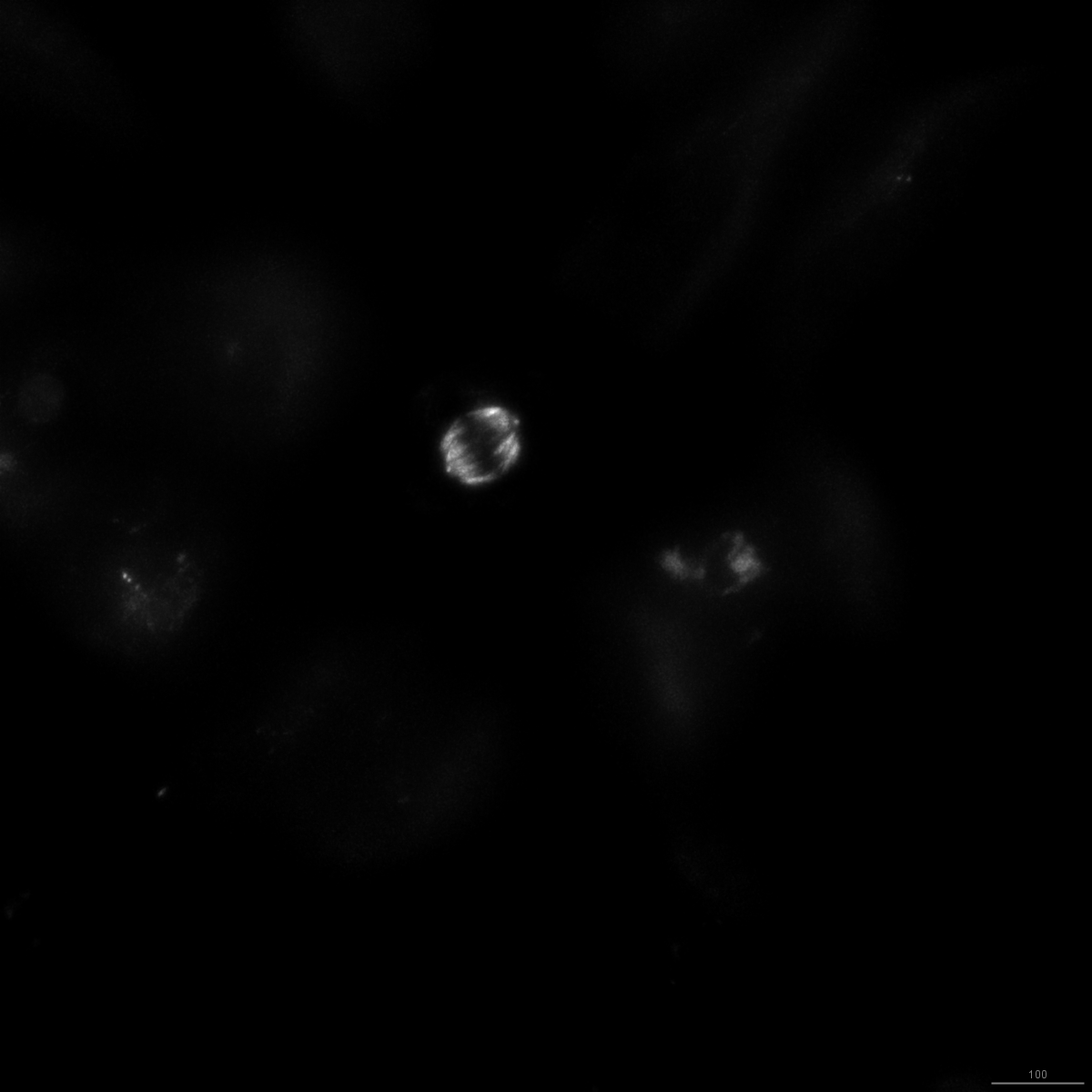

Supplement: Supplementary file 8 — Source Data for Figure 5 [file EMBJ-42-e113647-s003.zip › Figure 5/Figure 5D/2SD/SiR Cy5.tif]

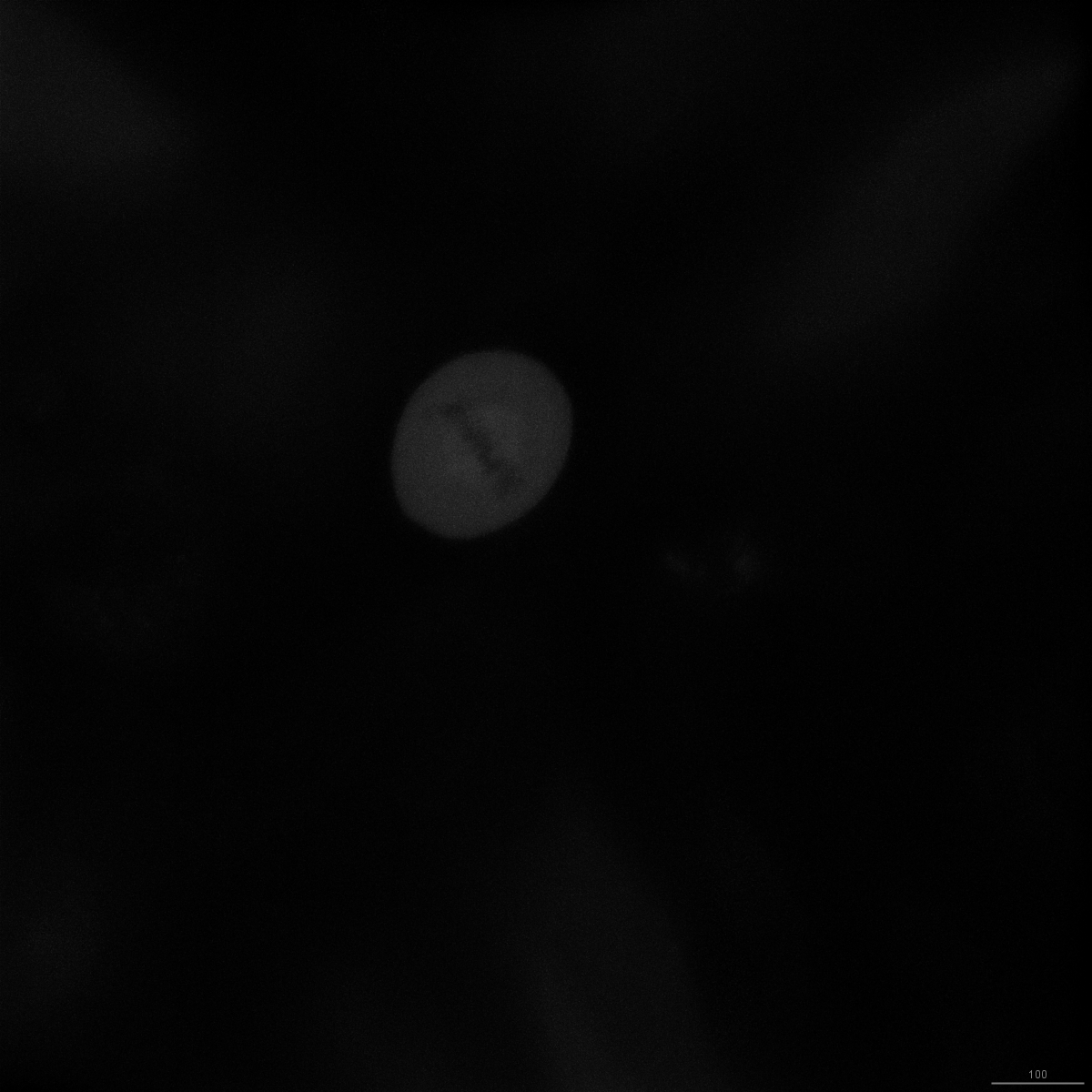

Supplement: Supplementary file 8 — Source Data for Figure 5 [file EMBJ-42-e113647-s003.zip › Figure 5/Figure 5D/2SD/GFP.tif]

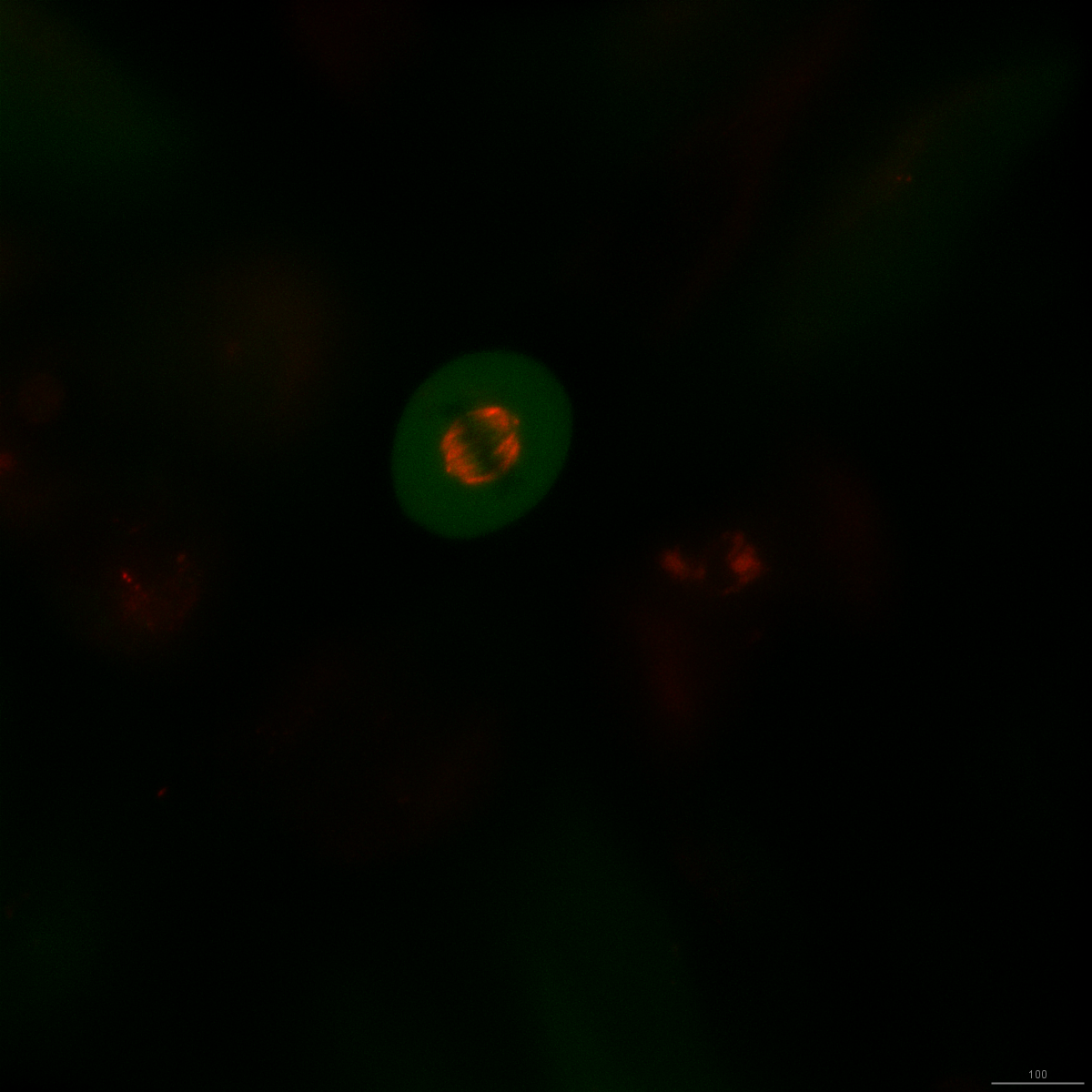

Supplement: Supplementary file 8 — Source Data for Figure 5 [file EMBJ-42-e113647-s003.zip › Figure 5/Figure 5D/2SD/merge.tif]

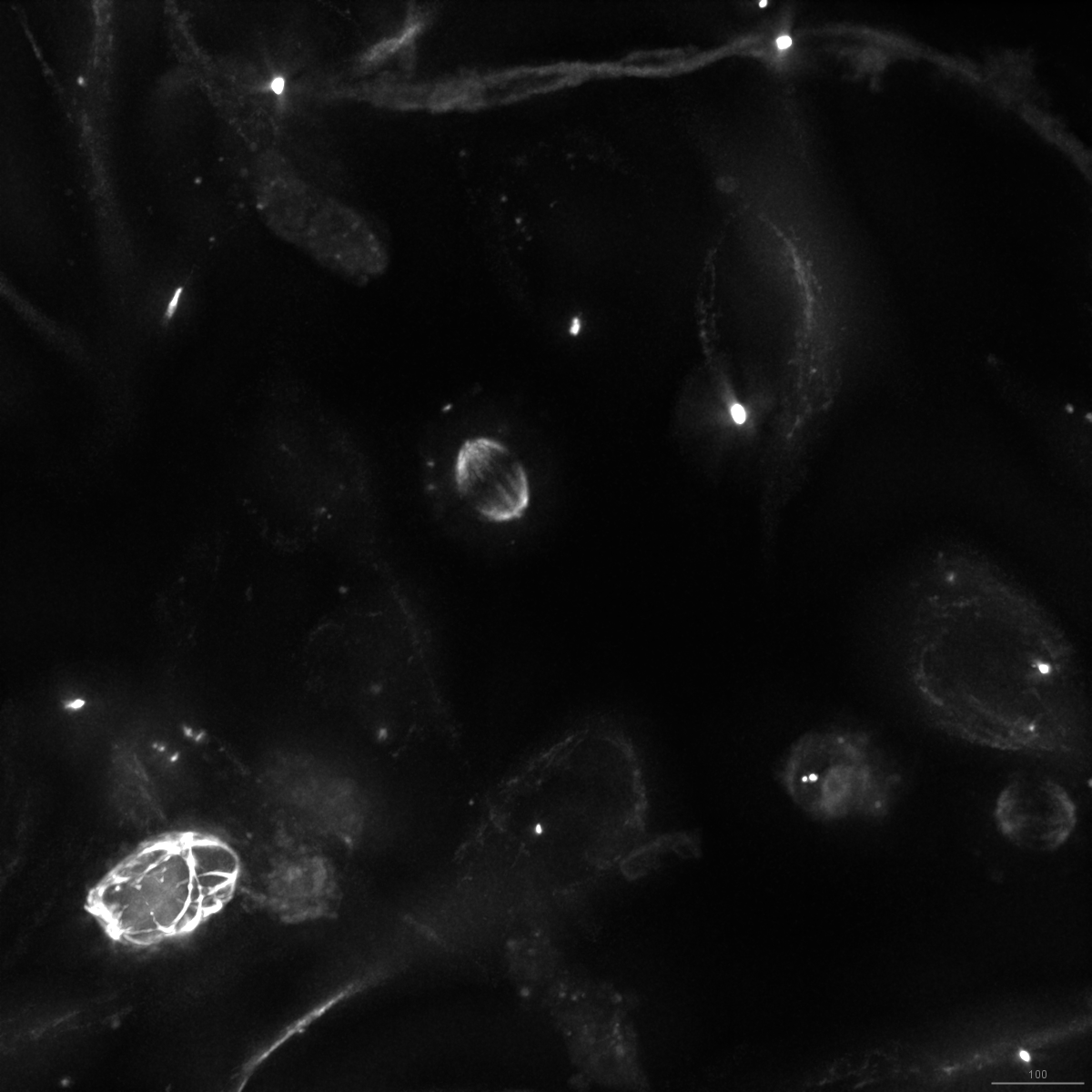

Supplement: Supplementary file 8 — Source Data for Figure 5 [file EMBJ-42-e113647-s003.zip › Figure 5/Figure 5D/GST-6SA/SiR Cy5.tif]

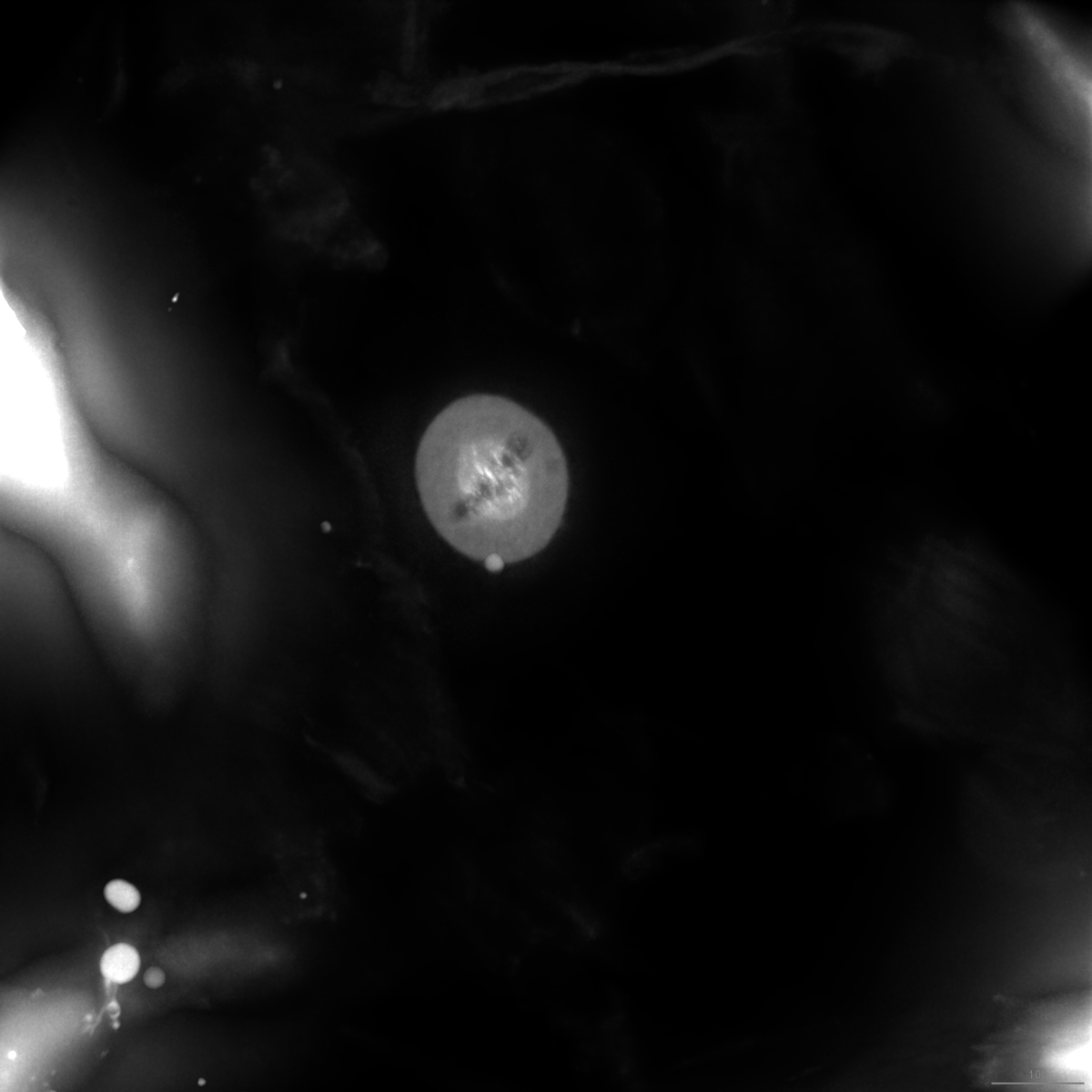

Supplement: Supplementary file 8 — Source Data for Figure 5 [file EMBJ-42-e113647-s003.zip › Figure 5/Figure 5D/GST-6SA/GFP.tif]

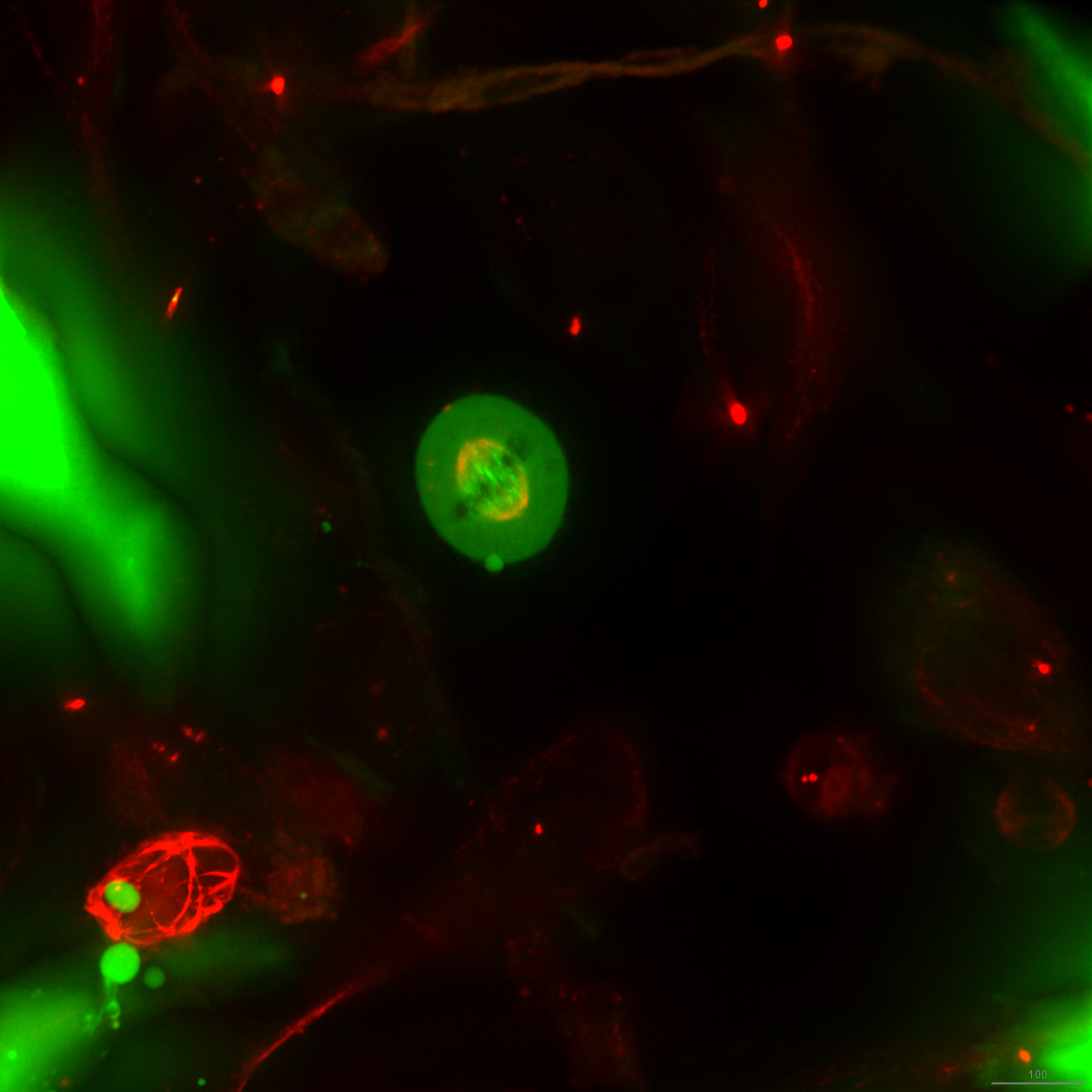

Supplement: Supplementary file 8 — Source Data for Figure 5 [file EMBJ-42-e113647-s003.zip › Figure 5/Figure 5D/GST-6SA/merge.tif]

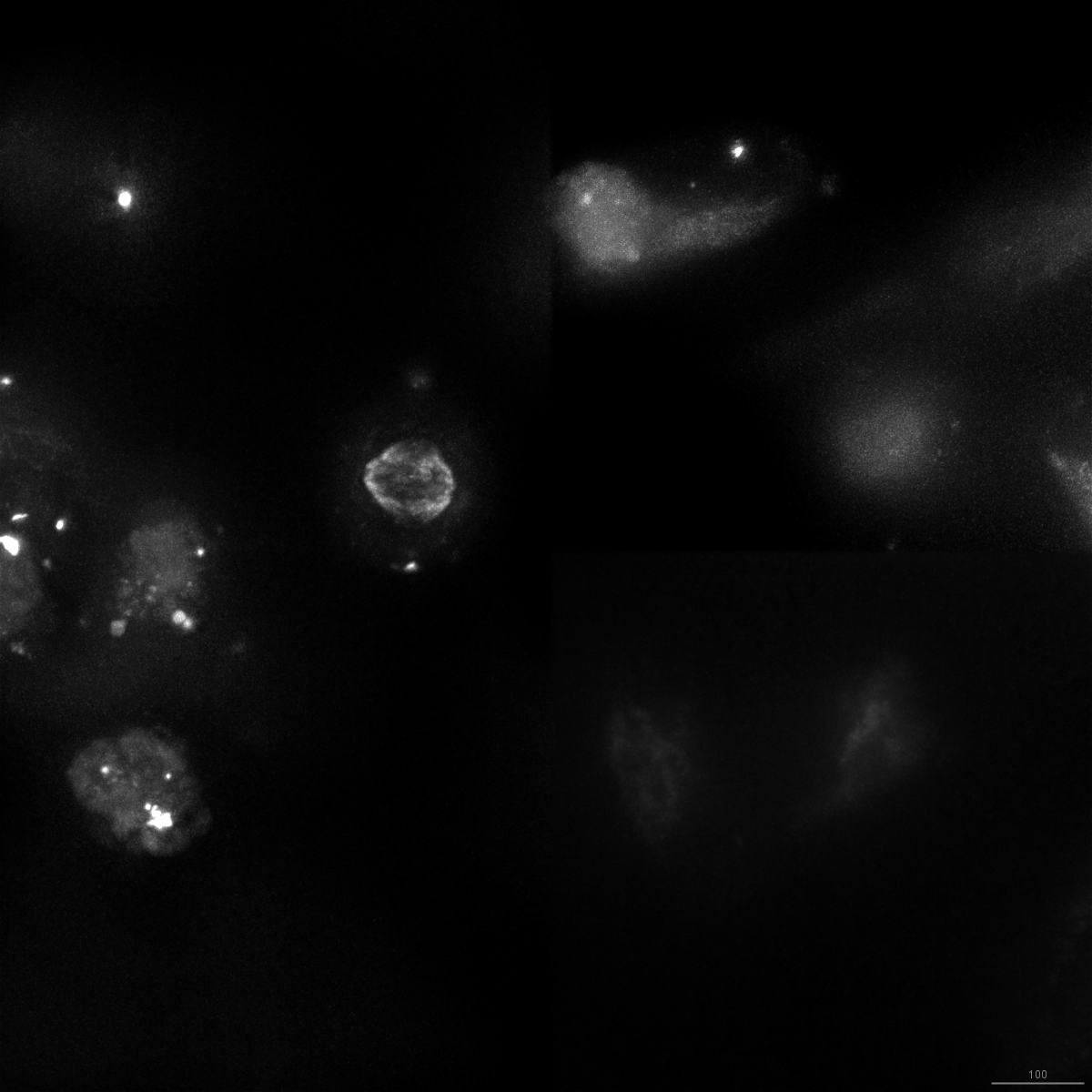

Supplement: Supplementary file 8 — Source Data for Figure 5 [file EMBJ-42-e113647-s003.zip › Figure 5/Figure 5D/GST-2SA/SiR Cy5.tif]

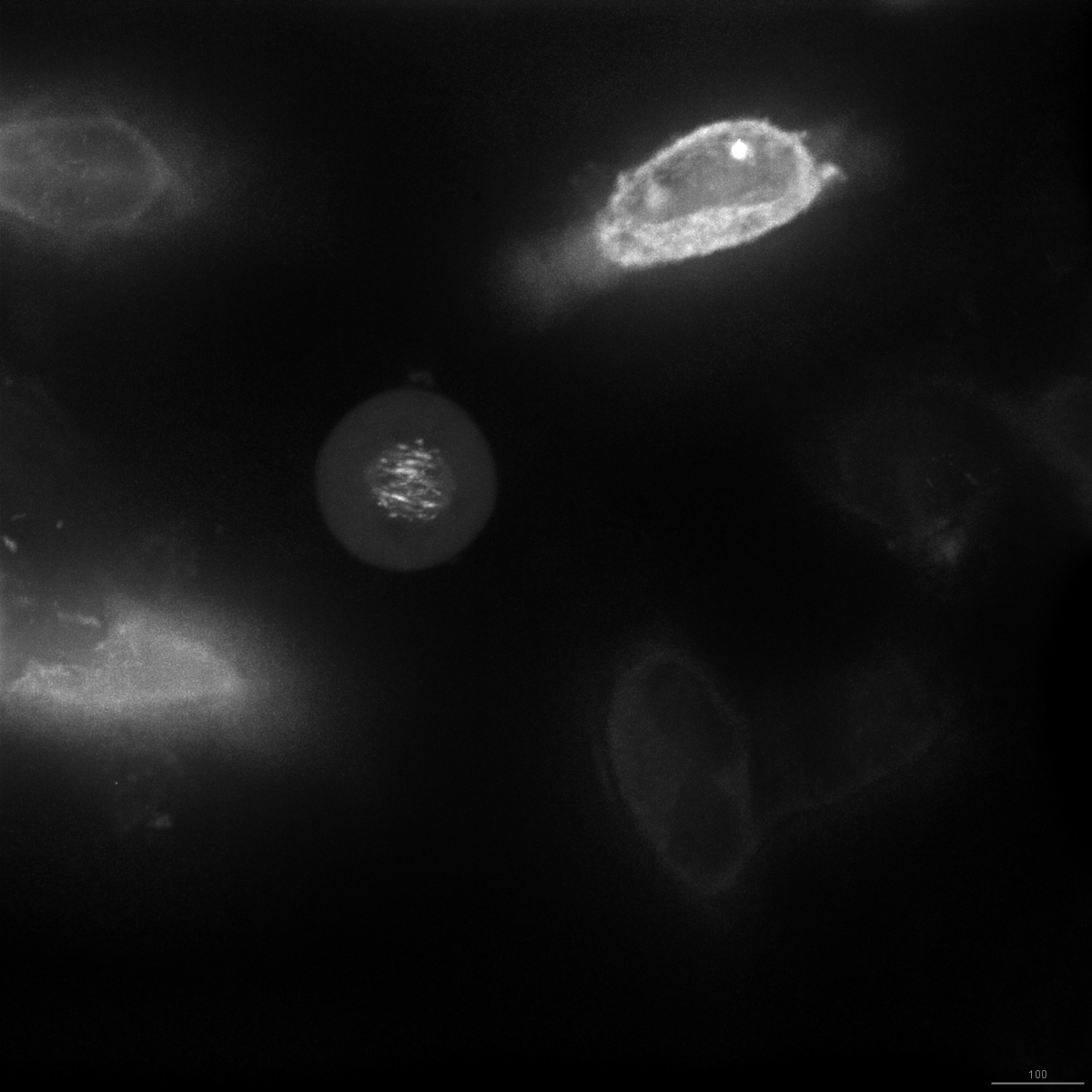

Supplement: Supplementary file 8 — Source Data for Figure 5 [file EMBJ-42-e113647-s003.zip › Figure 5/Figure 5D/GST-2SA/GFP.tif]

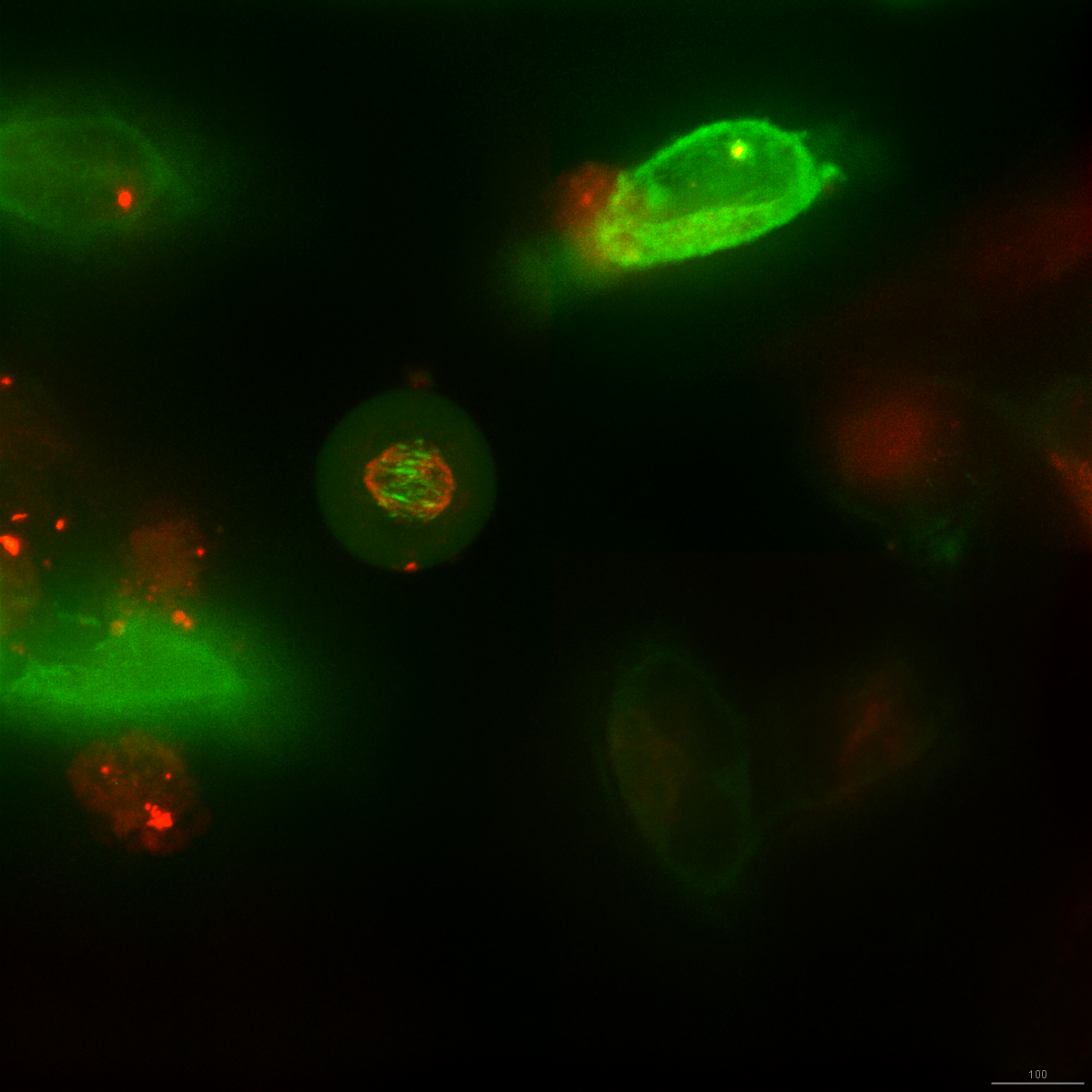

Supplement: Supplementary file 8 — Source Data for Figure 5 [file EMBJ-42-e113647-s003.zip › Figure 5/Figure 5D/GST-2SA/merge.tif]

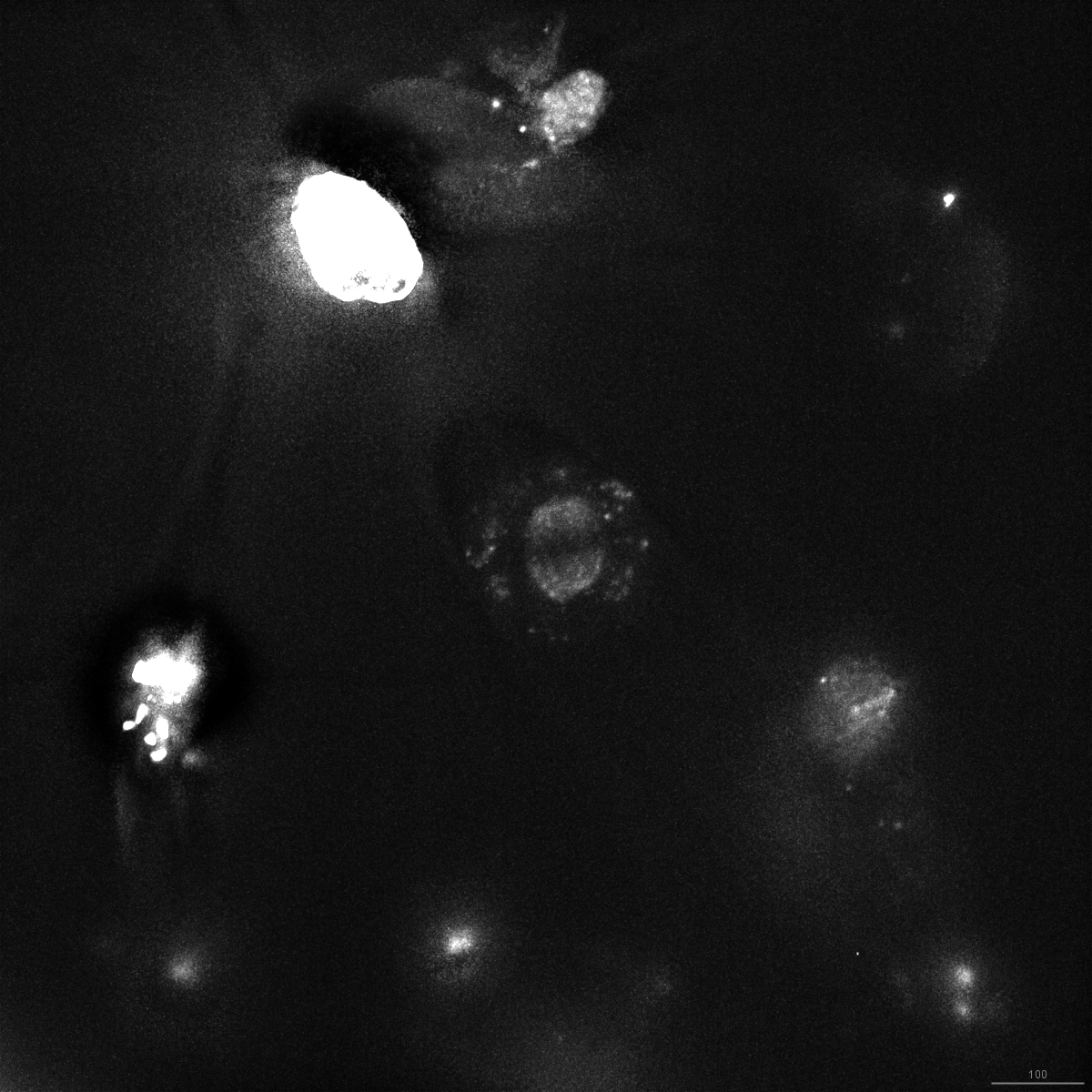

Supplement: Supplementary file 8 — Source Data for Figure 5 [file EMBJ-42-e113647-s003.zip › Figure 5/Figure 5D/2SA/SiR Cy5.tif]

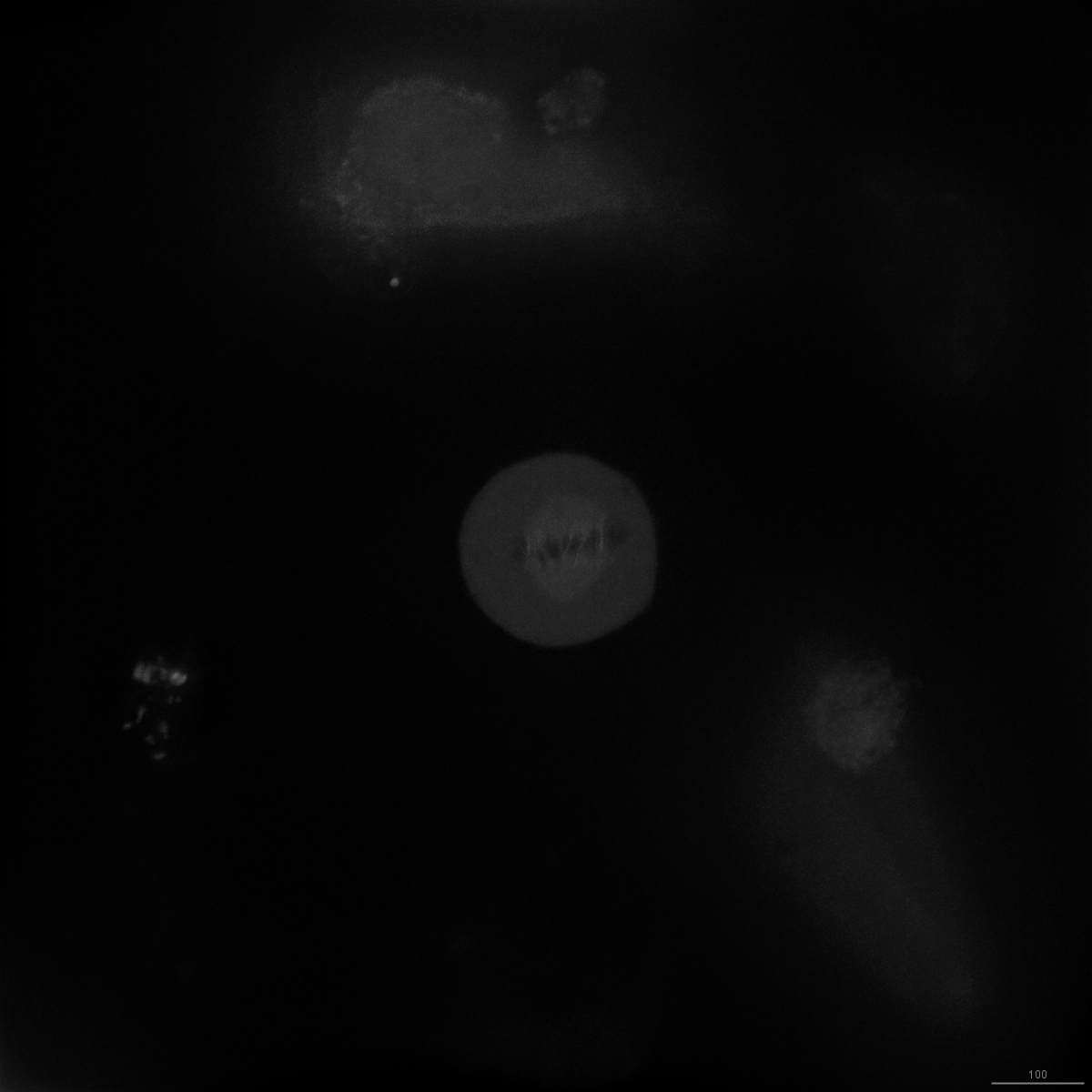

Supplement: Supplementary file 8 — Source Data for Figure 5 [file EMBJ-42-e113647-s003.zip › Figure 5/Figure 5D/2SA/GFP.tif]

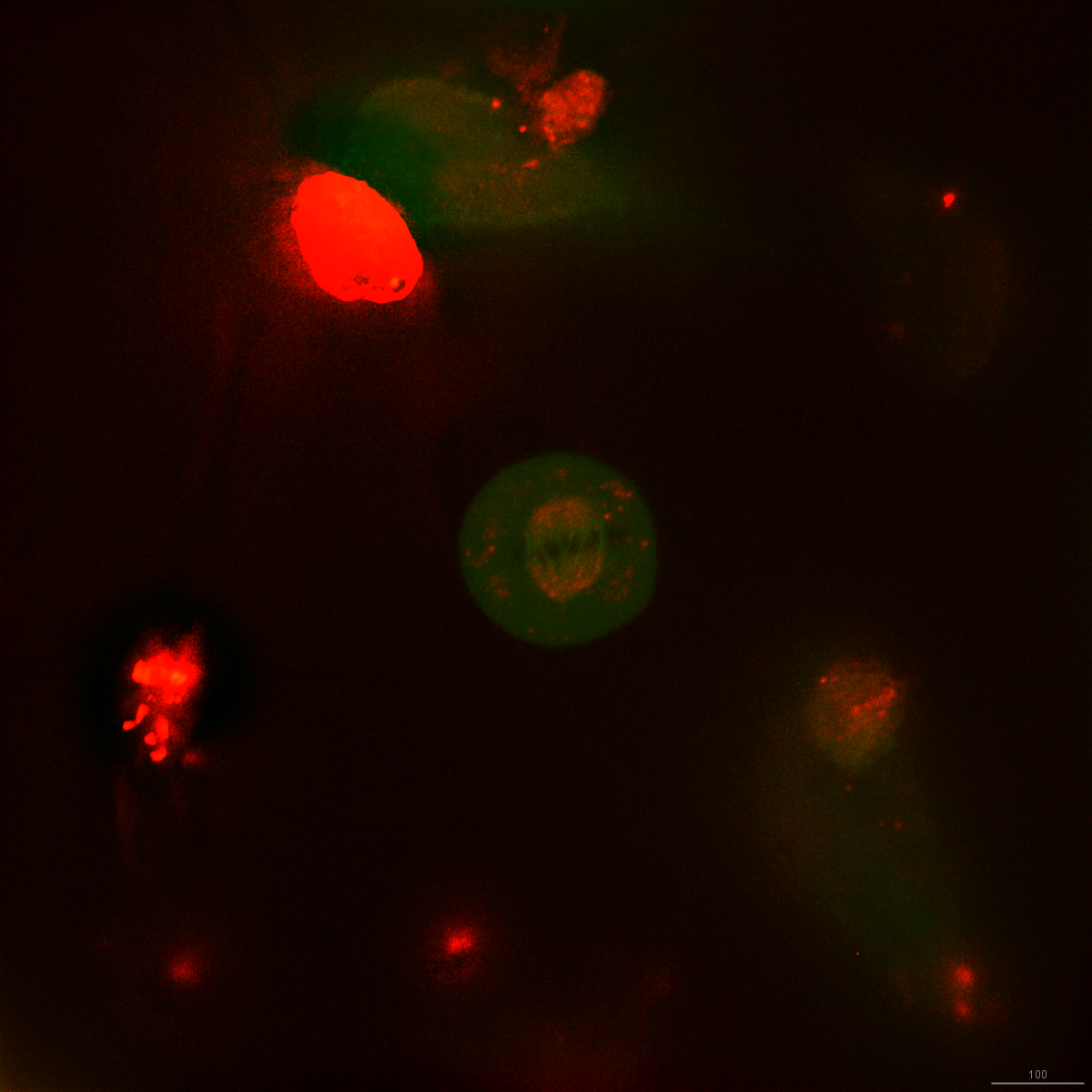

Supplement: Supplementary file 8 — Source Data for Figure 5 [file EMBJ-42-e113647-s003.zip › Figure 5/Figure 5D/2SA/merge.tif]

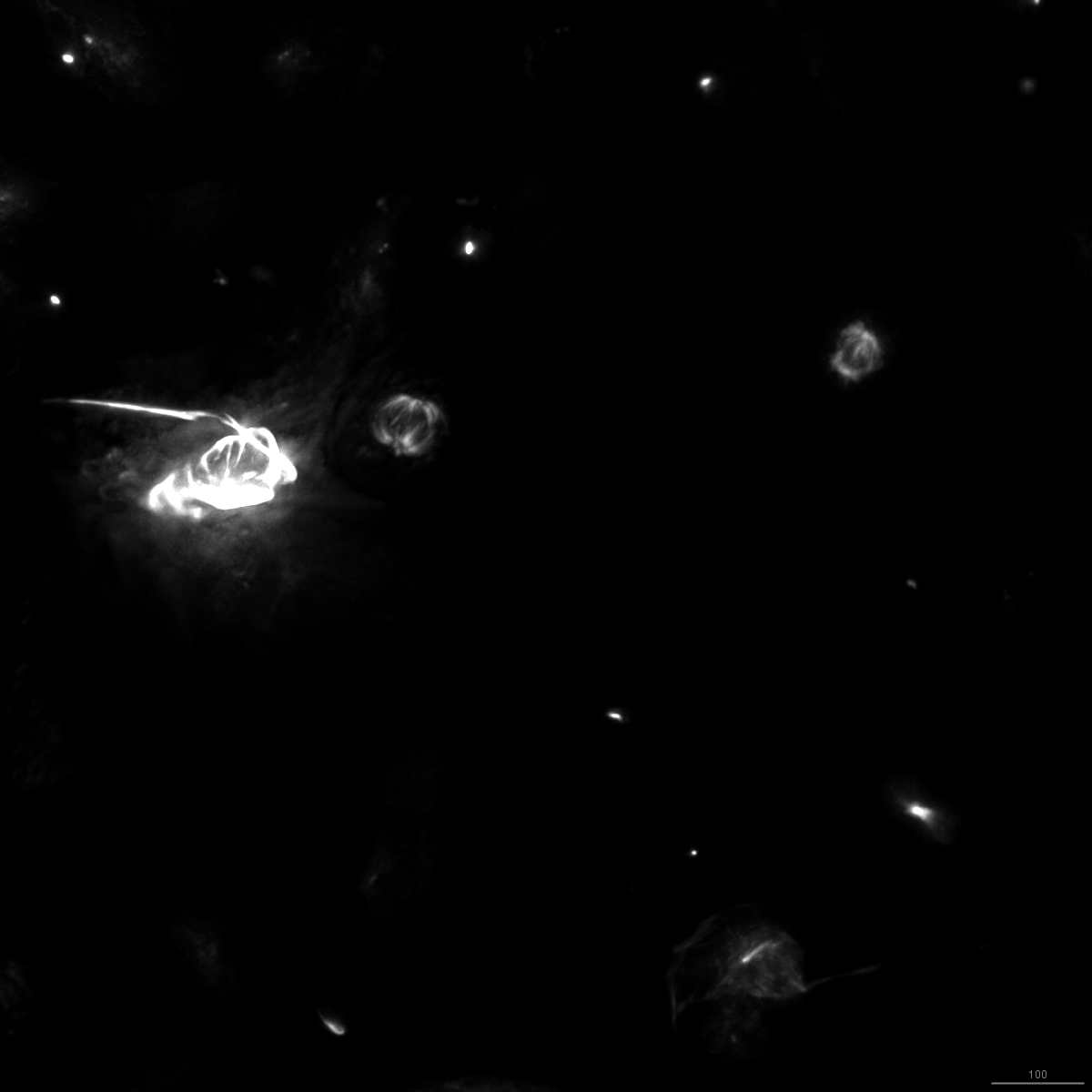

Supplement: Supplementary file 8 — Source Data for Figure 5 [file EMBJ-42-e113647-s003.zip › Figure 5/Figure 5D/wt/SiR Cy5.tif]

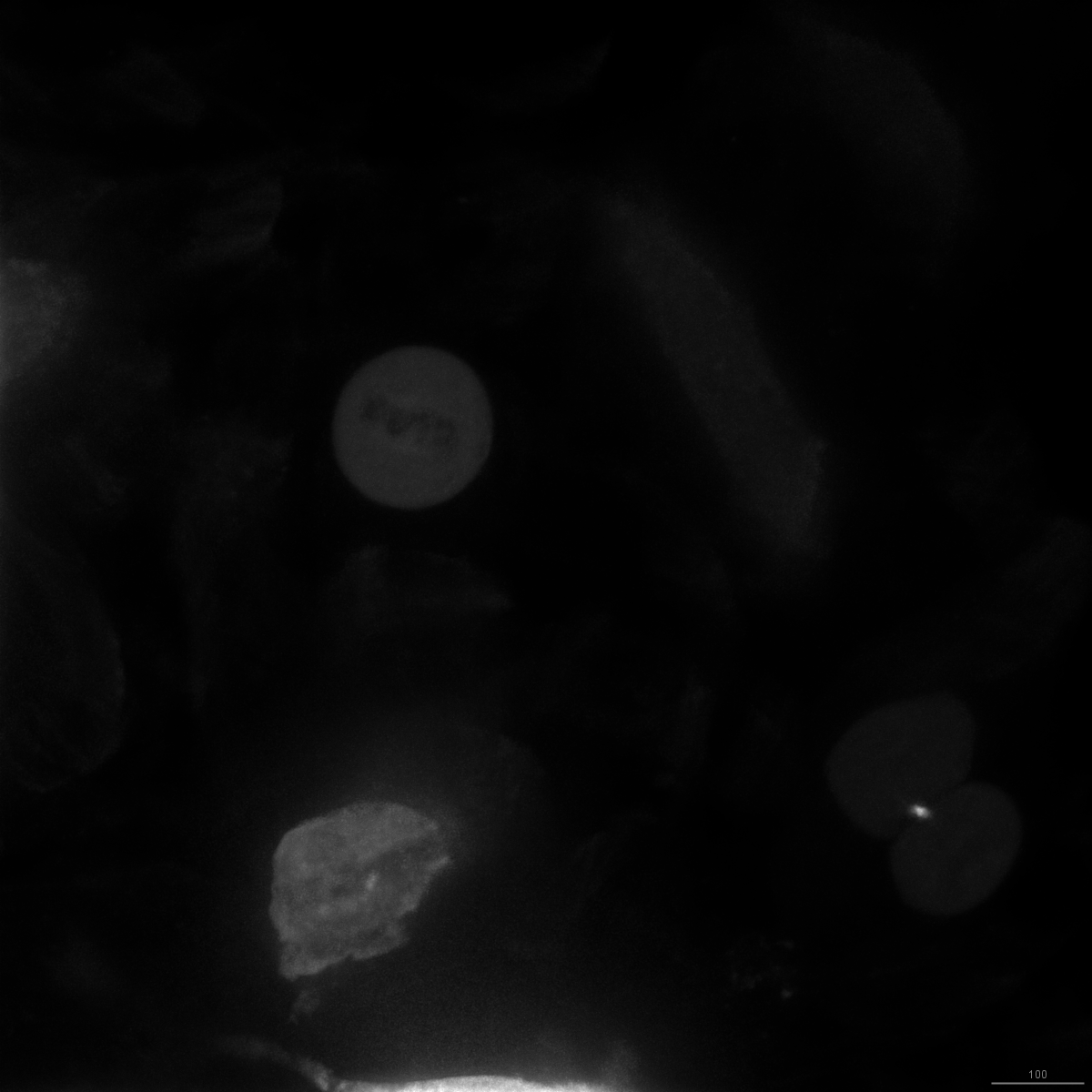

Supplement: Supplementary file 8 — Source Data for Figure 5 [file EMBJ-42-e113647-s003.zip › Figure 5/Figure 5D/wt/GFP.tif]

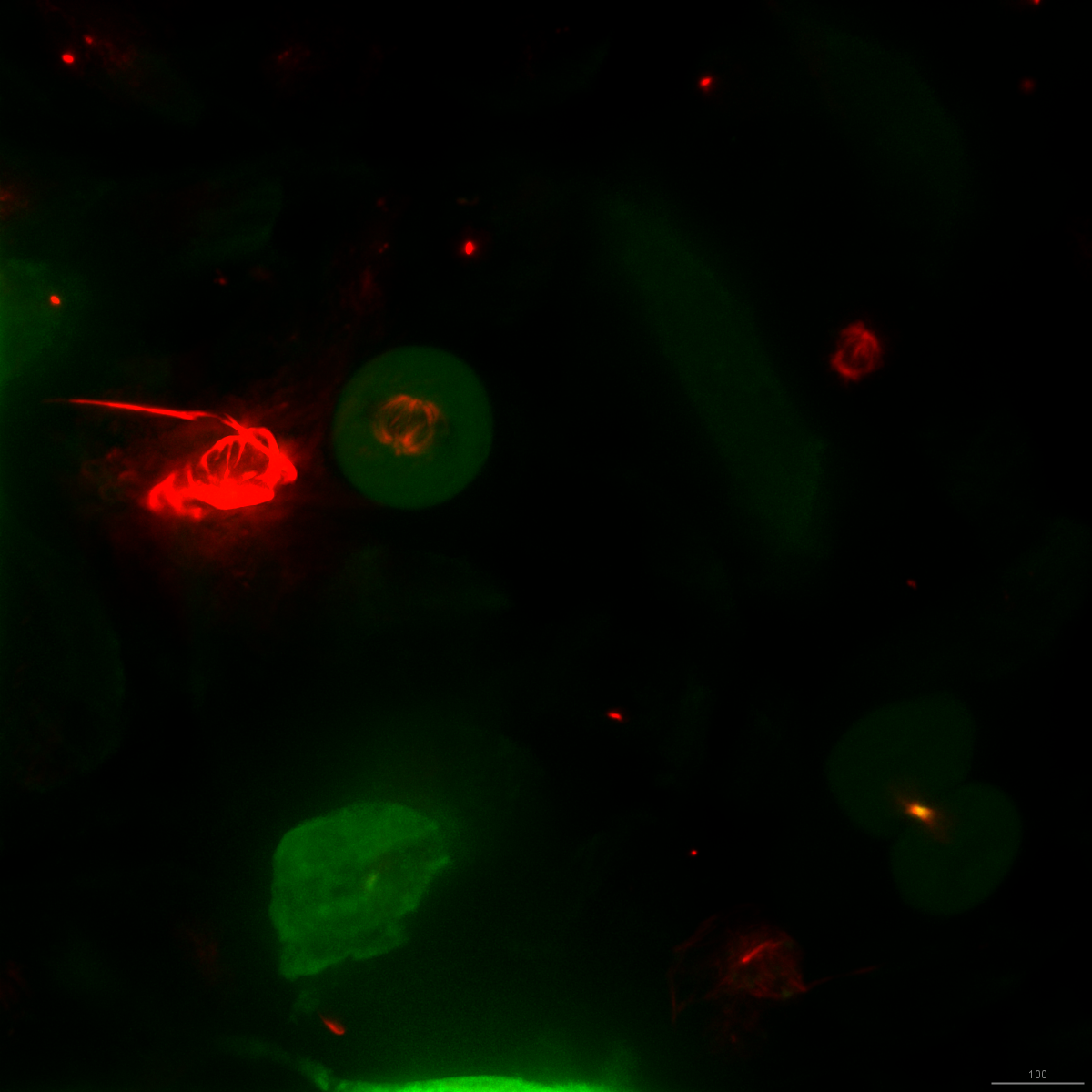

Supplement: Supplementary file 8 — Source Data for Figure 5 [file EMBJ-42-e113647-s003.zip › Figure 5/Figure 5D/wt/merge.tif]

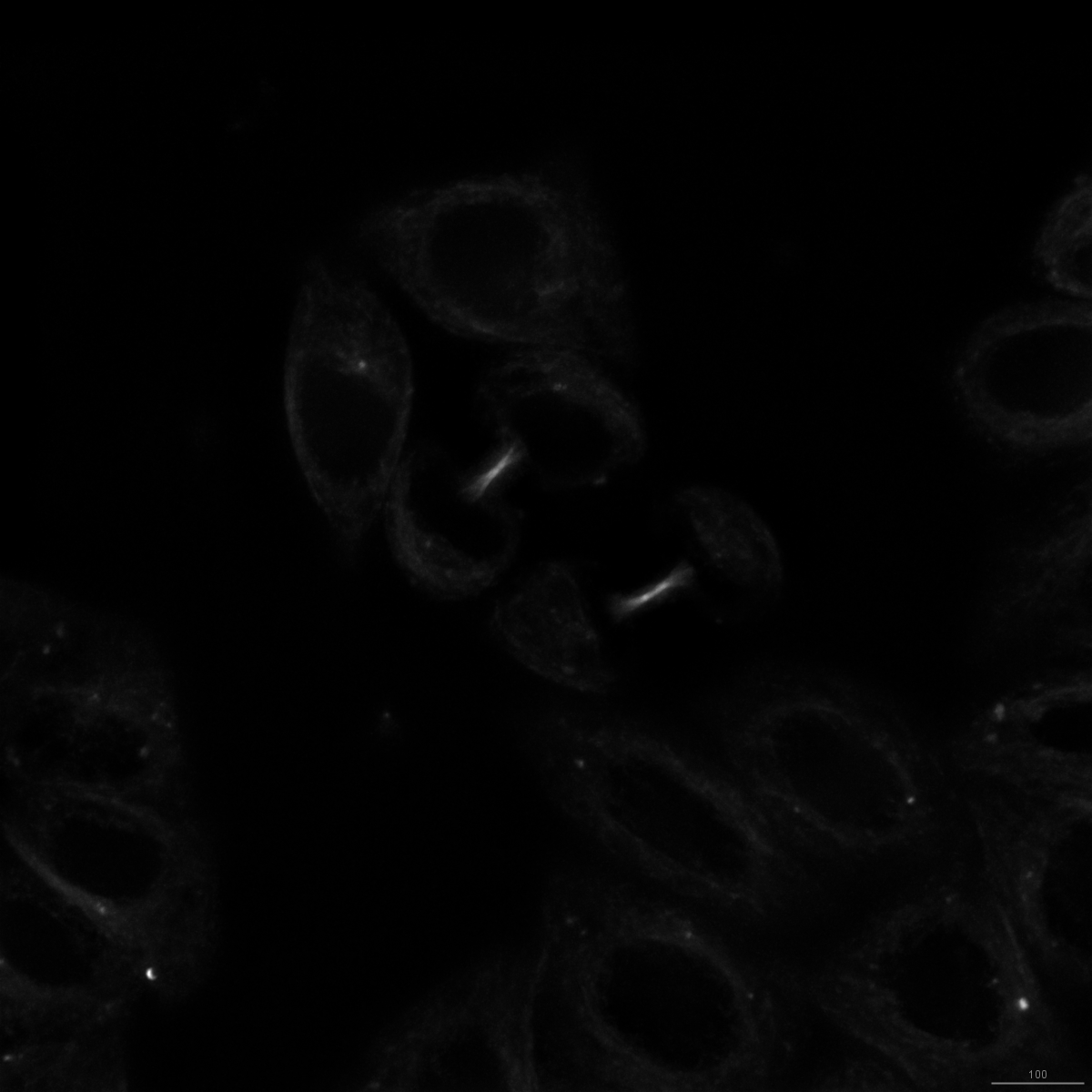

Supplement: Supplementary file 8 — Source Data for Figure 5 [file EMBJ-42-e113647-s003.zip › Figure 5/Figure 5E/GFPGSTCENPE6xStoA/GFPGSTCENPE6xStoA redSiR.tif]

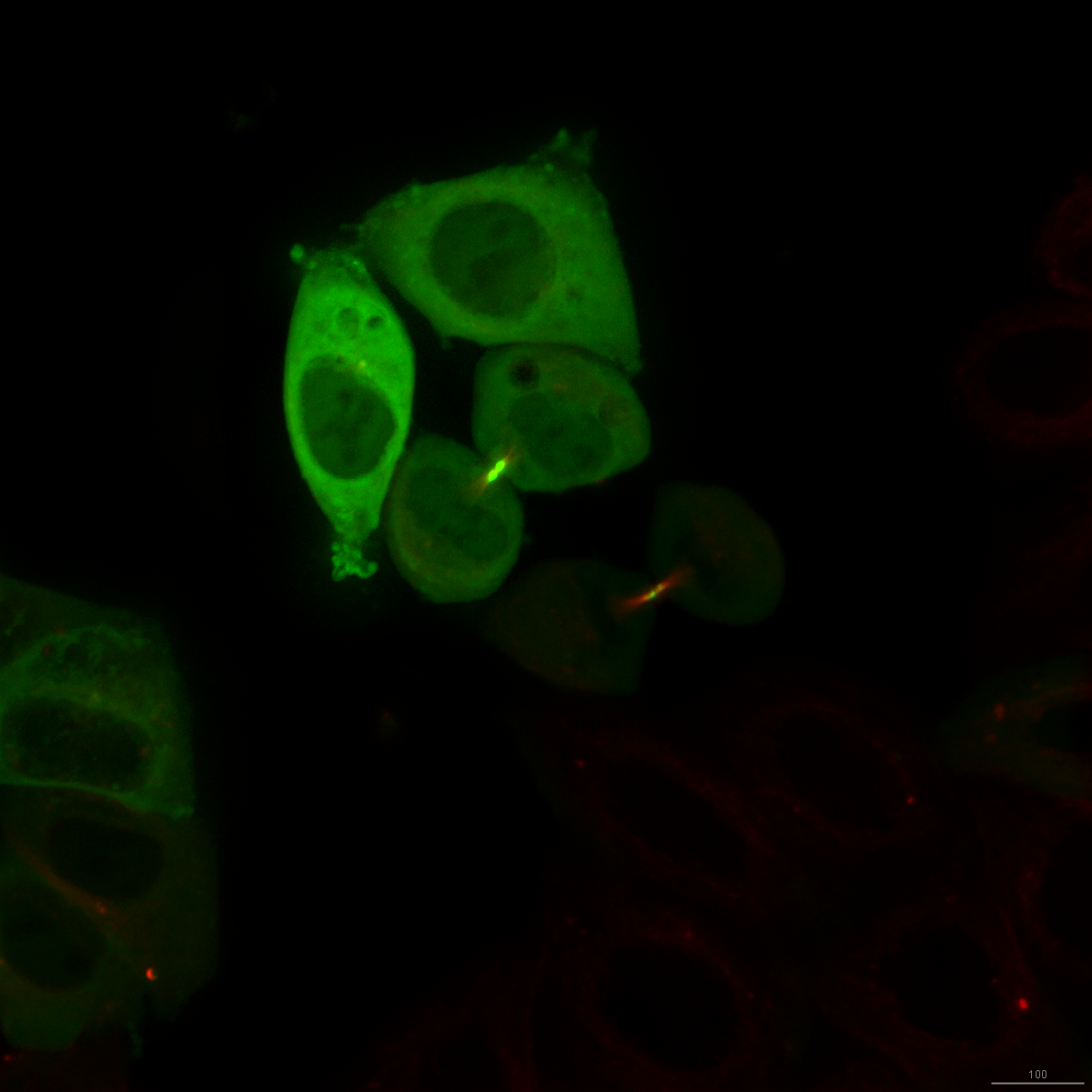

Supplement: Supplementary file 8 — Source Data for Figure 5 [file EMBJ-42-e113647-s003.zip › Figure 5/Figure 5E/GFPGSTCENPE6xStoA/GFPGSTCENPE6xStoA merge.tif]

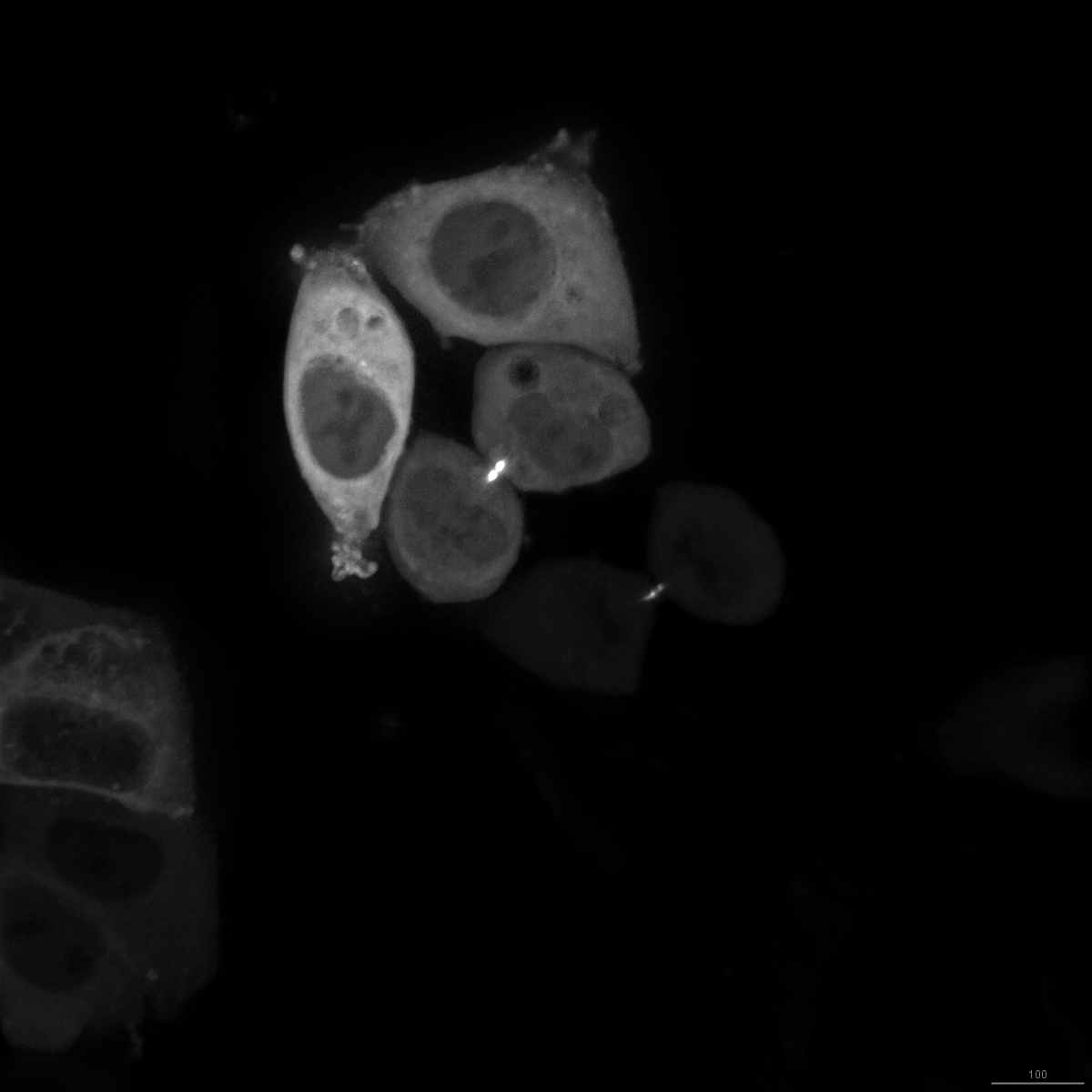

Supplement: Supplementary file 8 — Source Data for Figure 5 [file EMBJ-42-e113647-s003.zip › Figure 5/Figure 5E/GFPGSTCENPE6xStoA/GFPGSTCENPE6xStoA green.tif]

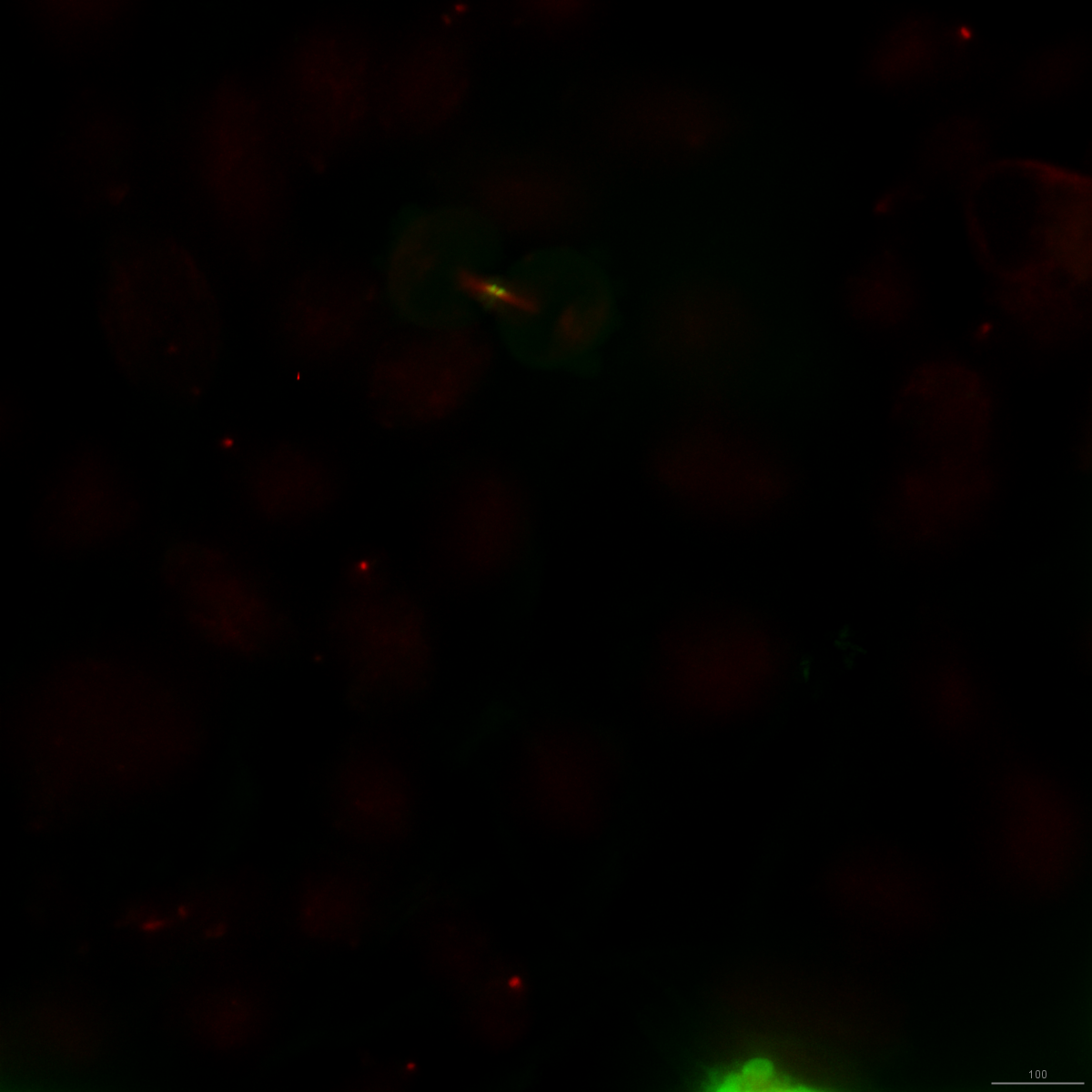

Supplement: Supplementary file 8 — Source Data for Figure 5 [file EMBJ-42-e113647-s003.zip › Figure 5/Figure 5E/GFPGSTCENPEwt/GFPGSTCENPEwt merge.tif]

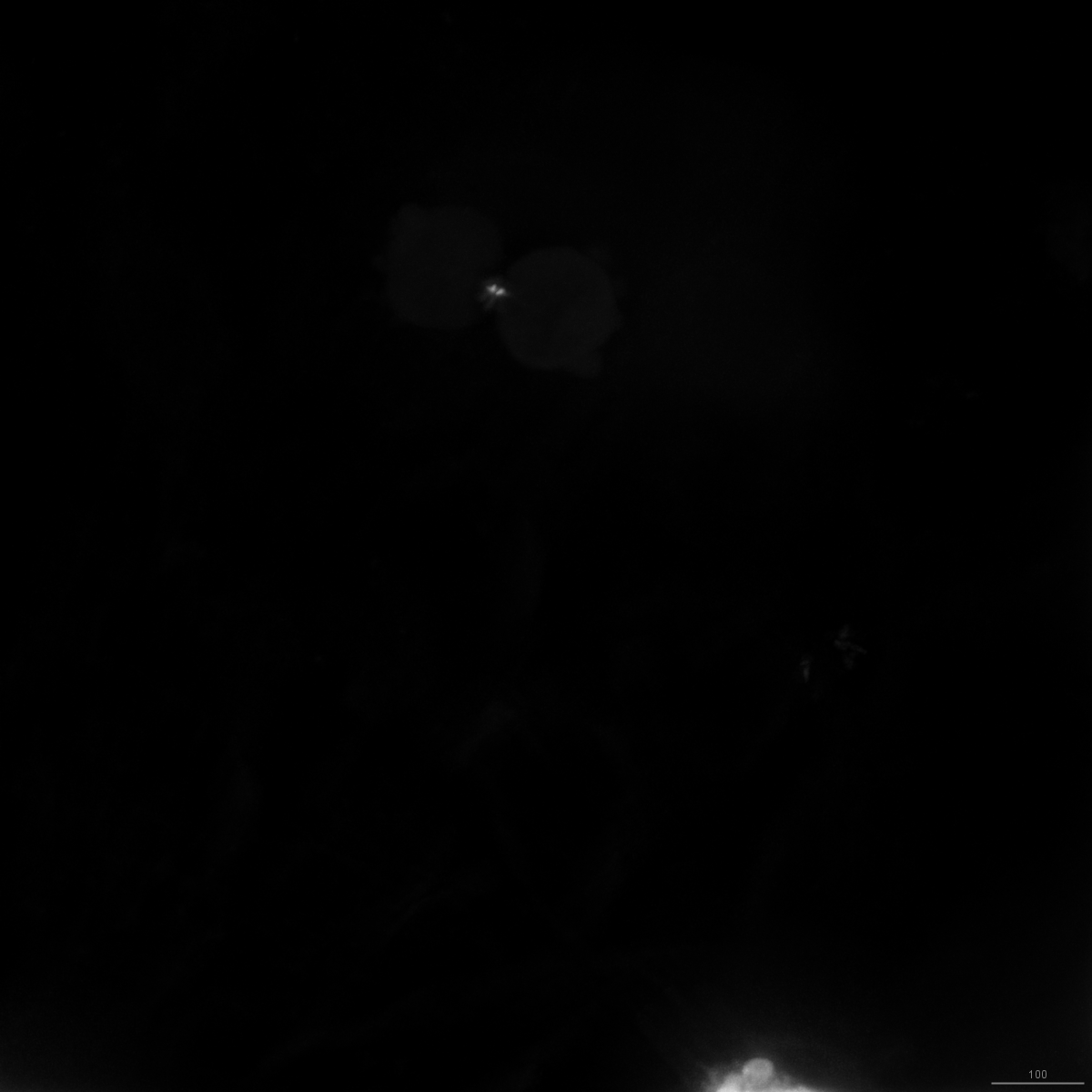

Supplement: Supplementary file 8 — Source Data for Figure 5 [file EMBJ-42-e113647-s003.zip › Figure 5/Figure 5E/GFPGSTCENPEwt/GFPGSTCENPEwt green.tif]

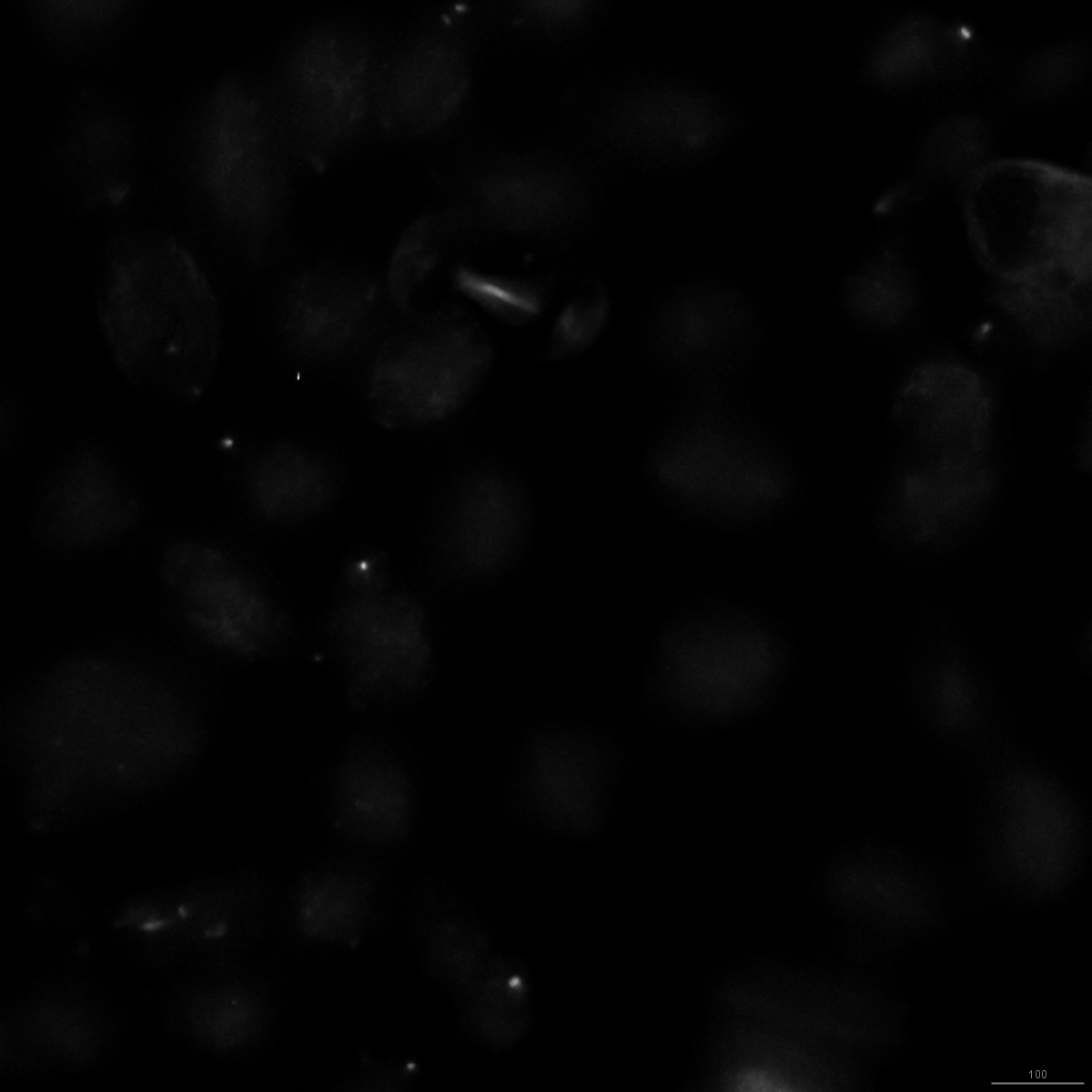

Supplement: Supplementary file 8 — Source Data for Figure 5 [file EMBJ-42-e113647-s003.zip › Figure 5/Figure 5E/GFPGSTCENPEwt/GFPGSTCENPEwt redSiR.tif]

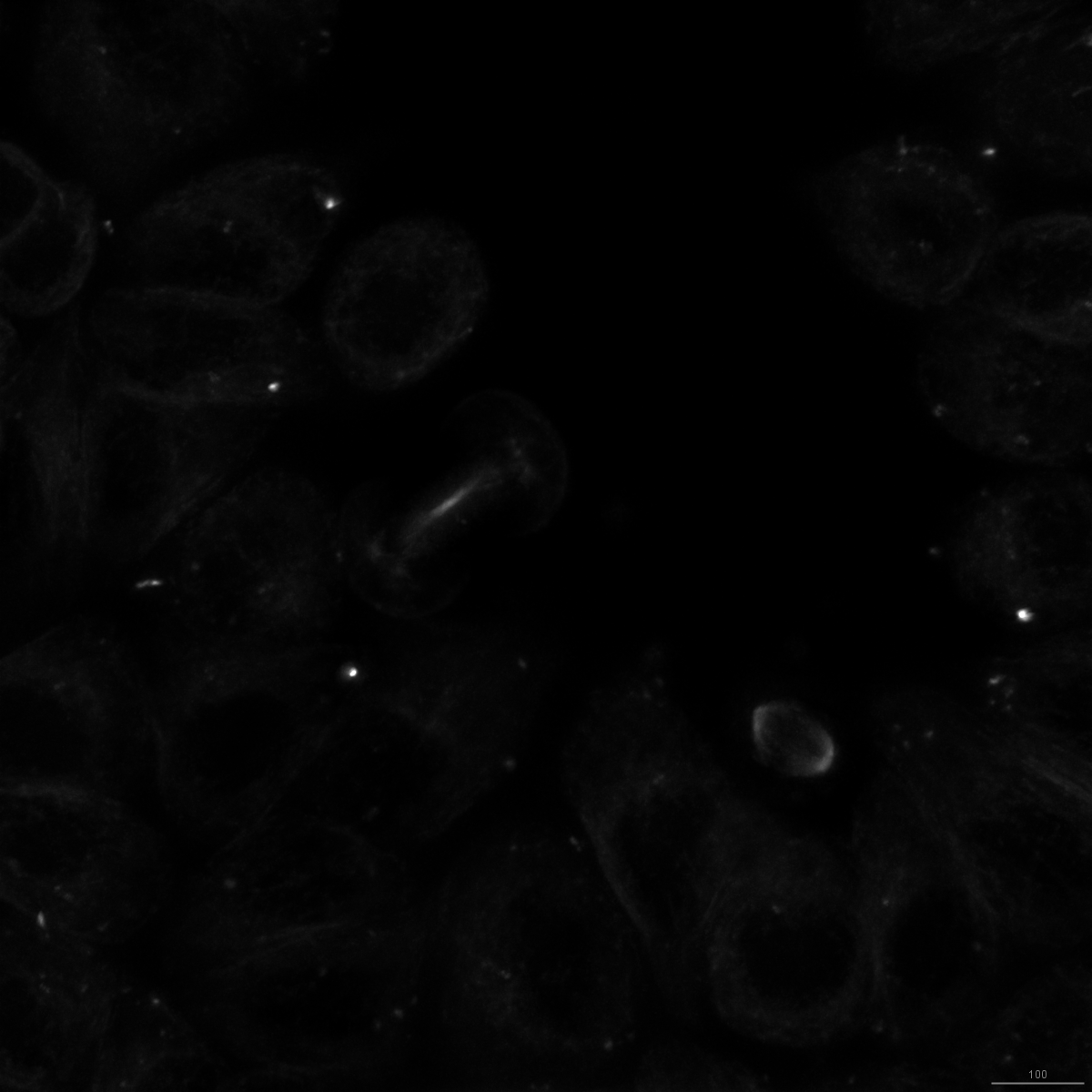

Supplement: Supplementary file 8 — Source Data for Figure 5 [file EMBJ-42-e113647-s003.zip › Figure 5/Figure 5E/GFPGSTCENPE6xStoD/GFPGSTCENPE6xStoD redSiR.tif]

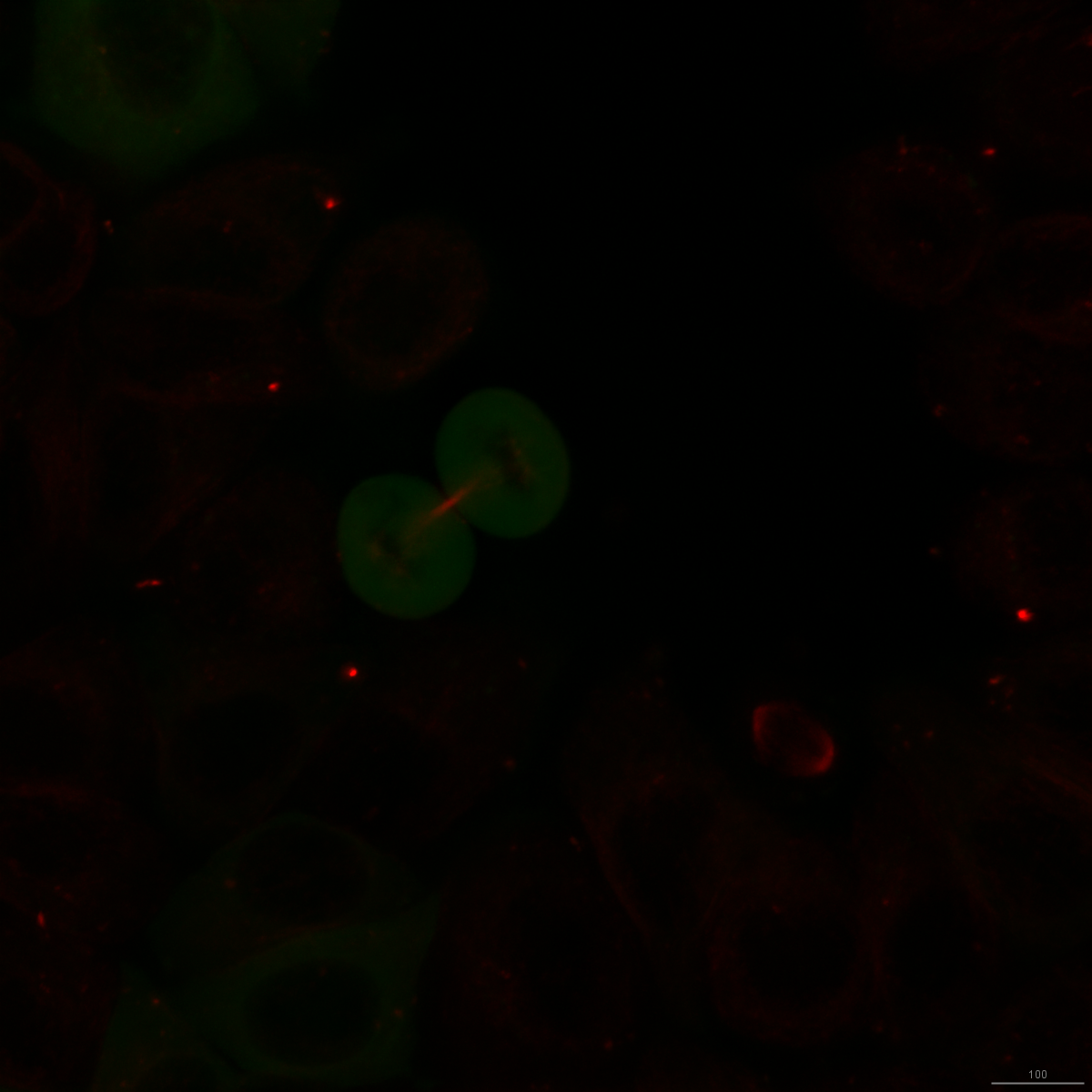

Supplement: Supplementary file 8 — Source Data for Figure 5 [file EMBJ-42-e113647-s003.zip › Figure 5/Figure 5E/GFPGSTCENPE6xStoD/GFPGSTCENPE6xStoD merge.tif]

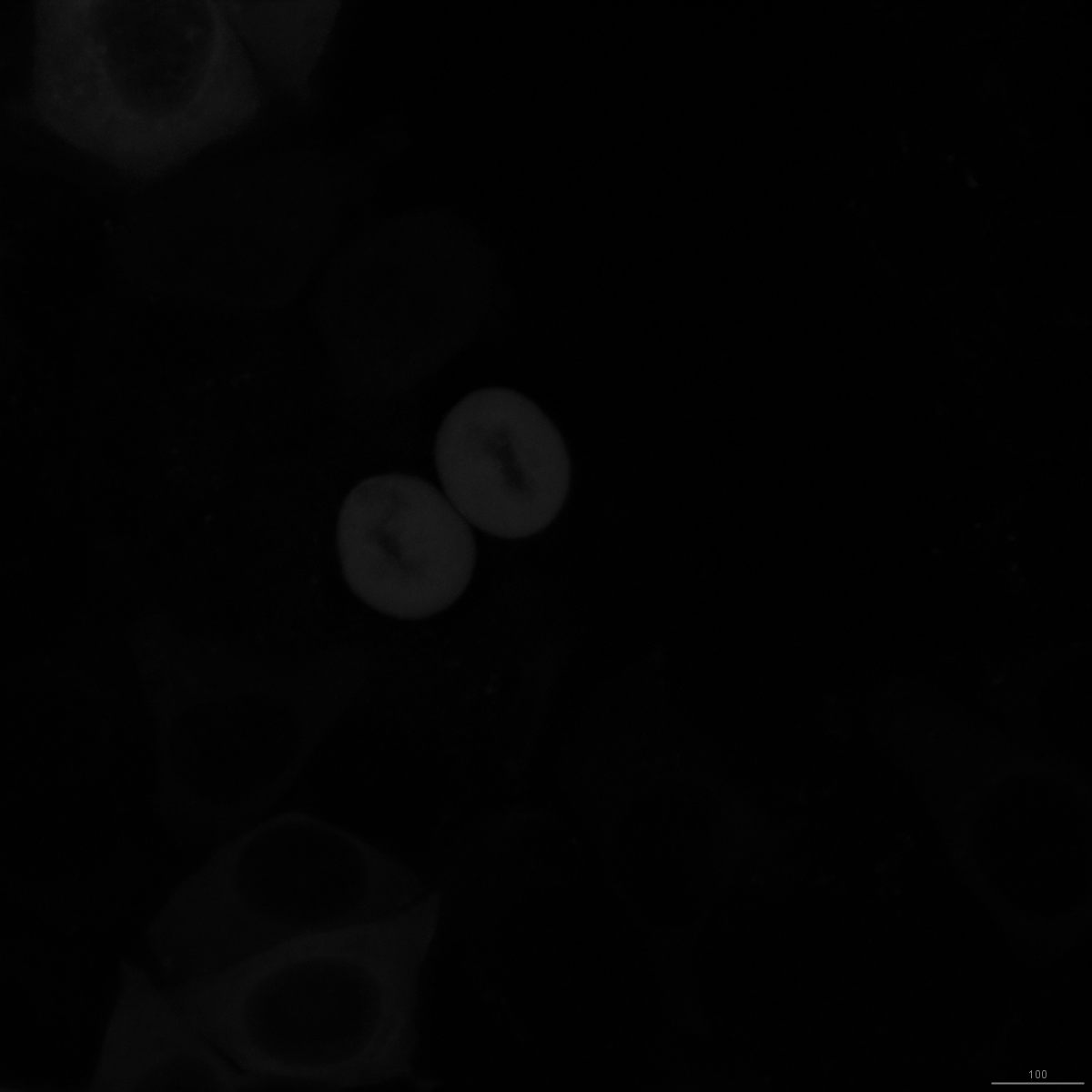

Supplement: Supplementary file 8 — Source Data for Figure 5 [file EMBJ-42-e113647-s003.zip › Figure 5/Figure 5E/GFPGSTCENPE6xStoD/GFPGSTCENPE6xStoD green.tif]

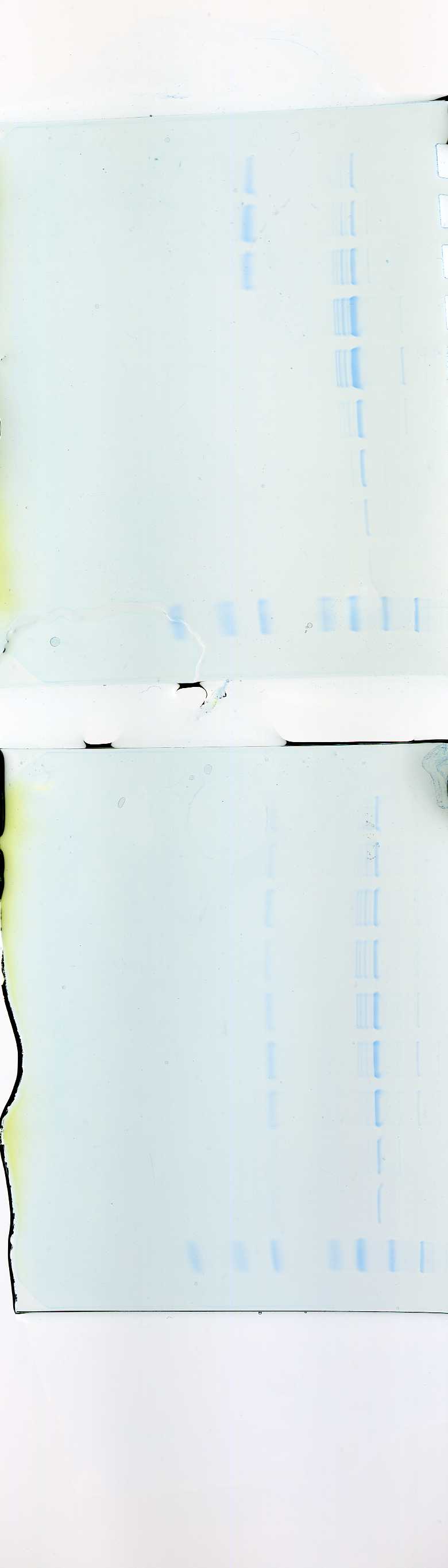

Supplement: Supplementary file 9 — Source Data for Figure 6 [file EMBJ-42-e113647-s002.zip › Figure 6/Figure 6E/MBPCENPEwith PRC1WT and PRC1MEE .jpg]

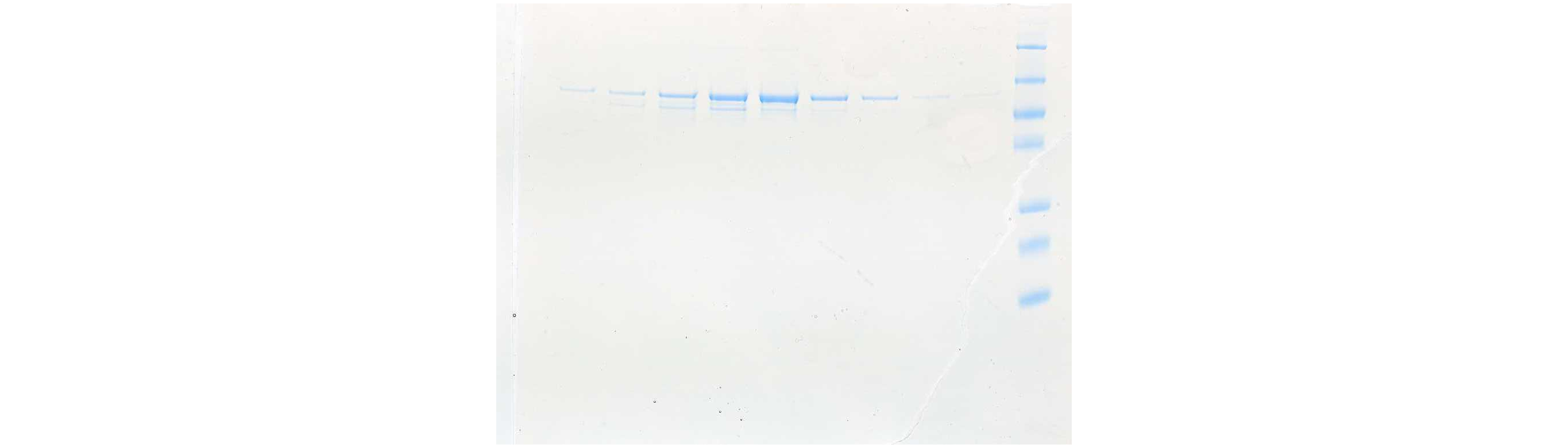

Supplement: Supplementary file 9 — Source Data for Figure 6 [file EMBJ-42-e113647-s002.zip › Figure 6/Figure 6E/MBPCENPE alone.jpg]

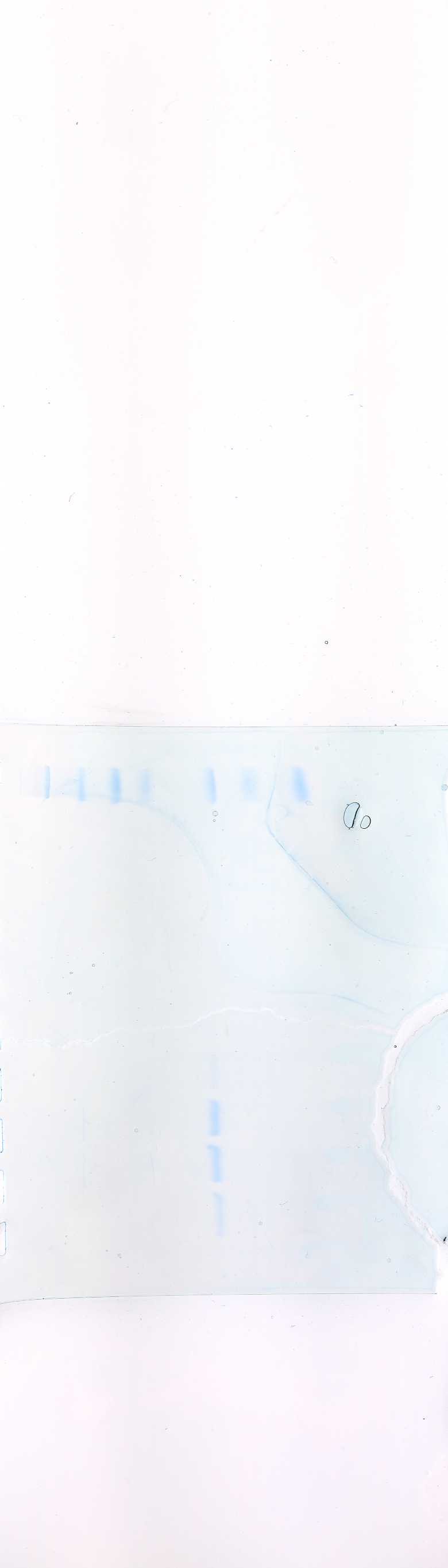

Supplement: Supplementary file 9 — Source Data for Figure 6 [file EMBJ-42-e113647-s002.zip › Figure 6/Figure 6E/PRC1MEE alone.jpg]

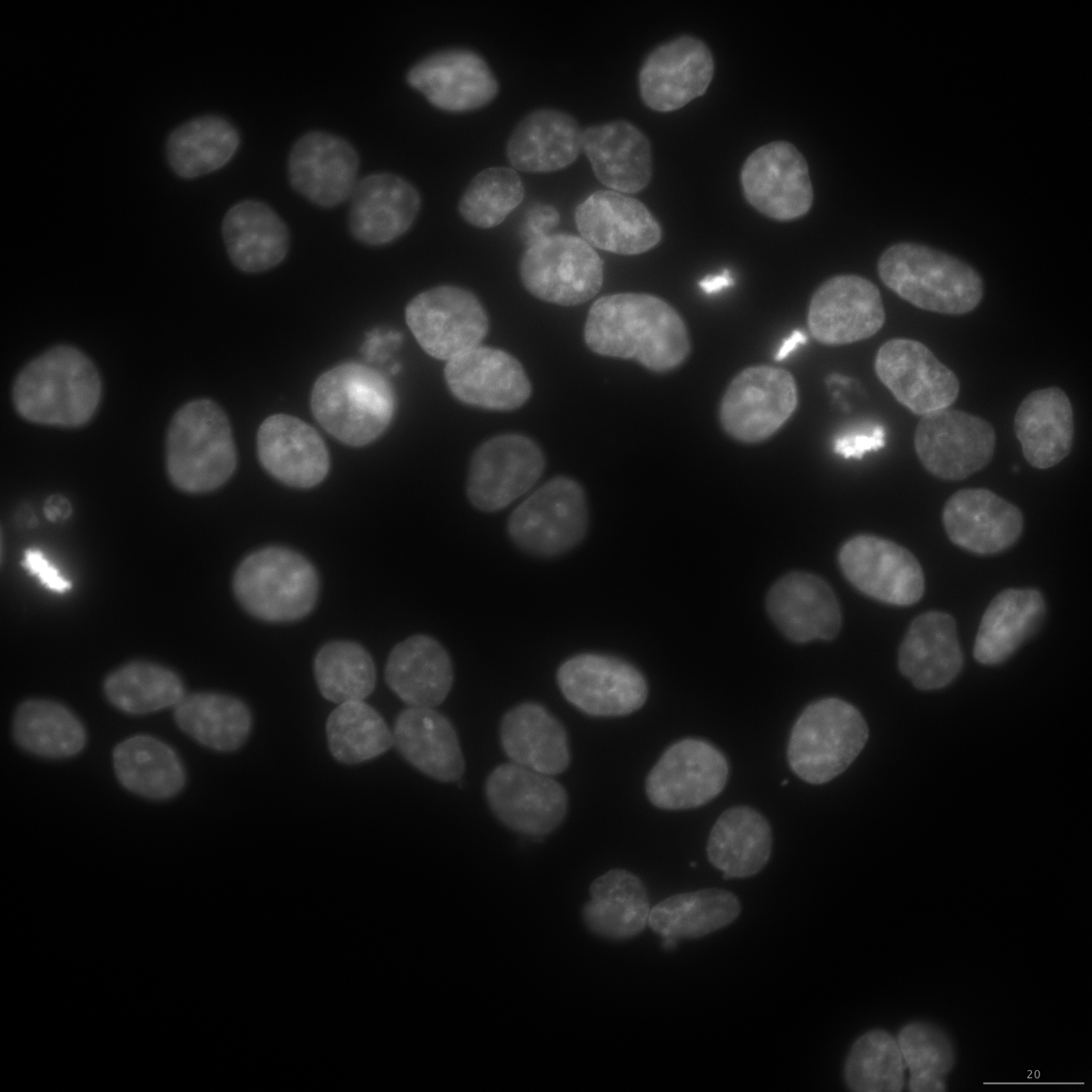

Supplement: Supplementary file 10 — Source Data for Figure 7 [file EMBJ-42-e113647-s009.zip › Figure 7/Figure 7J/Prc1MEE DNA.png]

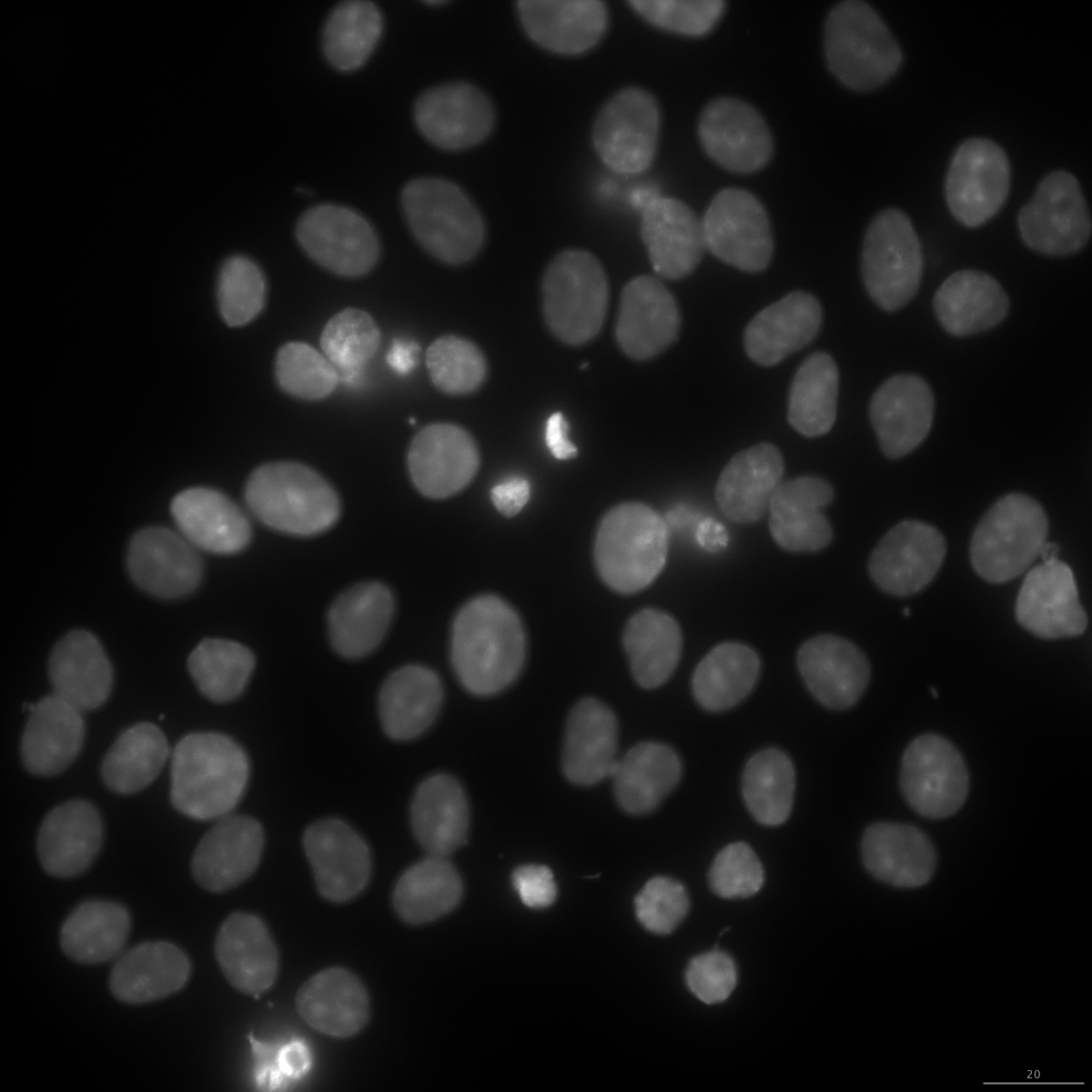

Supplement: Supplementary file 10 — Source Data for Figure 7 [file EMBJ-42-e113647-s009.zip › Figure 7/Figure 7J/Prc1WT DNA.png]

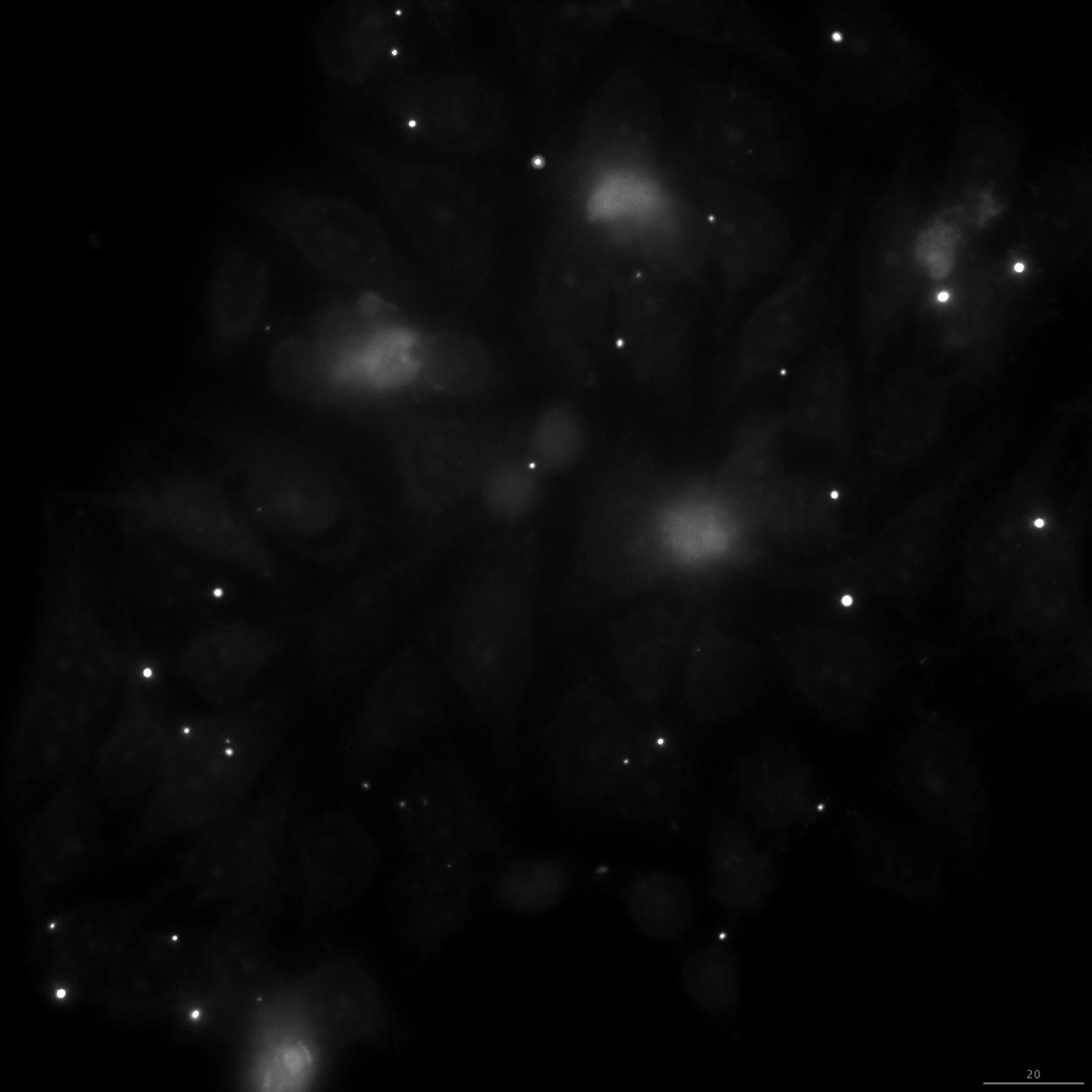

Supplement: Supplementary file 10 — Source Data for Figure 7 [file EMBJ-42-e113647-s009.zip › Figure 7/Figure 7J/Prc1WT GFP.png]

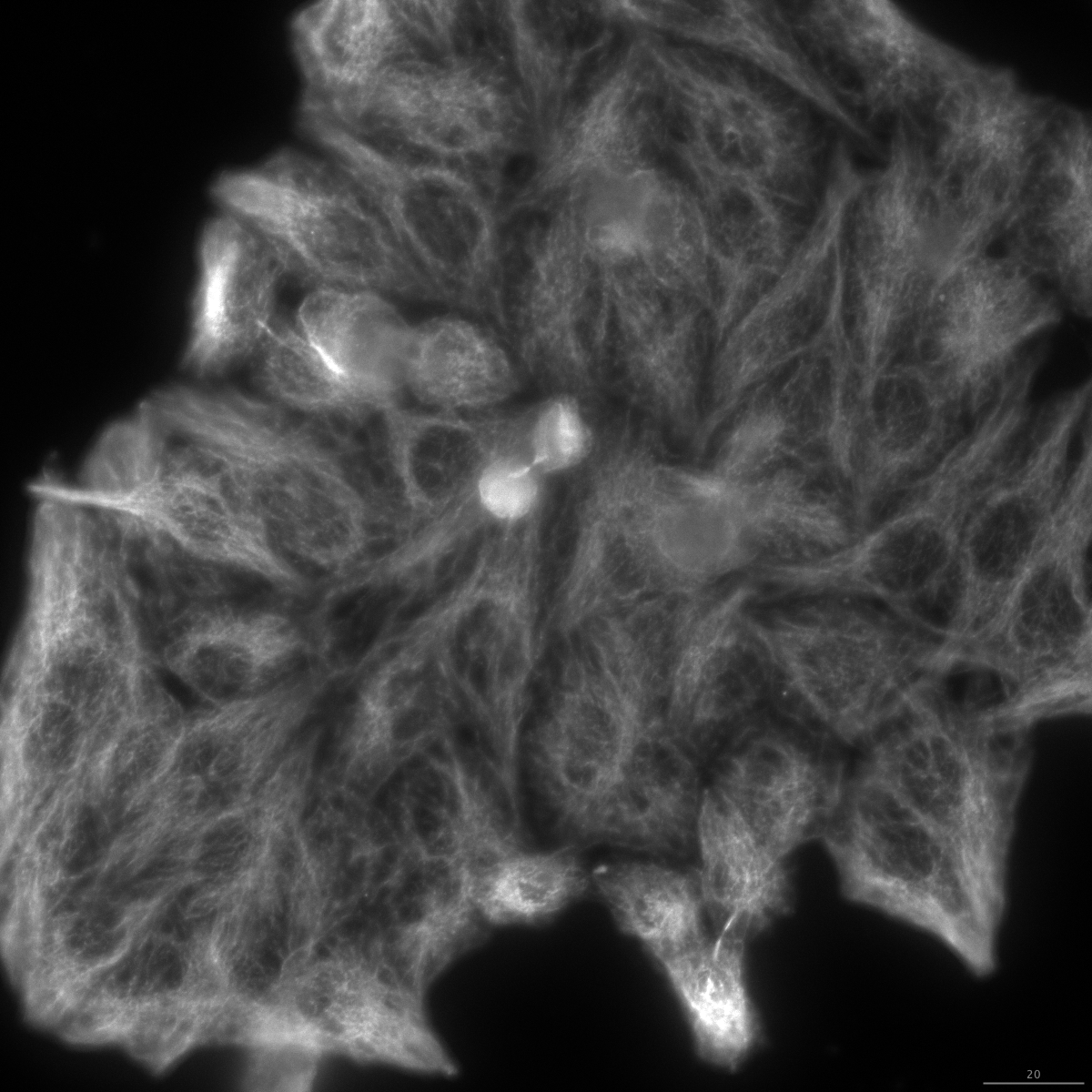

Supplement: Supplementary file 10 — Source Data for Figure 7 [file EMBJ-42-e113647-s009.zip › Figure 7/Figure 7J/Prc1WT MT.png]

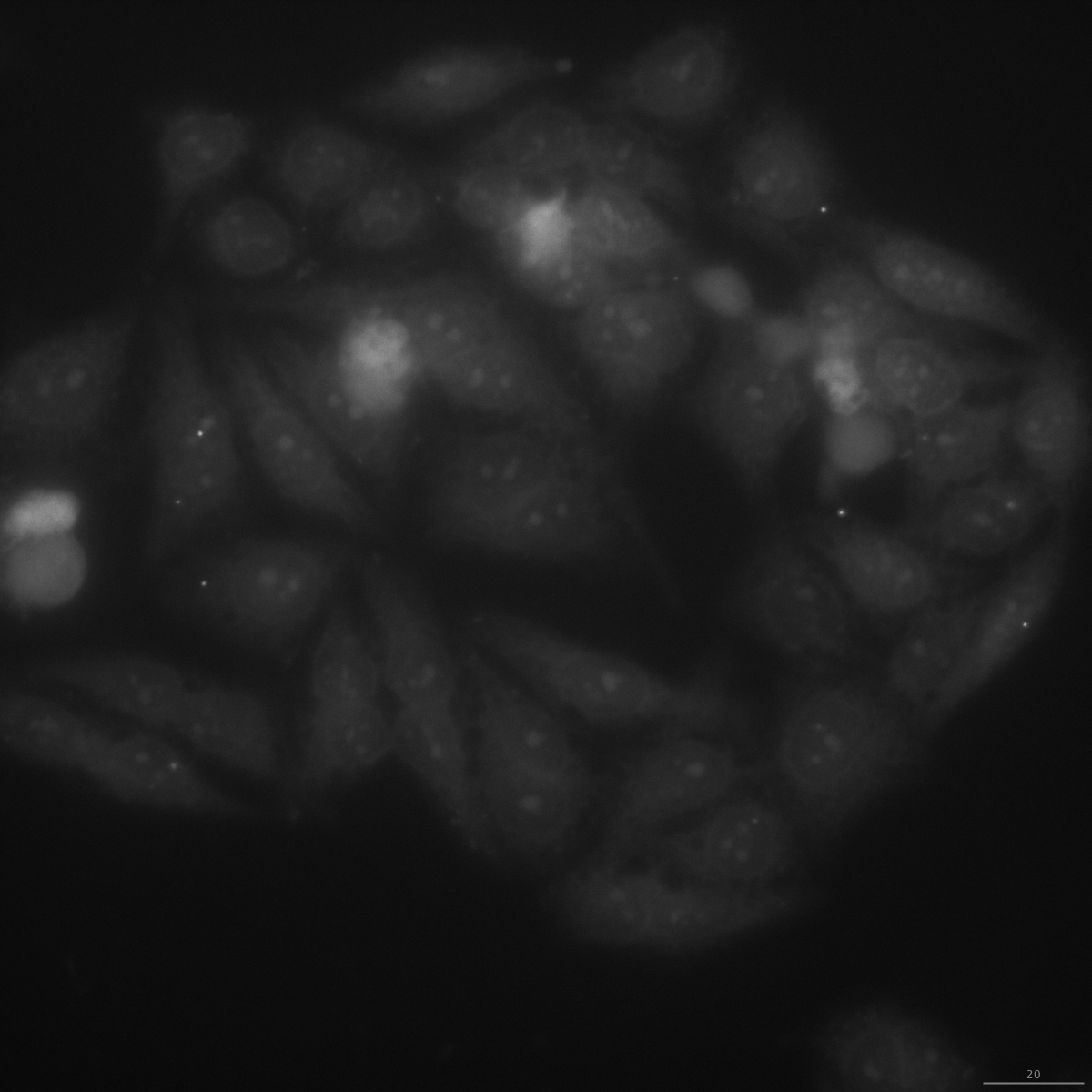

Supplement: Supplementary file 10 — Source Data for Figure 7 [file EMBJ-42-e113647-s009.zip › Figure 7/Figure 7J/Prc1MEE GFP.png]

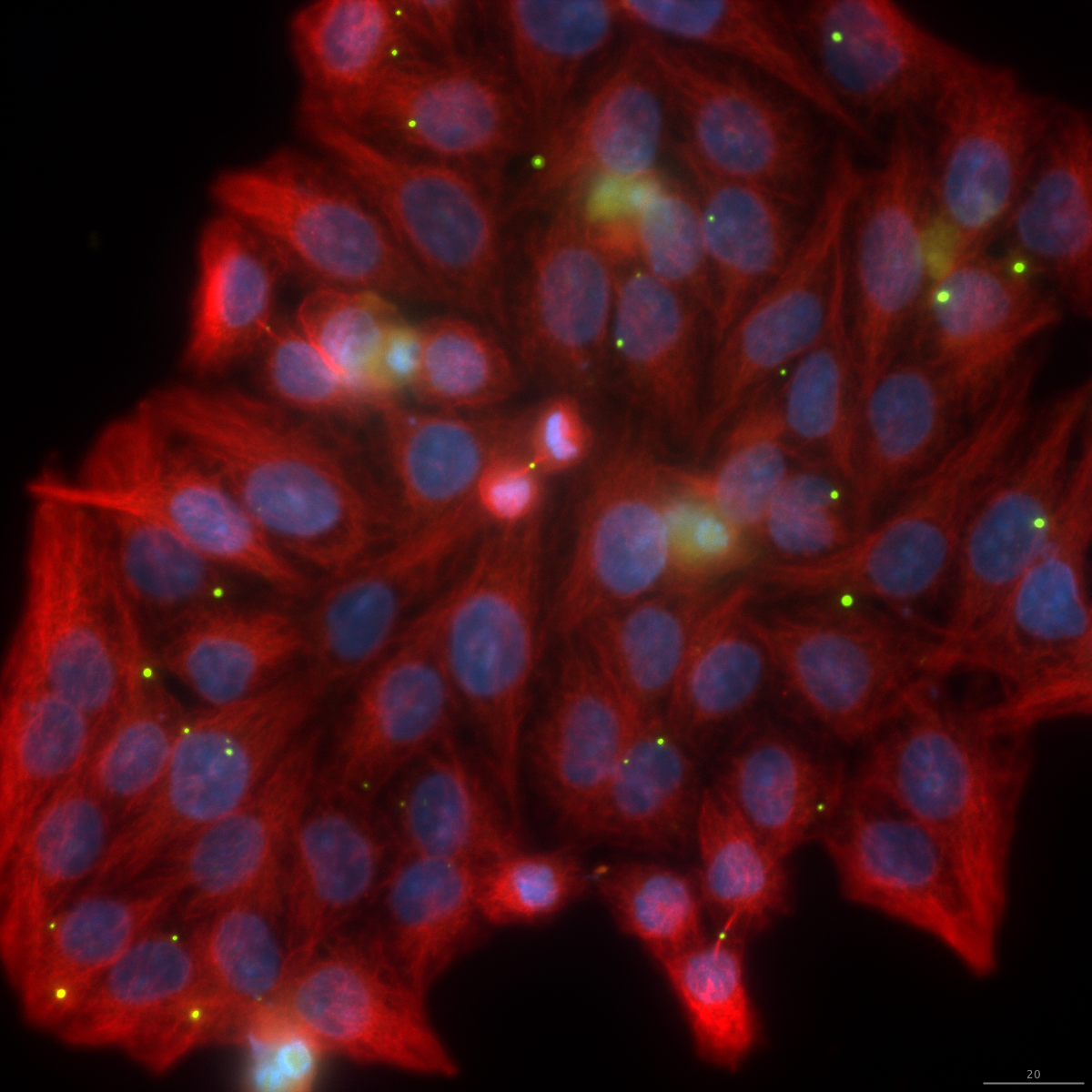

Supplement: Supplementary file 10 — Source Data for Figure 7 [file EMBJ-42-e113647-s009.zip › Figure 7/Figure 7J/Prc1WT merge.png]

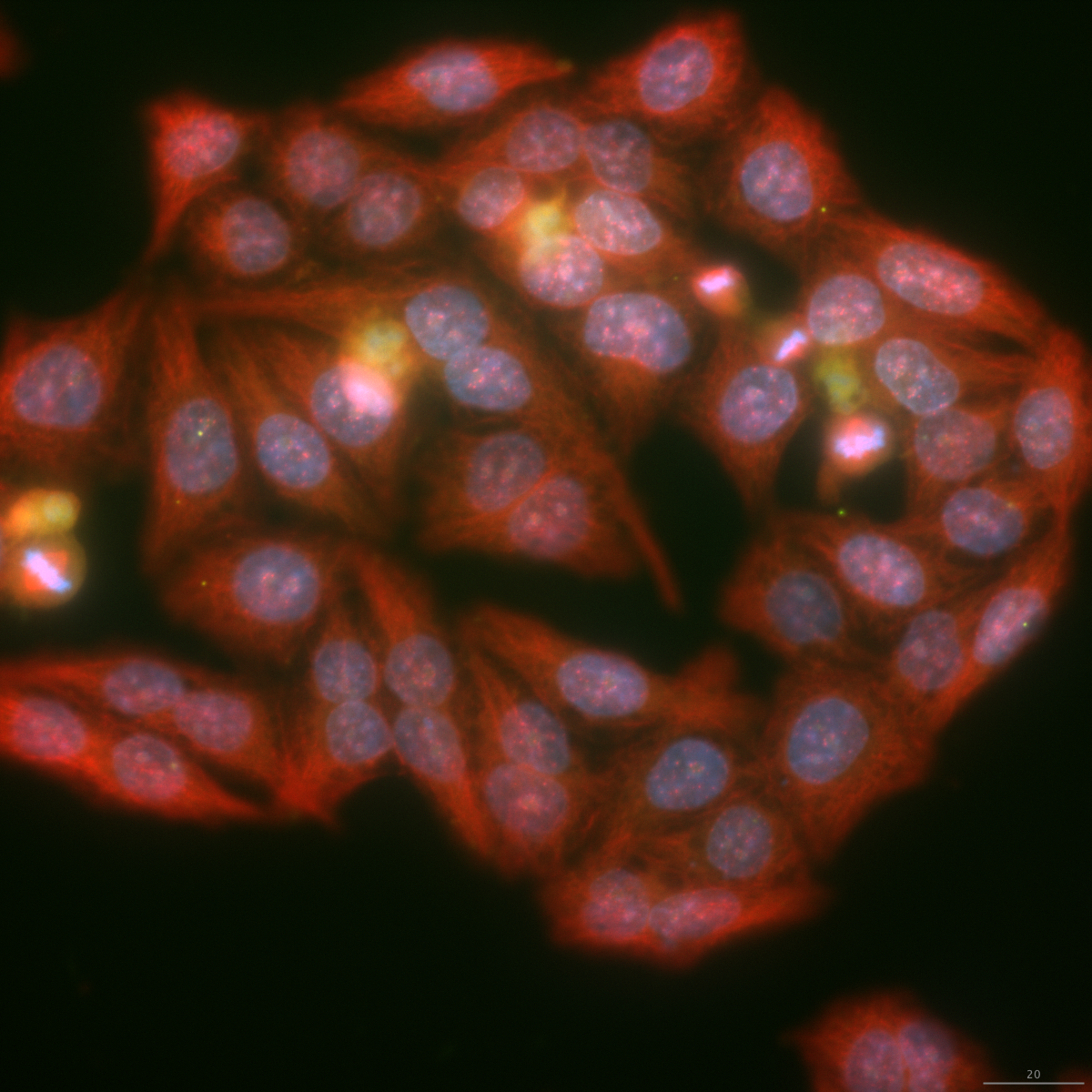

Supplement: Supplementary file 10 — Source Data for Figure 7 [file EMBJ-42-e113647-s009.zip › Figure 7/Figure 7J/Prc1MEE merge.png]

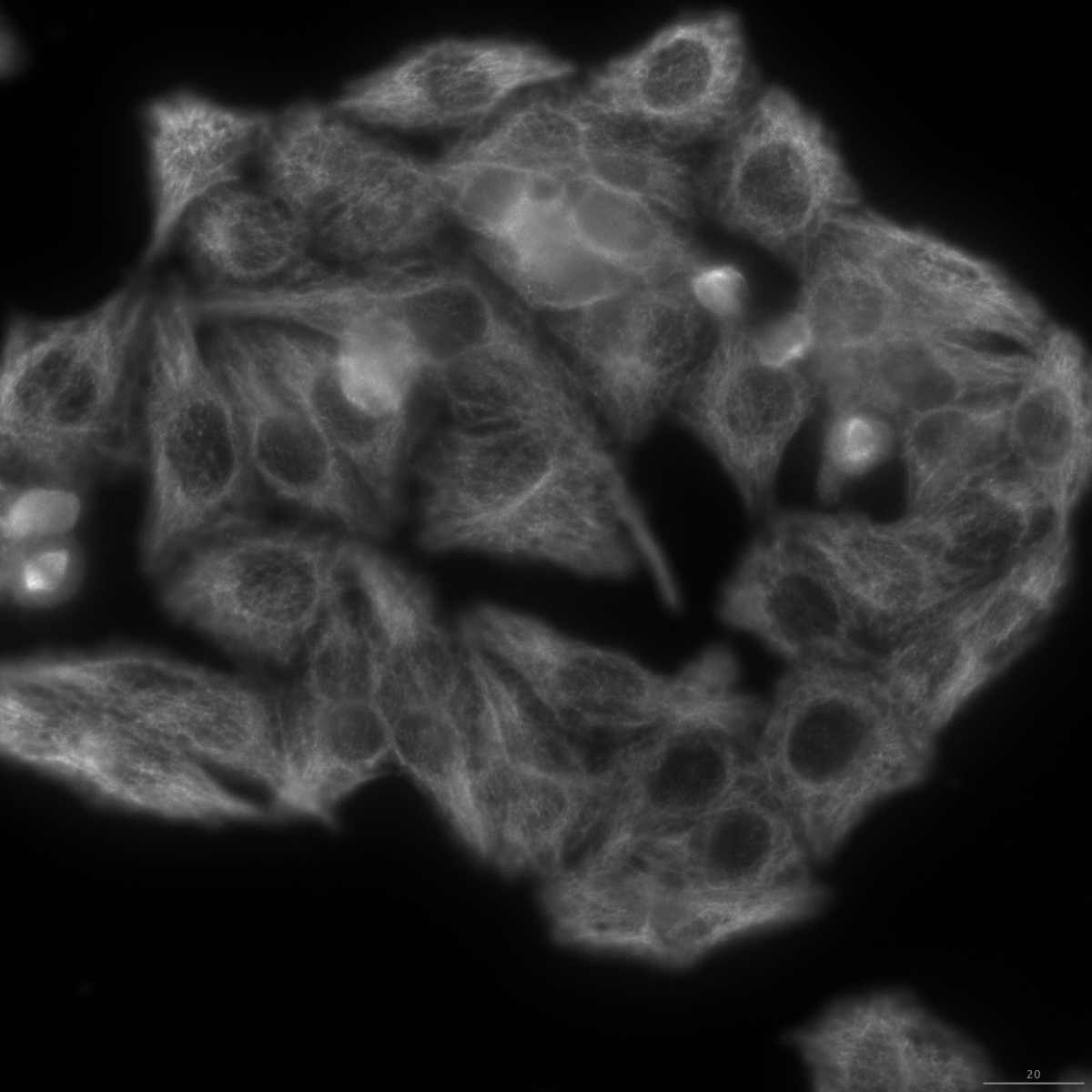

Supplement: Supplementary file 10 — Source Data for Figure 7 [file EMBJ-42-e113647-s009.zip › Figure 7/Figure 7J/Prc1MEE MT.png]

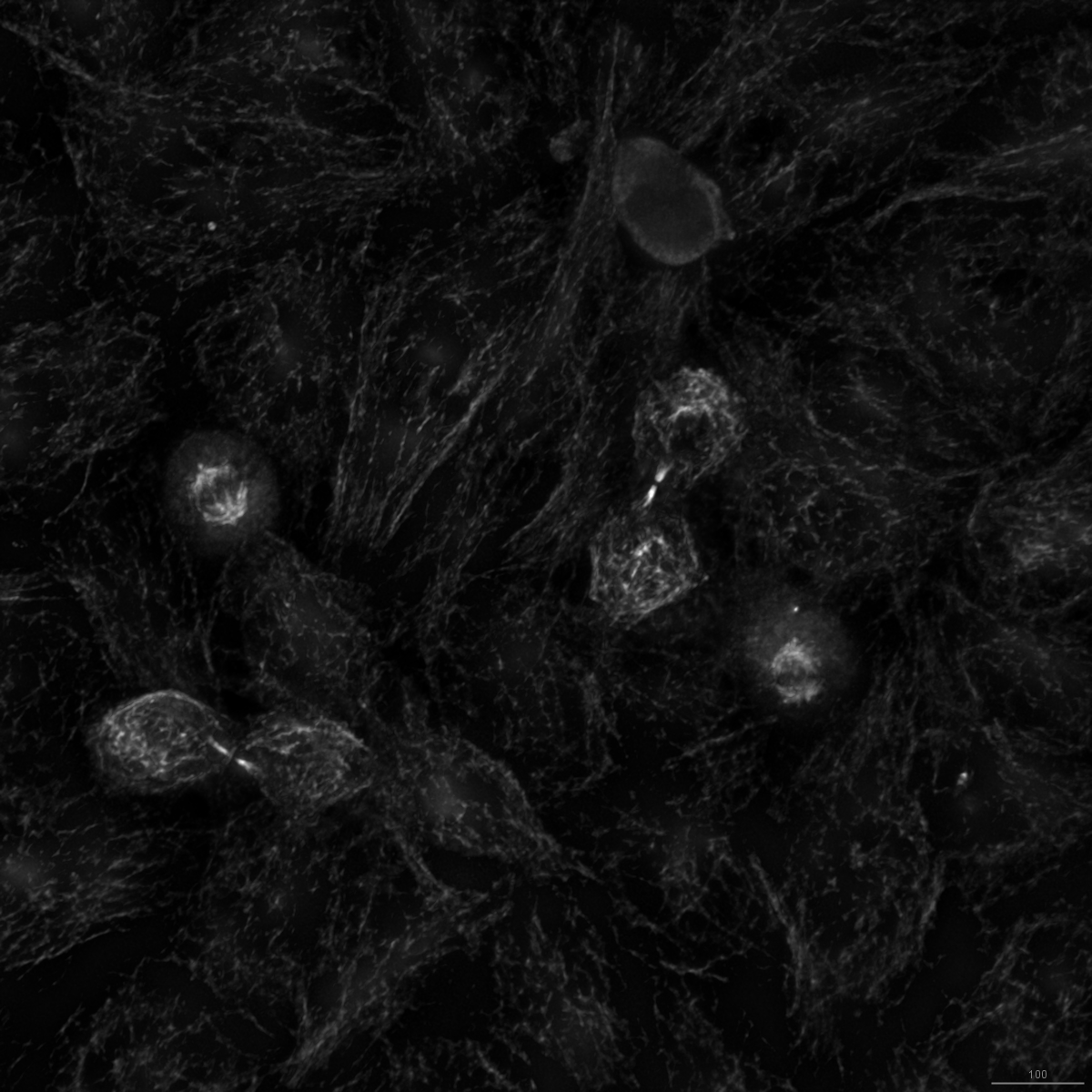

Supplement: Supplementary file 10 — Source Data for Figure 7 [file EMBJ-42-e113647-s009.zip › Figure 7/Figure 7D/PRC1 WT/MT.tif]

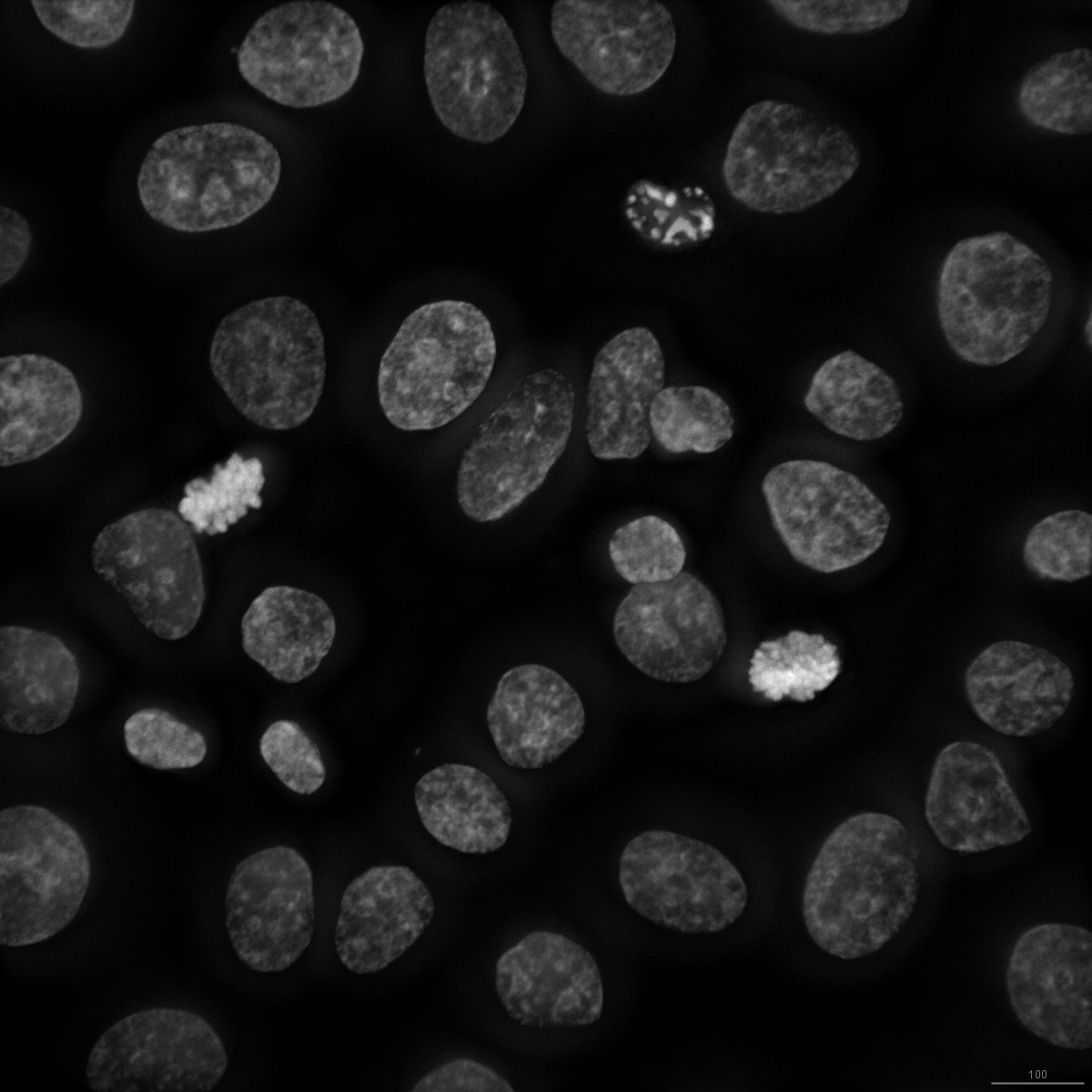

Supplement: Supplementary file 10 — Source Data for Figure 7 [file EMBJ-42-e113647-s009.zip › Figure 7/Figure 7D/PRC1 WT/DNA.tif]

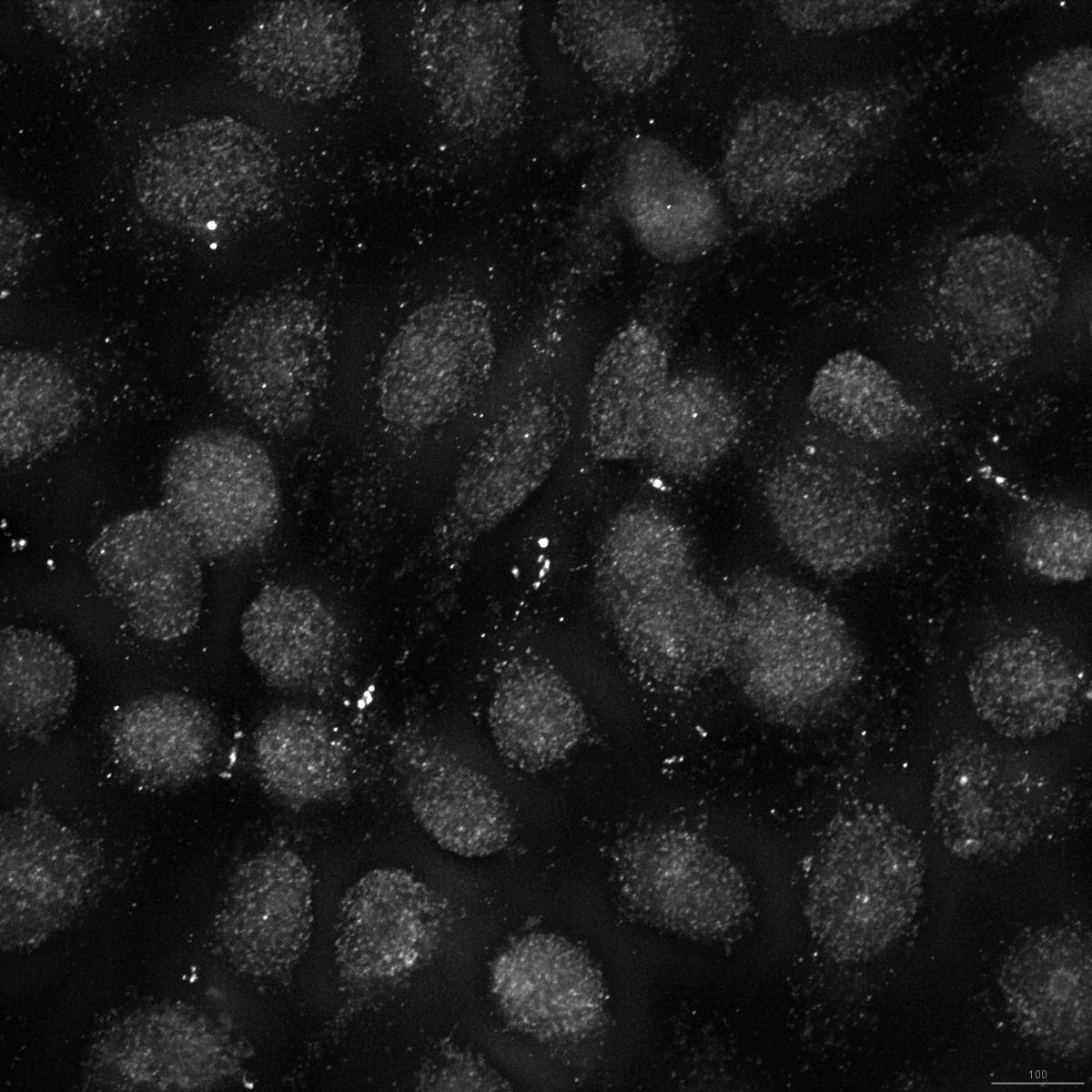

Supplement: Supplementary file 10 — Source Data for Figure 7 [file EMBJ-42-e113647-s009.zip › Figure 7/Figure 7D/PRC1 WT/Kif4.tif]

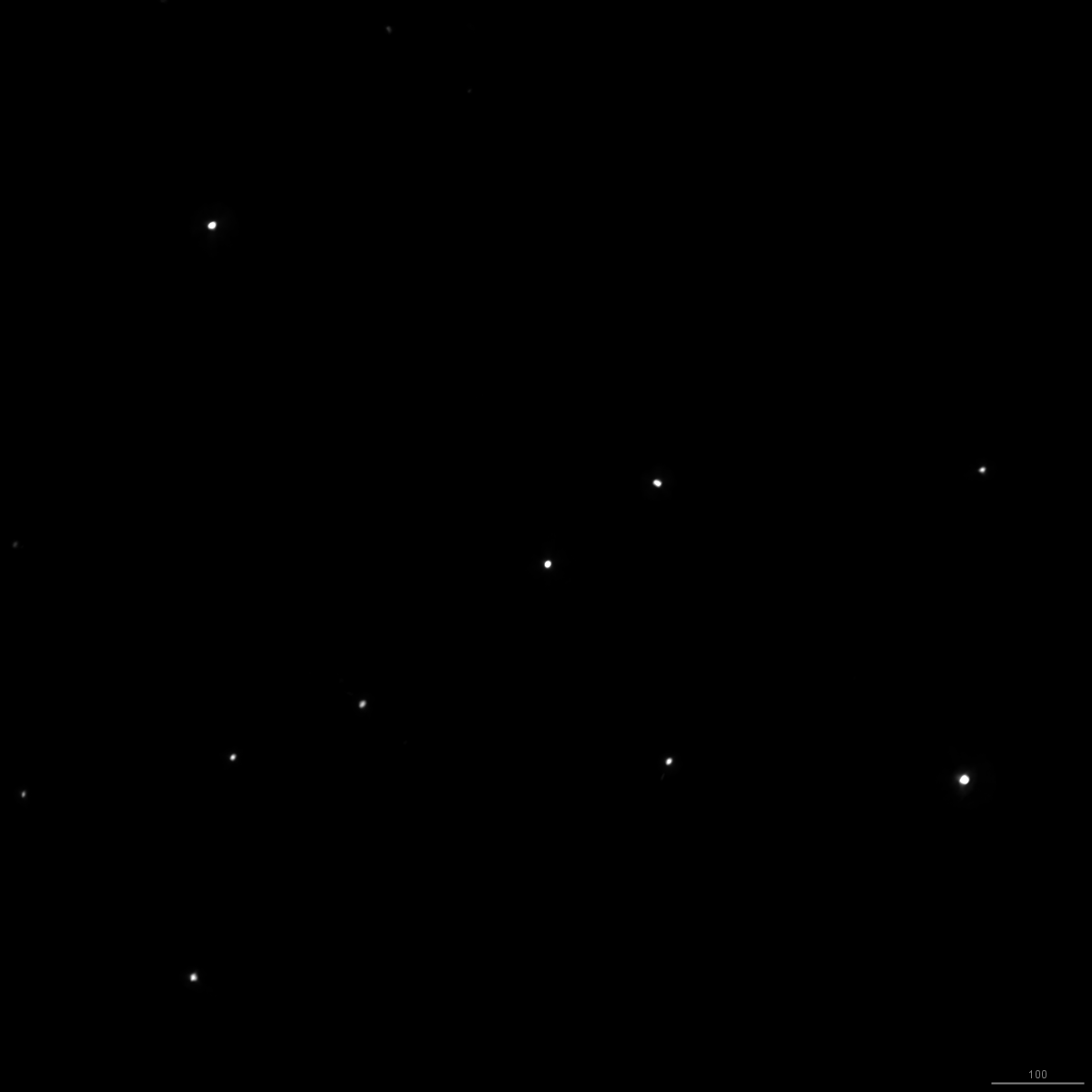

Supplement: Supplementary file 10 — Source Data for Figure 7 [file EMBJ-42-e113647-s009.zip › Figure 7/Figure 7D/PRC1 WT/Prc1.tif]

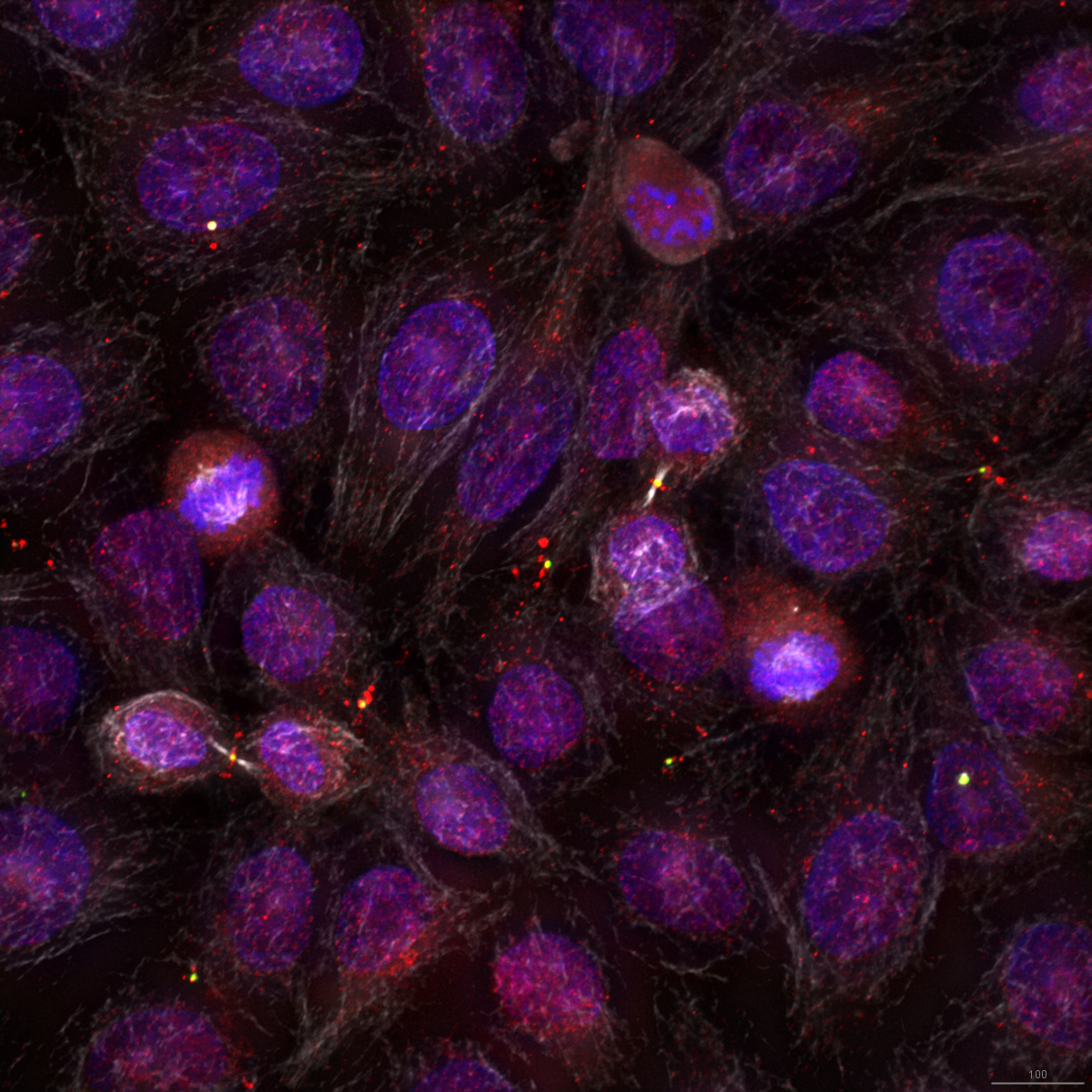

Supplement: Supplementary file 10 — Source Data for Figure 7 [file EMBJ-42-e113647-s009.zip › Figure 7/Figure 7D/PRC1 WT/merge.tif]

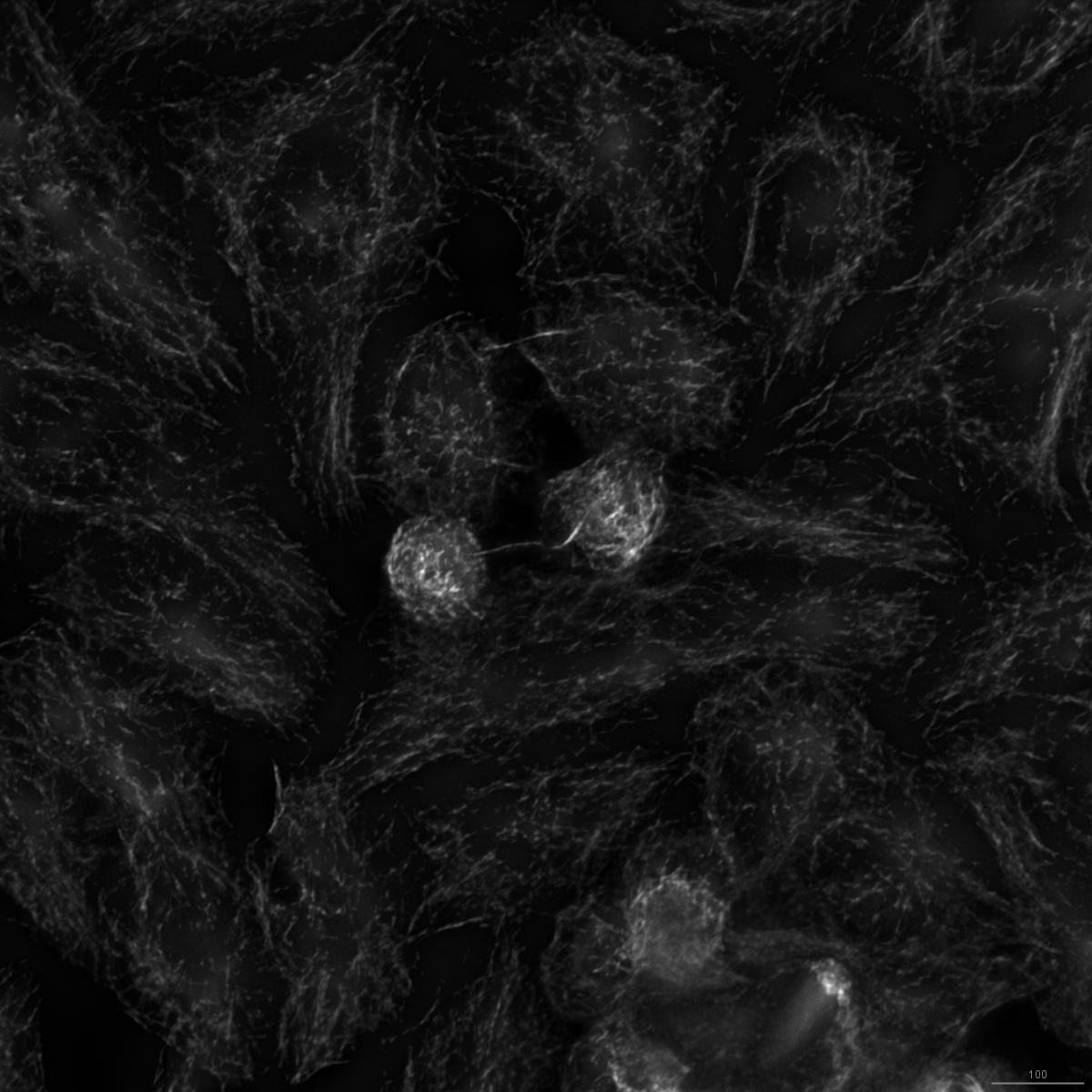

Supplement: Supplementary file 10 — Source Data for Figure 7 [file EMBJ-42-e113647-s009.zip › Figure 7/Figure 7D/PRC1 MEE /MT.tif]

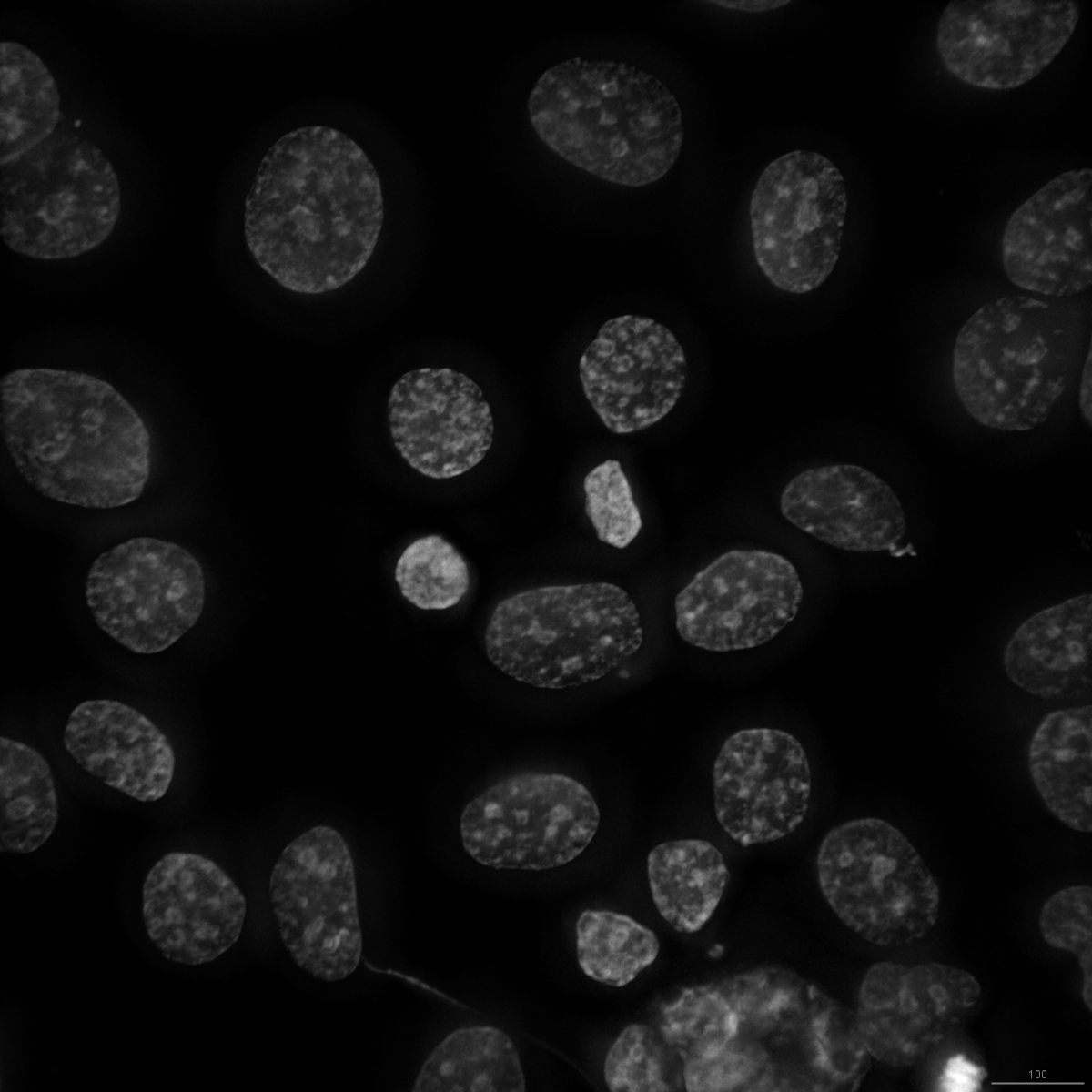

Supplement: Supplementary file 10 — Source Data for Figure 7 [file EMBJ-42-e113647-s009.zip › Figure 7/Figure 7D/PRC1 MEE /DNA.tif]

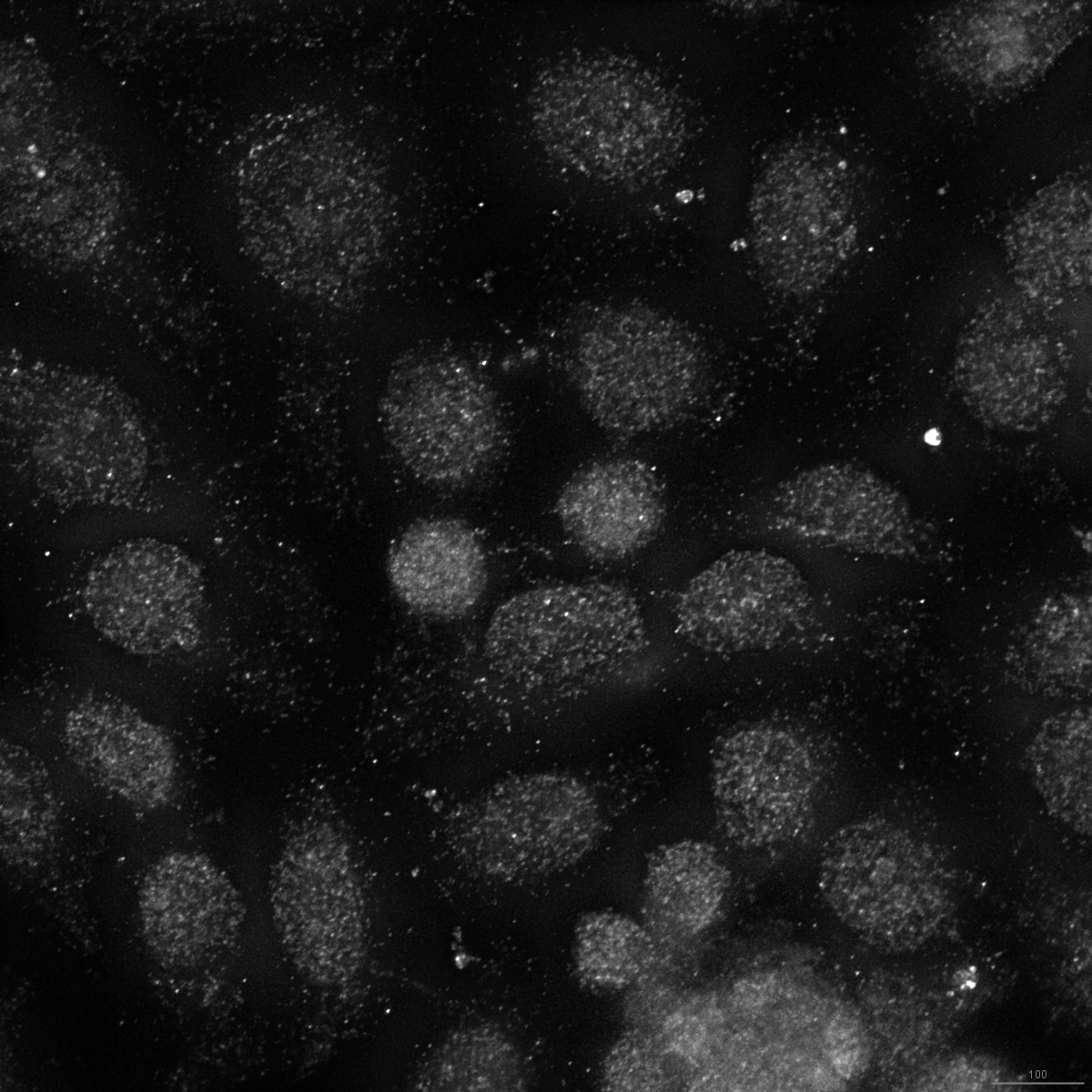

Supplement: Supplementary file 10 — Source Data for Figure 7 [file EMBJ-42-e113647-s009.zip › Figure 7/Figure 7D/PRC1 MEE /Kif4.tif]

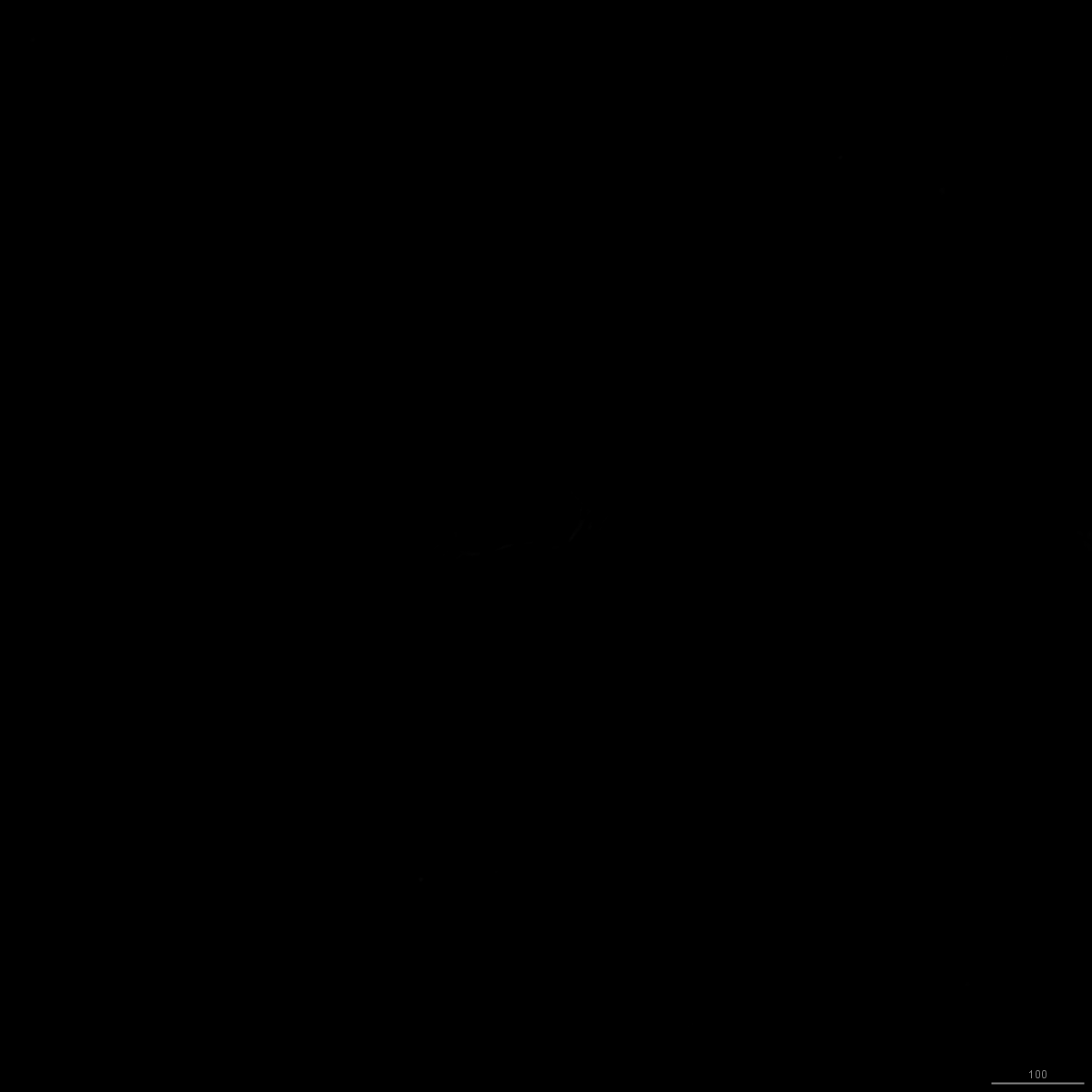

Supplement: Supplementary file 10 — Source Data for Figure 7 [file EMBJ-42-e113647-s009.zip › Figure 7/Figure 7D/PRC1 MEE /Prc1.tif]

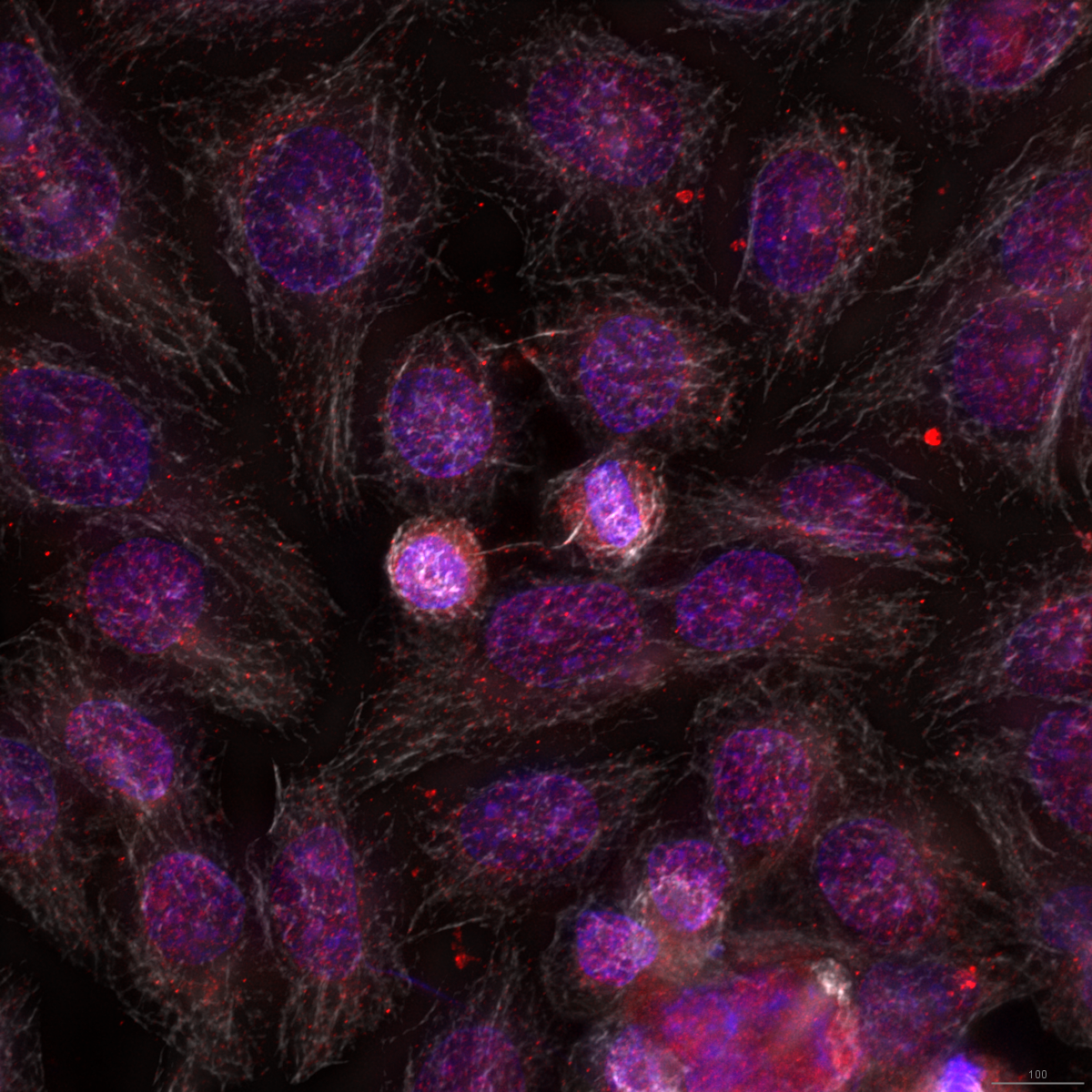

Supplement: Supplementary file 10 — Source Data for Figure 7 [file EMBJ-42-e113647-s009.zip › Figure 7/Figure 7D/PRC1 MEE /merge.tif]

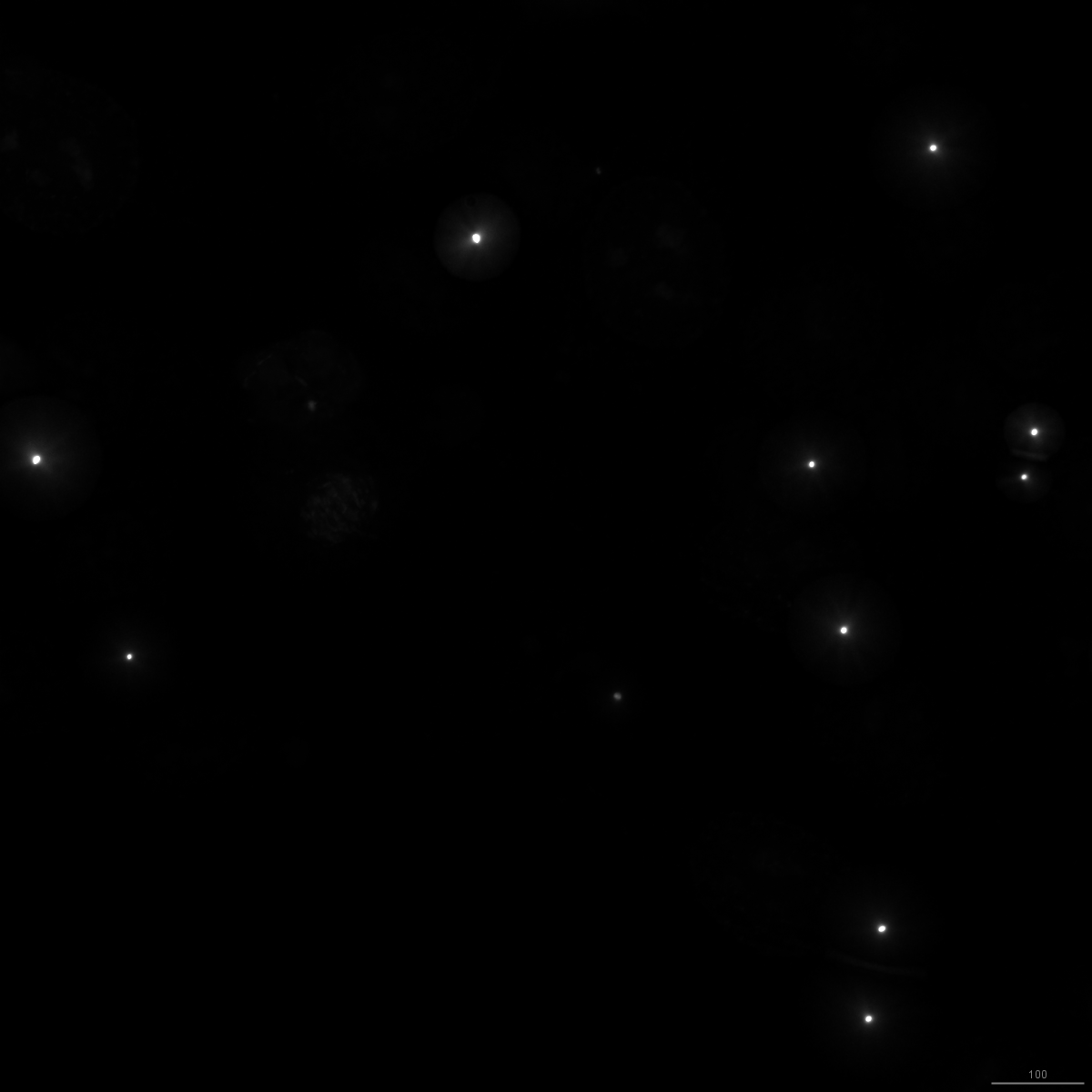

Supplement: Supplementary file 10 — Source Data for Figure 7 [file EMBJ-42-e113647-s009.zip › Figure 7/Fig 7A/PRC1 WT/telophase Prc1.tif]
