# Supplementary figures and images for: Phosphorylation controls spatial and temporal activities of motor‐PRC1 complexes to complete mitosis (part 2 of 2)
Source: EMBO J. 2023 Aug 18;42(21):e113647. doi: 10.15252/embj.2023113647 (PMC10620760; doi:10.15252/embj.2023113647)

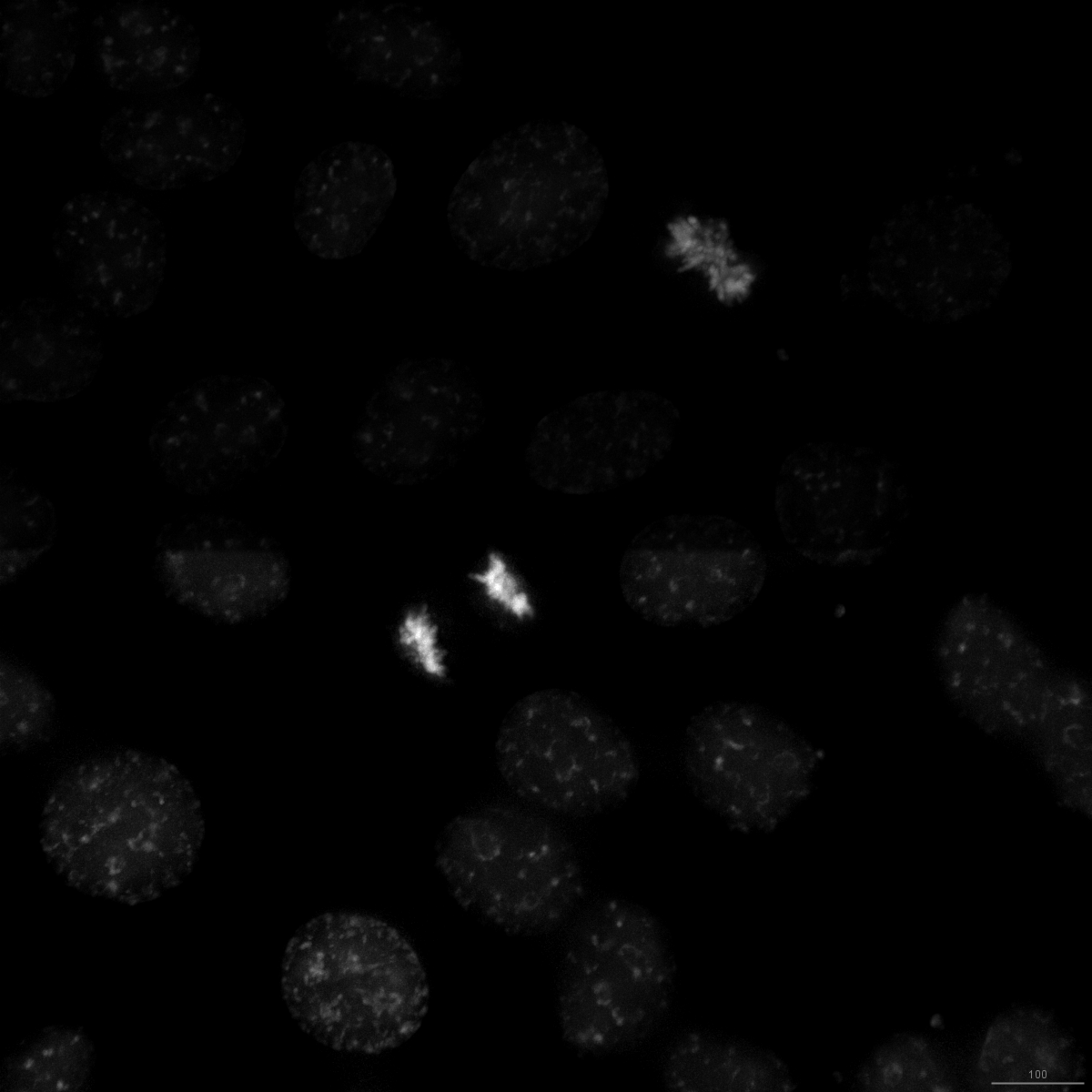

Supplement: Supplementary file 10 — Source Data for Figure 7 [file EMBJ-42-e113647-s009.zip › Figure 7/Fig 7A/PRC1 WT/anaphaseDNA.tif]

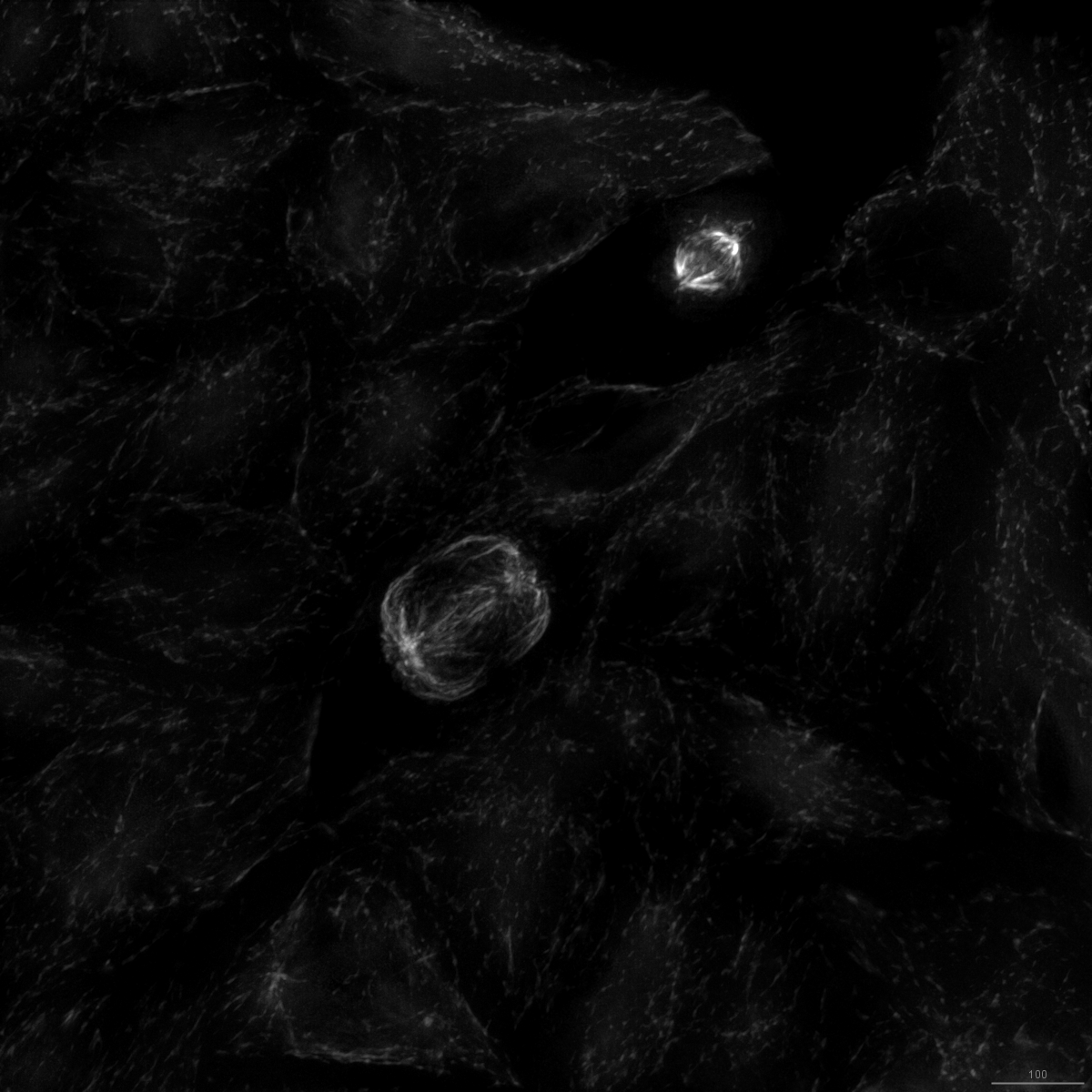

Supplement: Supplementary file 10 — Source Data for Figure 7 [file EMBJ-42-e113647-s009.zip › Figure 7/Fig 7A/PRC1 WT/anaphaseMT.tif]

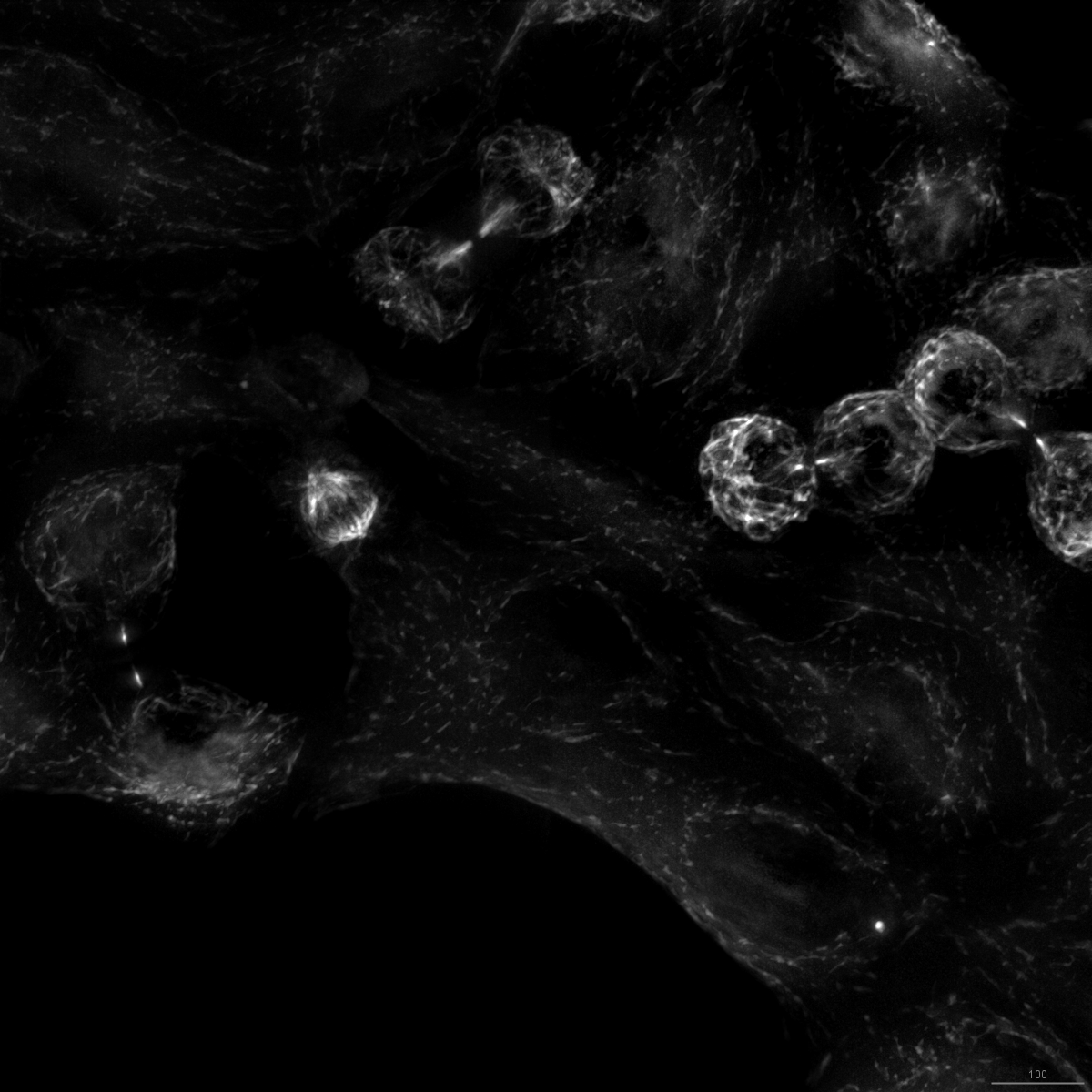

Supplement: Supplementary file 10 — Source Data for Figure 7 [file EMBJ-42-e113647-s009.zip › Figure 7/Fig 7A/PRC1 WT/telophase MT.tif]

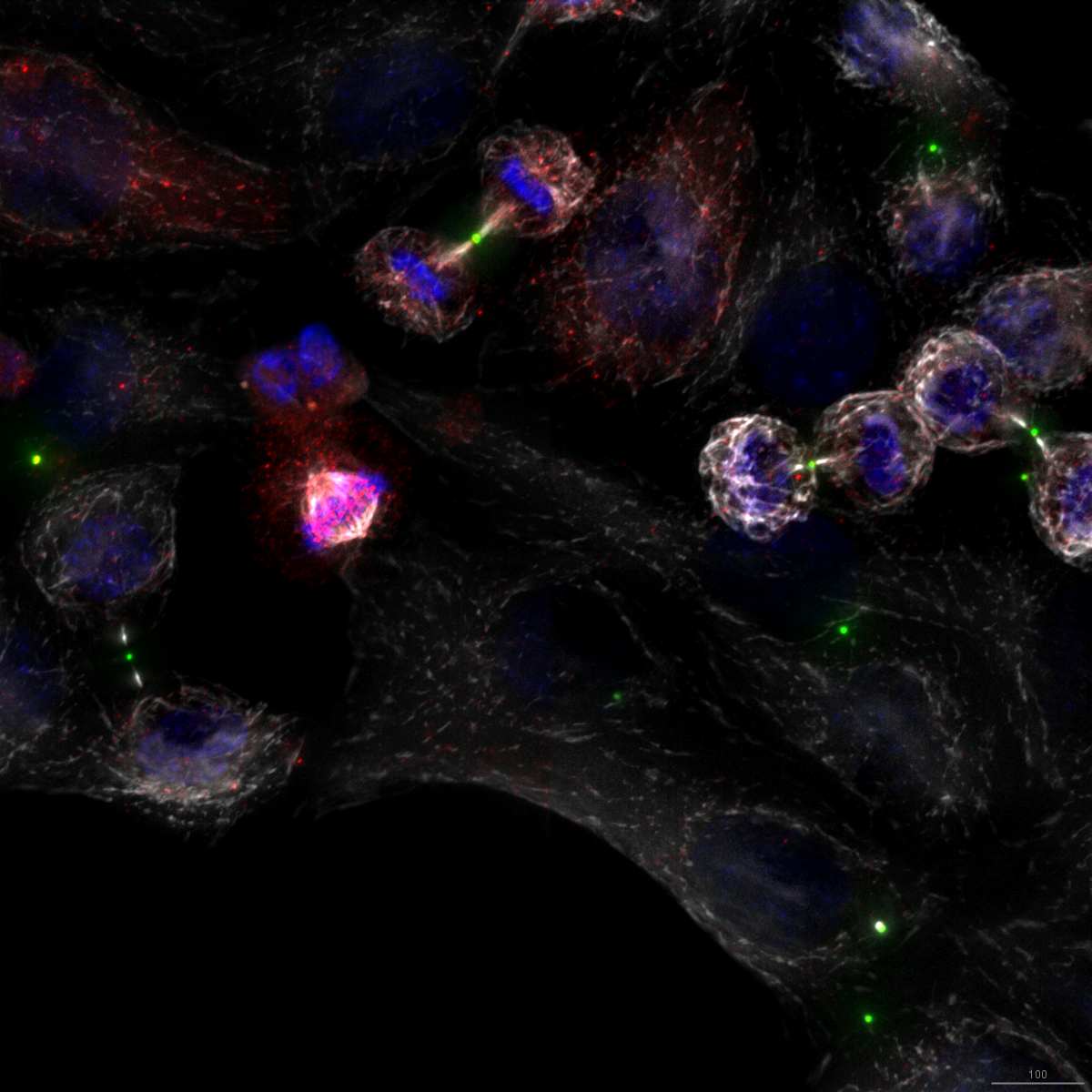

Supplement: Supplementary file 10 — Source Data for Figure 7 [file EMBJ-42-e113647-s009.zip › Figure 7/Fig 7A/PRC1 WT/telophase merge.tif]

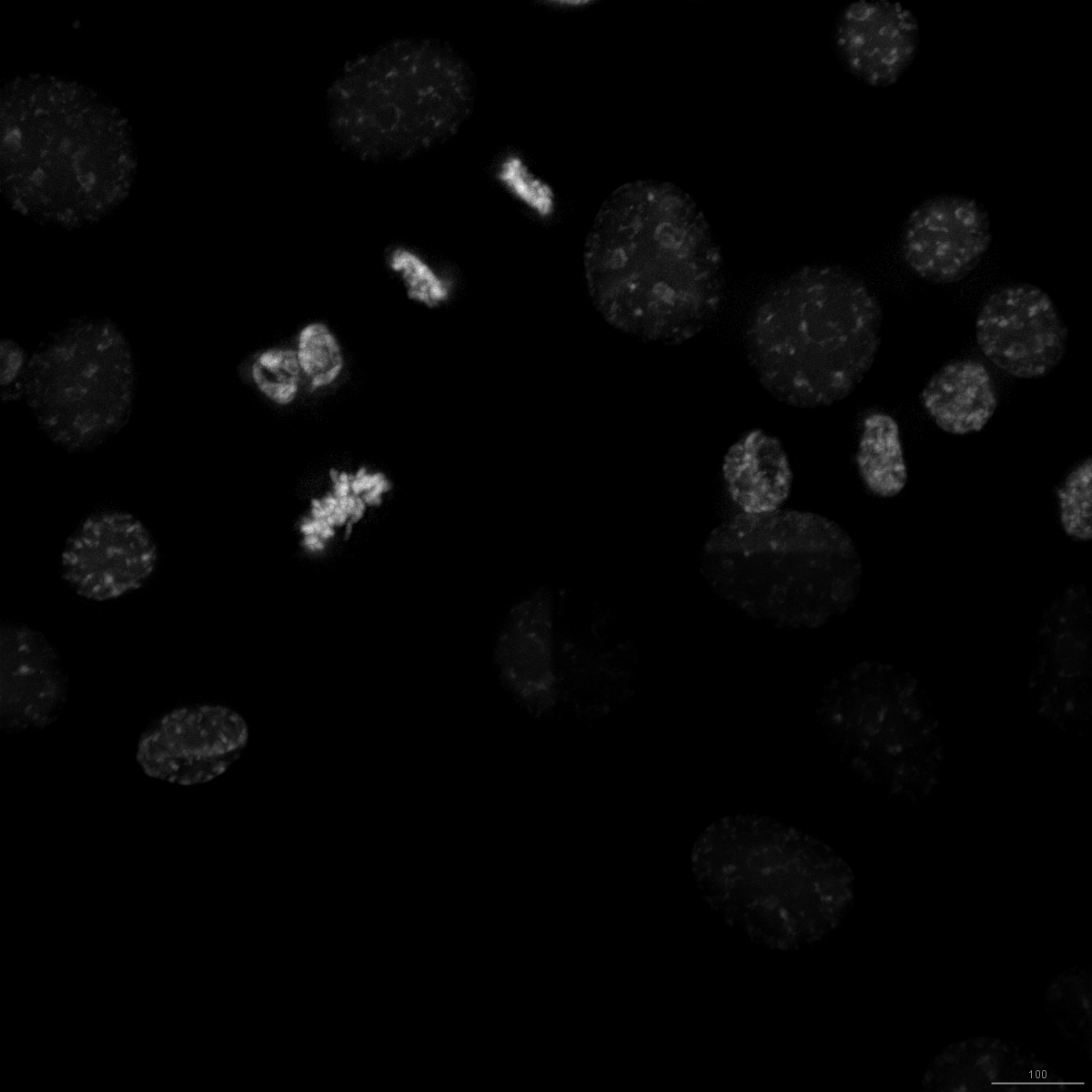

Supplement: Supplementary file 10 — Source Data for Figure 7 [file EMBJ-42-e113647-s009.zip › Figure 7/Fig 7A/PRC1 WT/telophase DNa.tif]

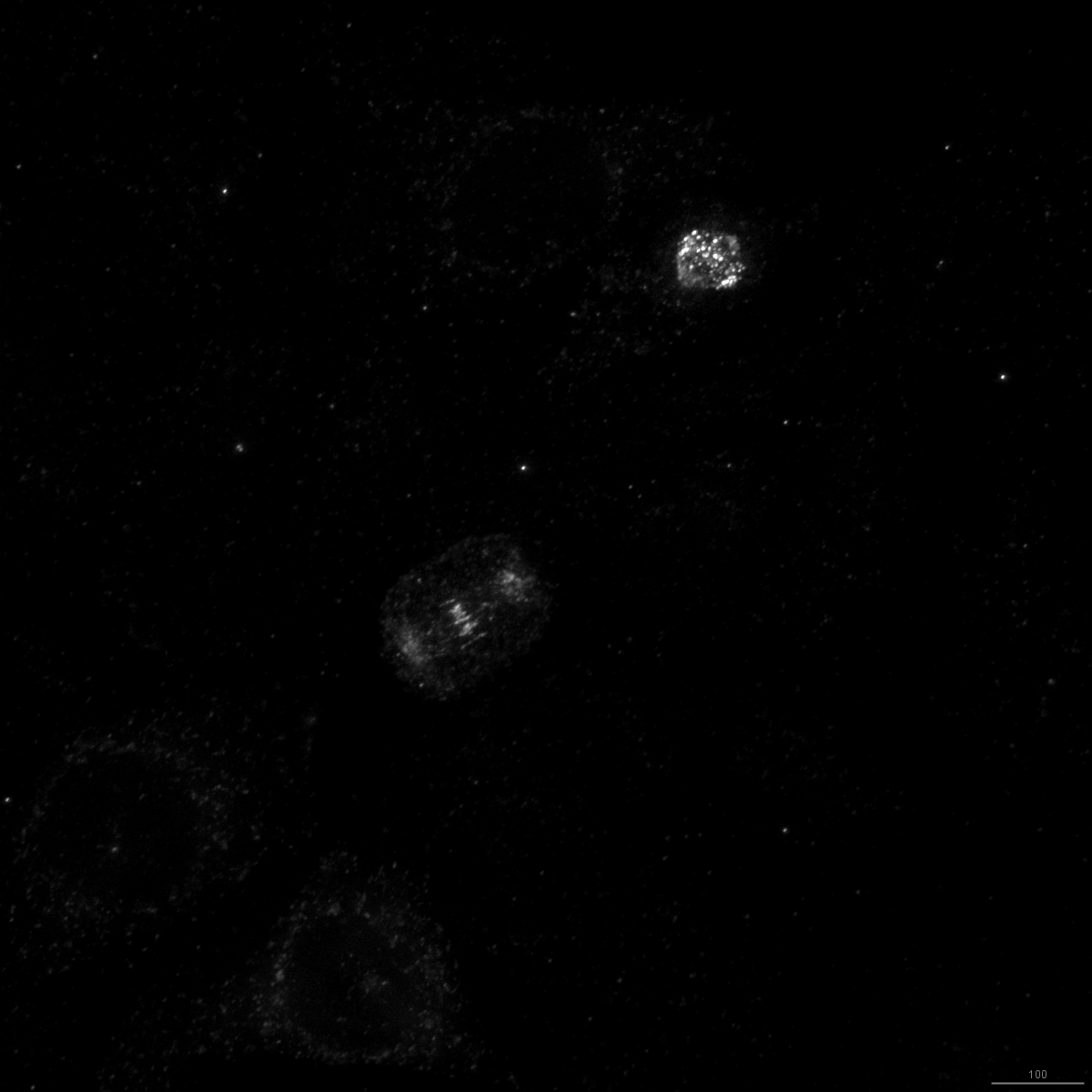

Supplement: Supplementary file 10 — Source Data for Figure 7 [file EMBJ-42-e113647-s009.zip › Figure 7/Fig 7A/PRC1 WT/anaphaseCenpE.tif]

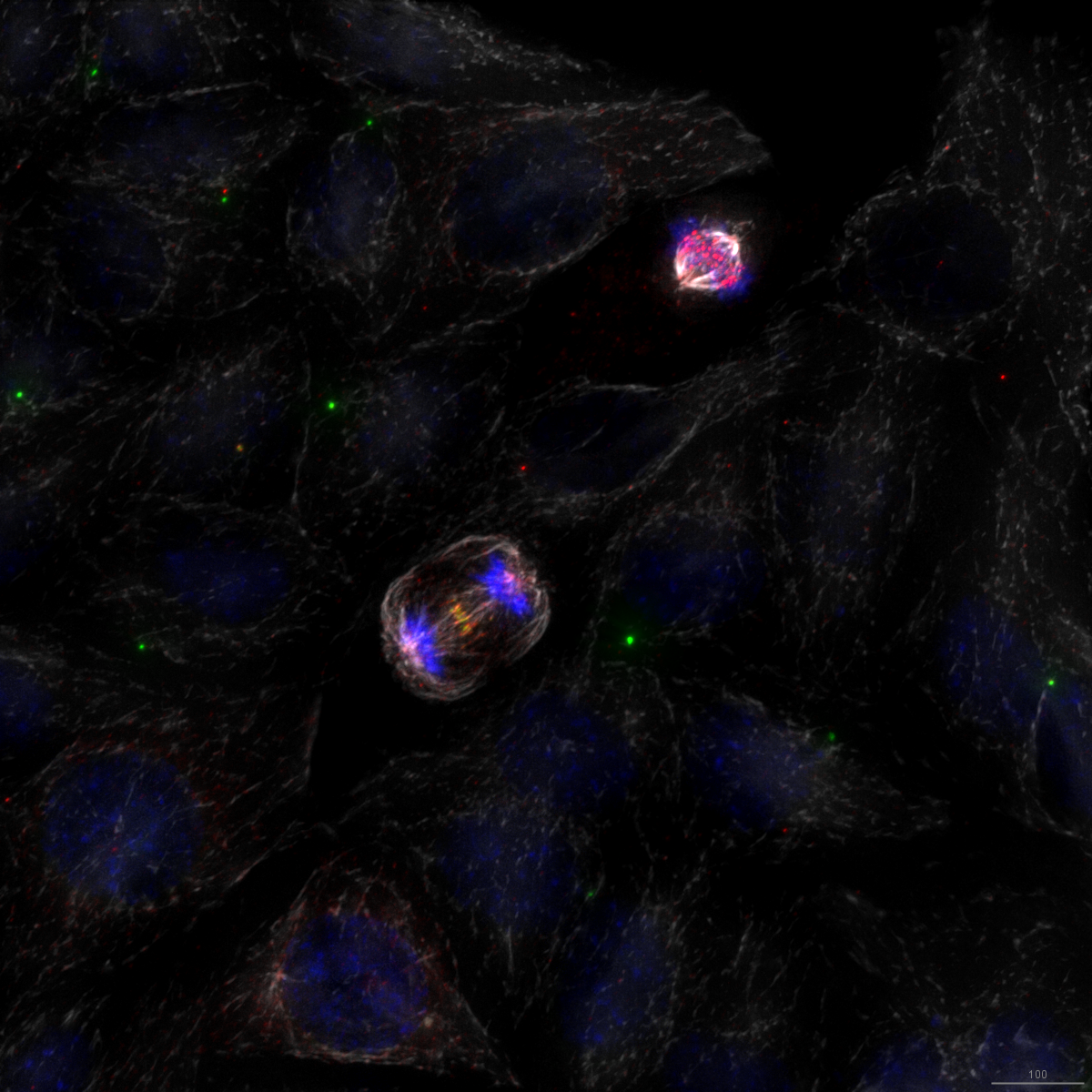

Supplement: Supplementary file 10 — Source Data for Figure 7 [file EMBJ-42-e113647-s009.zip › Figure 7/Fig 7A/PRC1 WT/anaphase merge.tif]

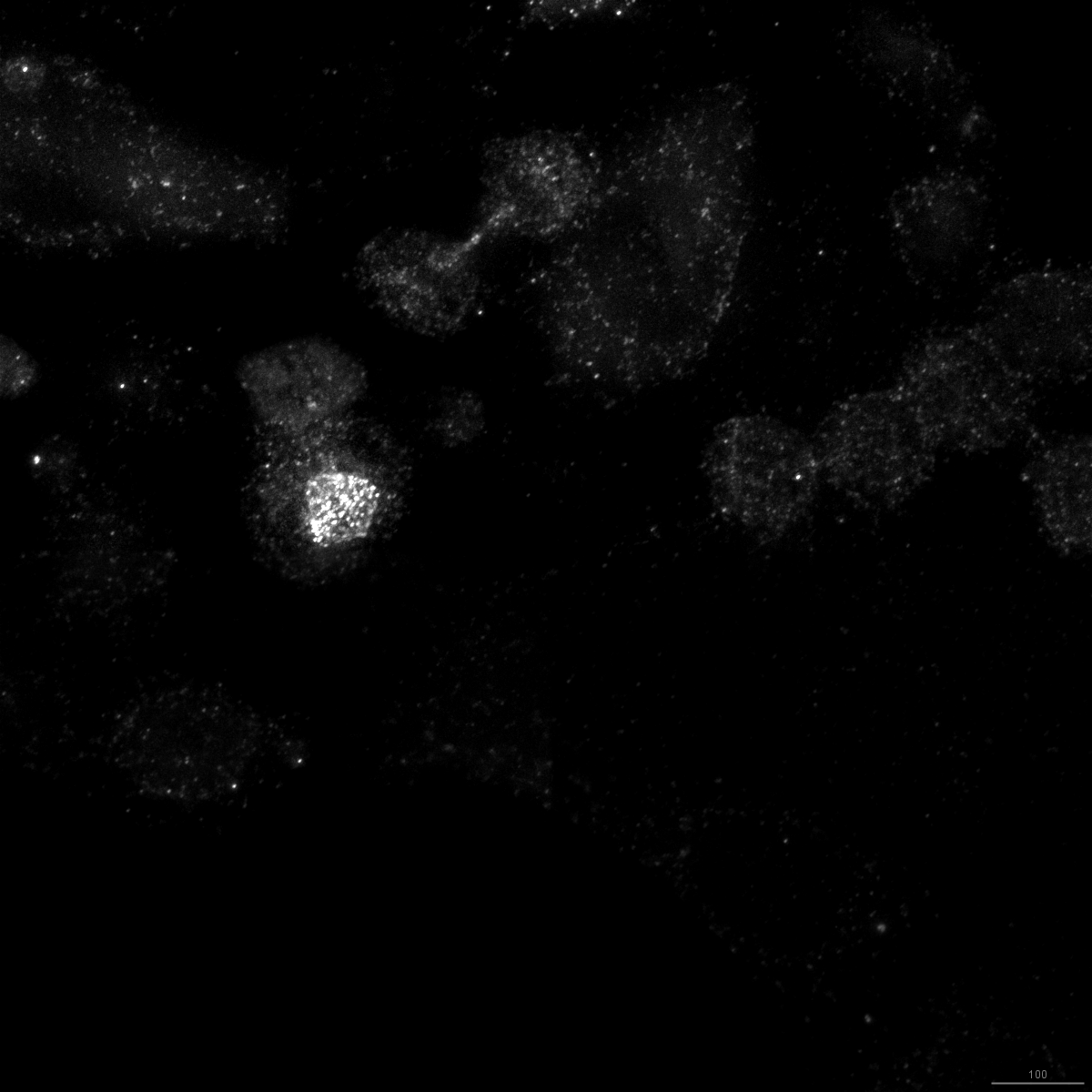

Supplement: Supplementary file 10 — Source Data for Figure 7 [file EMBJ-42-e113647-s009.zip › Figure 7/Fig 7A/PRC1 WT/telophase CenpE.tif]

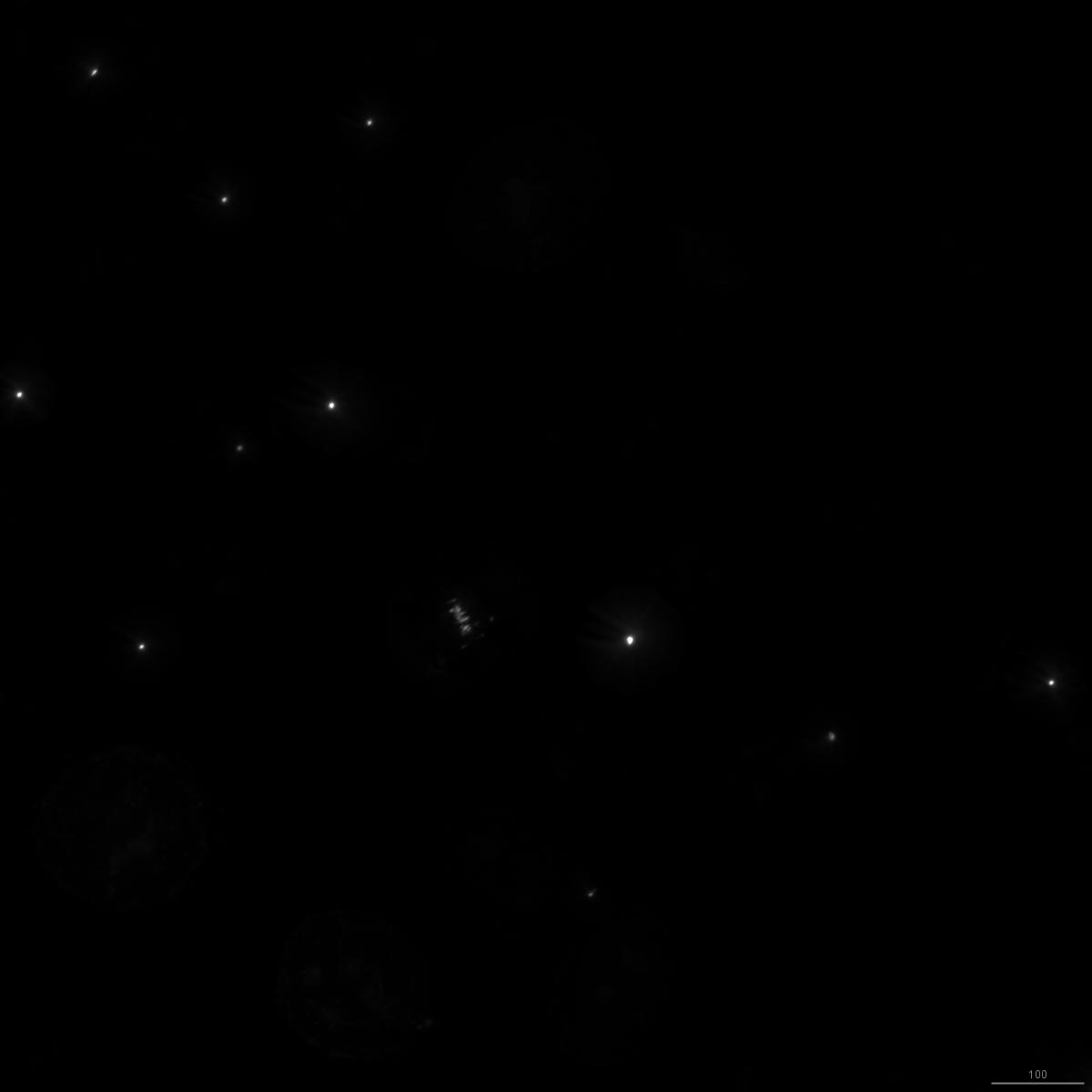

Supplement: Supplementary file 10 — Source Data for Figure 7 [file EMBJ-42-e113647-s009.zip › Figure 7/Fig 7A/PRC1 WT/anaphasePrc1.tif]

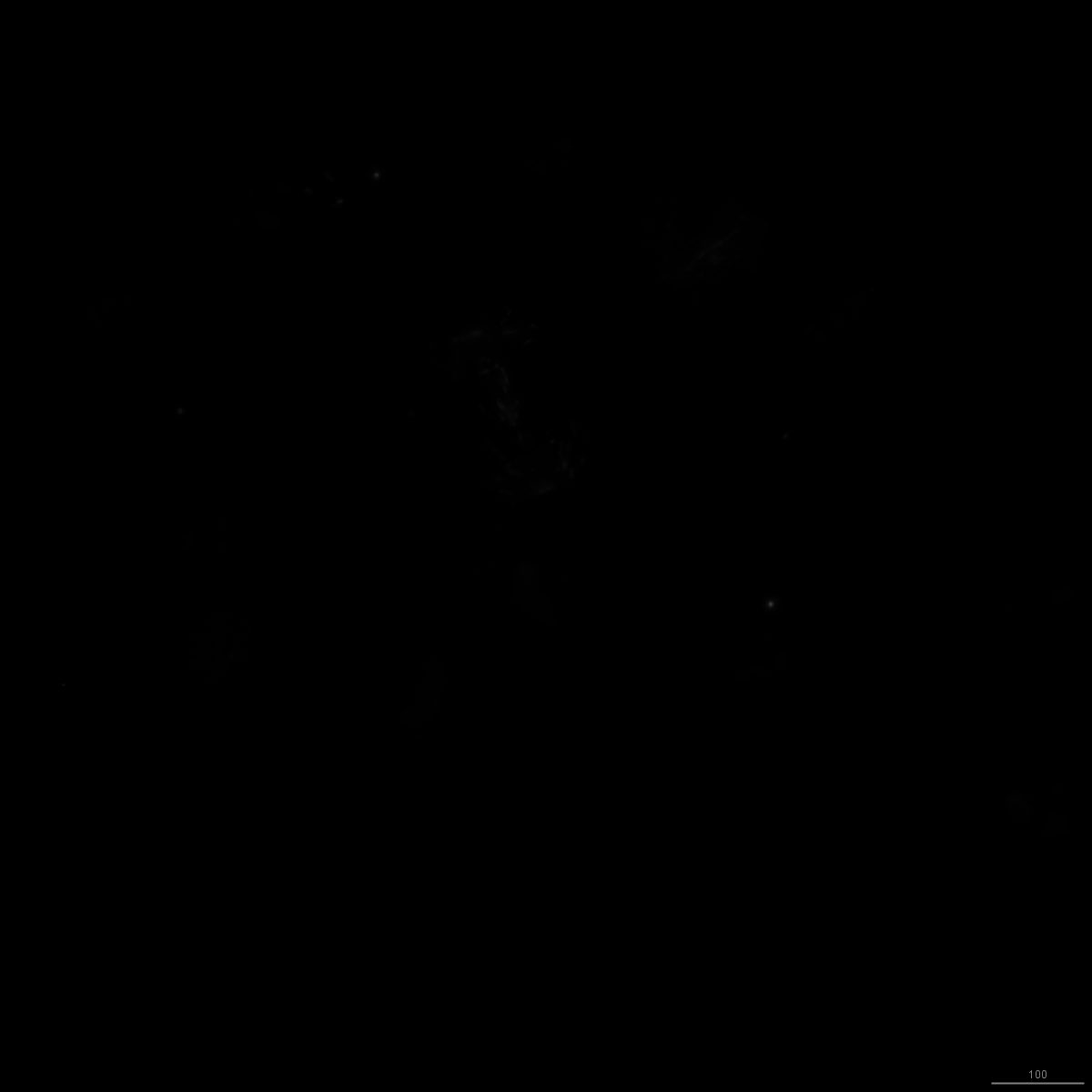

Supplement: Supplementary file 10 — Source Data for Figure 7 [file EMBJ-42-e113647-s009.zip › Figure 7/Fig 7A/PRC1 MEE/telophase PRc1.tif]

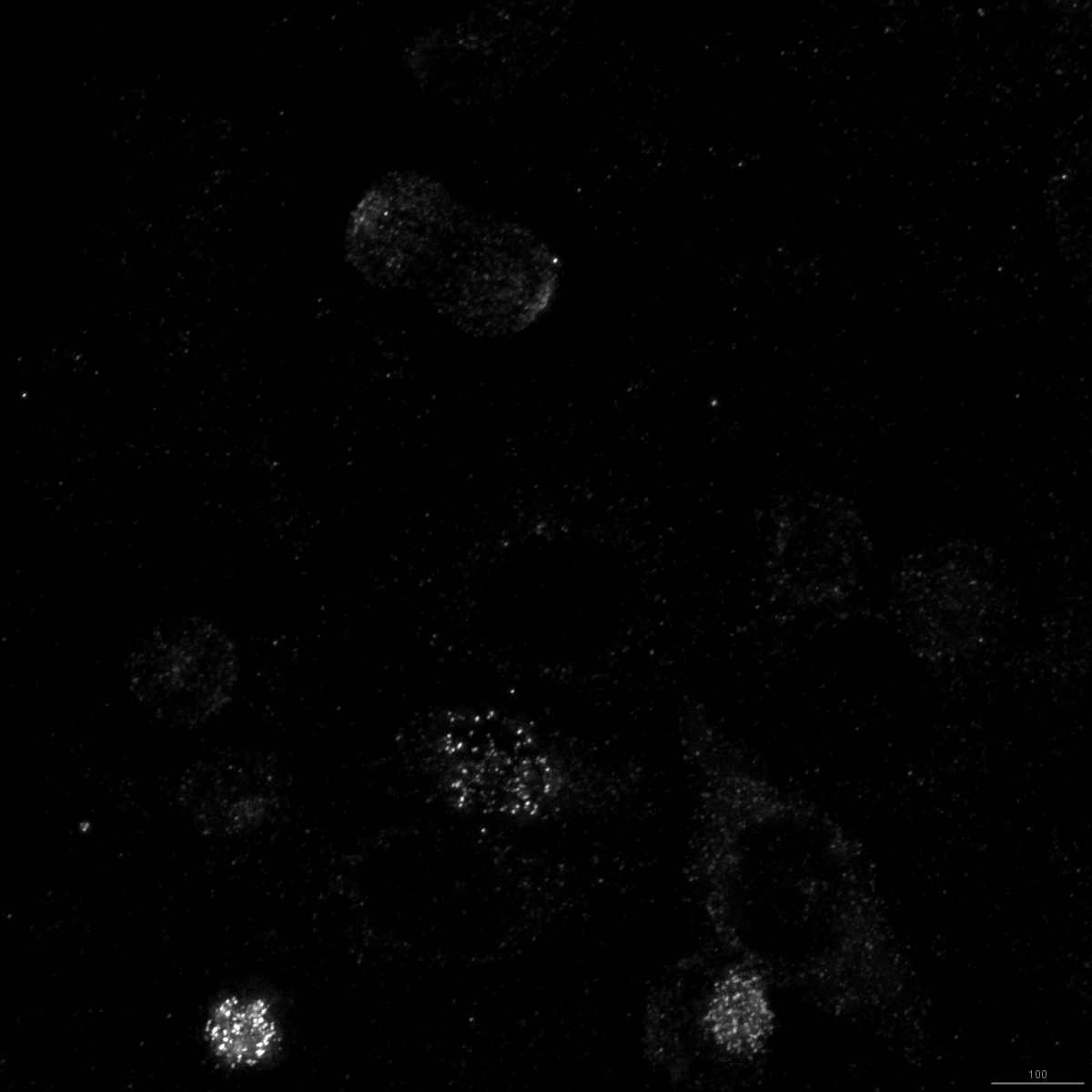

Supplement: Supplementary file 10 — Source Data for Figure 7 [file EMBJ-42-e113647-s009.zip › Figure 7/Fig 7A/PRC1 MEE/anaphase CenpE.tif]

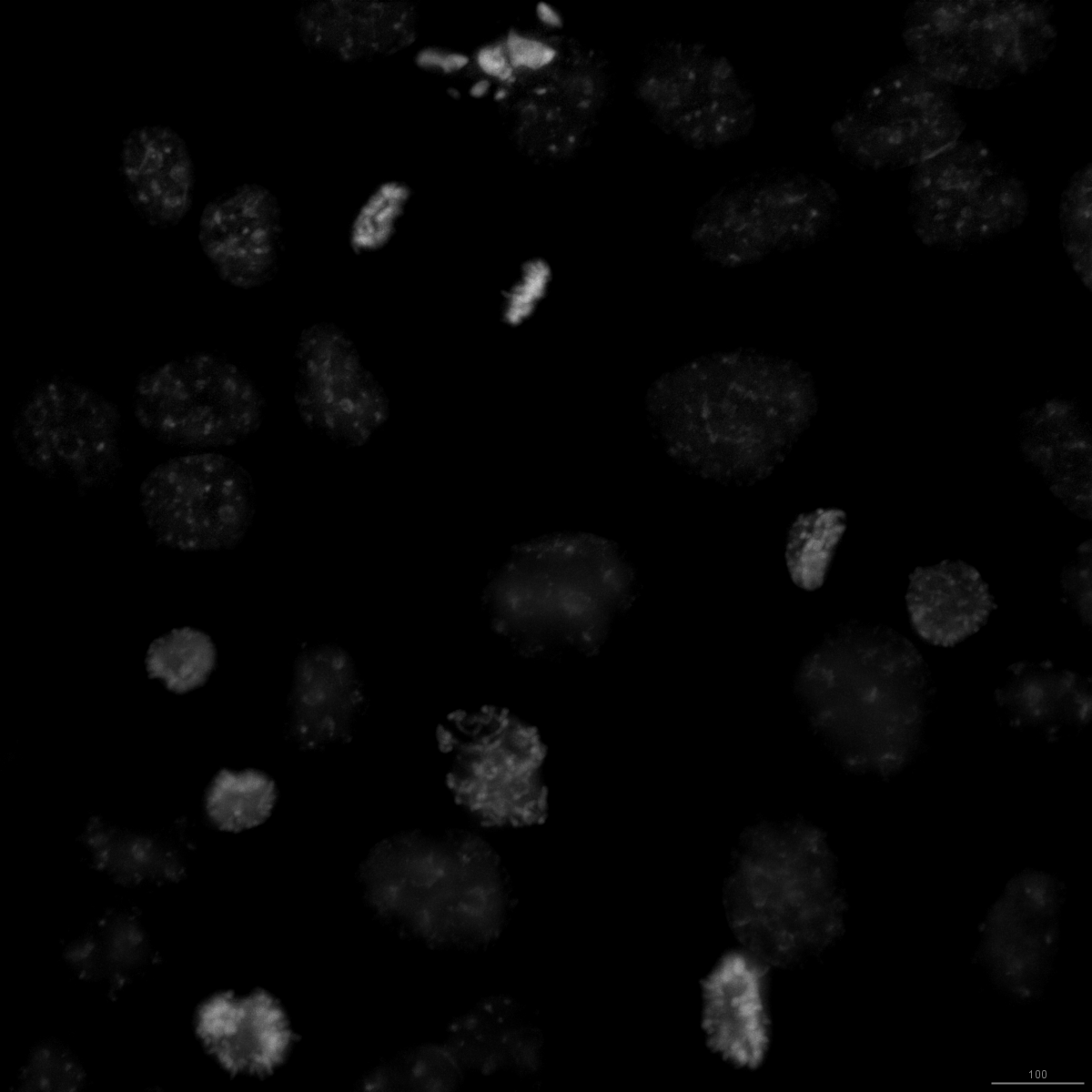

Supplement: Supplementary file 10 — Source Data for Figure 7 [file EMBJ-42-e113647-s009.zip › Figure 7/Fig 7A/PRC1 MEE/ anaphaseDNA.tif]

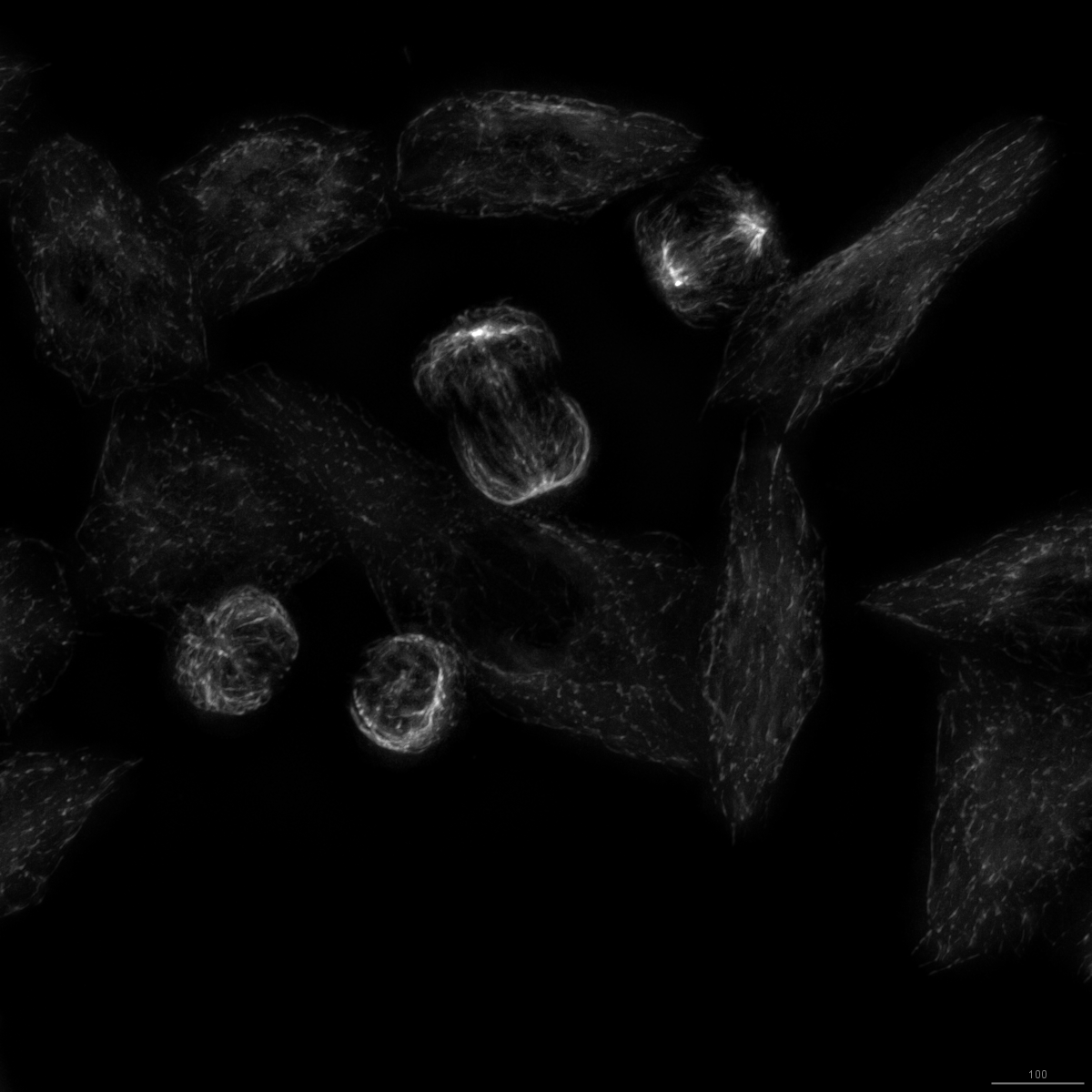

Supplement: Supplementary file 10 — Source Data for Figure 7 [file EMBJ-42-e113647-s009.zip › Figure 7/Fig 7A/PRC1 MEE/telophase MT.tif]

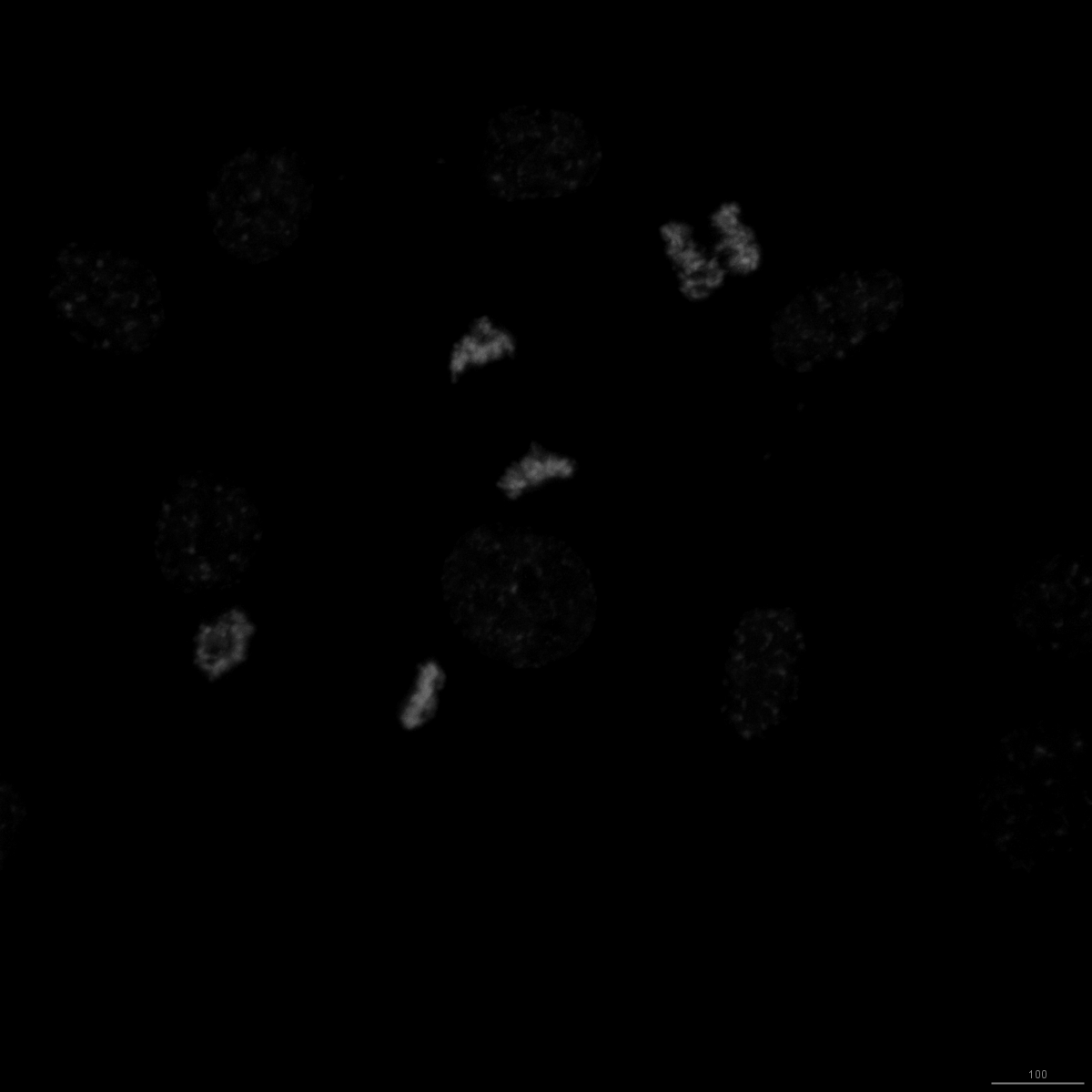

Supplement: Supplementary file 10 — Source Data for Figure 7 [file EMBJ-42-e113647-s009.zip › Figure 7/Fig 7A/PRC1 MEE/telophase DNA.tif]

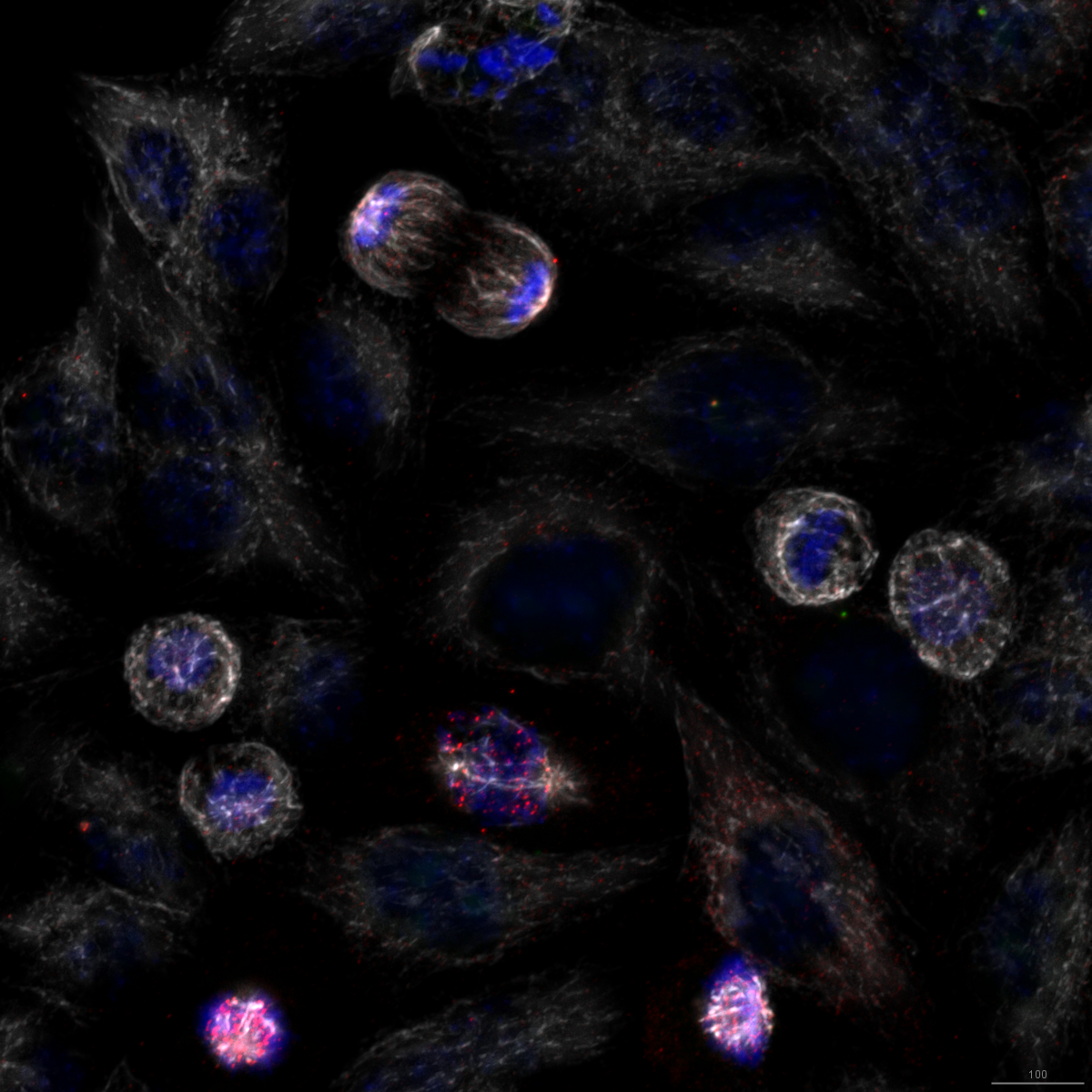

Supplement: Supplementary file 10 — Source Data for Figure 7 [file EMBJ-42-e113647-s009.zip › Figure 7/Fig 7A/PRC1 MEE/anaphase merge.tif]

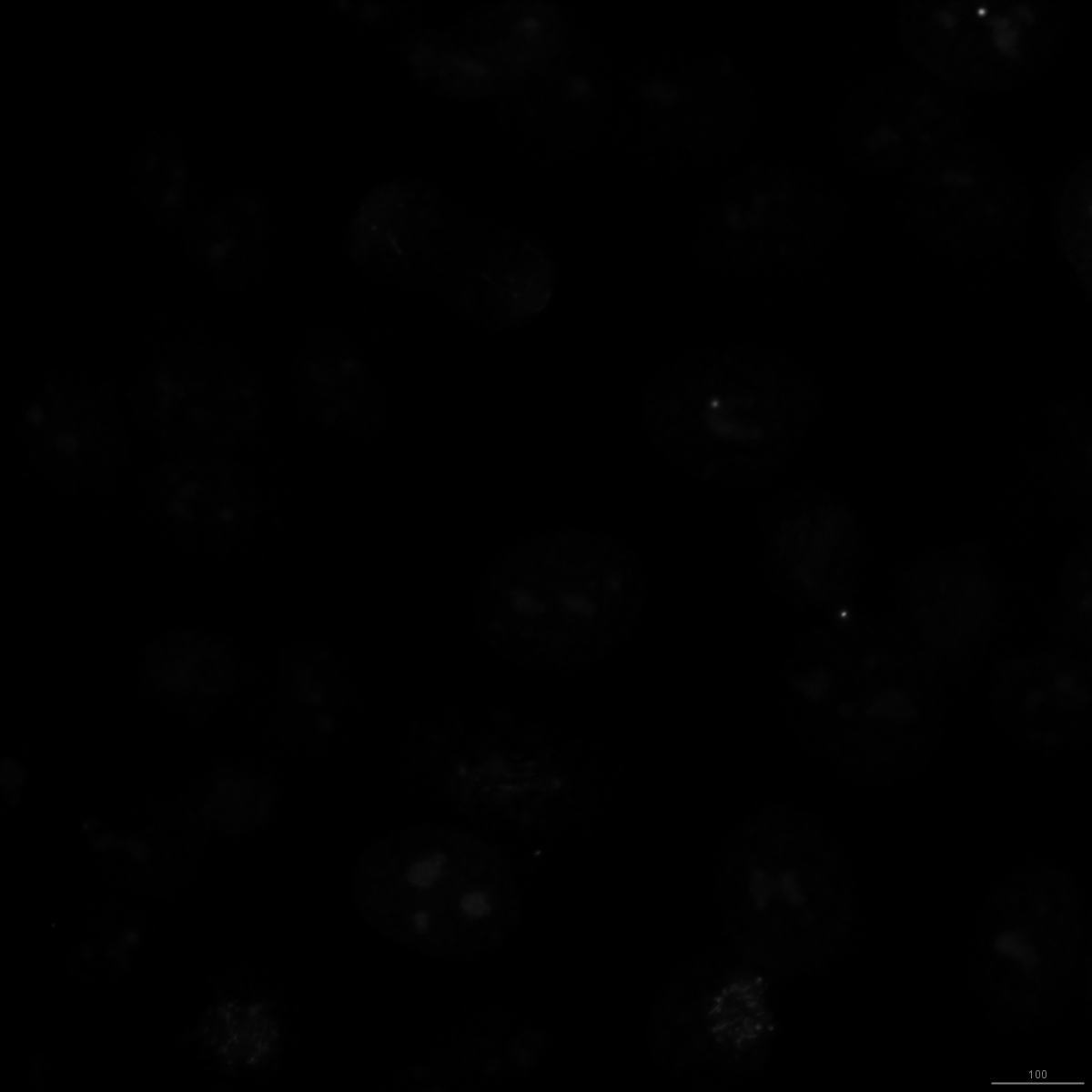

Supplement: Supplementary file 10 — Source Data for Figure 7 [file EMBJ-42-e113647-s009.zip › Figure 7/Fig 7A/PRC1 MEE/anaphasePrc1.tif]

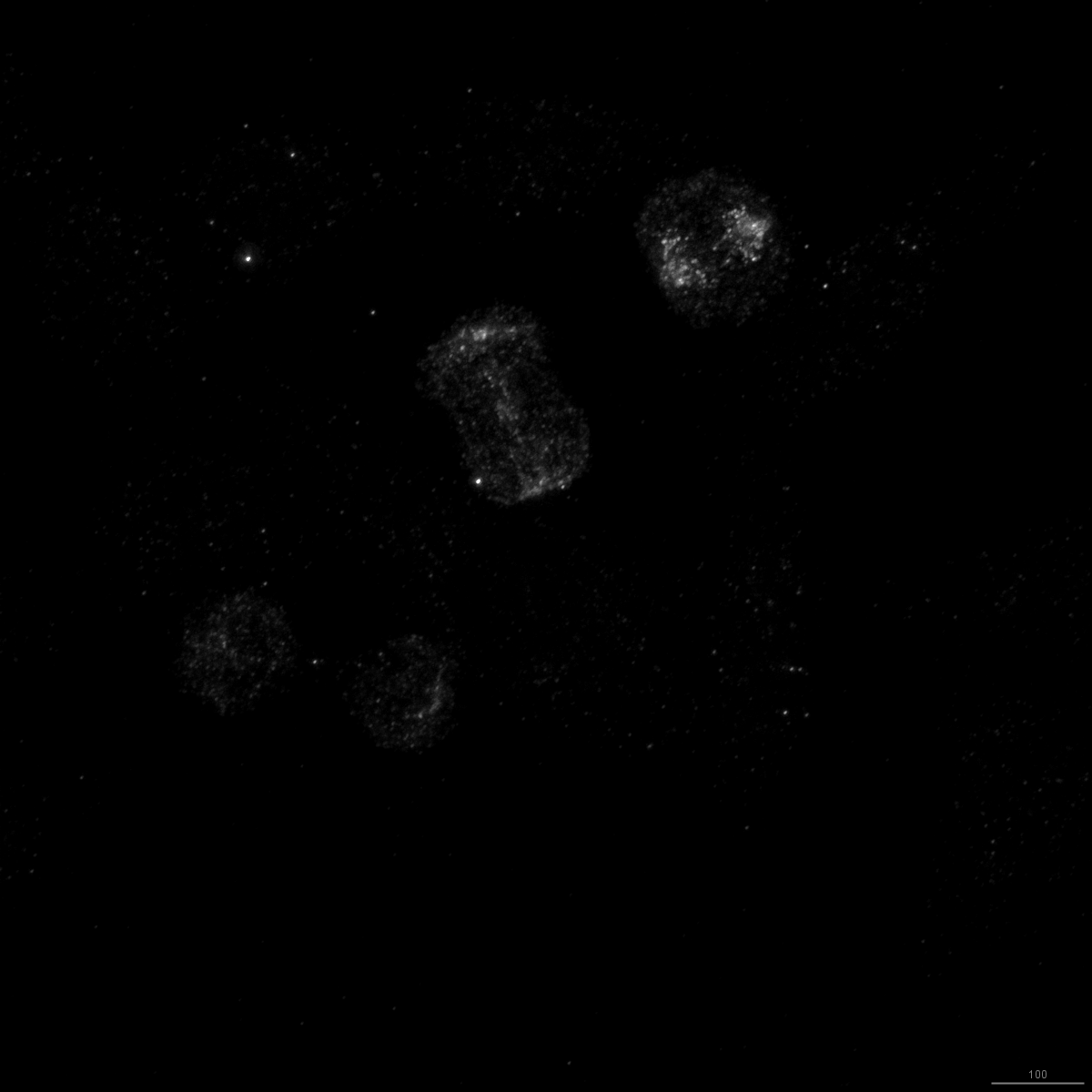

Supplement: Supplementary file 10 — Source Data for Figure 7 [file EMBJ-42-e113647-s009.zip › Figure 7/Fig 7A/PRC1 MEE/telphase CenpE.tif]

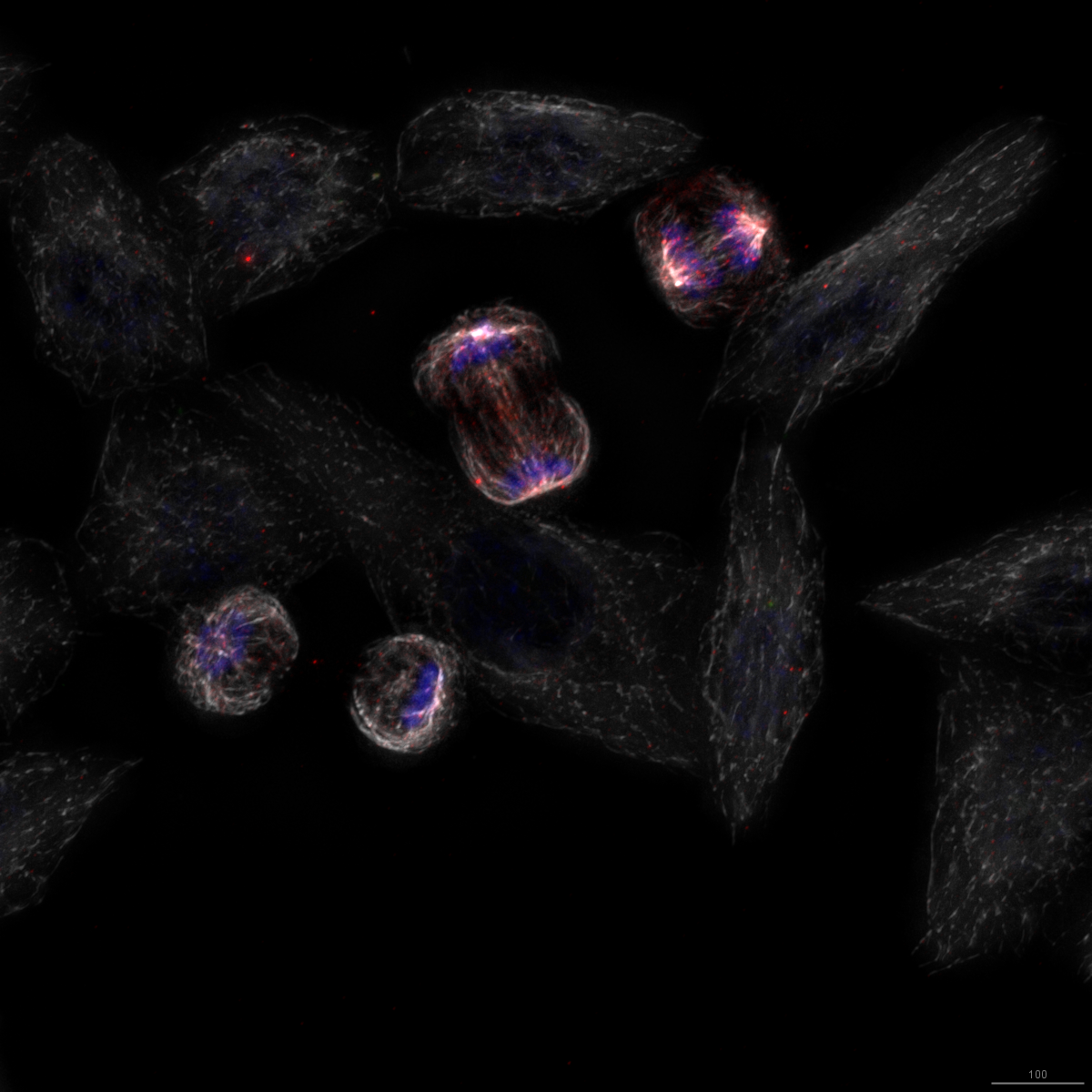

Supplement: Supplementary file 10 — Source Data for Figure 7 [file EMBJ-42-e113647-s009.zip › Figure 7/Fig 7A/PRC1 MEE/telophase merge.tif]

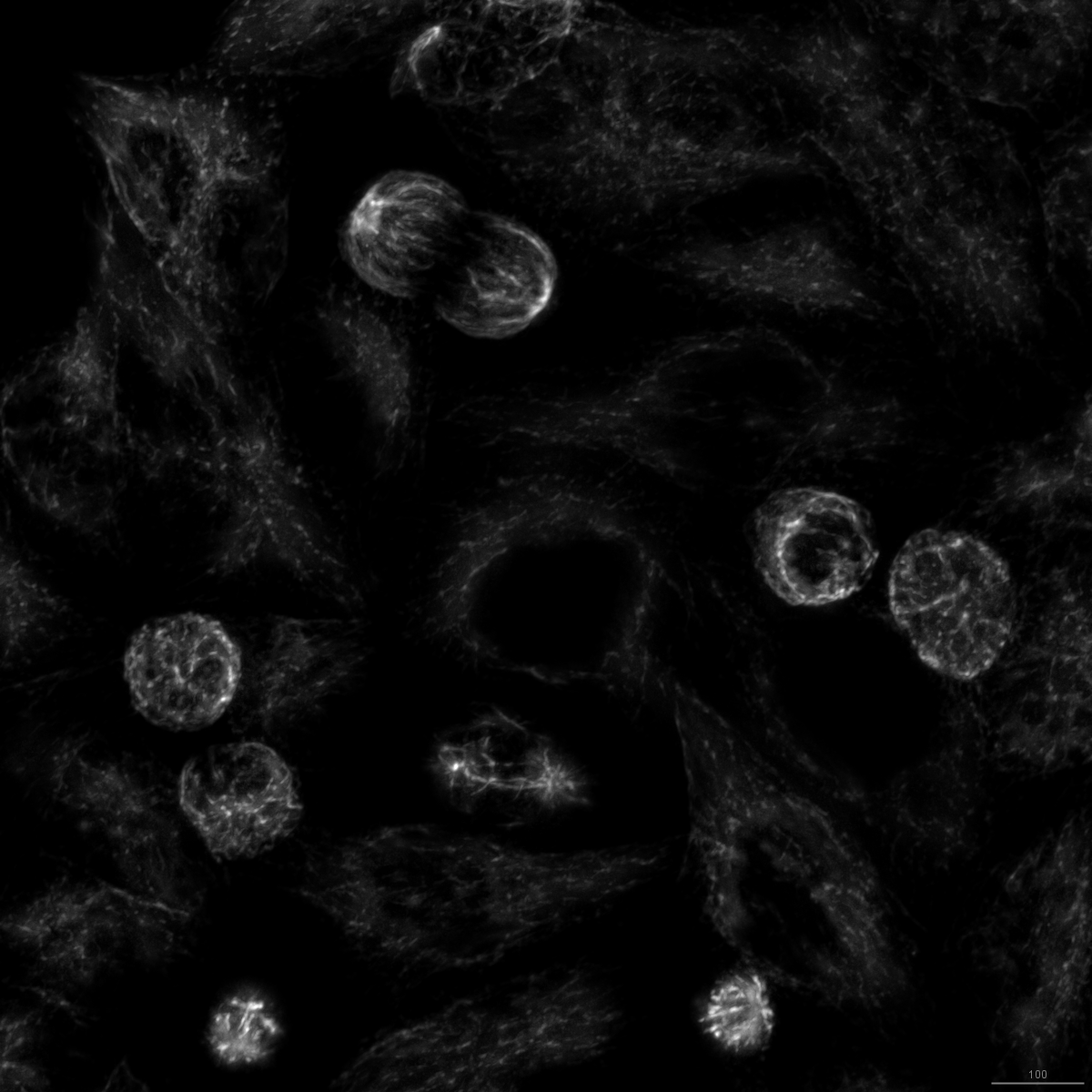

Supplement: Supplementary file 10 — Source Data for Figure 7 [file EMBJ-42-e113647-s009.zip › Figure 7/Fig 7A/PRC1 MEE/anaphase MT.tif]

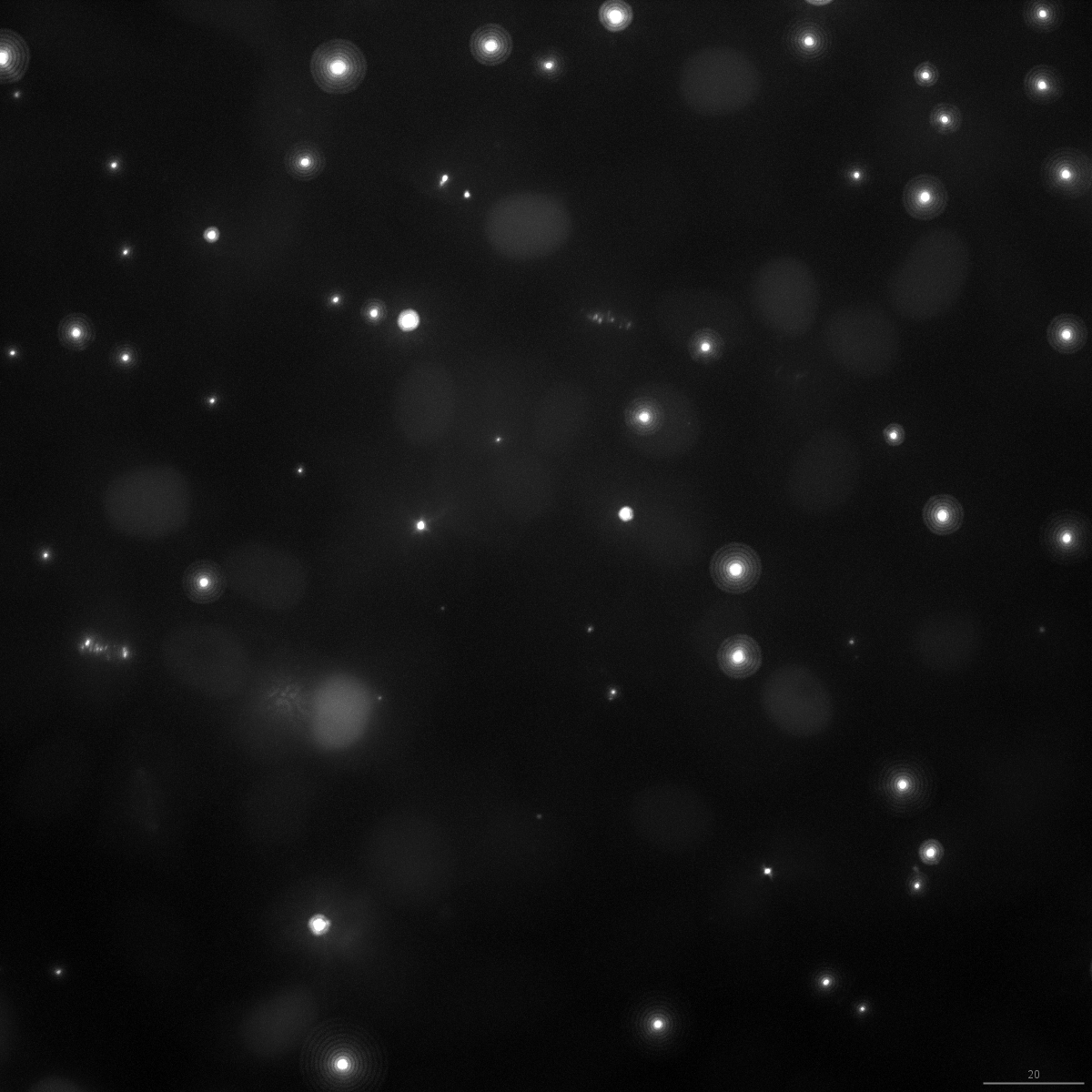

Supplement: Supplementary file 10 — Source Data for Figure 7 [file EMBJ-42-e113647-s009.zip › Figure 7/Figure 7G/uncropped PRC1/4 min/PRC1wtGFP.tif]

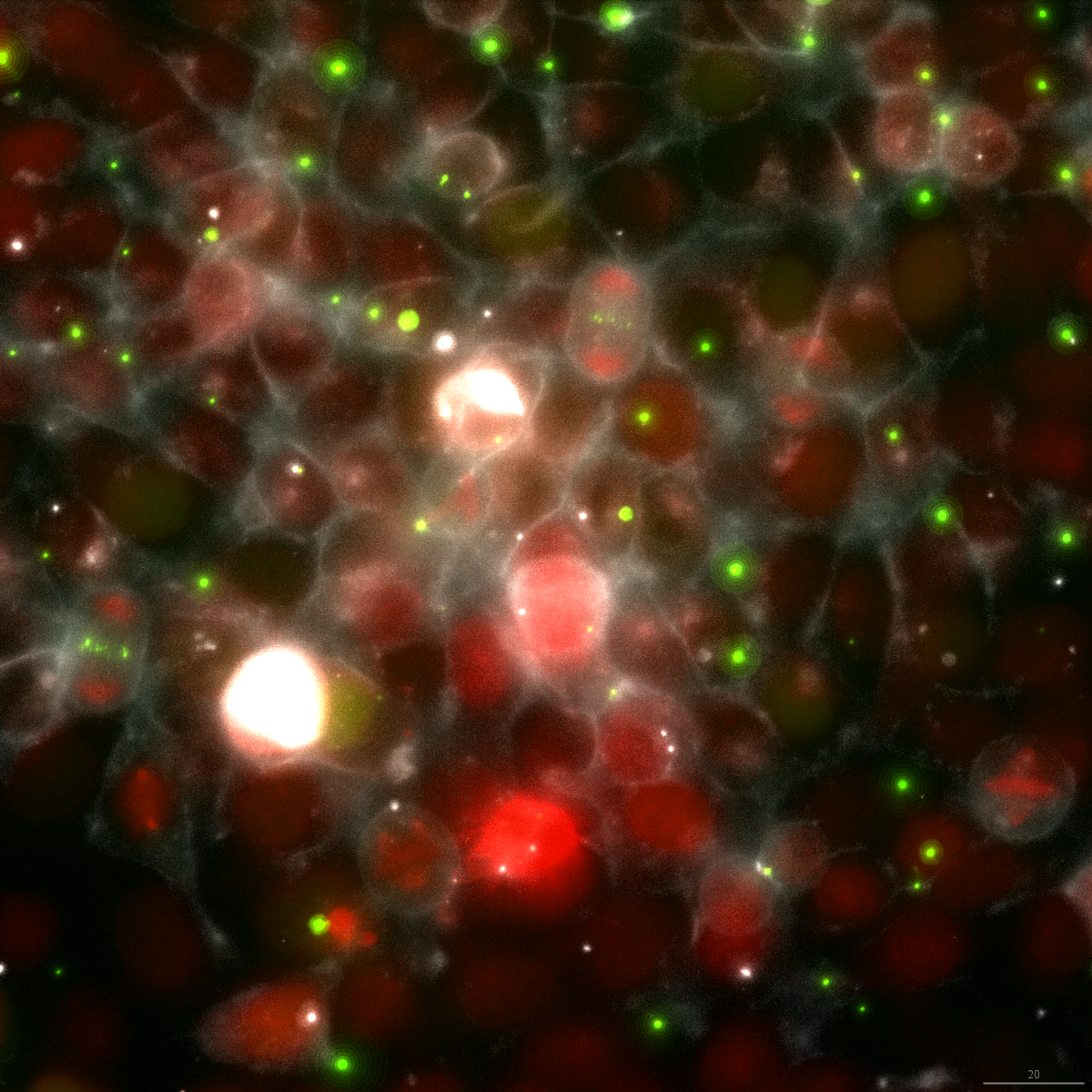

Supplement: Supplementary file 10 — Source Data for Figure 7 [file EMBJ-42-e113647-s009.zip › Figure 7/Figure 7G/uncropped PRC1/4 min/PRC1wtMerge.tif]

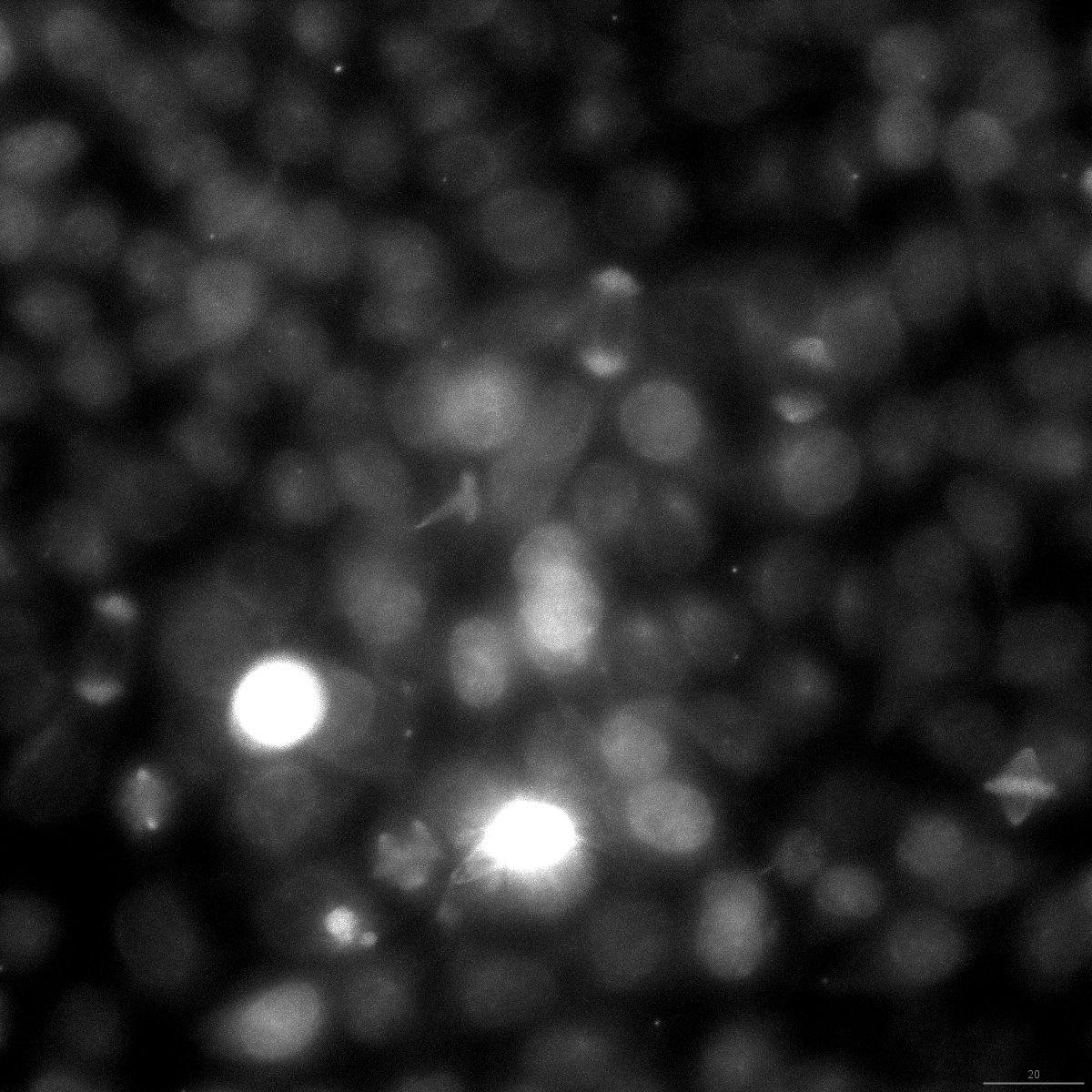

Supplement: Supplementary file 10 — Source Data for Figure 7 [file EMBJ-42-e113647-s009.zip › Figure 7/Figure 7G/uncropped PRC1/4 min/PRC1wtMT.tif]

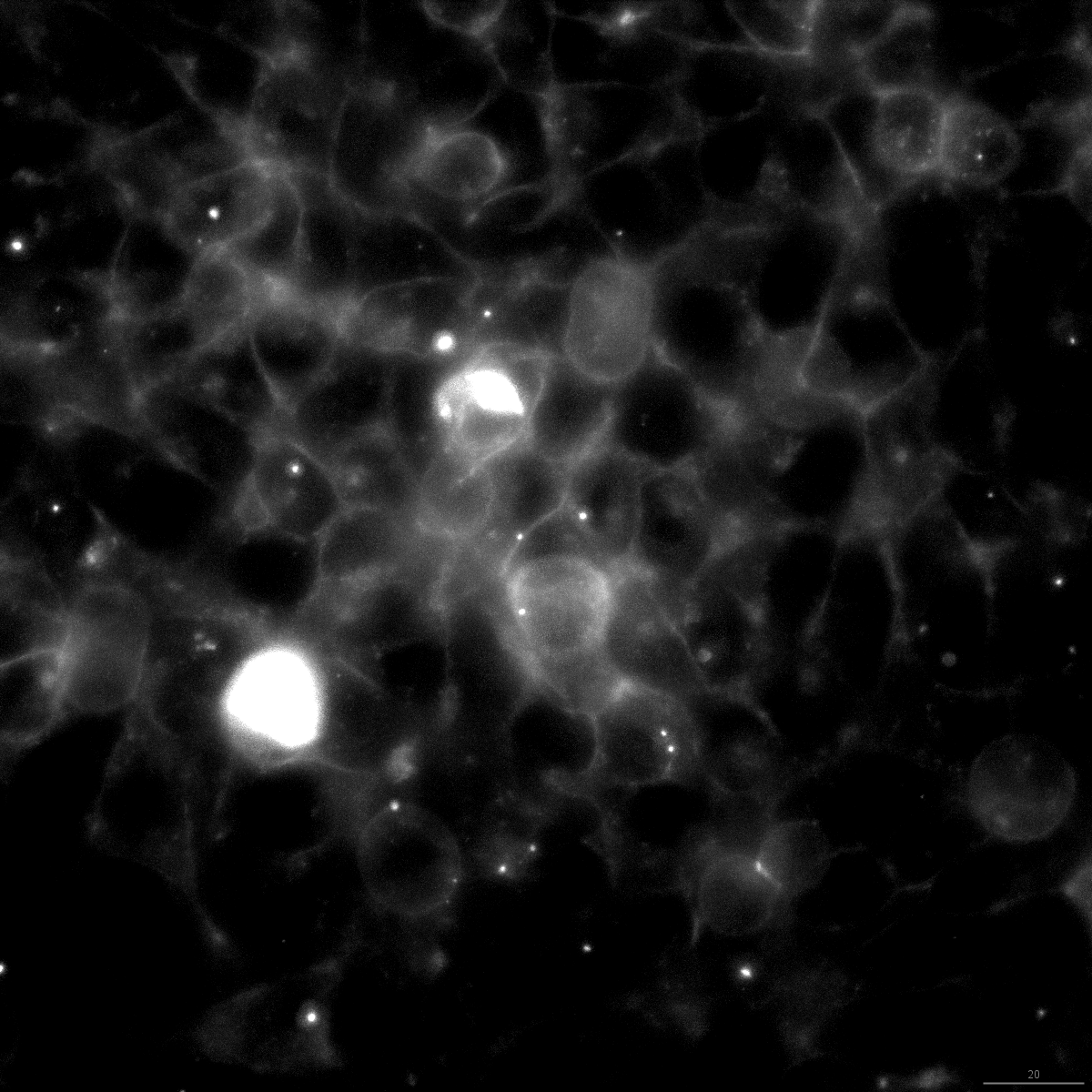

Supplement: Supplementary file 10 — Source Data for Figure 7 [file EMBJ-42-e113647-s009.zip › Figure 7/Figure 7G/uncropped PRC1/4 min/PRC1wtMembrane.tif]

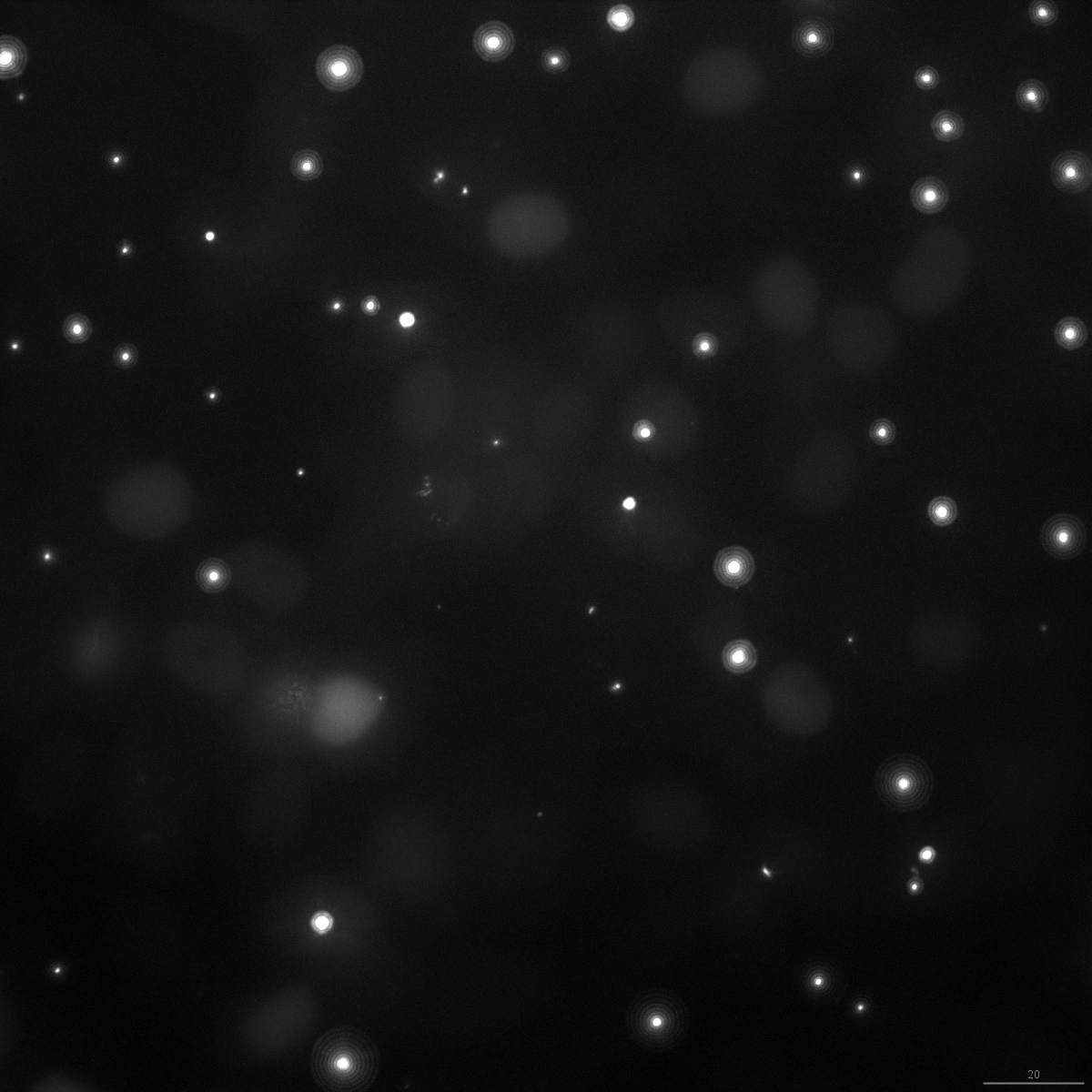

Supplement: Supplementary file 10 — Source Data for Figure 7 [file EMBJ-42-e113647-s009.zip › Figure 7/Figure 7G/uncropped PRC1/-2 min/PRC1wtGFP.tif]

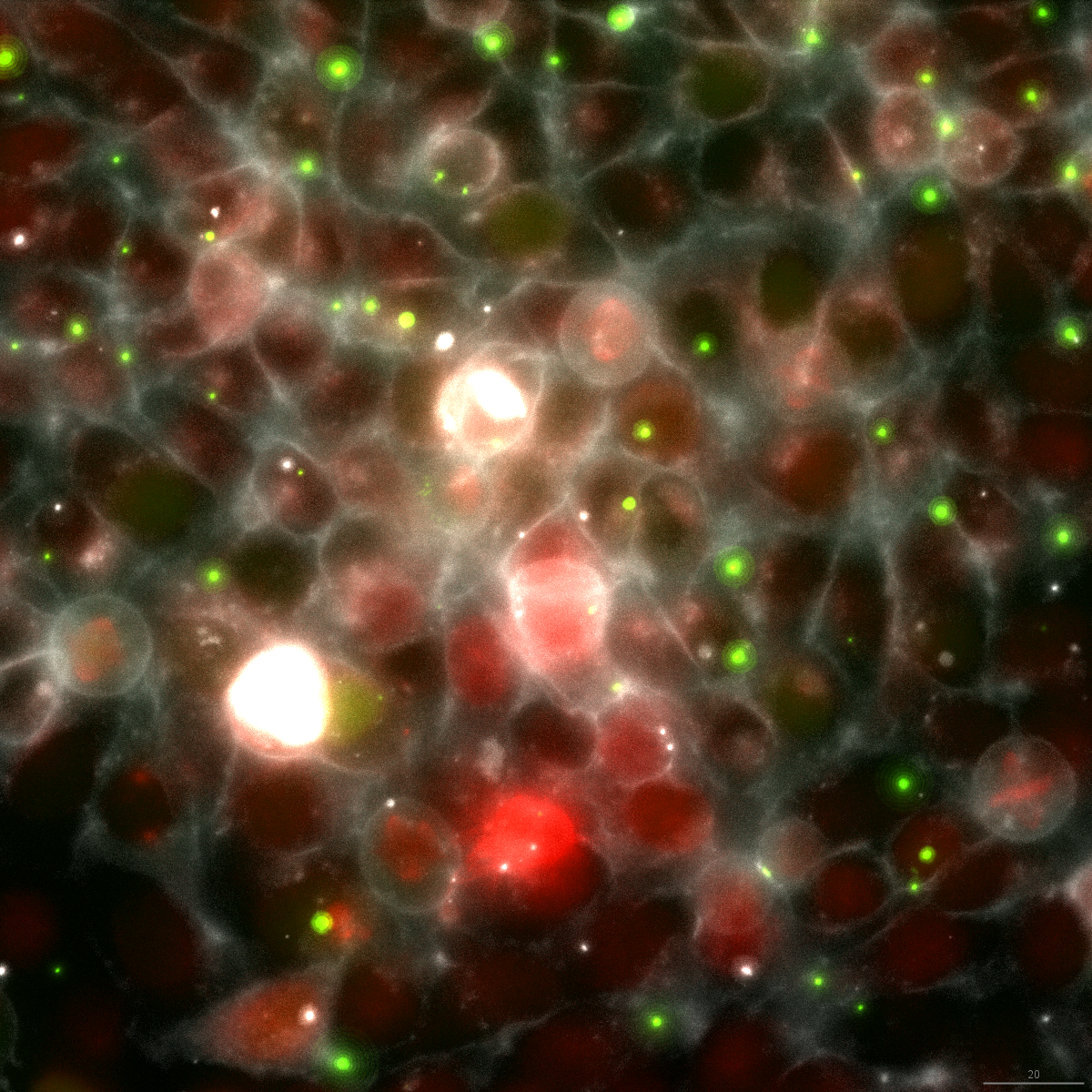

Supplement: Supplementary file 10 — Source Data for Figure 7 [file EMBJ-42-e113647-s009.zip › Figure 7/Figure 7G/uncropped PRC1/-2 min/PRC1wtMerge.tif]

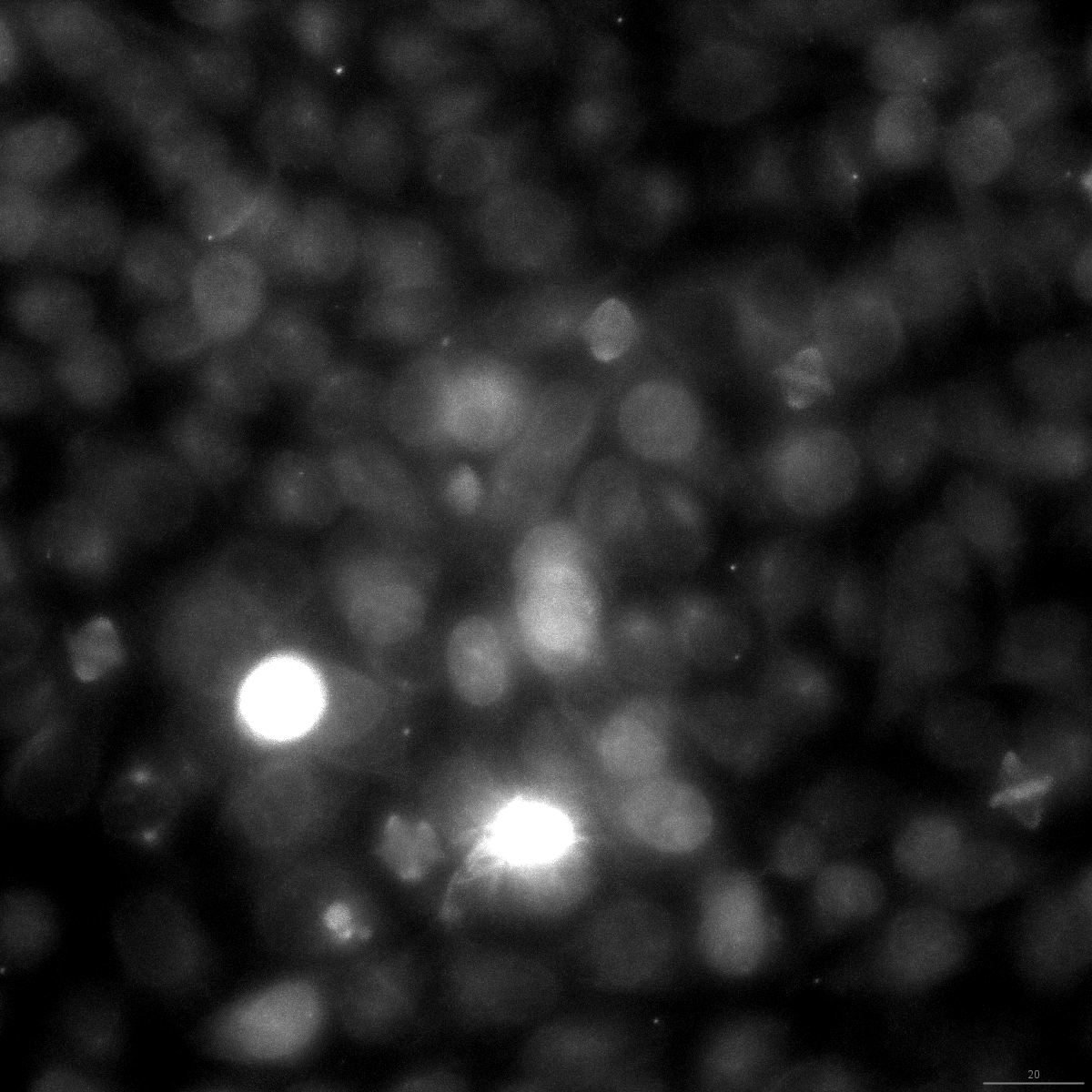

Supplement: Supplementary file 10 — Source Data for Figure 7 [file EMBJ-42-e113647-s009.zip › Figure 7/Figure 7G/uncropped PRC1/-2 min/PRC1wtMT.tif]

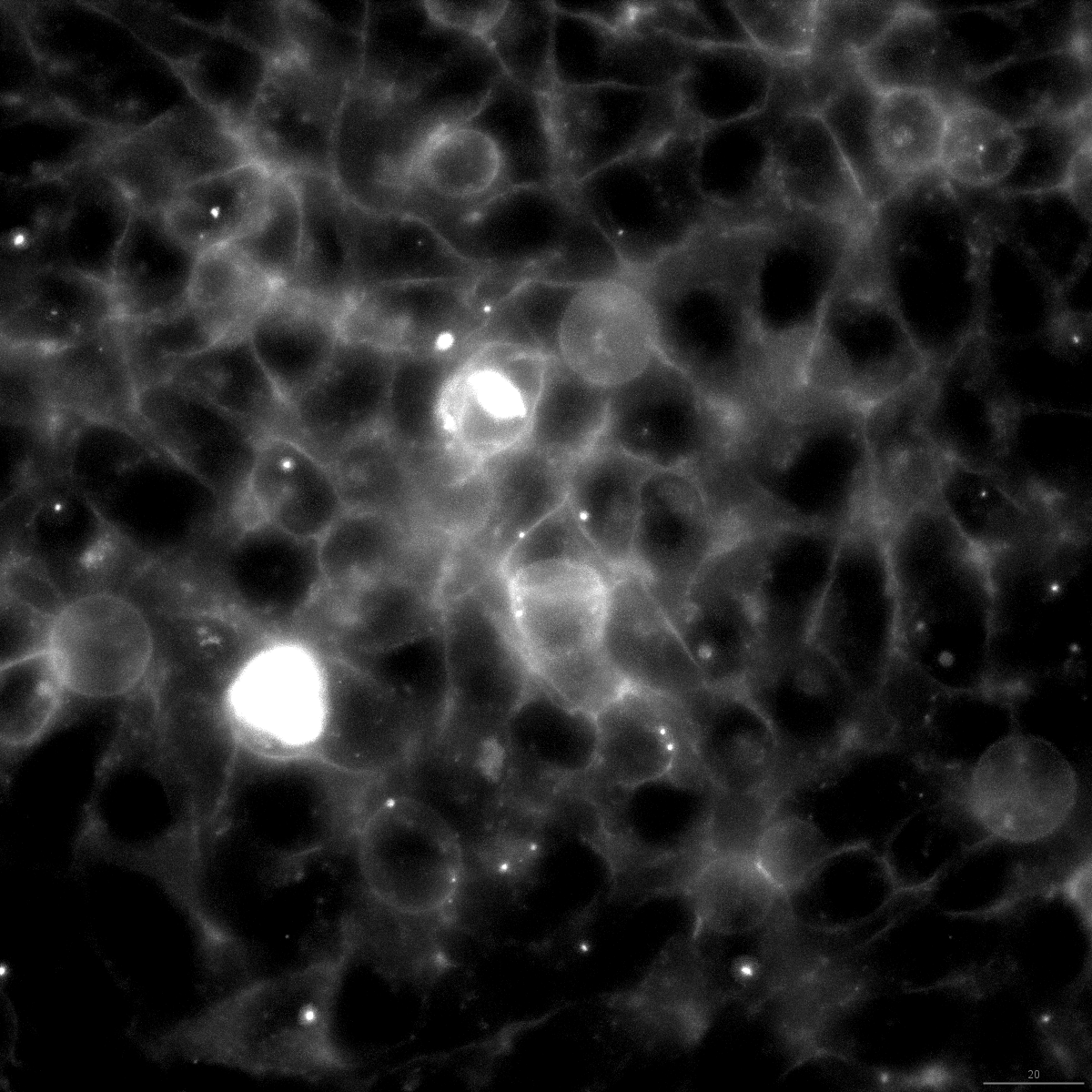

Supplement: Supplementary file 10 — Source Data for Figure 7 [file EMBJ-42-e113647-s009.zip › Figure 7/Figure 7G/uncropped PRC1/-2 min/PRC1wtMembrane.tif]

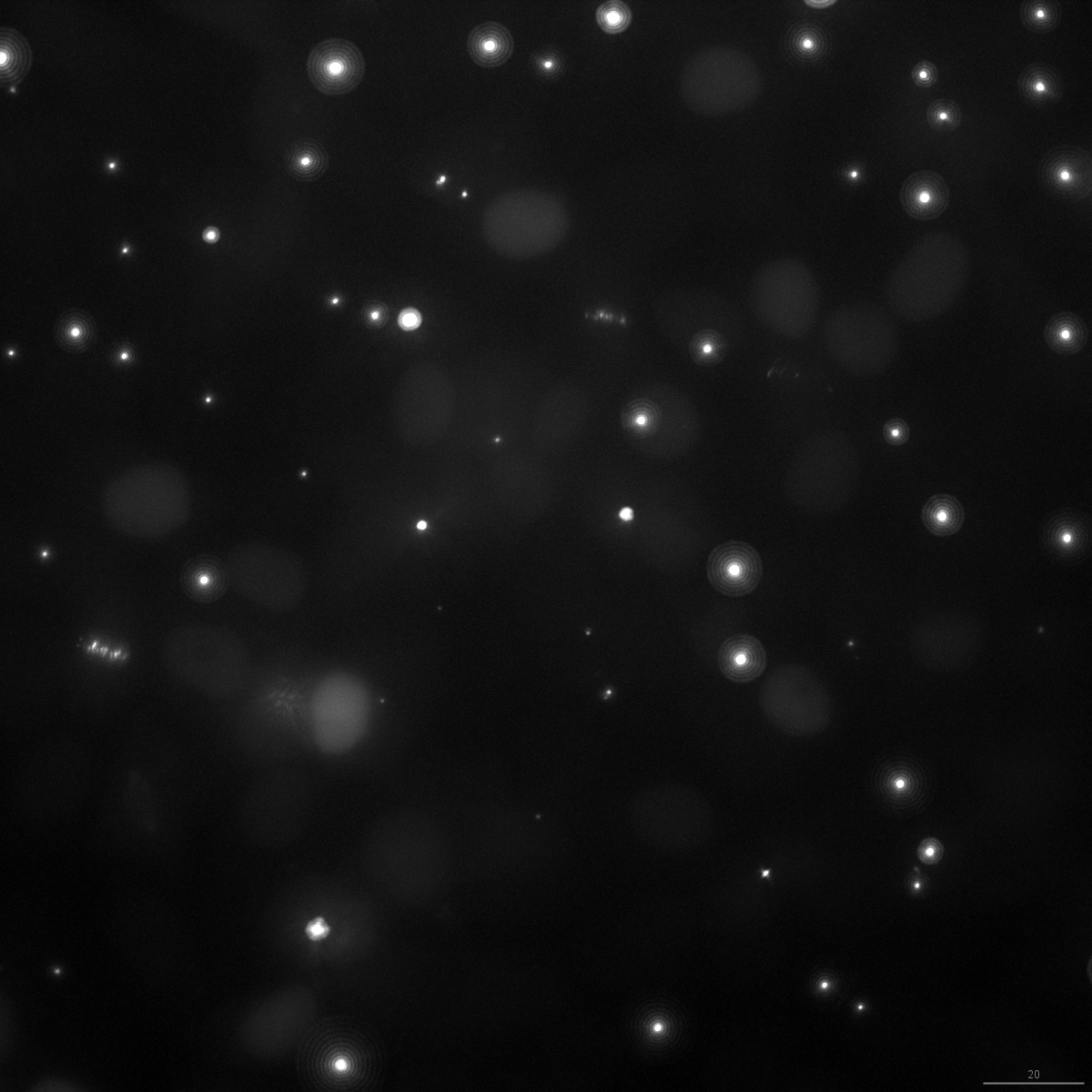

Supplement: Supplementary file 10 — Source Data for Figure 7 [file EMBJ-42-e113647-s009.zip › Figure 7/Figure 7G/uncropped PRC1/6 min/PRC1wtGFP.tif]

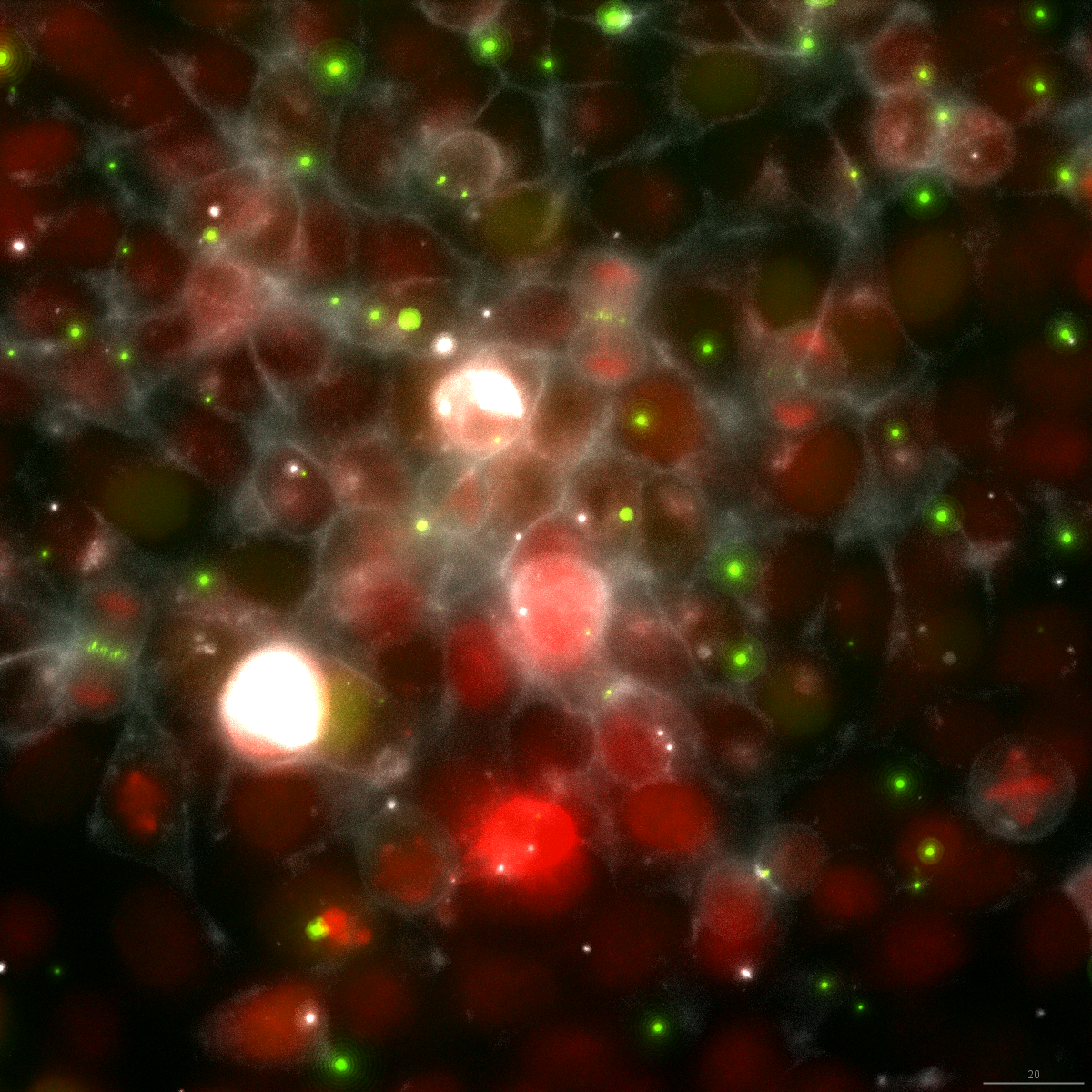

Supplement: Supplementary file 10 — Source Data for Figure 7 [file EMBJ-42-e113647-s009.zip › Figure 7/Figure 7G/uncropped PRC1/6 min/PRC1wtMerge.tif]

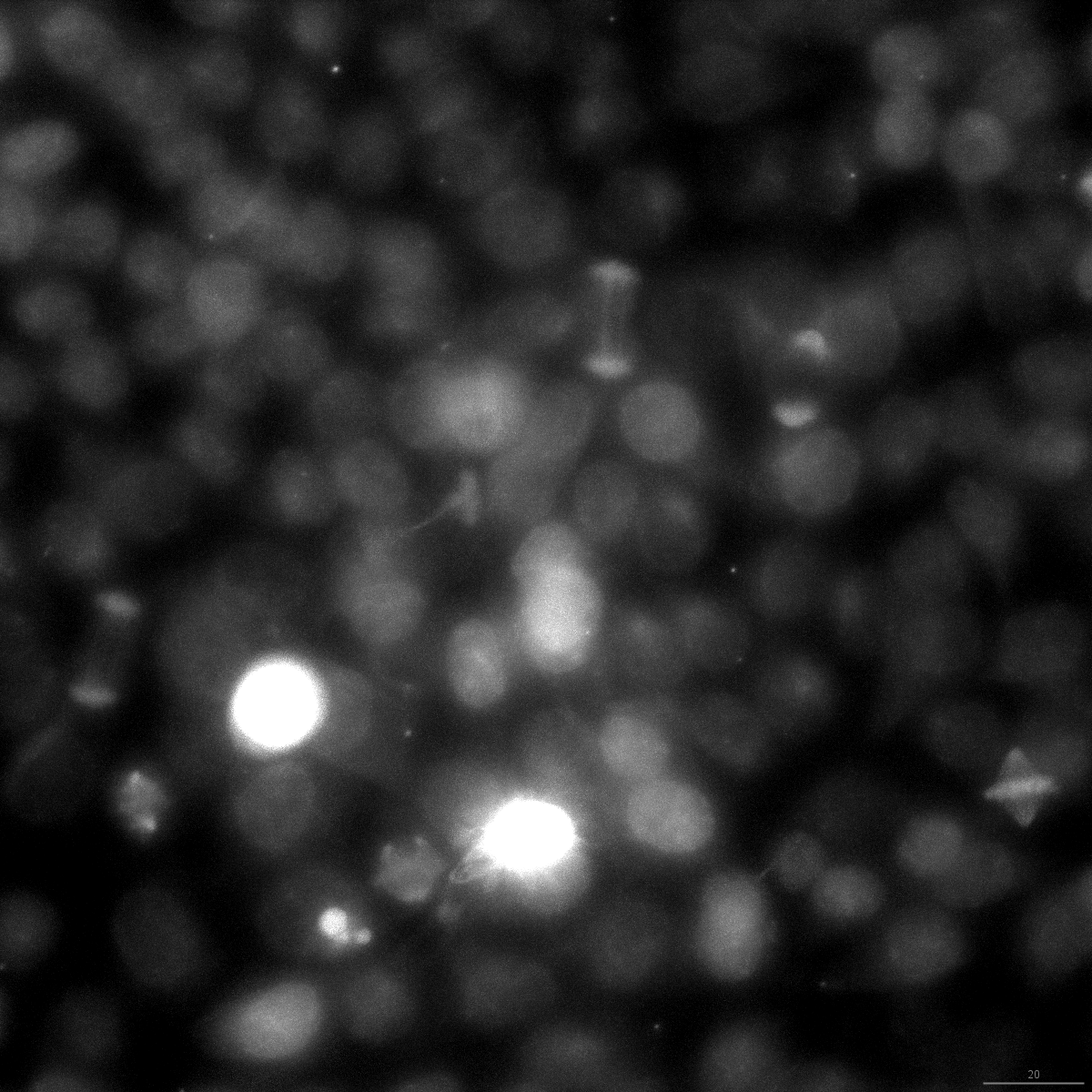

Supplement: Supplementary file 10 — Source Data for Figure 7 [file EMBJ-42-e113647-s009.zip › Figure 7/Figure 7G/uncropped PRC1/6 min/PRC1wtMT.tif]

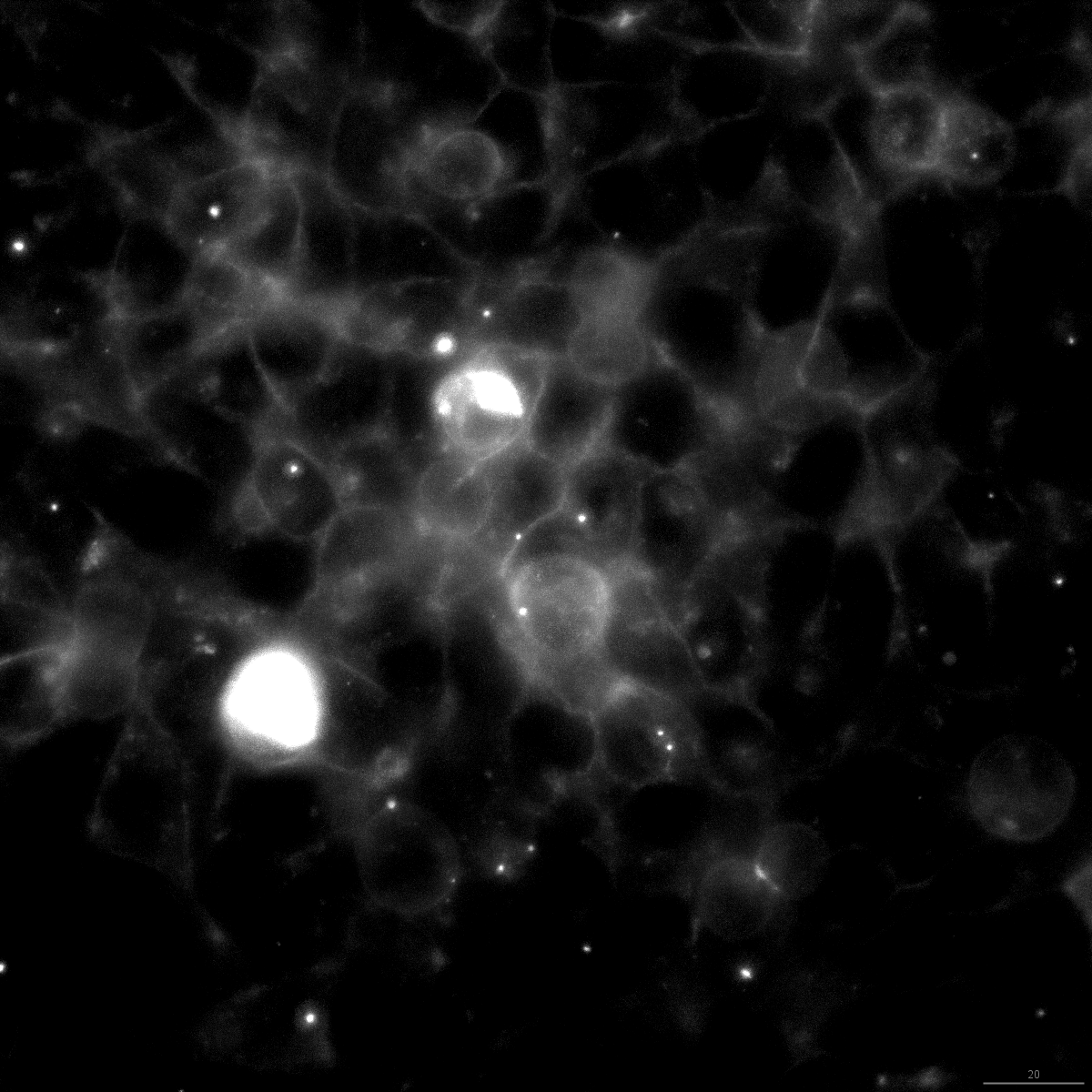

Supplement: Supplementary file 10 — Source Data for Figure 7 [file EMBJ-42-e113647-s009.zip › Figure 7/Figure 7G/uncropped PRC1/6 min/PRC1wtMembrane.tif]

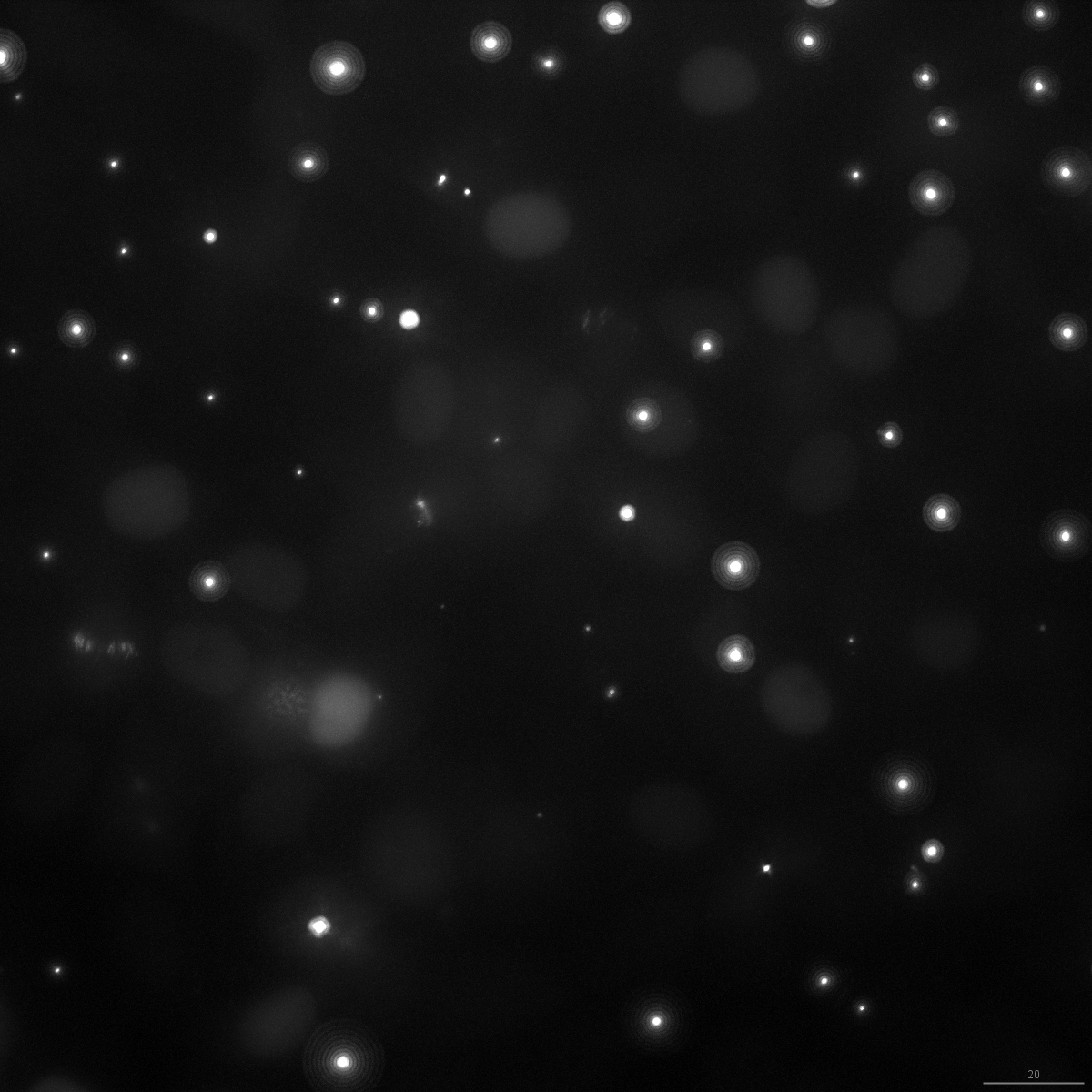

Supplement: Supplementary file 10 — Source Data for Figure 7 [file EMBJ-42-e113647-s009.zip › Figure 7/Figure 7G/uncropped PRC1/2 min/PRC1wtGFP.tif]

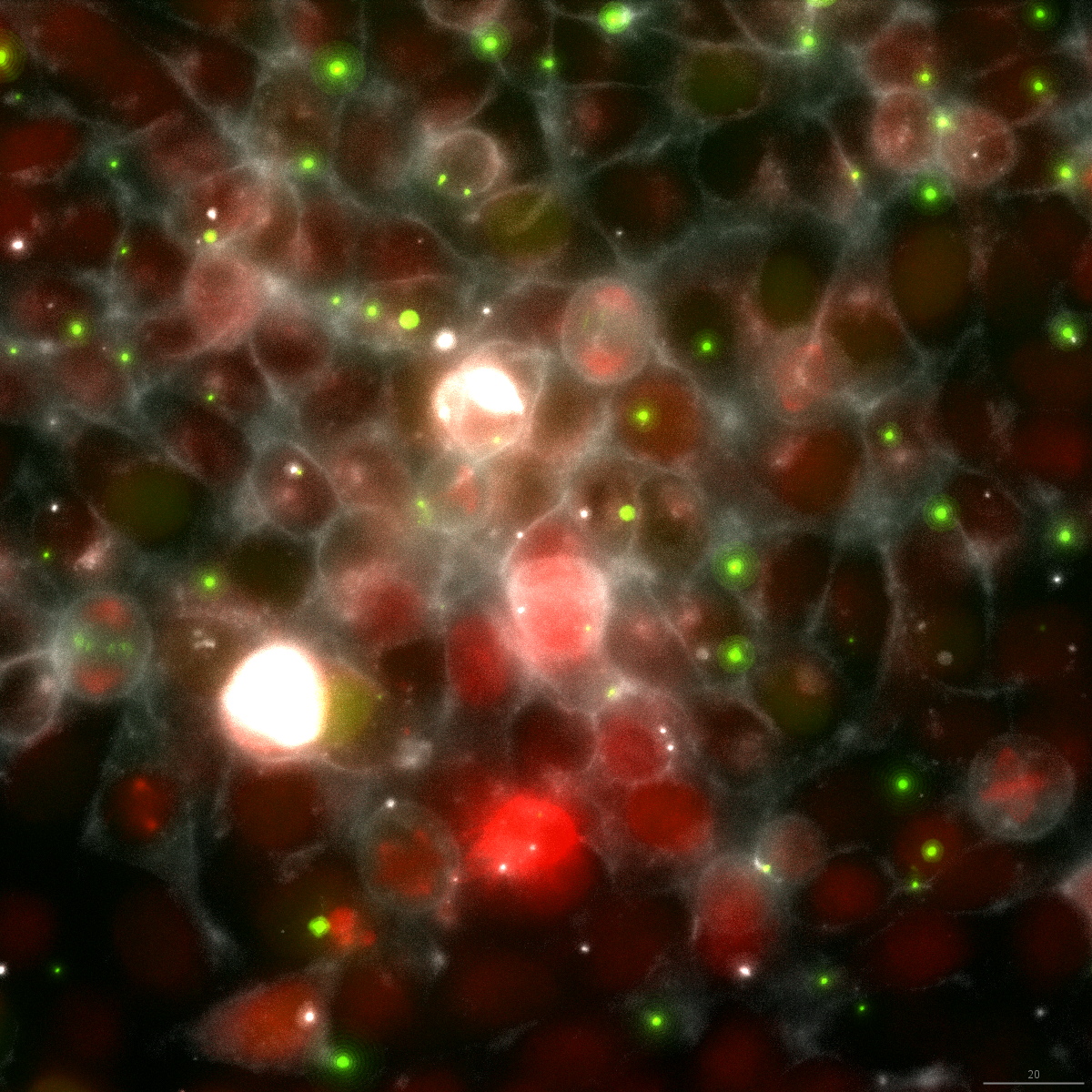

Supplement: Supplementary file 10 — Source Data for Figure 7 [file EMBJ-42-e113647-s009.zip › Figure 7/Figure 7G/uncropped PRC1/2 min/PRC1wtMerge.tif]

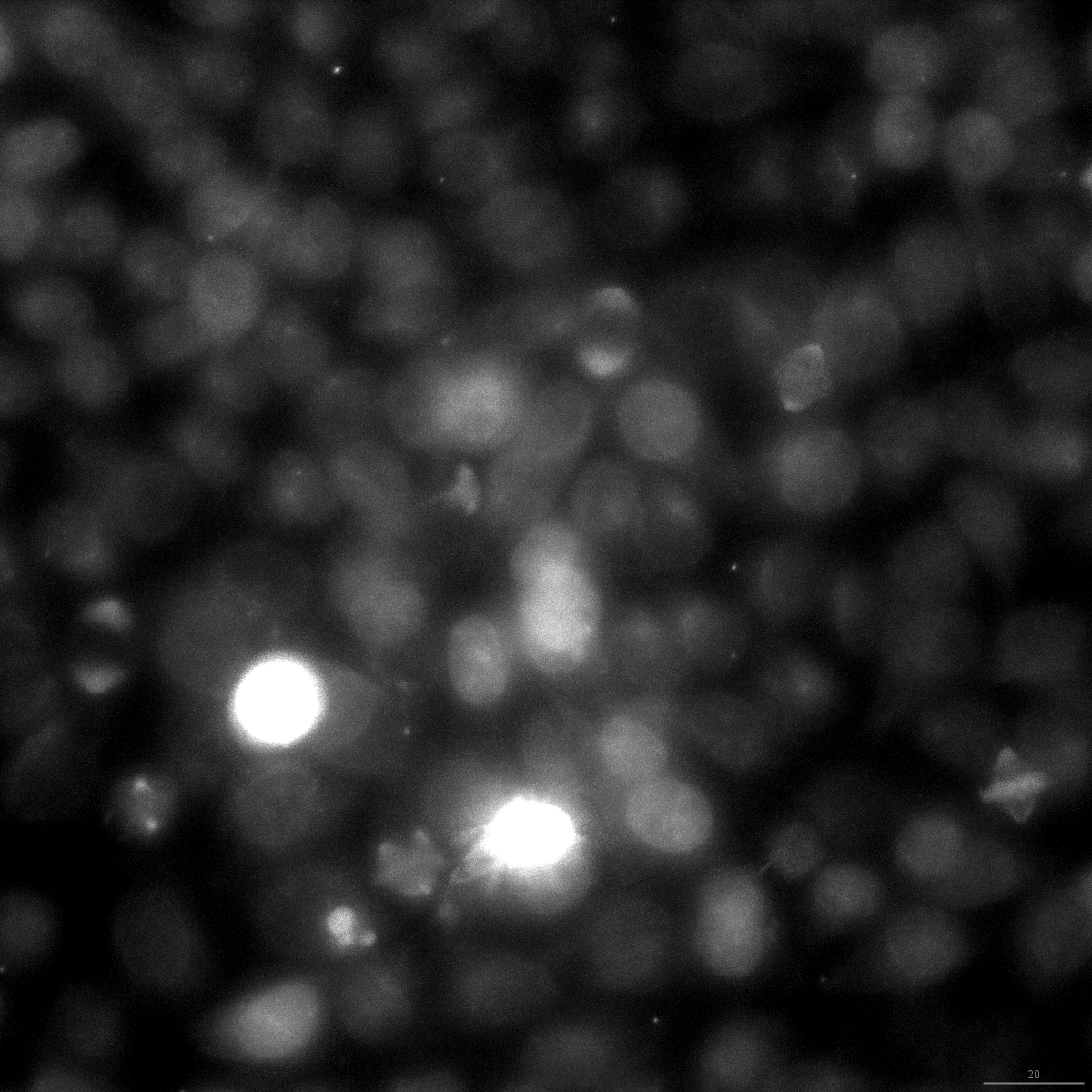

Supplement: Supplementary file 10 — Source Data for Figure 7 [file EMBJ-42-e113647-s009.zip › Figure 7/Figure 7G/uncropped PRC1/2 min/PRC1wtMT.tif]

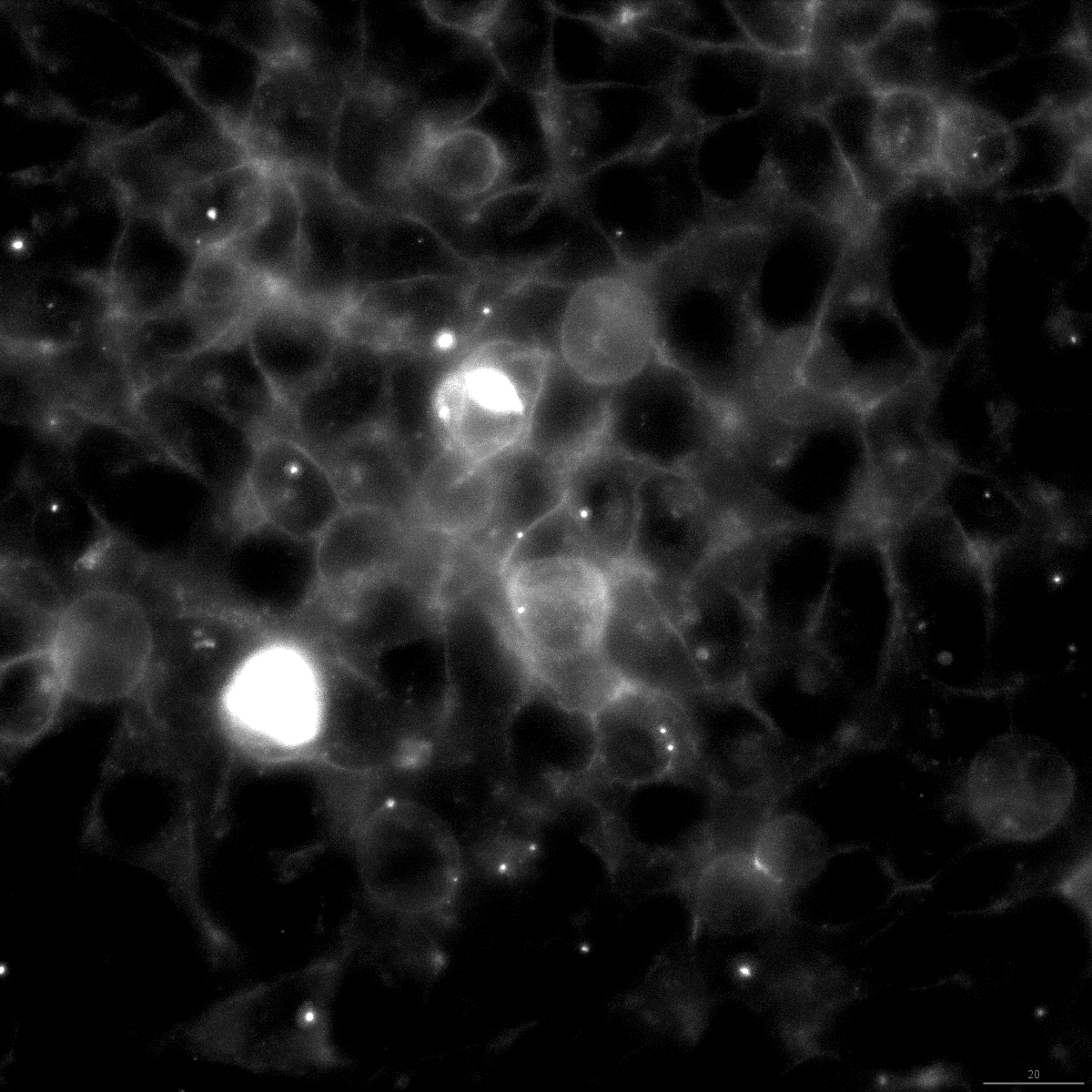

Supplement: Supplementary file 10 — Source Data for Figure 7 [file EMBJ-42-e113647-s009.zip › Figure 7/Figure 7G/uncropped PRC1/2 min/PRC1wtMembrane.tif]

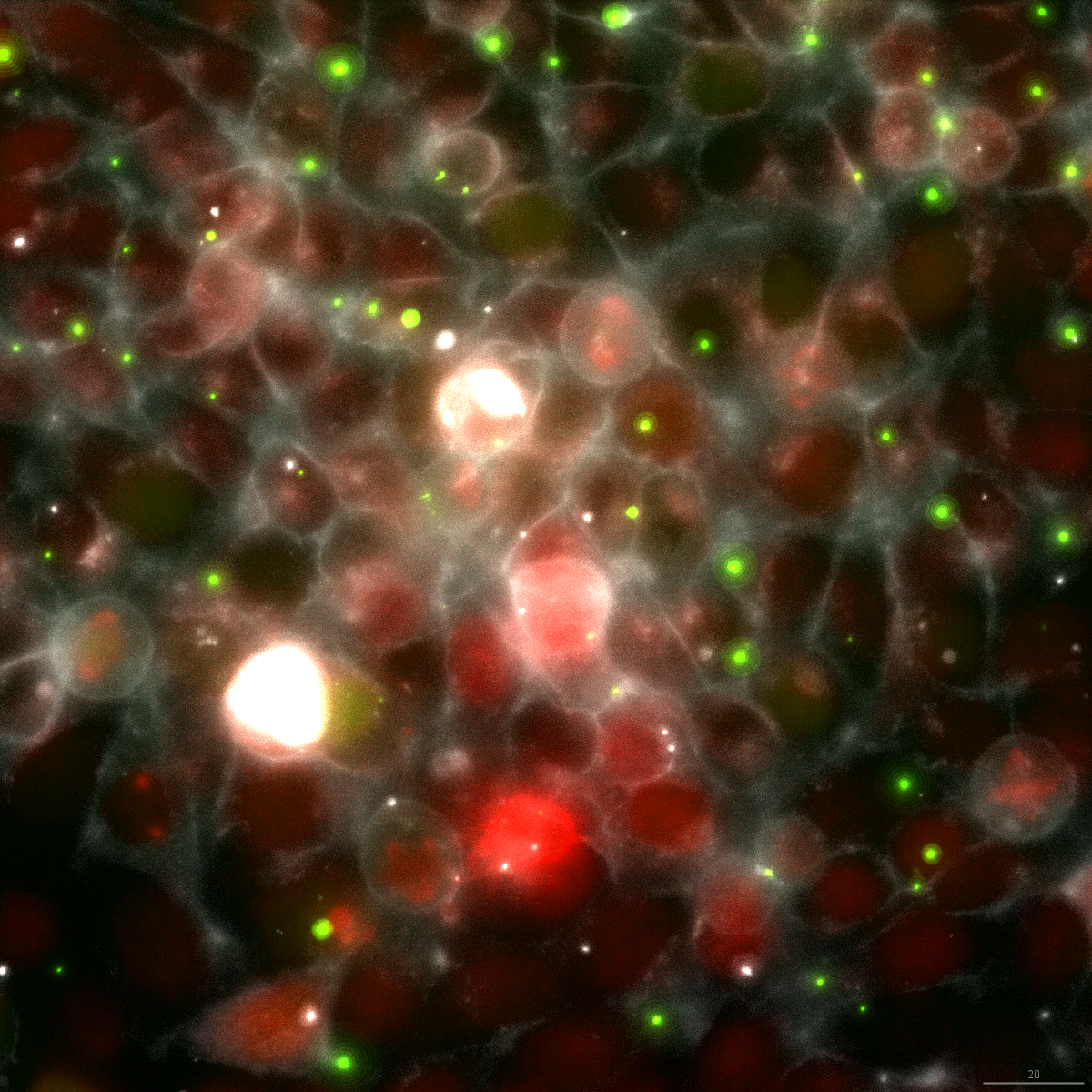

Supplement: Supplementary file 10 — Source Data for Figure 7 [file EMBJ-42-e113647-s009.zip › Figure 7/Figure 7G/uncropped PRC1/0 min/PRC1wtmerge.tif]

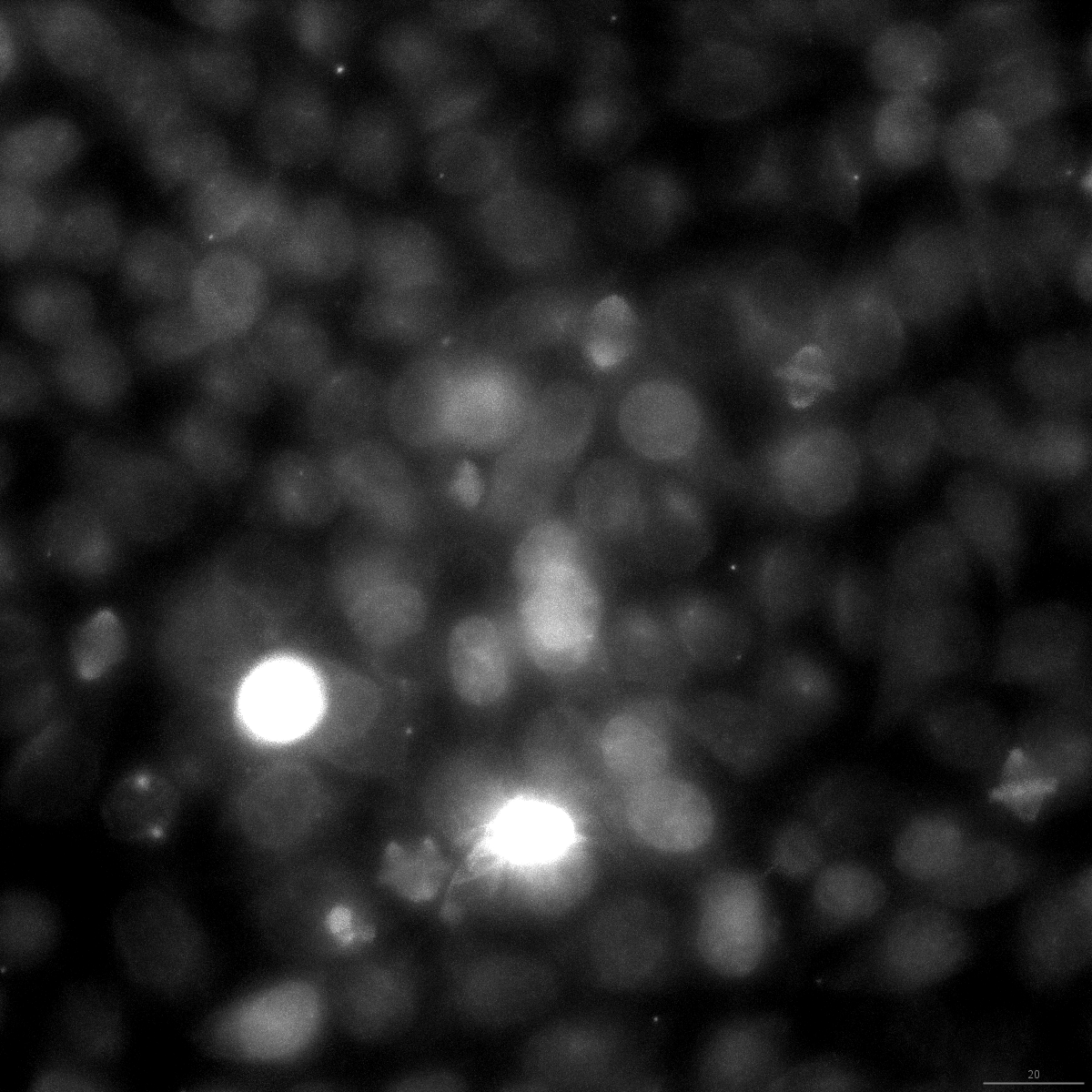

Supplement: Supplementary file 10 — Source Data for Figure 7 [file EMBJ-42-e113647-s009.zip › Figure 7/Figure 7G/uncropped PRC1/0 min/PRC1wtMT.tif]

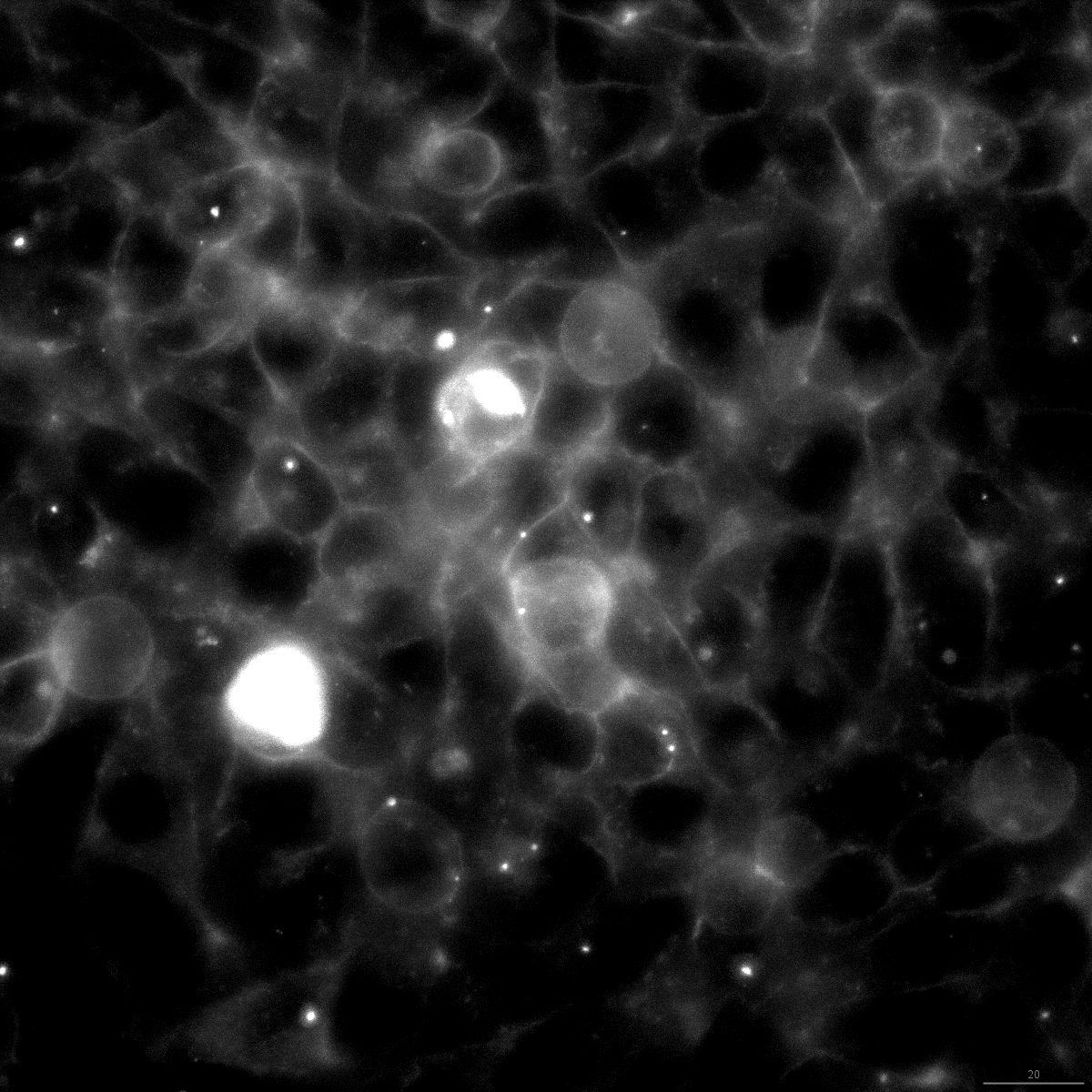

Supplement: Supplementary file 10 — Source Data for Figure 7 [file EMBJ-42-e113647-s009.zip › Figure 7/Figure 7G/uncropped PRC1/0 min/PRC1wtMembrane.tif]

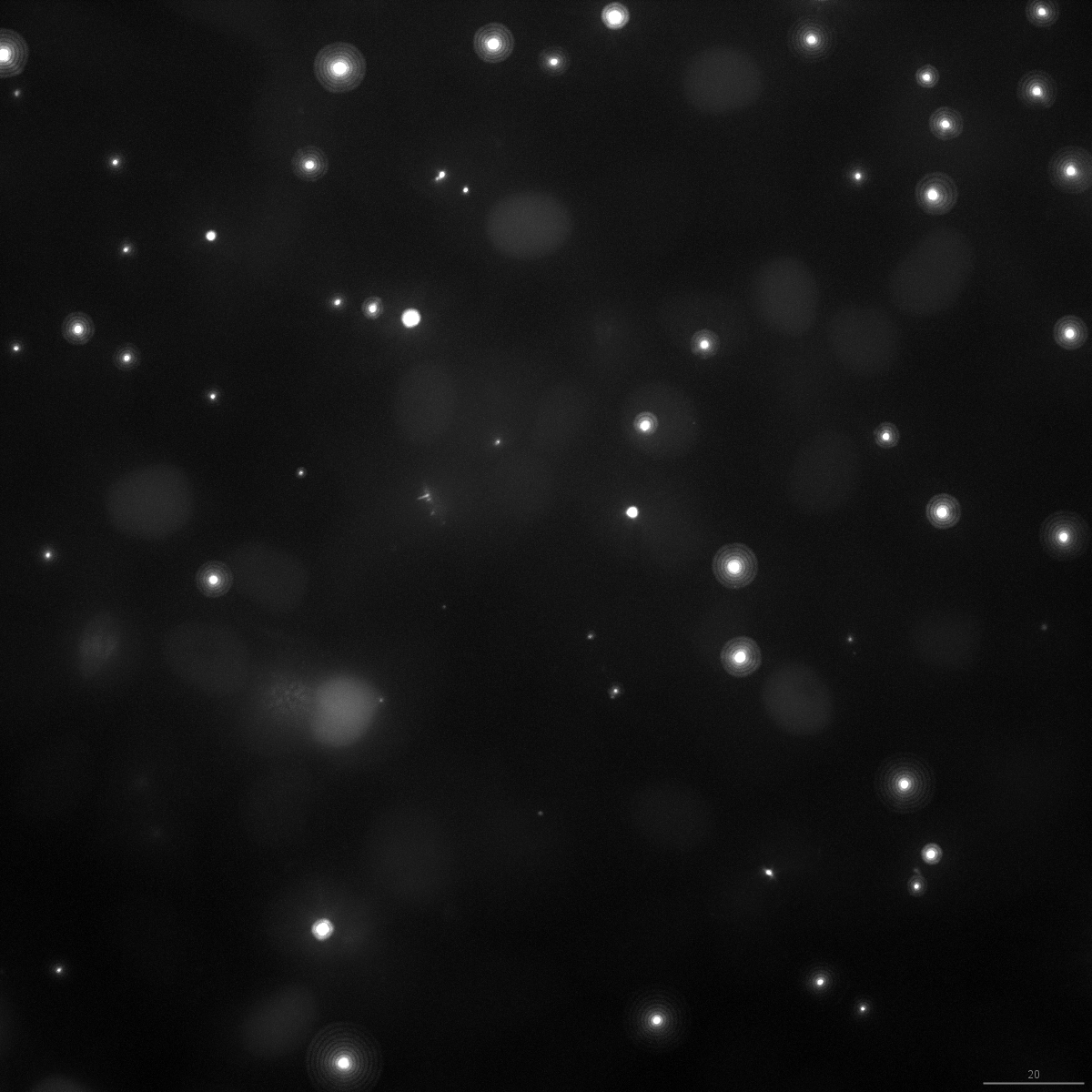

Supplement: Supplementary file 10 — Source Data for Figure 7 [file EMBJ-42-e113647-s009.zip › Figure 7/Figure 7G/uncropped PRC1/0 min/PRC1wtjGFP.tif]

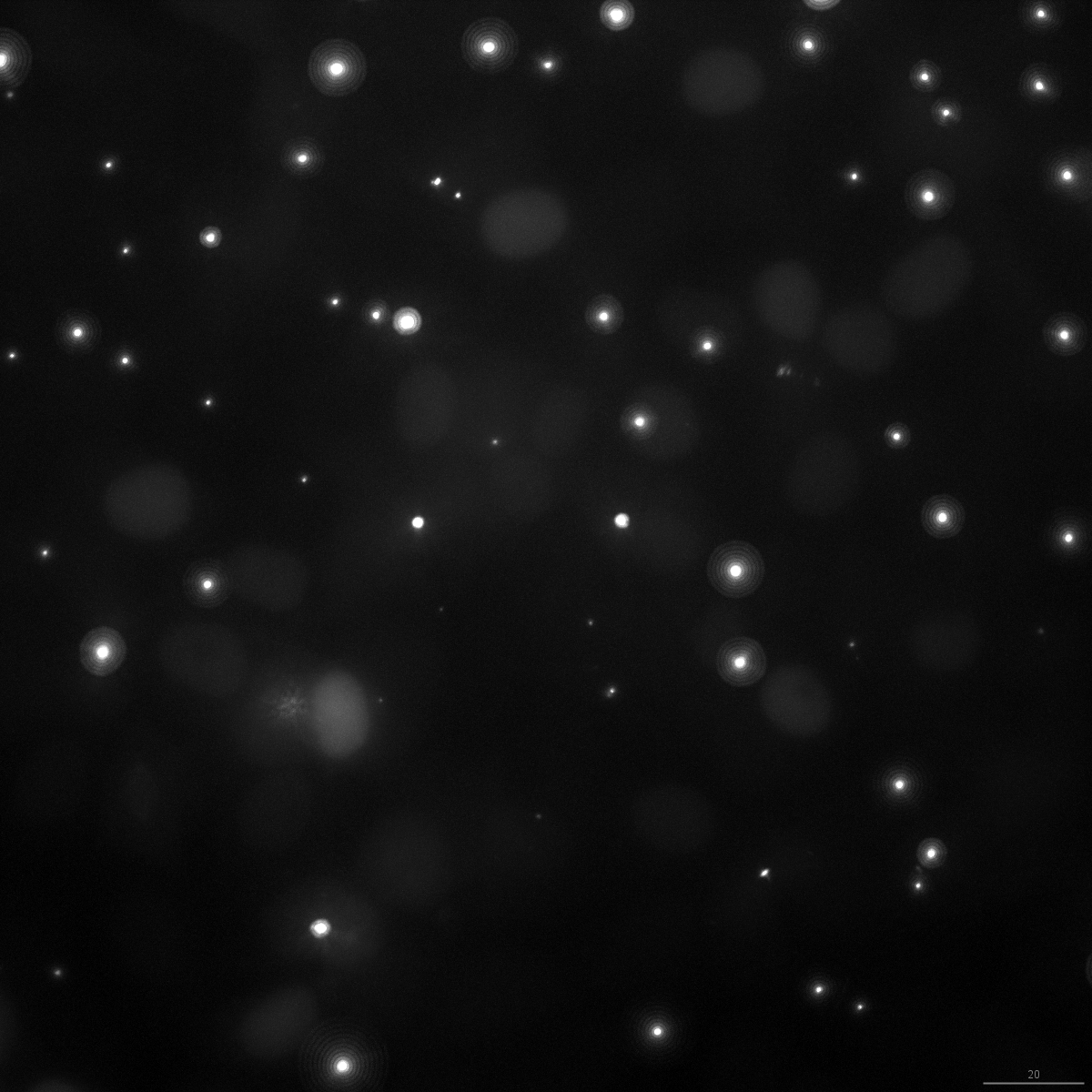

Supplement: Supplementary file 10 — Source Data for Figure 7 [file EMBJ-42-e113647-s009.zip › Figure 7/Figure 7G/uncropped PRC1/10 min/PRC1wtGFP.tif]

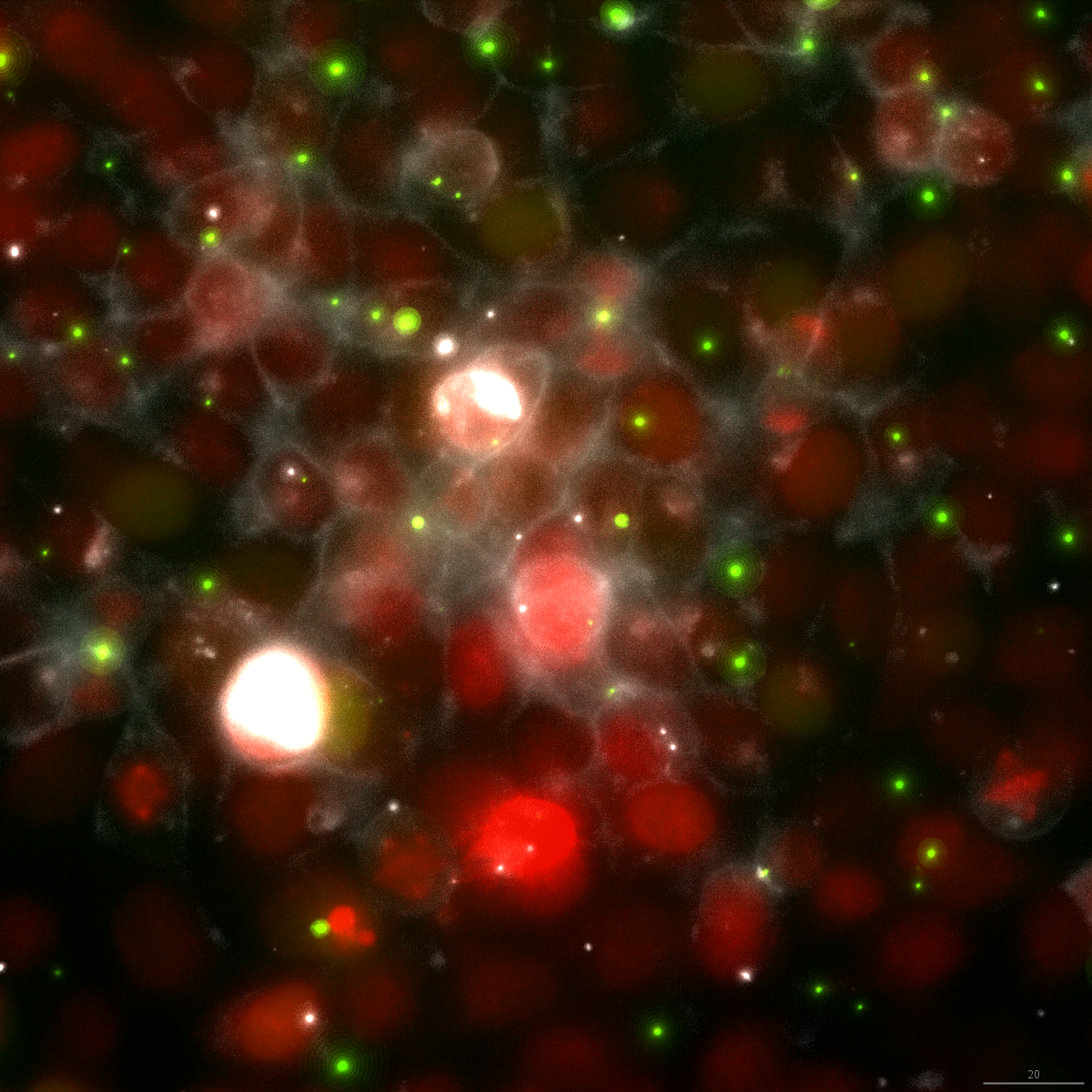

Supplement: Supplementary file 10 — Source Data for Figure 7 [file EMBJ-42-e113647-s009.zip › Figure 7/Figure 7G/uncropped PRC1/10 min/PRC1wtMerge.tif]

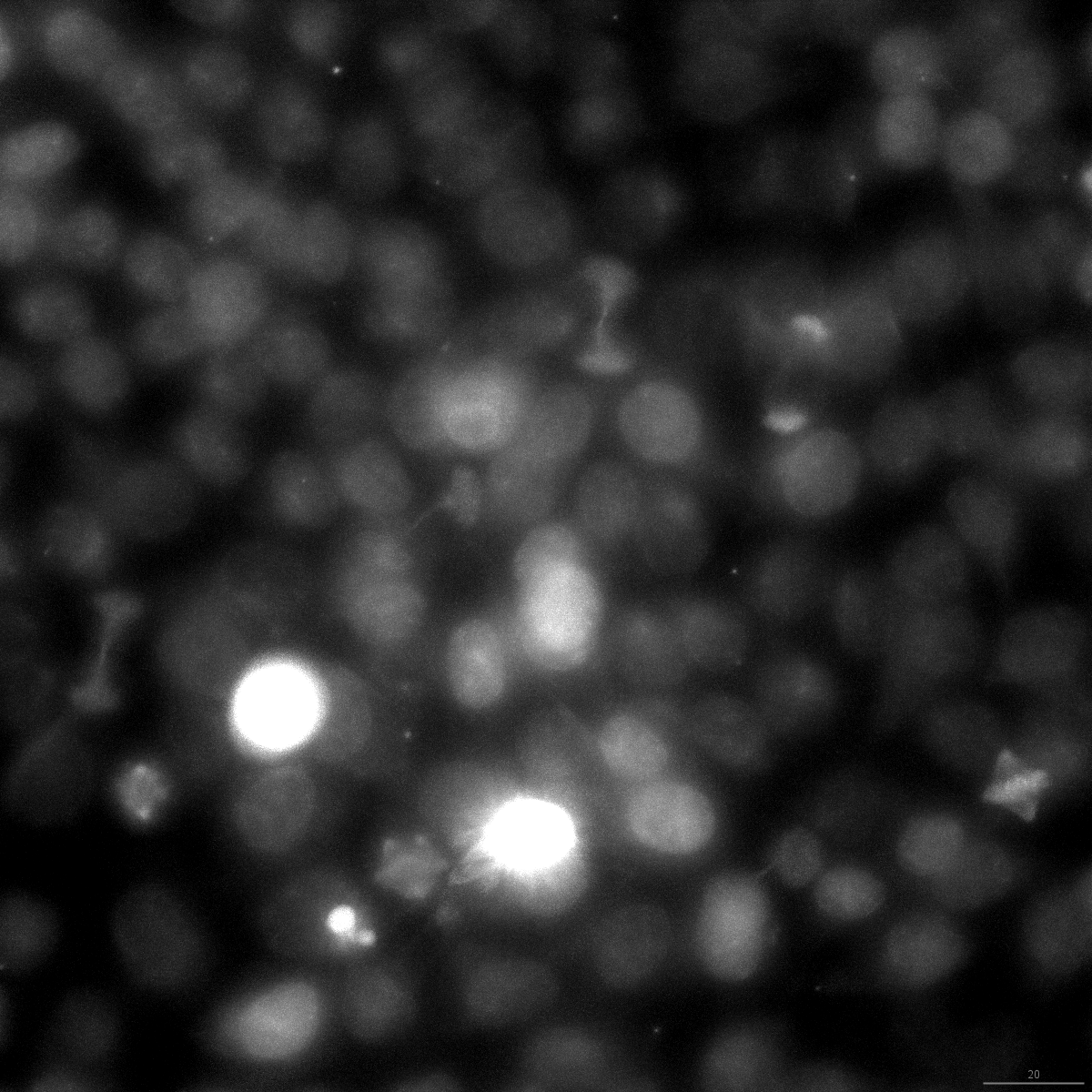

Supplement: Supplementary file 10 — Source Data for Figure 7 [file EMBJ-42-e113647-s009.zip › Figure 7/Figure 7G/uncropped PRC1/10 min/PRC1wtMT.tif]

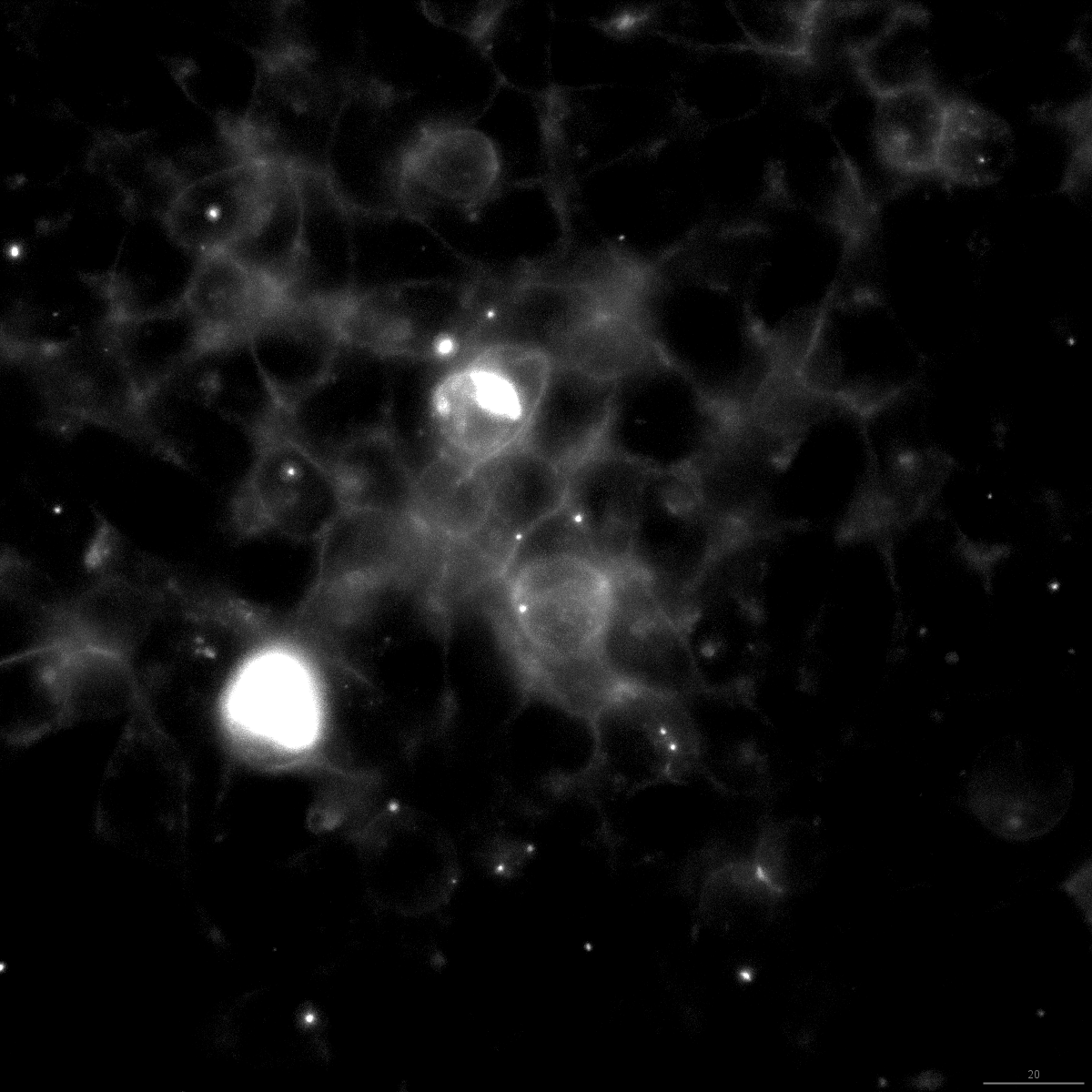

Supplement: Supplementary file 10 — Source Data for Figure 7 [file EMBJ-42-e113647-s009.zip › Figure 7/Figure 7G/uncropped PRC1/10 min/PRC1wtMembrane.tif]

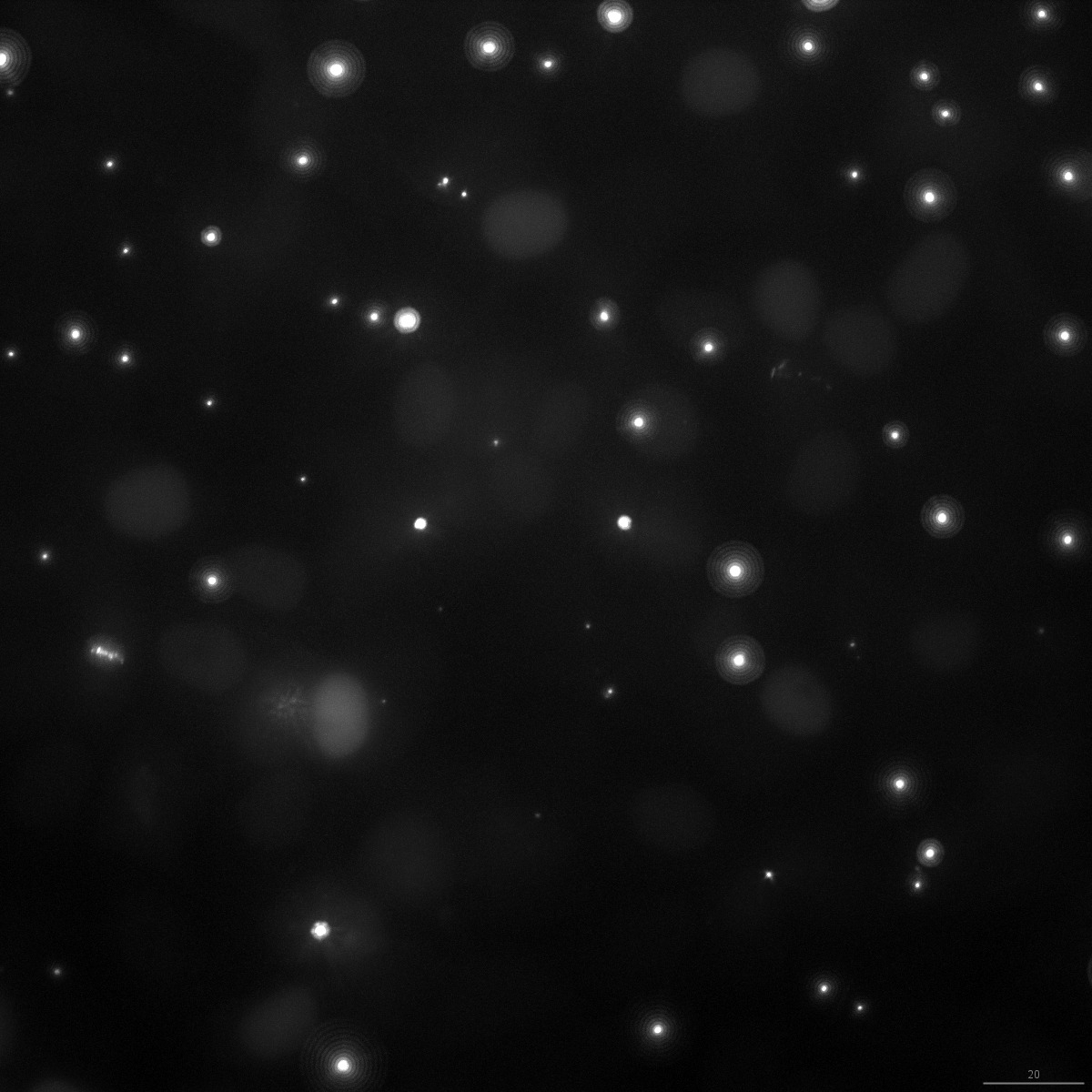

Supplement: Supplementary file 10 — Source Data for Figure 7 [file EMBJ-42-e113647-s009.zip › Figure 7/Figure 7G/uncropped PRC1/8 min/PRC1wtGFP.tif]

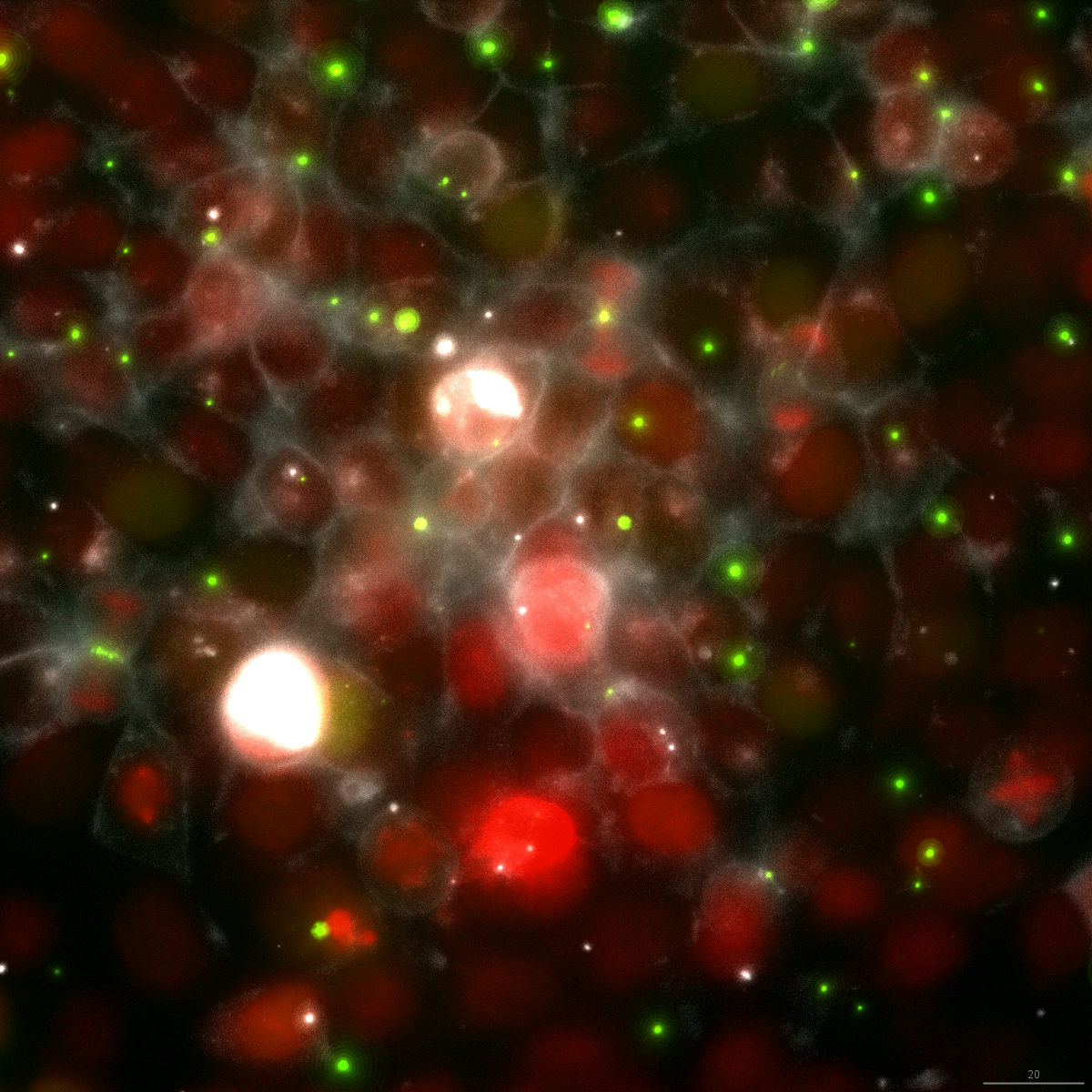

Supplement: Supplementary file 10 — Source Data for Figure 7 [file EMBJ-42-e113647-s009.zip › Figure 7/Figure 7G/uncropped PRC1/8 min/PRC1wtMerge.tif]

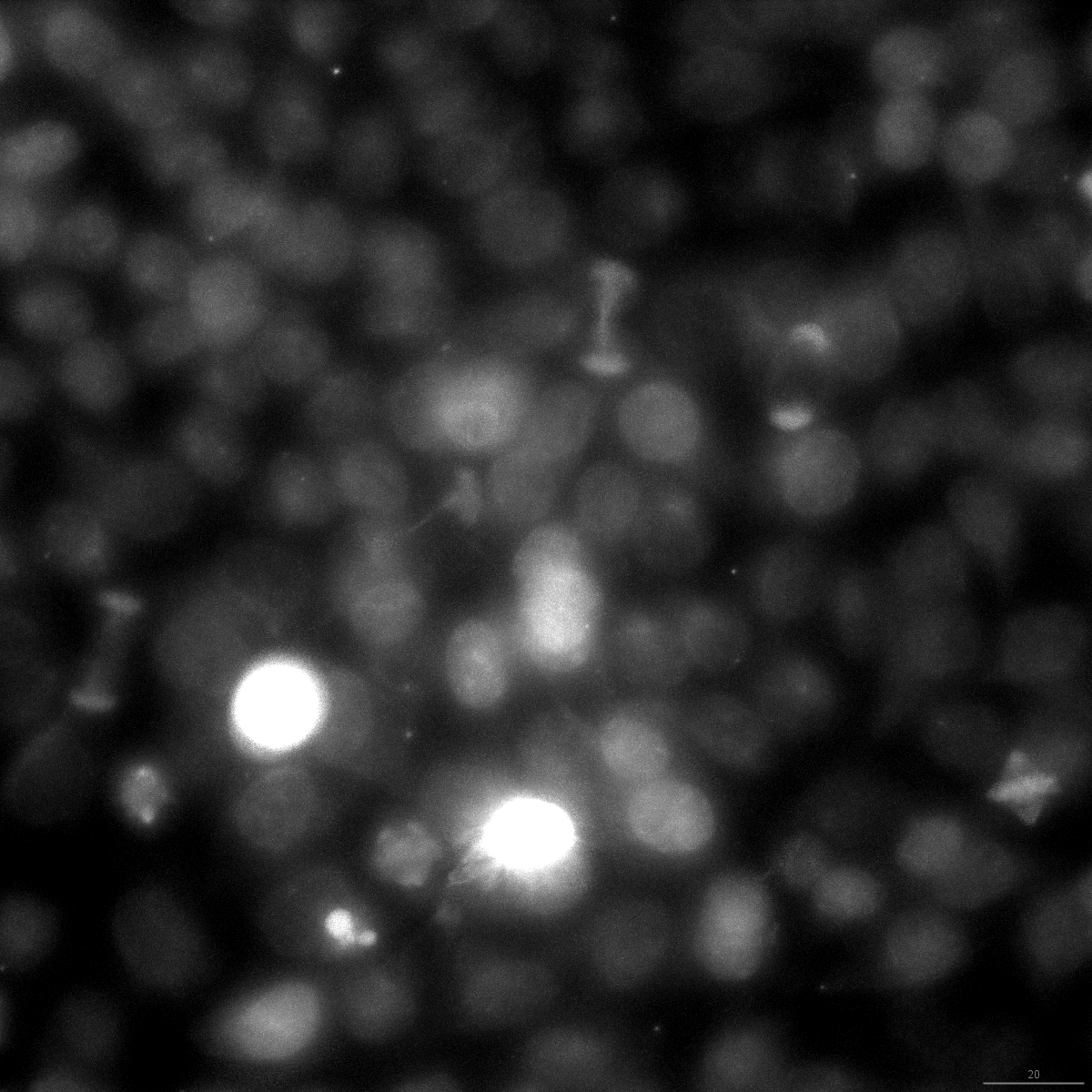

Supplement: Supplementary file 10 — Source Data for Figure 7 [file EMBJ-42-e113647-s009.zip › Figure 7/Figure 7G/uncropped PRC1/8 min/PRC1wtMT.tif]

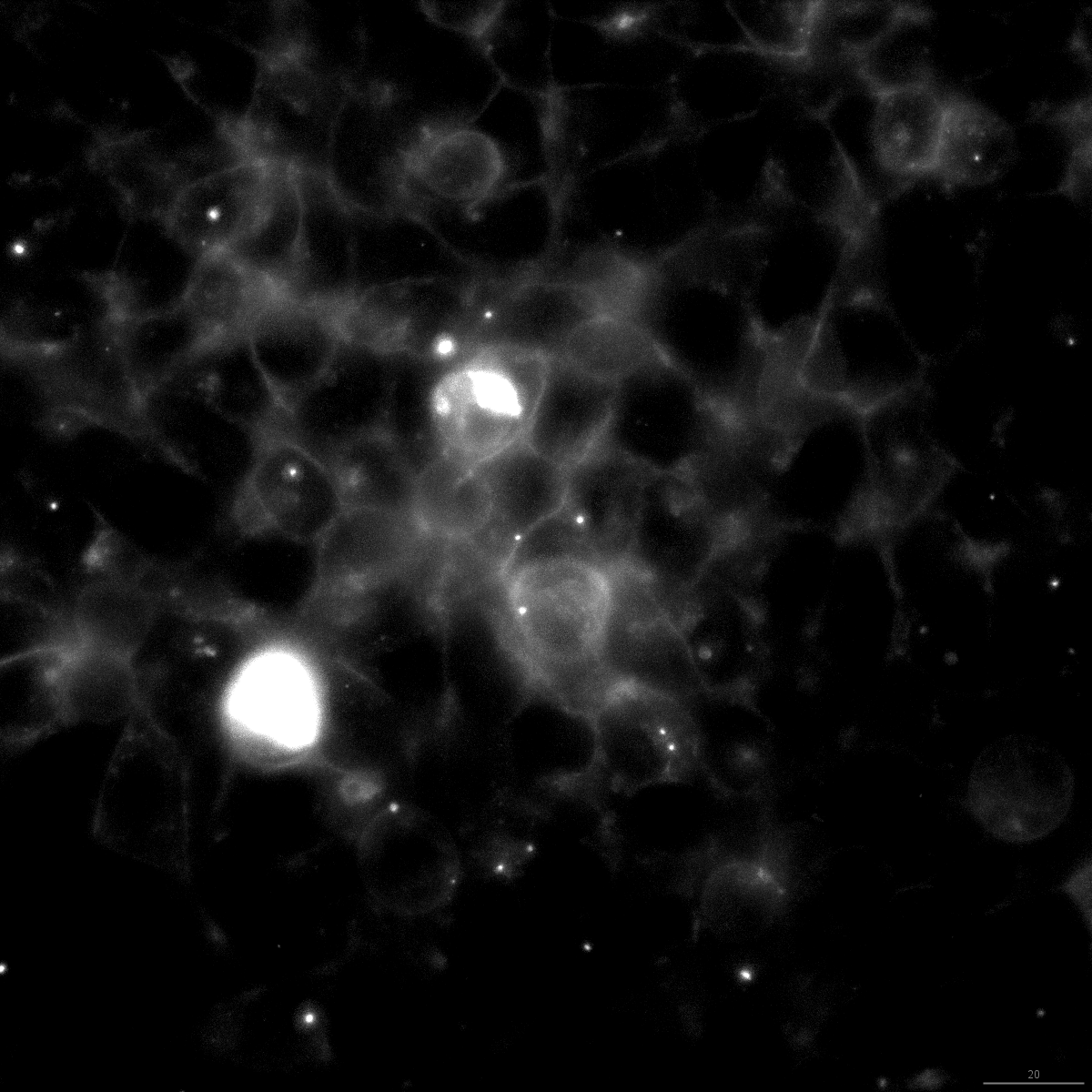

Supplement: Supplementary file 10 — Source Data for Figure 7 [file EMBJ-42-e113647-s009.zip › Figure 7/Figure 7G/uncropped PRC1/8 min/PRC1wtMembrane.tif]

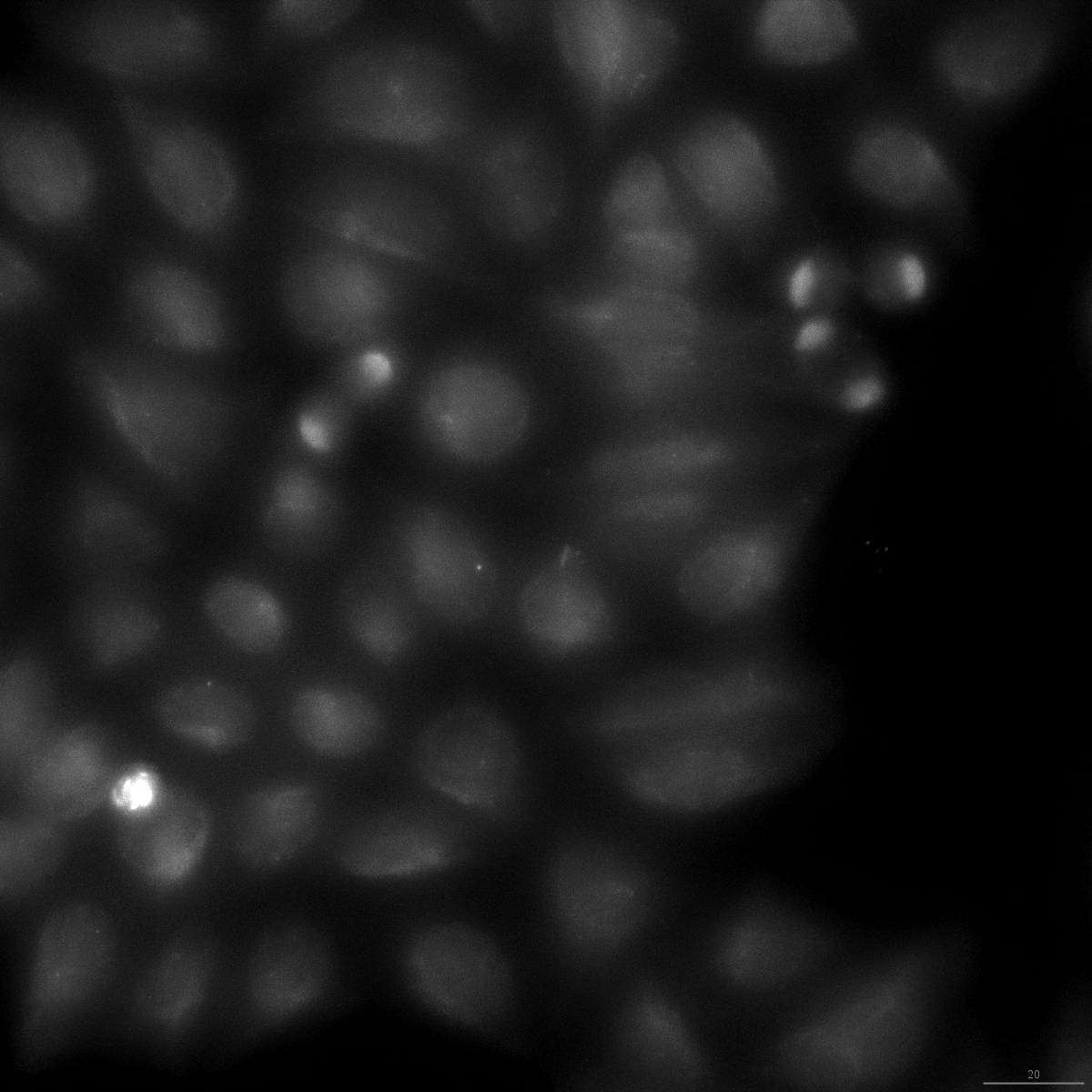

Supplement: Supplementary file 10 — Source Data for Figure 7 [file EMBJ-42-e113647-s009.zip › Figure 7/Figure 7G/uncropped MEE/4 min/PRC1MEEto3AMT.tif]

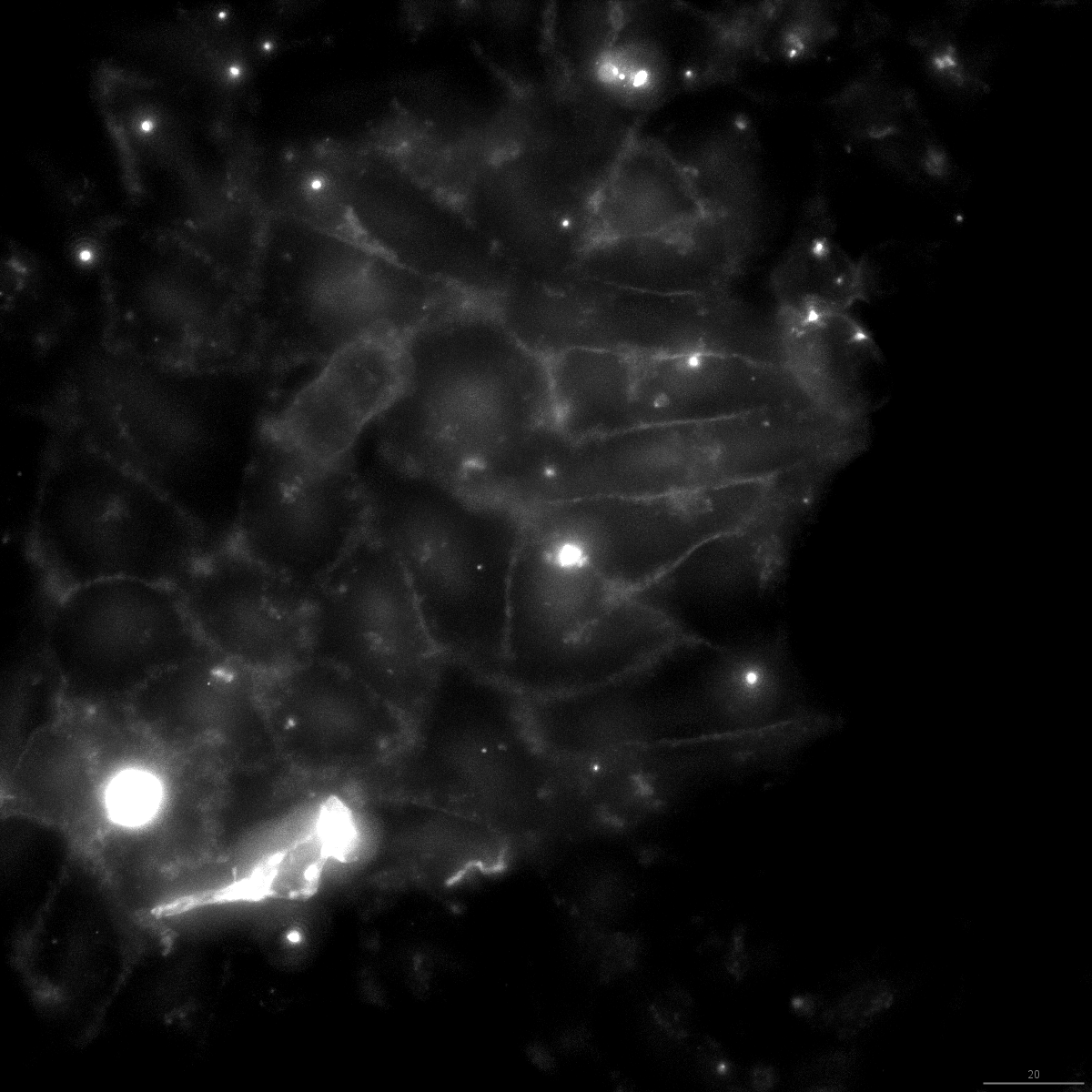

Supplement: Supplementary file 10 — Source Data for Figure 7 [file EMBJ-42-e113647-s009.zip › Figure 7/Figure 7G/uncropped MEE/4 min/PRC1MEEto3AMembrane.tif]

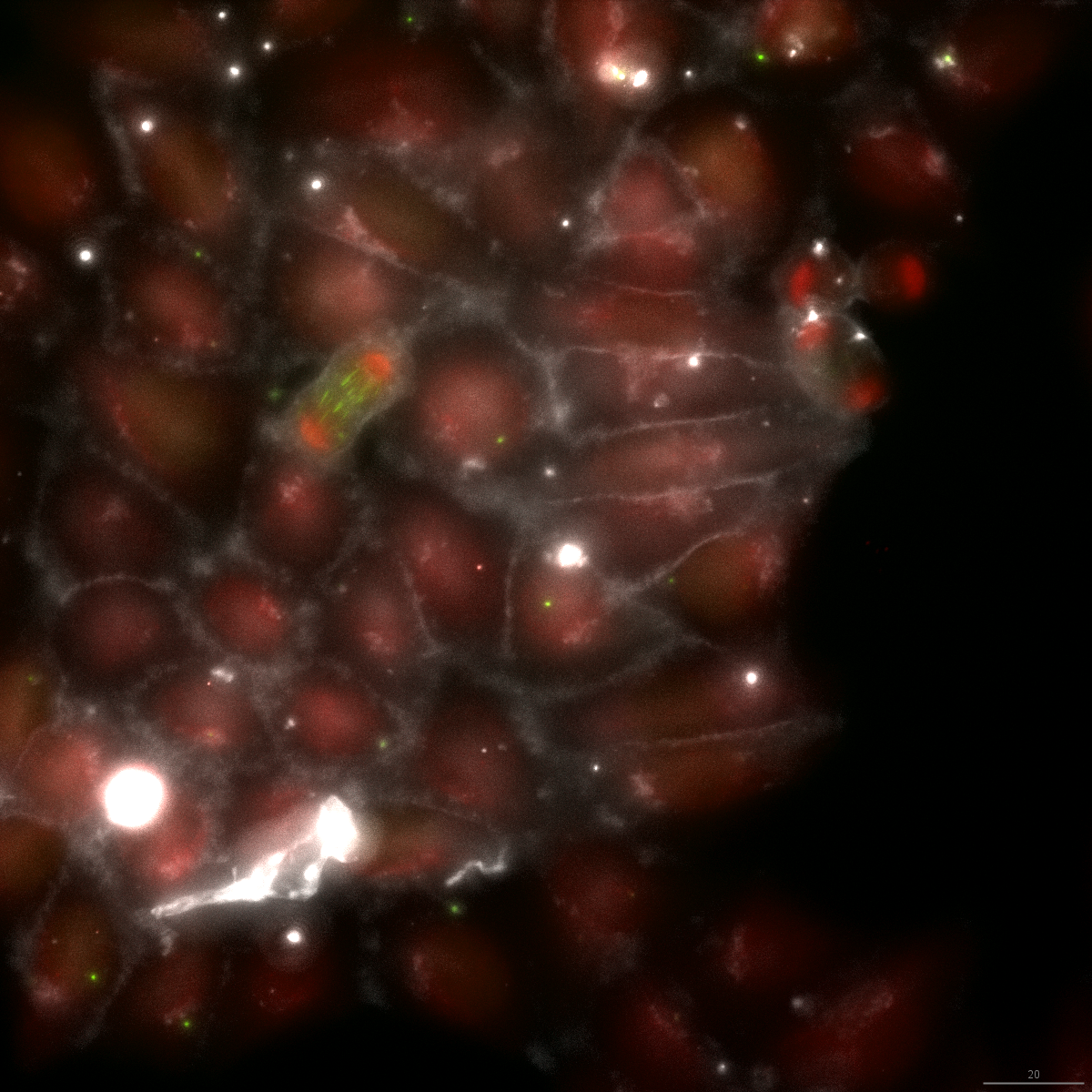

Supplement: Supplementary file 10 — Source Data for Figure 7 [file EMBJ-42-e113647-s009.zip › Figure 7/Figure 7G/uncropped MEE/4 min/PRC1MEEto3Amerge.tif]

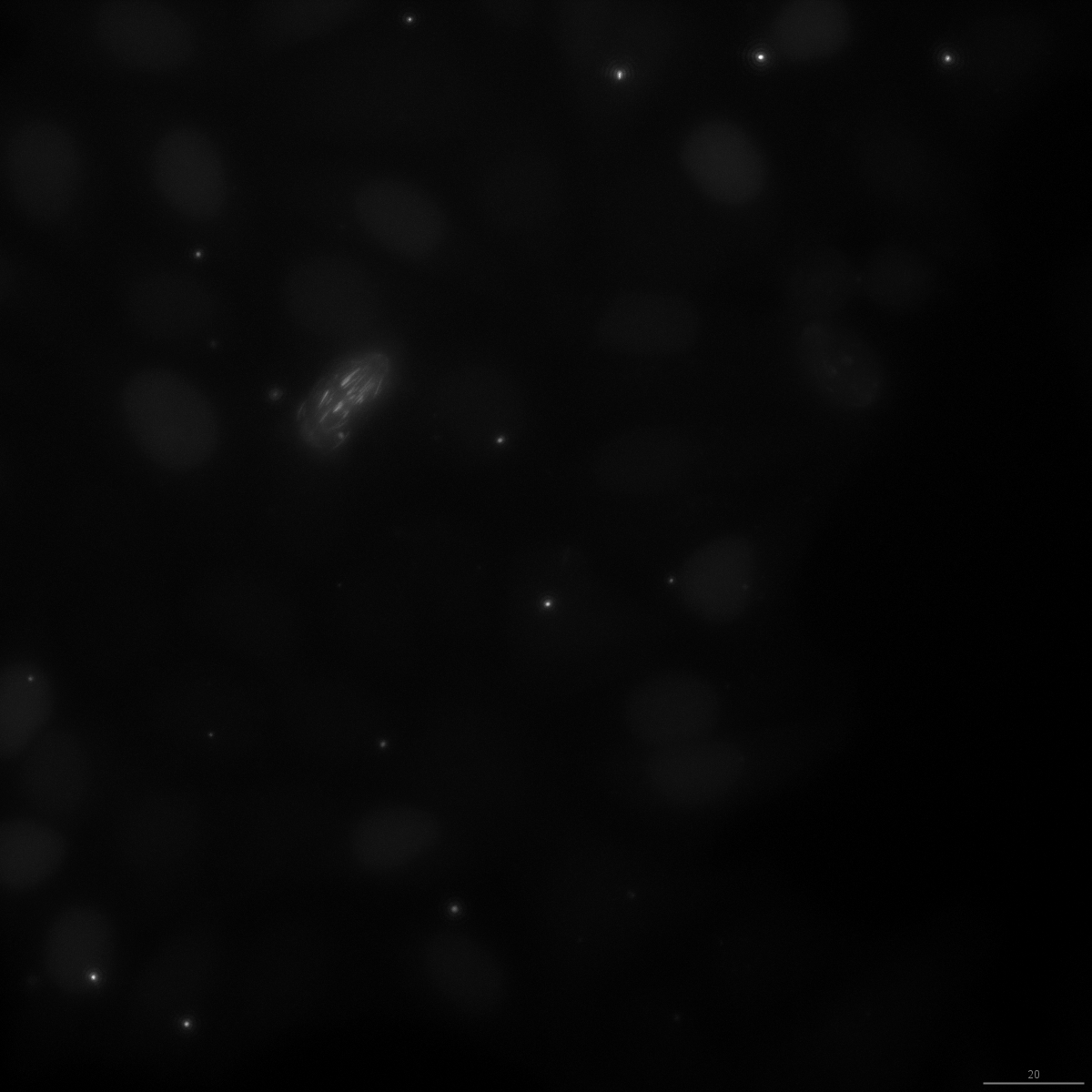

Supplement: Supplementary file 10 — Source Data for Figure 7 [file EMBJ-42-e113647-s009.zip › Figure 7/Figure 7G/uncropped MEE/4 min/PRC1MEEto3AGFP.tif]

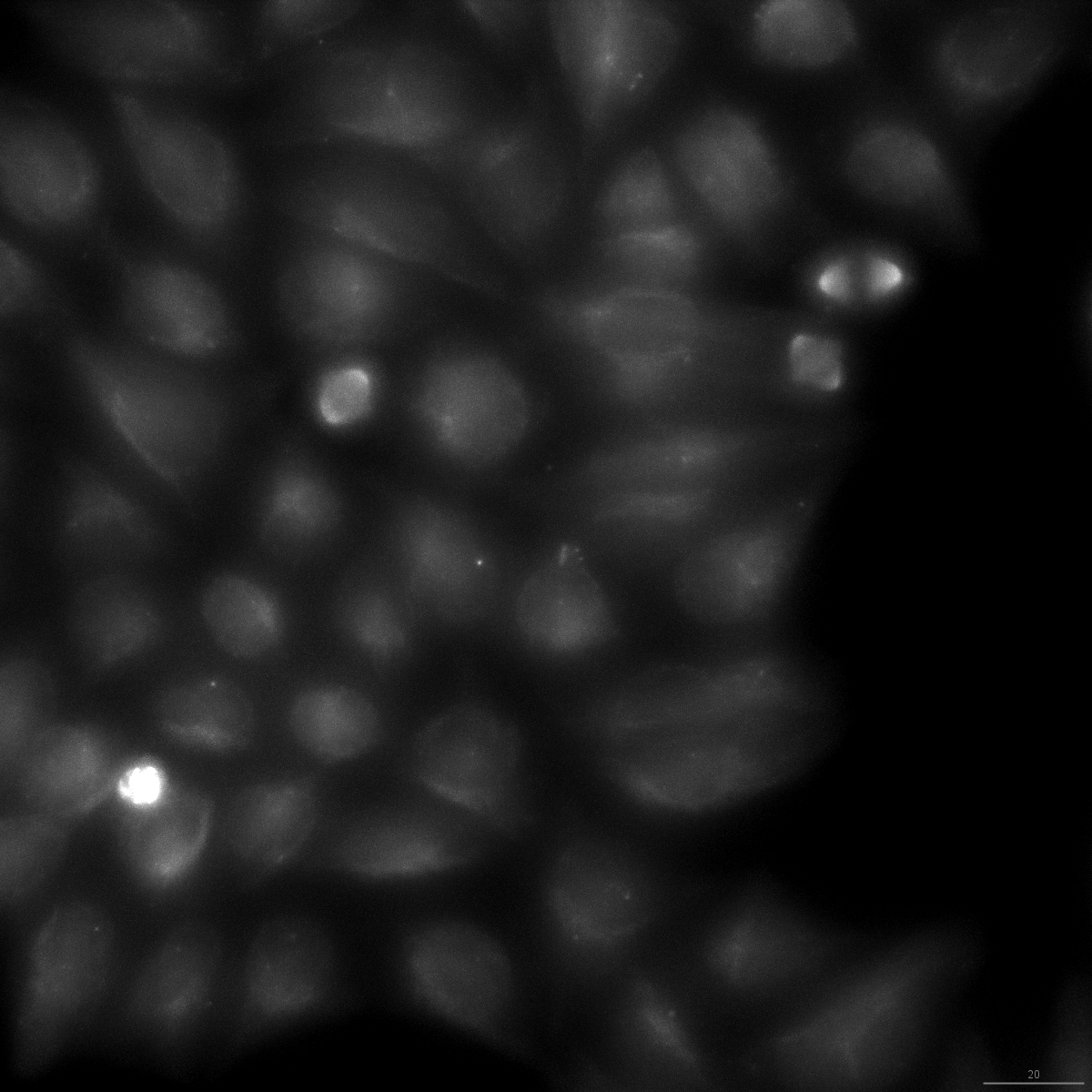

Supplement: Supplementary file 10 — Source Data for Figure 7 [file EMBJ-42-e113647-s009.zip › Figure 7/Figure 7G/uncropped MEE/-2 min/PRC1MEEto3AMT.tif]

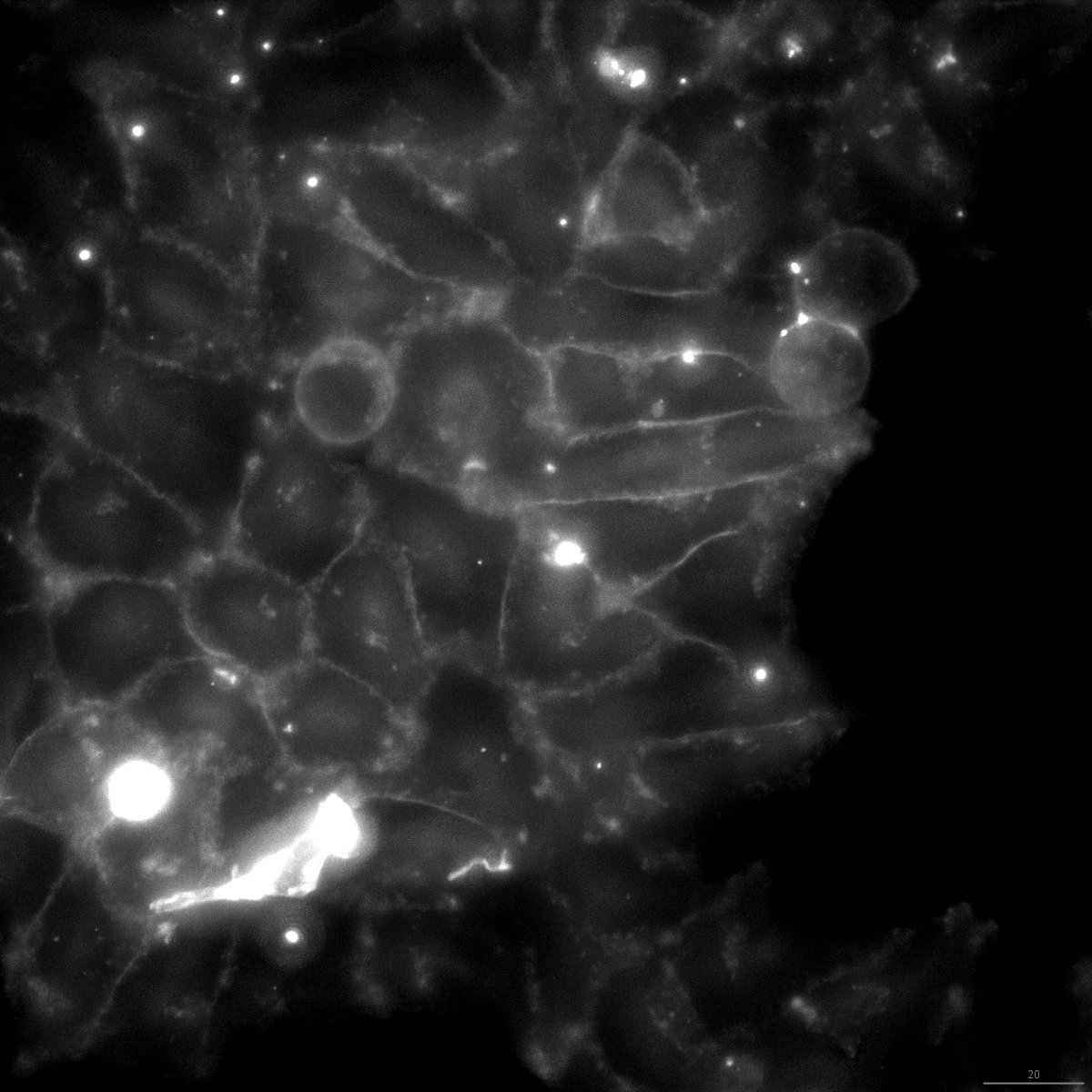

Supplement: Supplementary file 10 — Source Data for Figure 7 [file EMBJ-42-e113647-s009.zip › Figure 7/Figure 7G/uncropped MEE/-2 min/PRC1MEEto3AMembrane.tif]

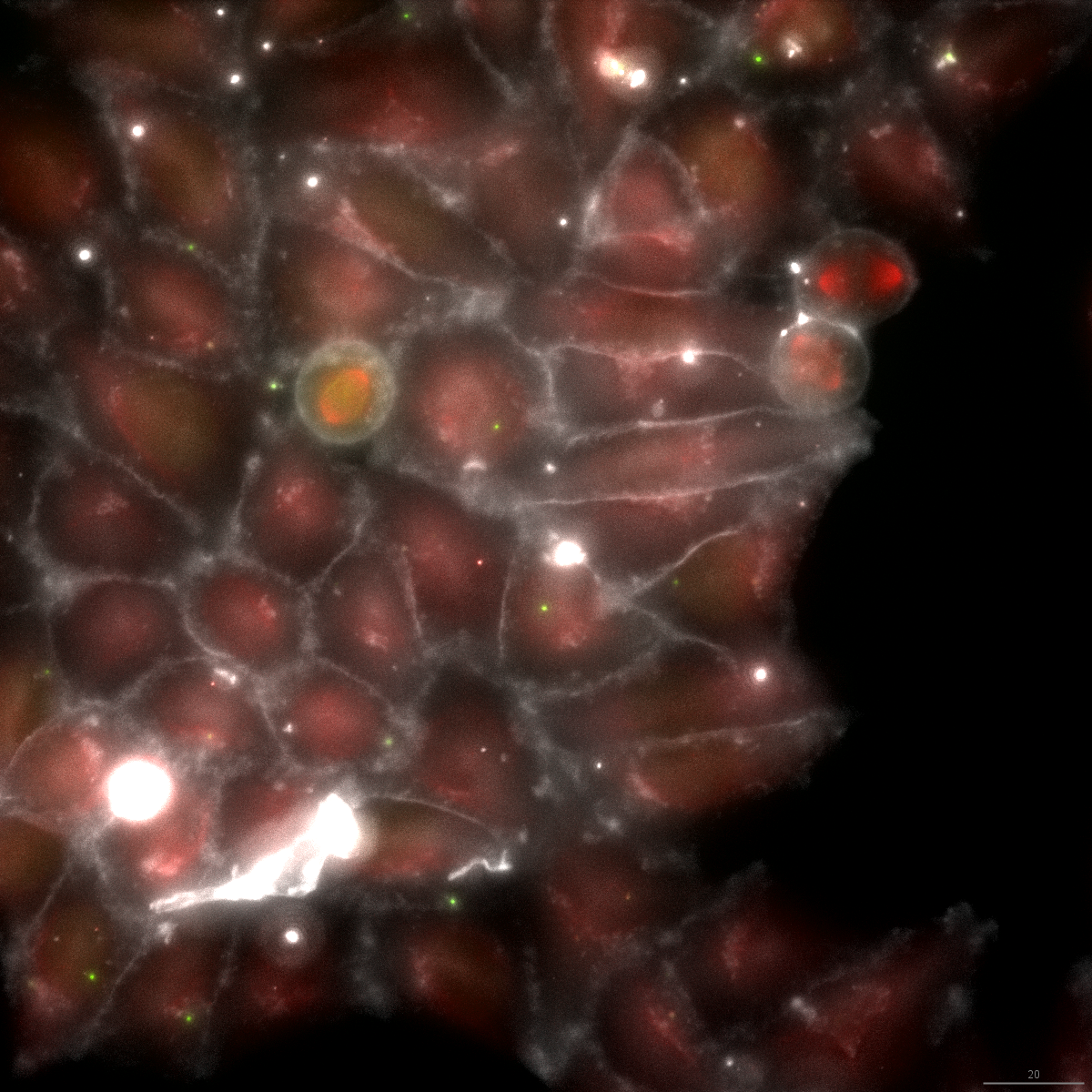

Supplement: Supplementary file 10 — Source Data for Figure 7 [file EMBJ-42-e113647-s009.zip › Figure 7/Figure 7G/uncropped MEE/-2 min/PRC1MEEto3Amerge.tif]

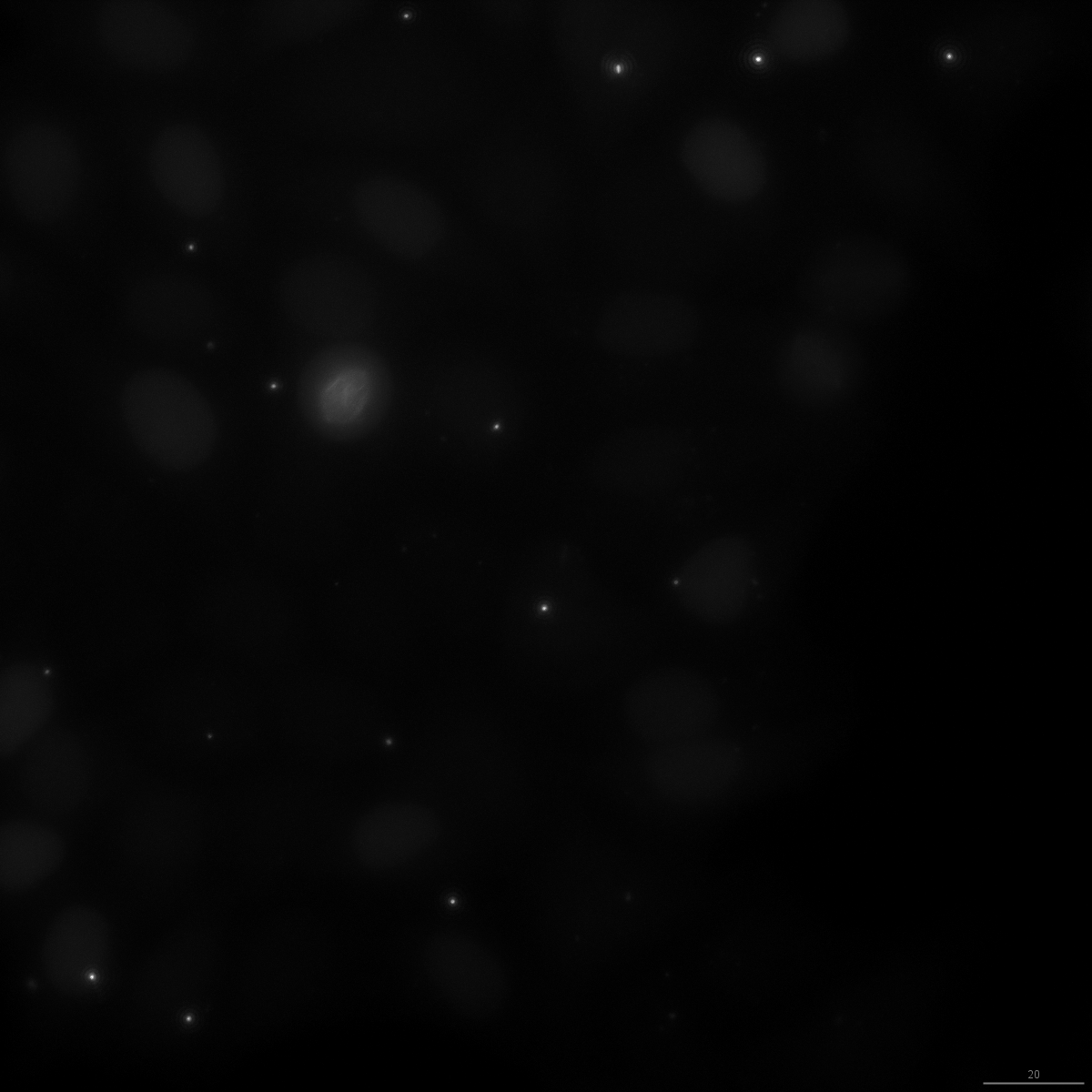

Supplement: Supplementary file 10 — Source Data for Figure 7 [file EMBJ-42-e113647-s009.zip › Figure 7/Figure 7G/uncropped MEE/-2 min/PRC1MEEto3AGFP.tif]

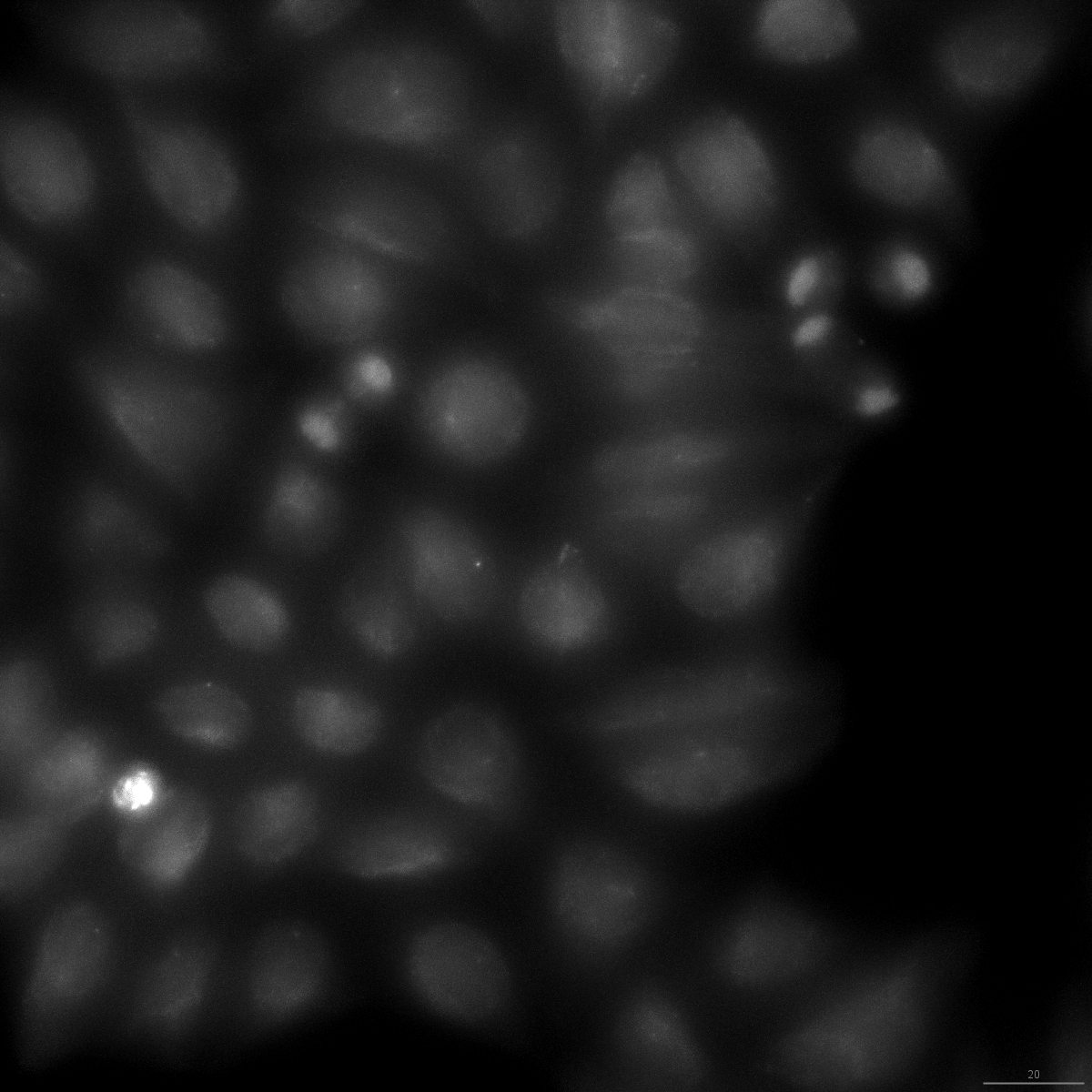

Supplement: Supplementary file 10 — Source Data for Figure 7 [file EMBJ-42-e113647-s009.zip › Figure 7/Figure 7G/uncropped MEE/6 min/PRC1MEEto3AMT.tif]

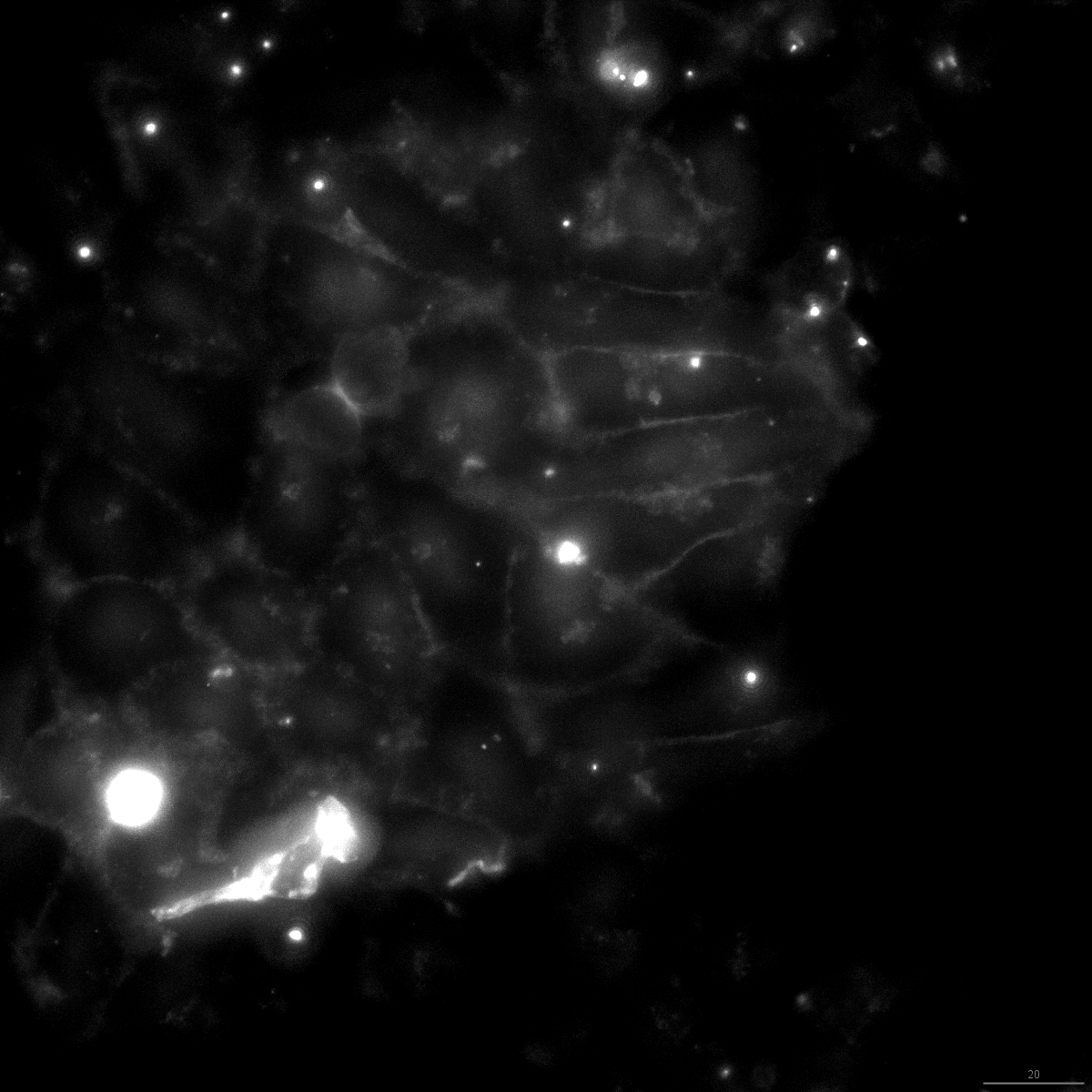

Supplement: Supplementary file 10 — Source Data for Figure 7 [file EMBJ-42-e113647-s009.zip › Figure 7/Figure 7G/uncropped MEE/6 min/PRC1MEEto3AMembrane.tif]

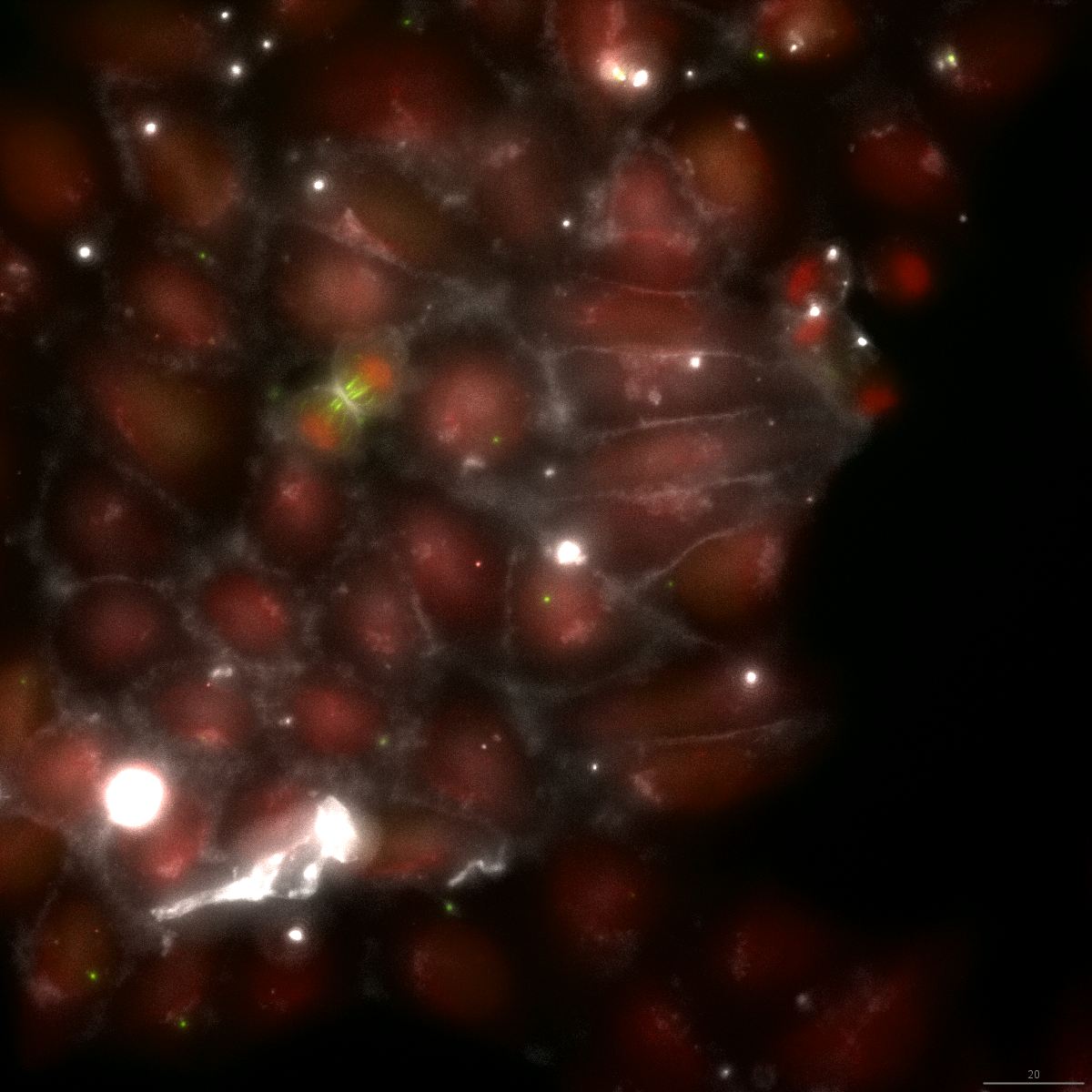

Supplement: Supplementary file 10 — Source Data for Figure 7 [file EMBJ-42-e113647-s009.zip › Figure 7/Figure 7G/uncropped MEE/6 min/PRC1MEEto3Amerge.tif]

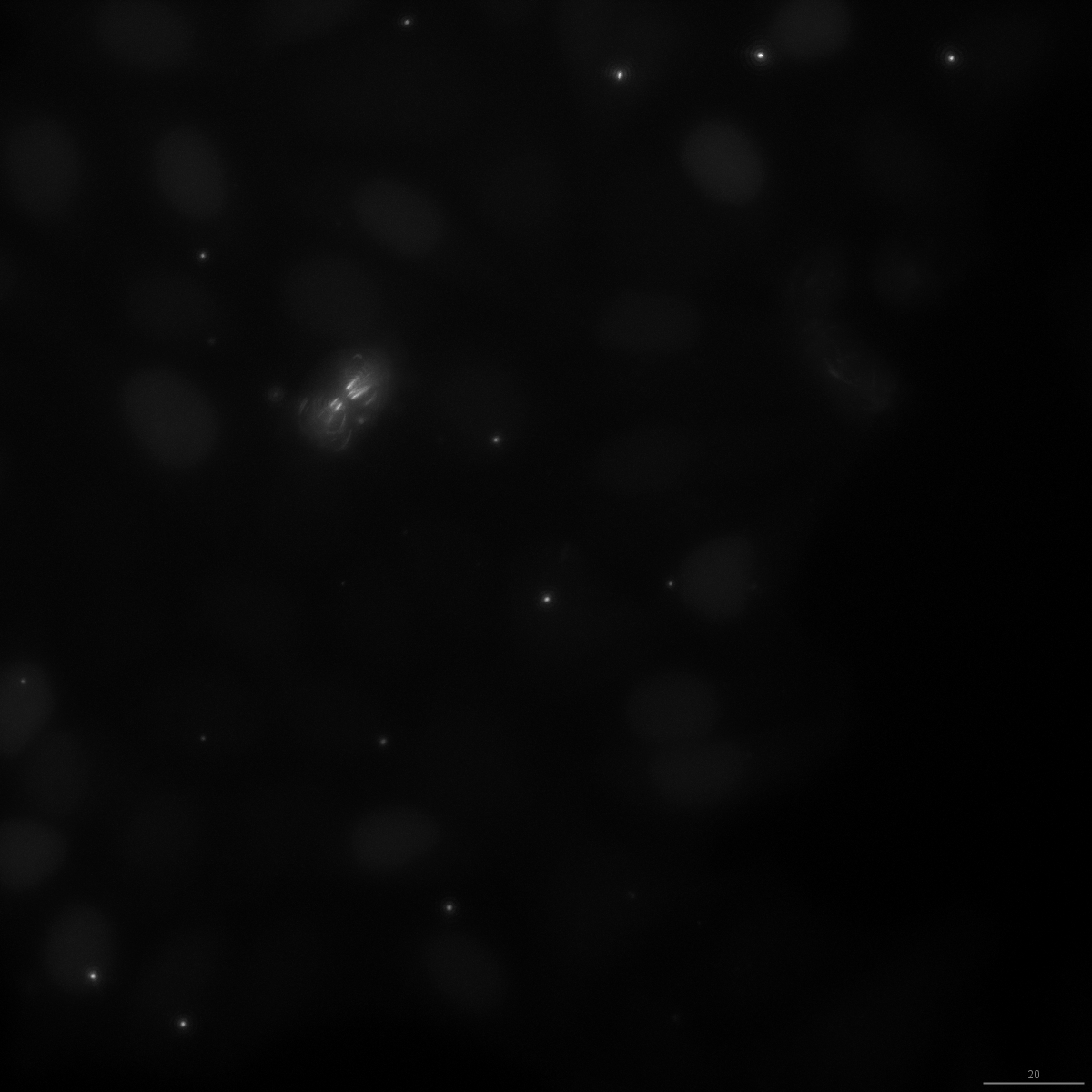

Supplement: Supplementary file 10 — Source Data for Figure 7 [file EMBJ-42-e113647-s009.zip › Figure 7/Figure 7G/uncropped MEE/6 min/PRC1MEEto3AGFP.tif]

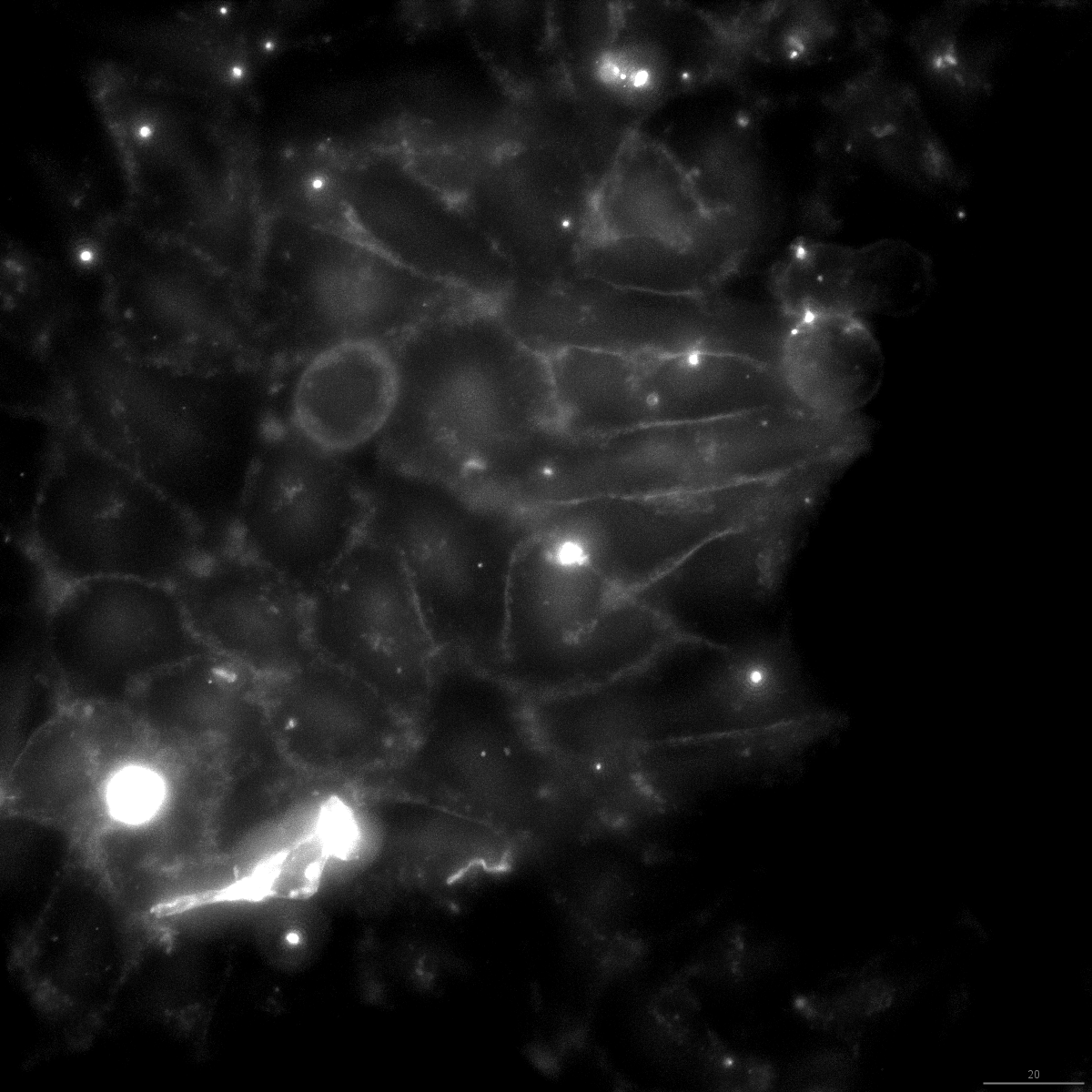

Supplement: Supplementary file 10 — Source Data for Figure 7 [file EMBJ-42-e113647-s009.zip › Figure 7/Figure 7G/uncropped MEE/2 min/PRC1MEEto3AMembrane.tif]

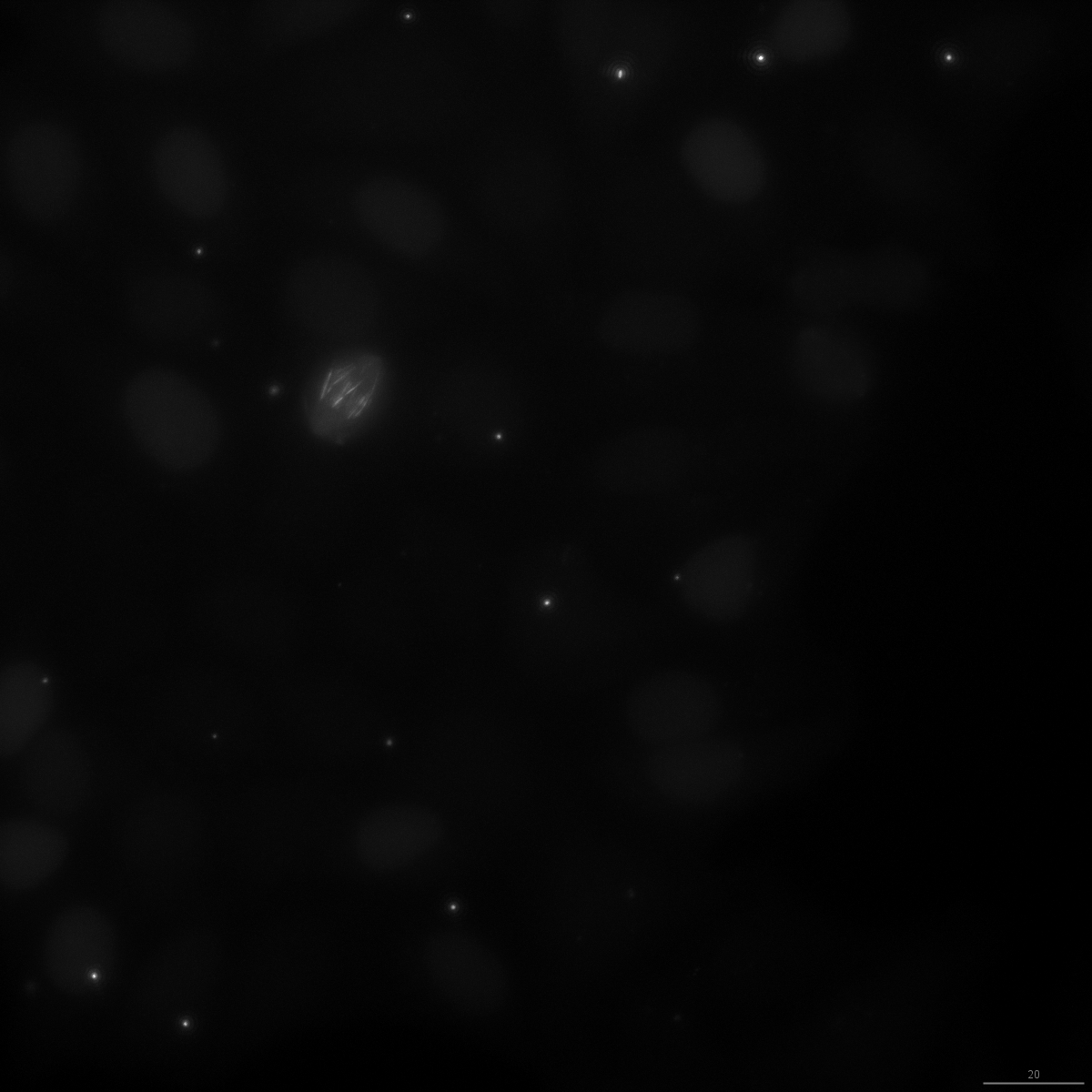

Supplement: Supplementary file 10 — Source Data for Figure 7 [file EMBJ-42-e113647-s009.zip › Figure 7/Figure 7G/uncropped MEE/2 min/PRC1MEEGFP.tif]

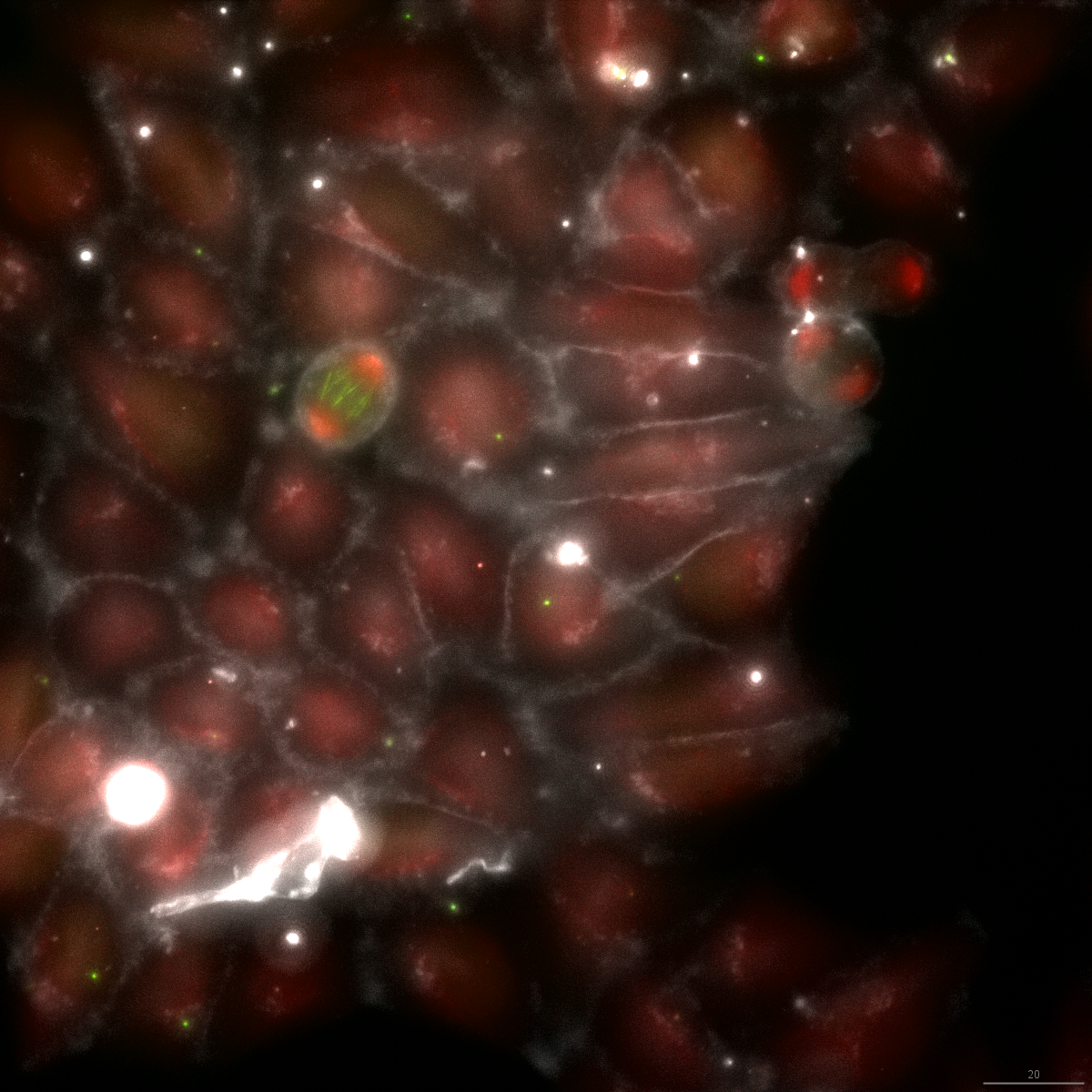

Supplement: Supplementary file 10 — Source Data for Figure 7 [file EMBJ-42-e113647-s009.zip › Figure 7/Figure 7G/uncropped MEE/2 min/PRC1MEEmerge.tif]

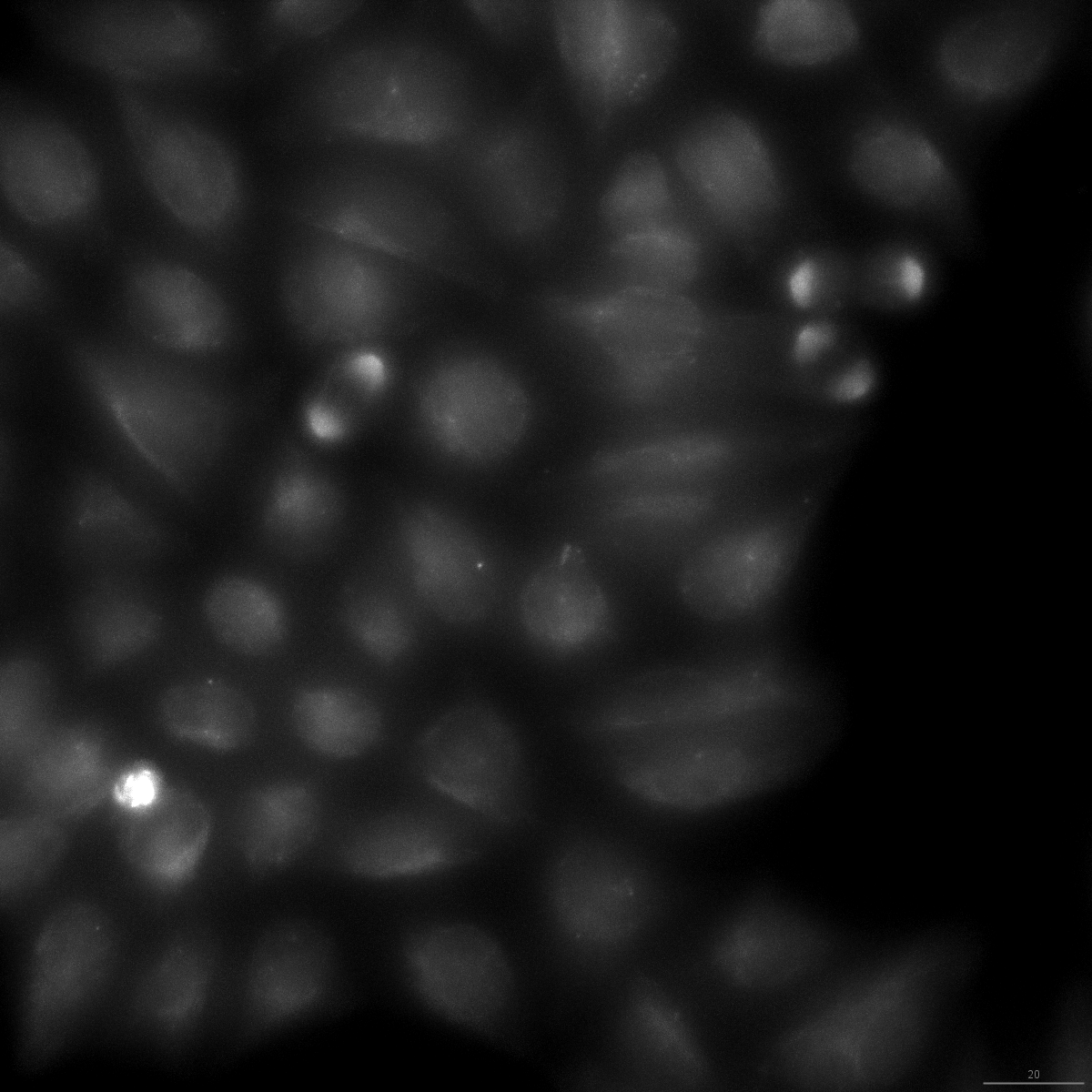

Supplement: Supplementary file 10 — Source Data for Figure 7 [file EMBJ-42-e113647-s009.zip › Figure 7/Figure 7G/uncropped MEE/2 min/PRC1MEEMT.tif]

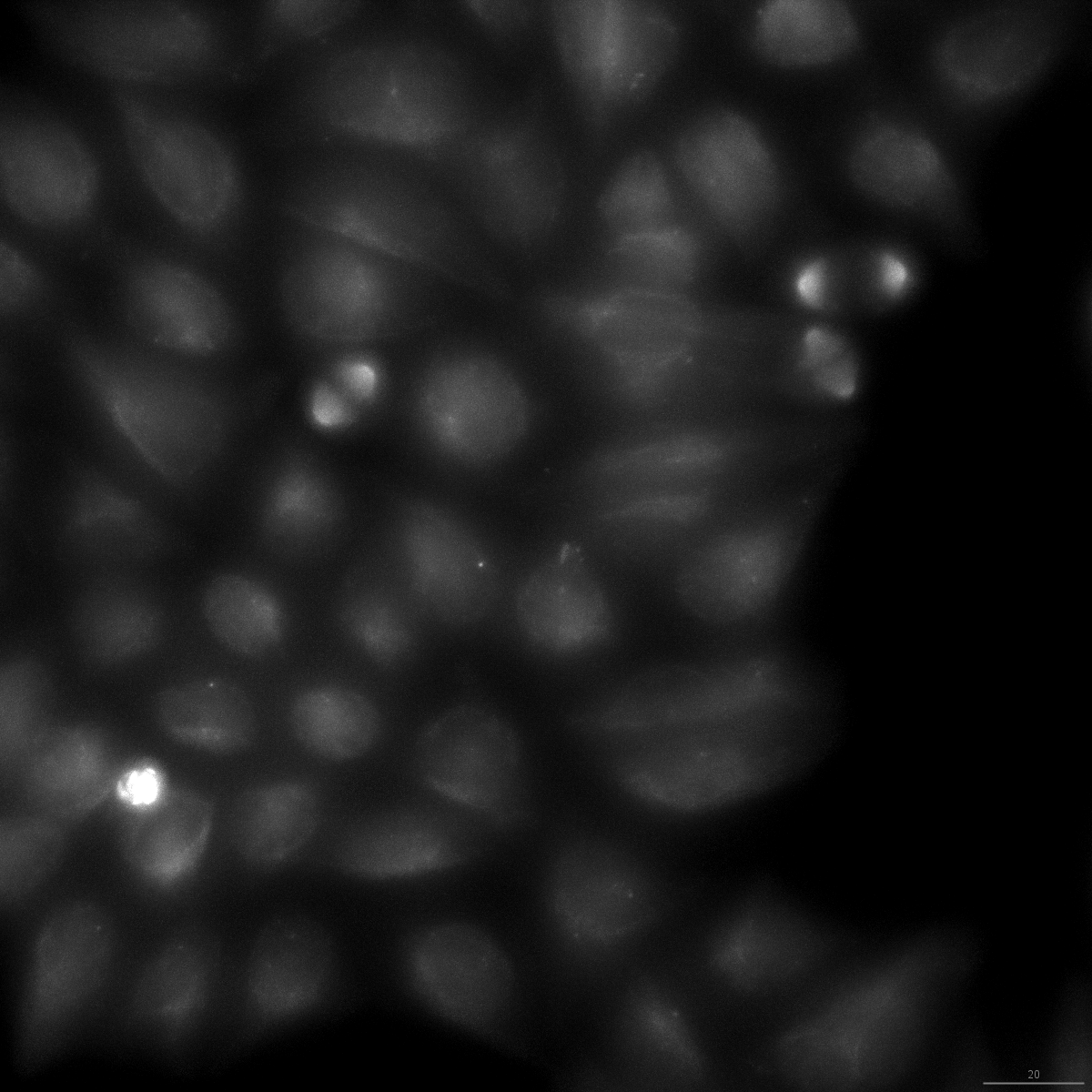

Supplement: Supplementary file 10 — Source Data for Figure 7 [file EMBJ-42-e113647-s009.zip › Figure 7/Figure 7G/uncropped MEE/0 min/PRC1MEEto3AMT.tif]

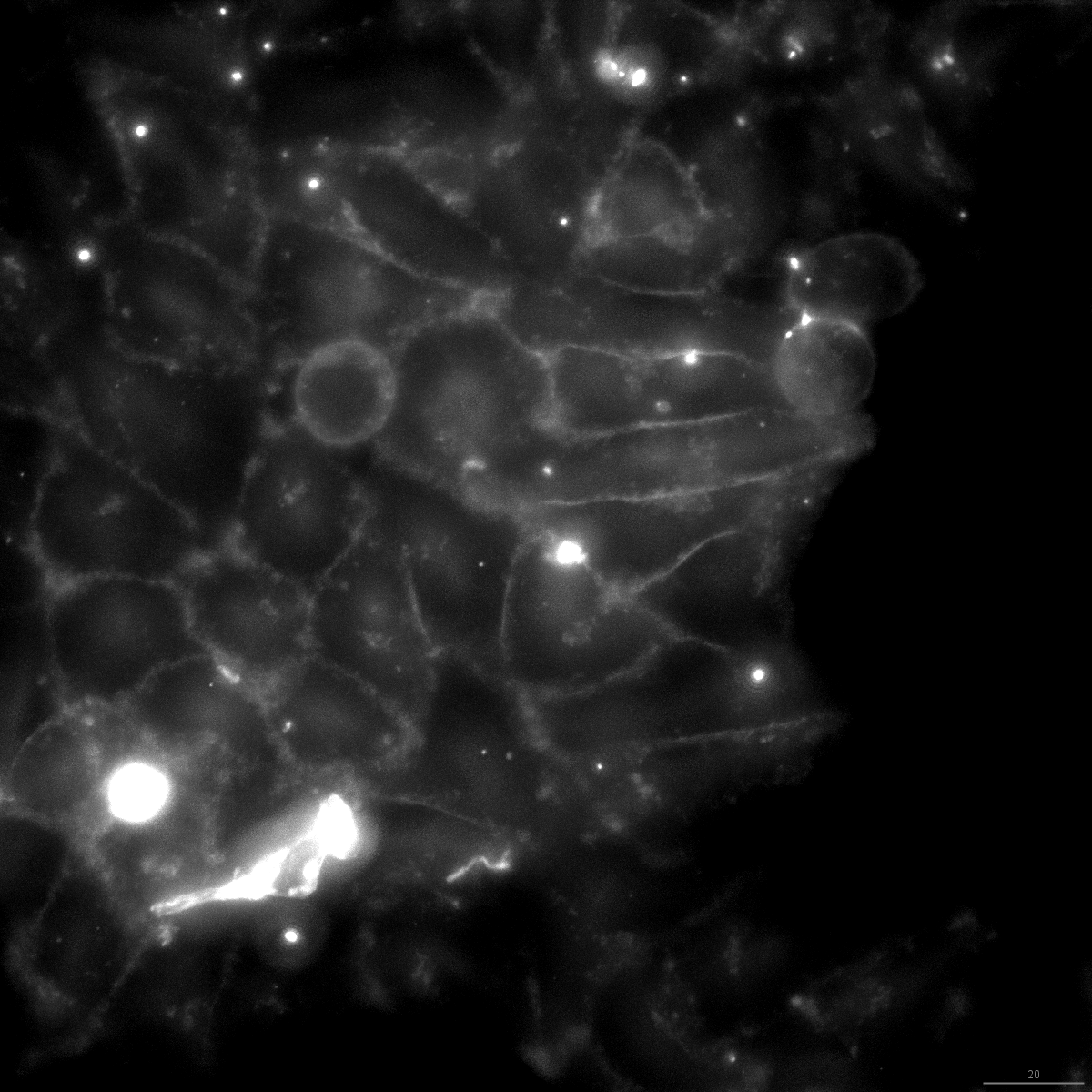

Supplement: Supplementary file 10 — Source Data for Figure 7 [file EMBJ-42-e113647-s009.zip › Figure 7/Figure 7G/uncropped MEE/0 min/PRC1MEEto3AMembrane.tif]

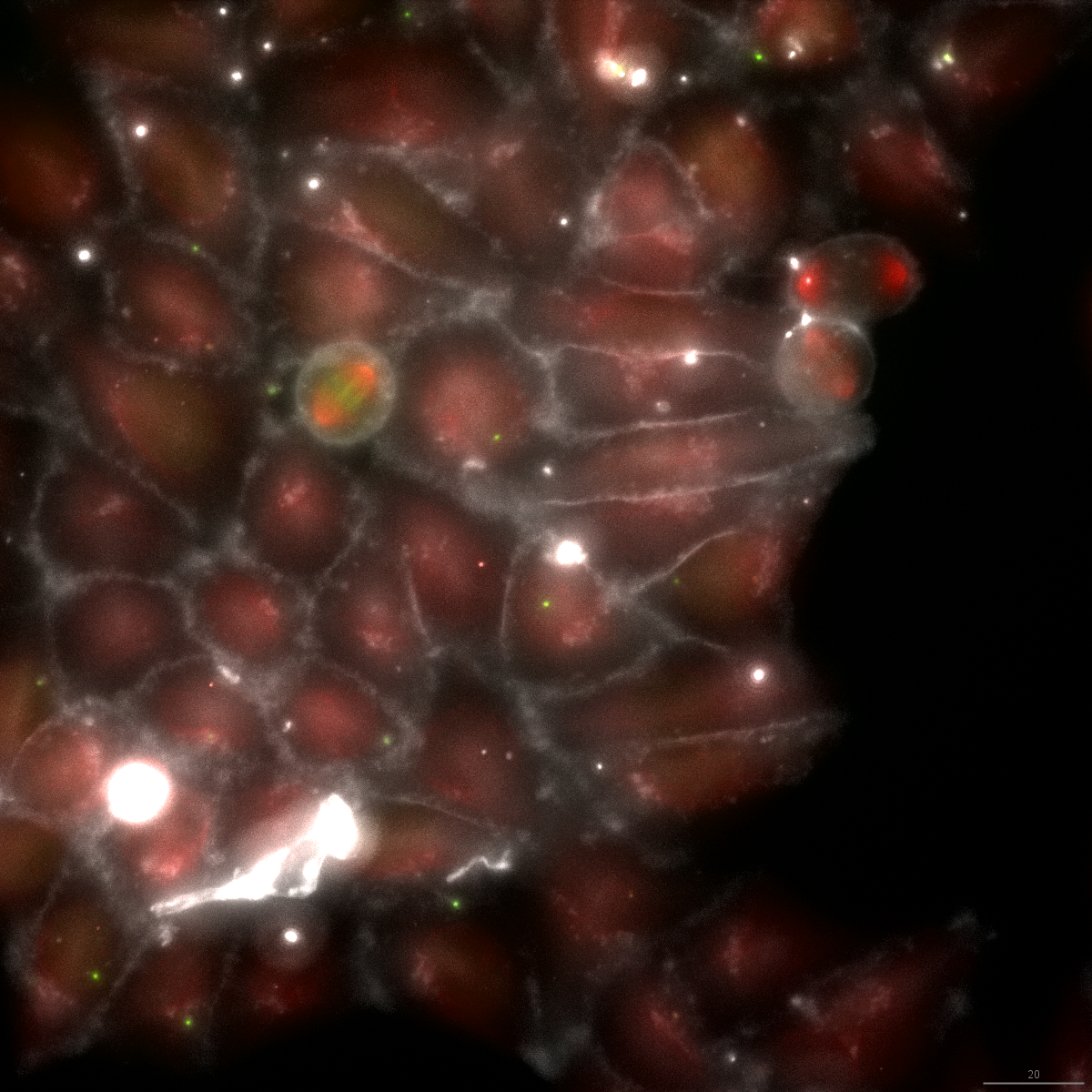

Supplement: Supplementary file 10 — Source Data for Figure 7 [file EMBJ-42-e113647-s009.zip › Figure 7/Figure 7G/uncropped MEE/0 min/PRC1MEEto3Amerge.tif]

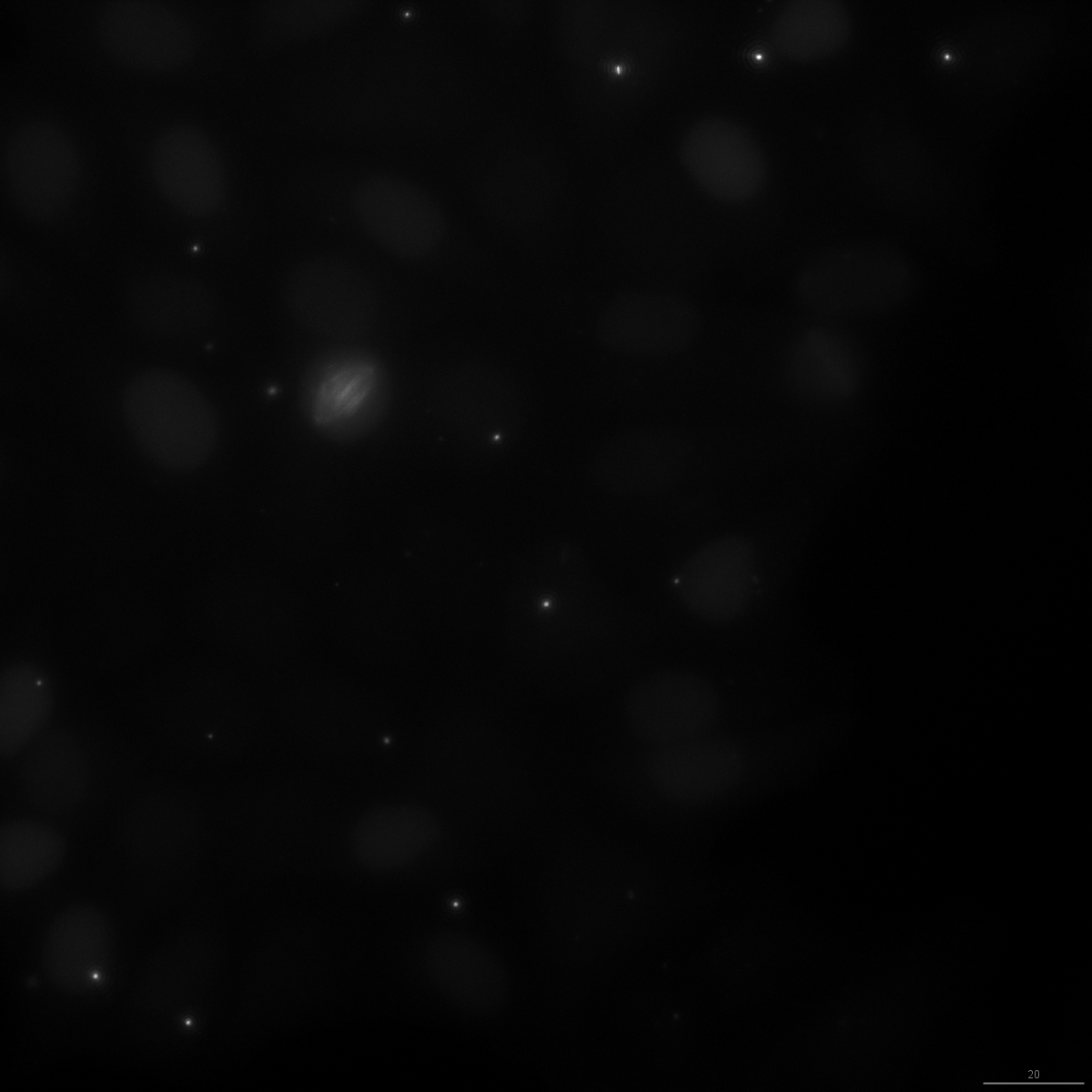

Supplement: Supplementary file 10 — Source Data for Figure 7 [file EMBJ-42-e113647-s009.zip › Figure 7/Figure 7G/uncropped MEE/0 min/PRC1MEEto3AGFP.tif]

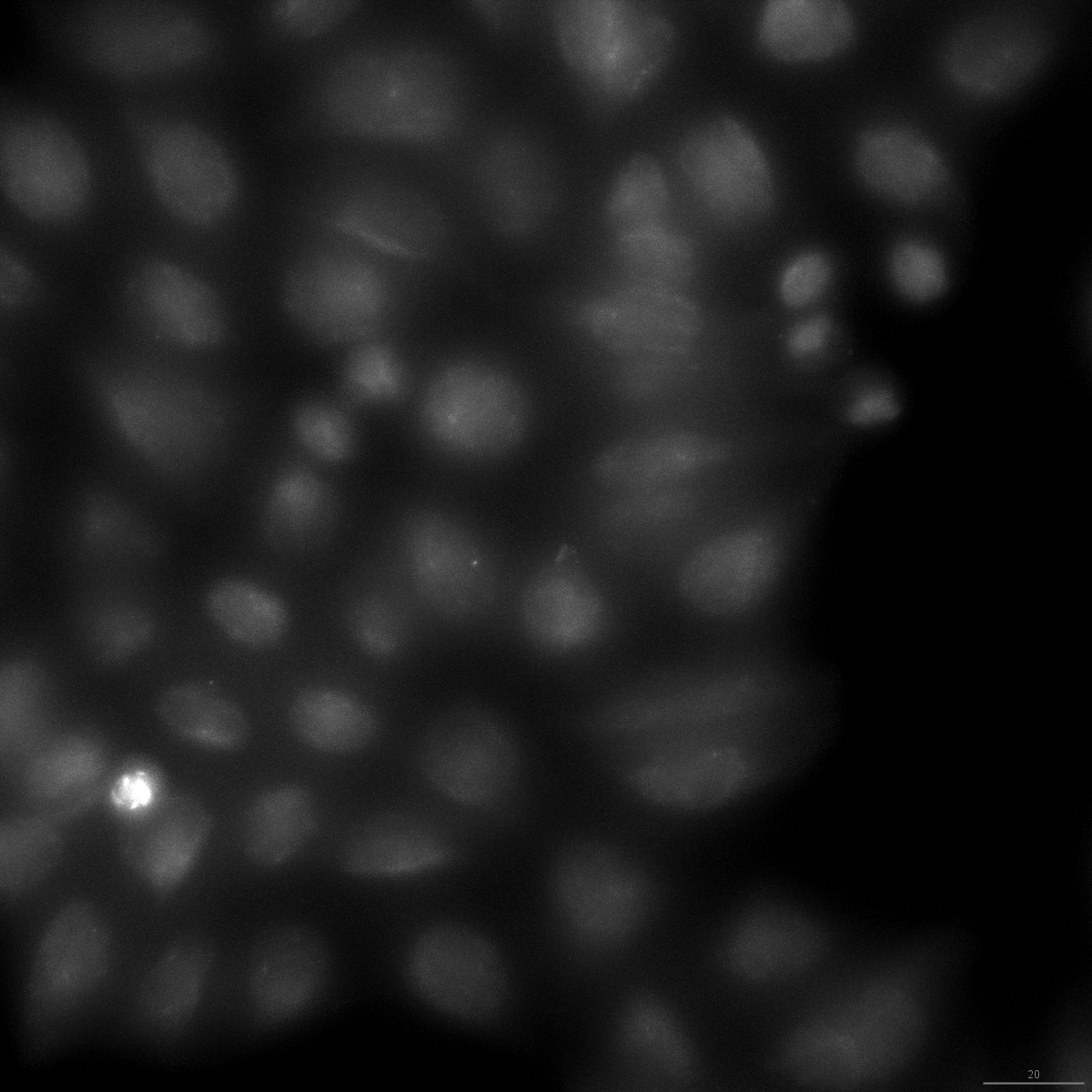

Supplement: Supplementary file 10 — Source Data for Figure 7 [file EMBJ-42-e113647-s009.zip › Figure 7/Figure 7G/uncropped MEE/10 min/PRC1MEEto3AMT.tif]

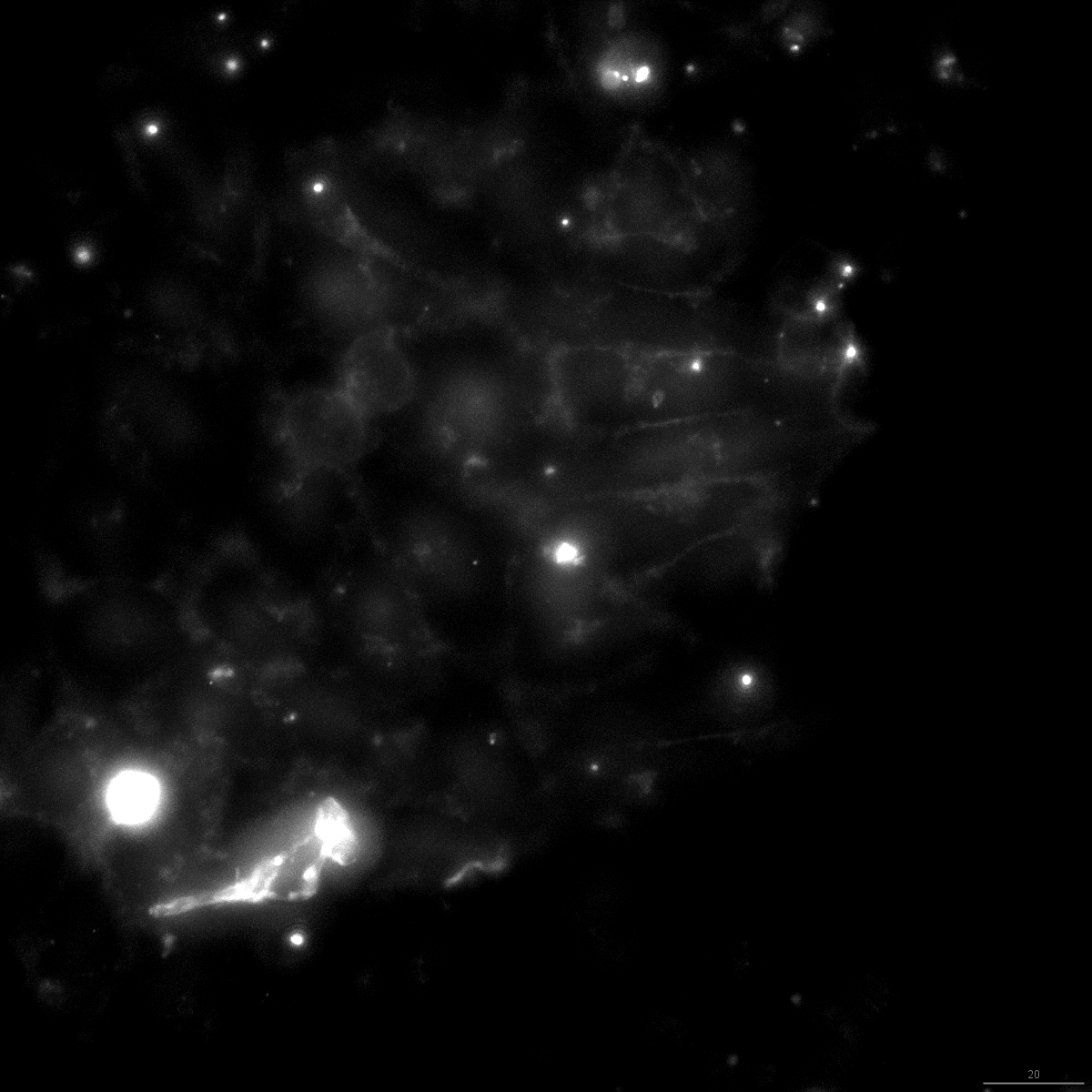

Supplement: Supplementary file 10 — Source Data for Figure 7 [file EMBJ-42-e113647-s009.zip › Figure 7/Figure 7G/uncropped MEE/10 min/PRC1MEEto3AMembrane.tif]

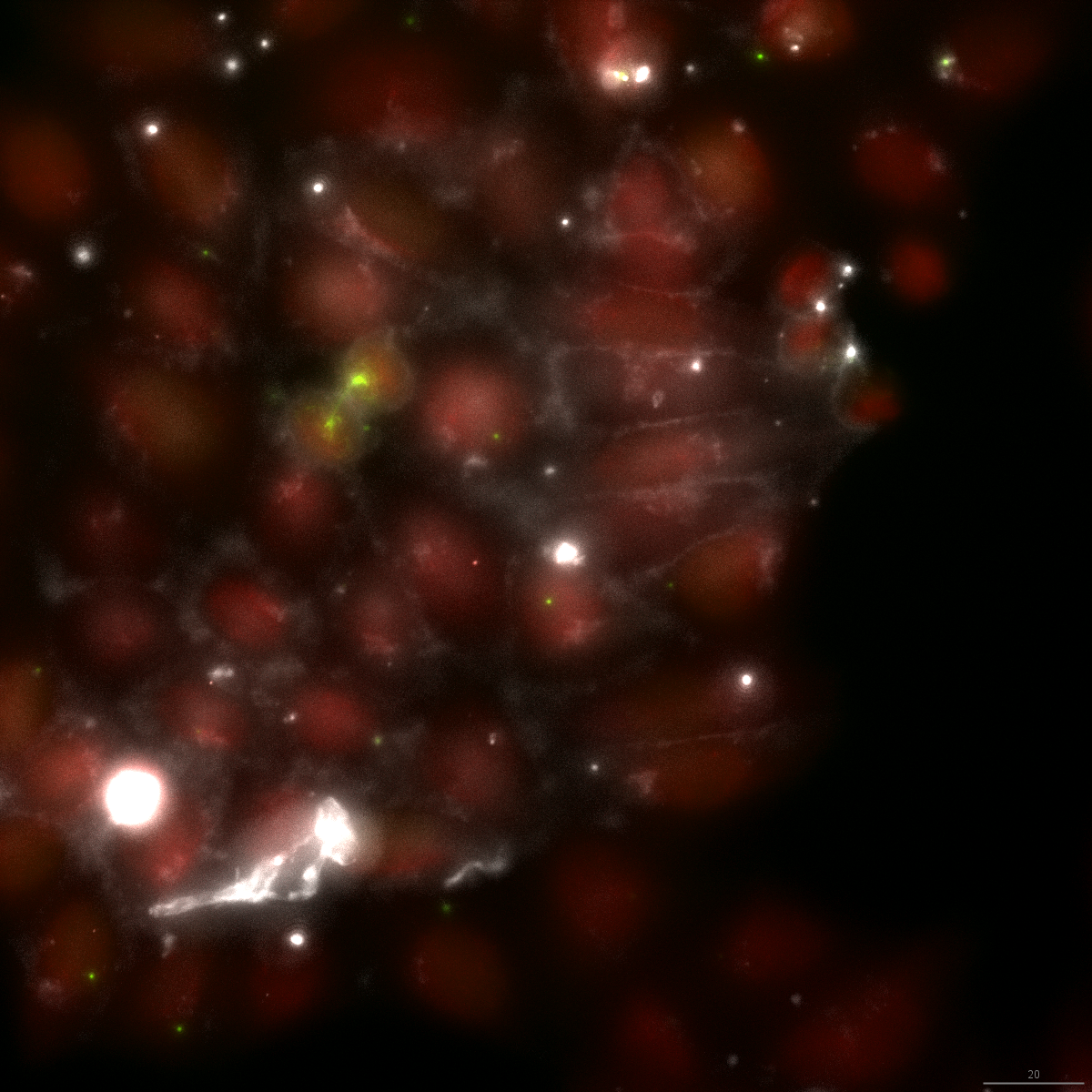

Supplement: Supplementary file 10 — Source Data for Figure 7 [file EMBJ-42-e113647-s009.zip › Figure 7/Figure 7G/uncropped MEE/10 min/PRC1MEEto3Amerge.tif]

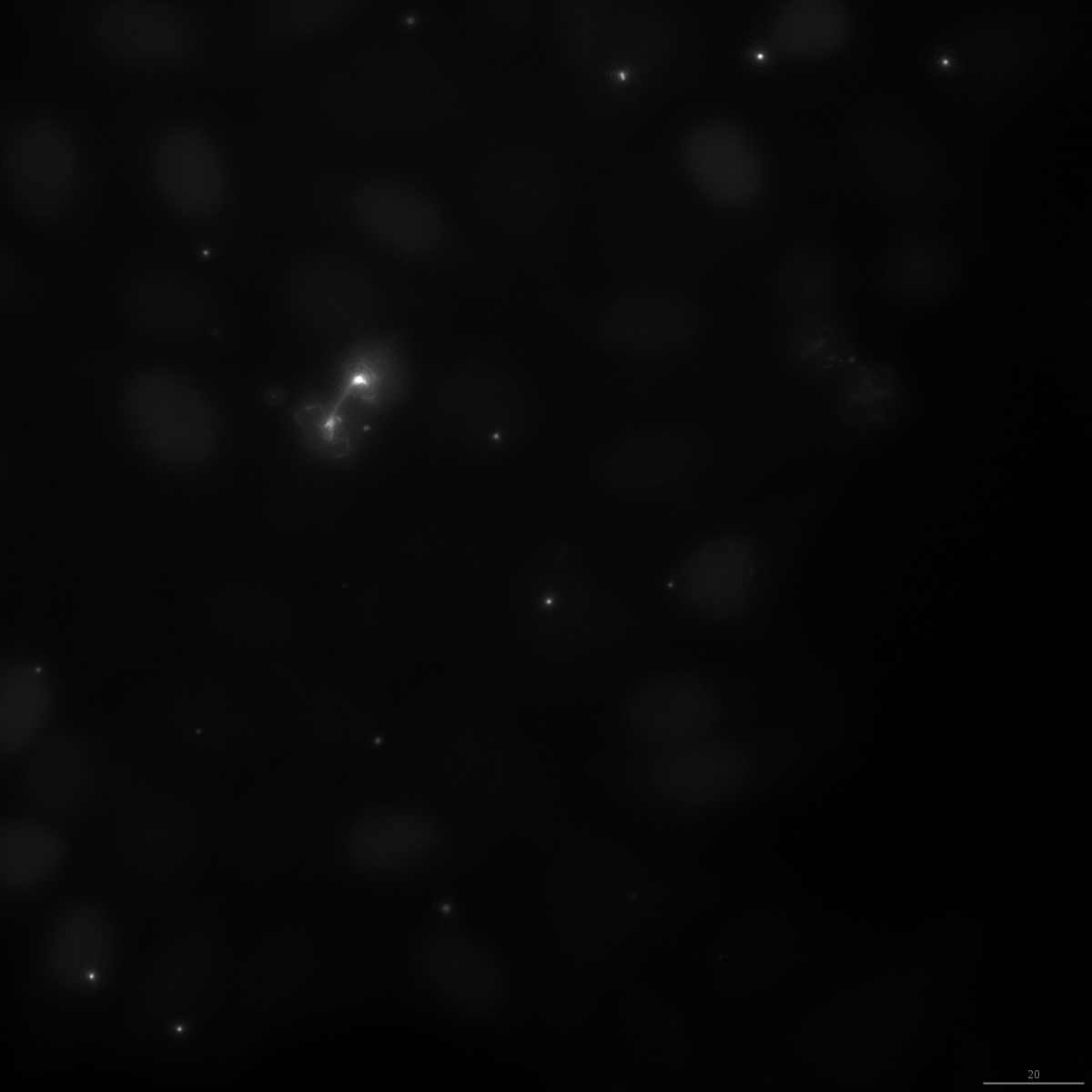

Supplement: Supplementary file 10 — Source Data for Figure 7 [file EMBJ-42-e113647-s009.zip › Figure 7/Figure 7G/uncropped MEE/10 min/PRC1MEEto3AGFP.tif]

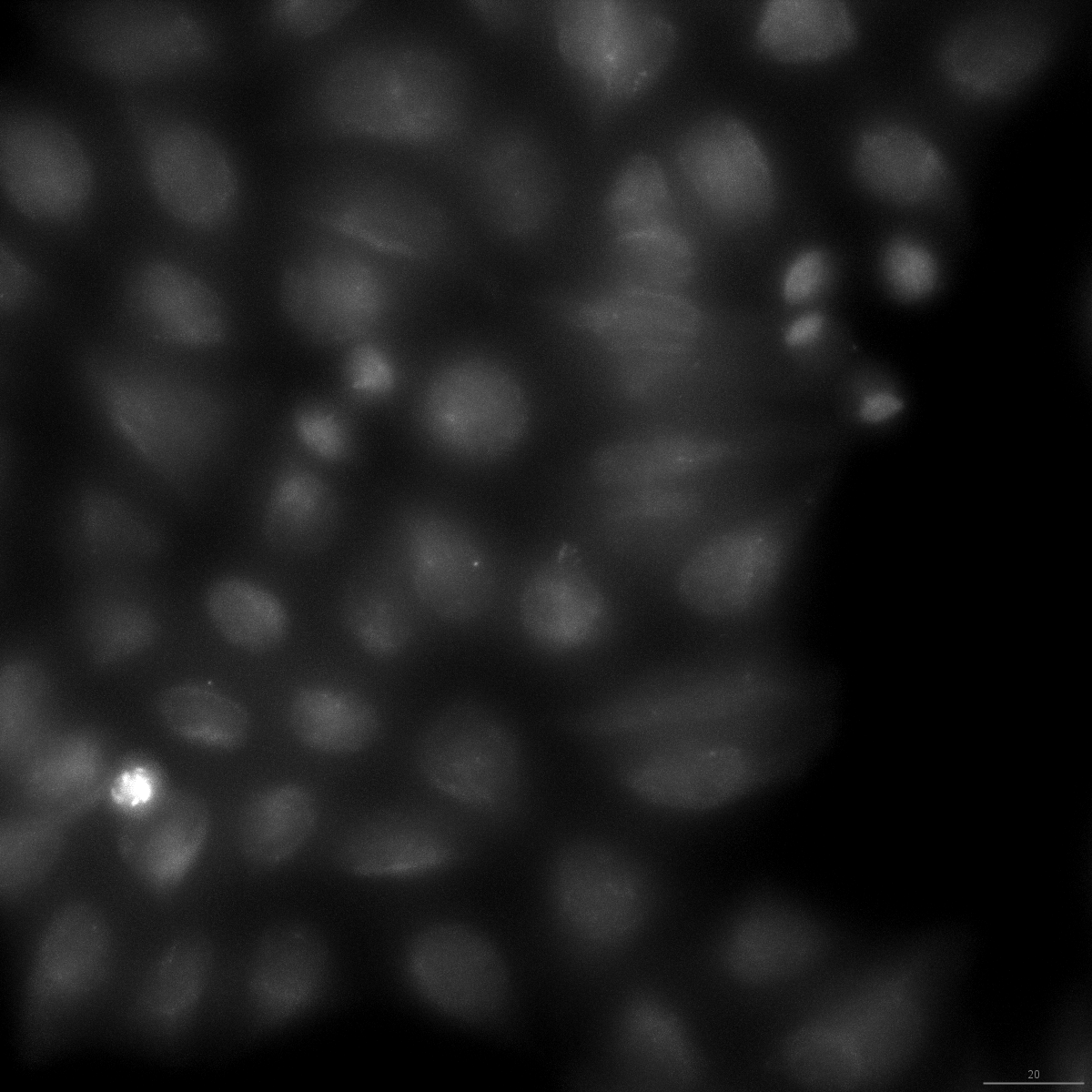

Supplement: Supplementary file 10 — Source Data for Figure 7 [file EMBJ-42-e113647-s009.zip › Figure 7/Figure 7G/uncropped MEE/8 min/PRC1MEEto3AMT.tif]

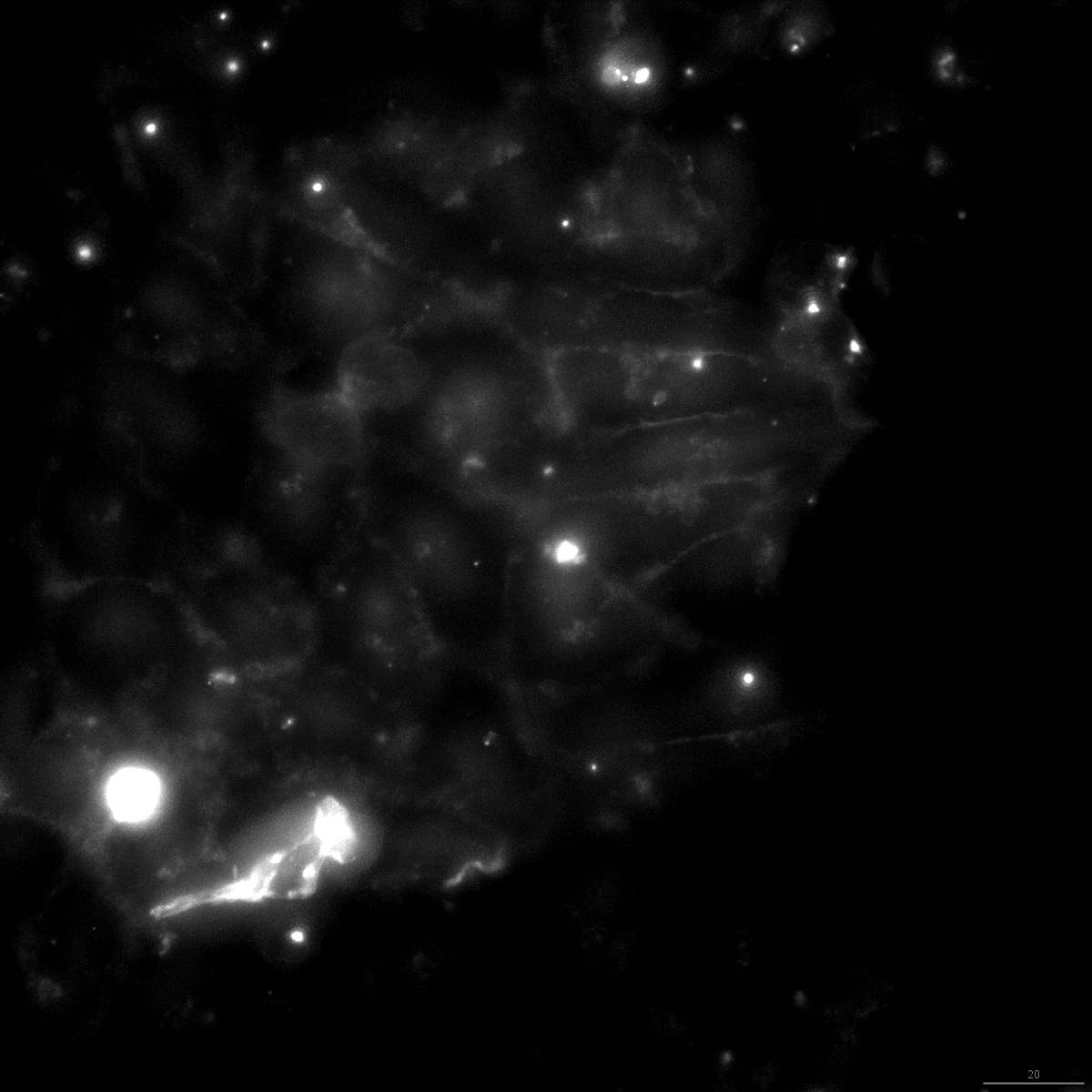

Supplement: Supplementary file 10 — Source Data for Figure 7 [file EMBJ-42-e113647-s009.zip › Figure 7/Figure 7G/uncropped MEE/8 min/PRC1MEEto3AMembrane.tif]

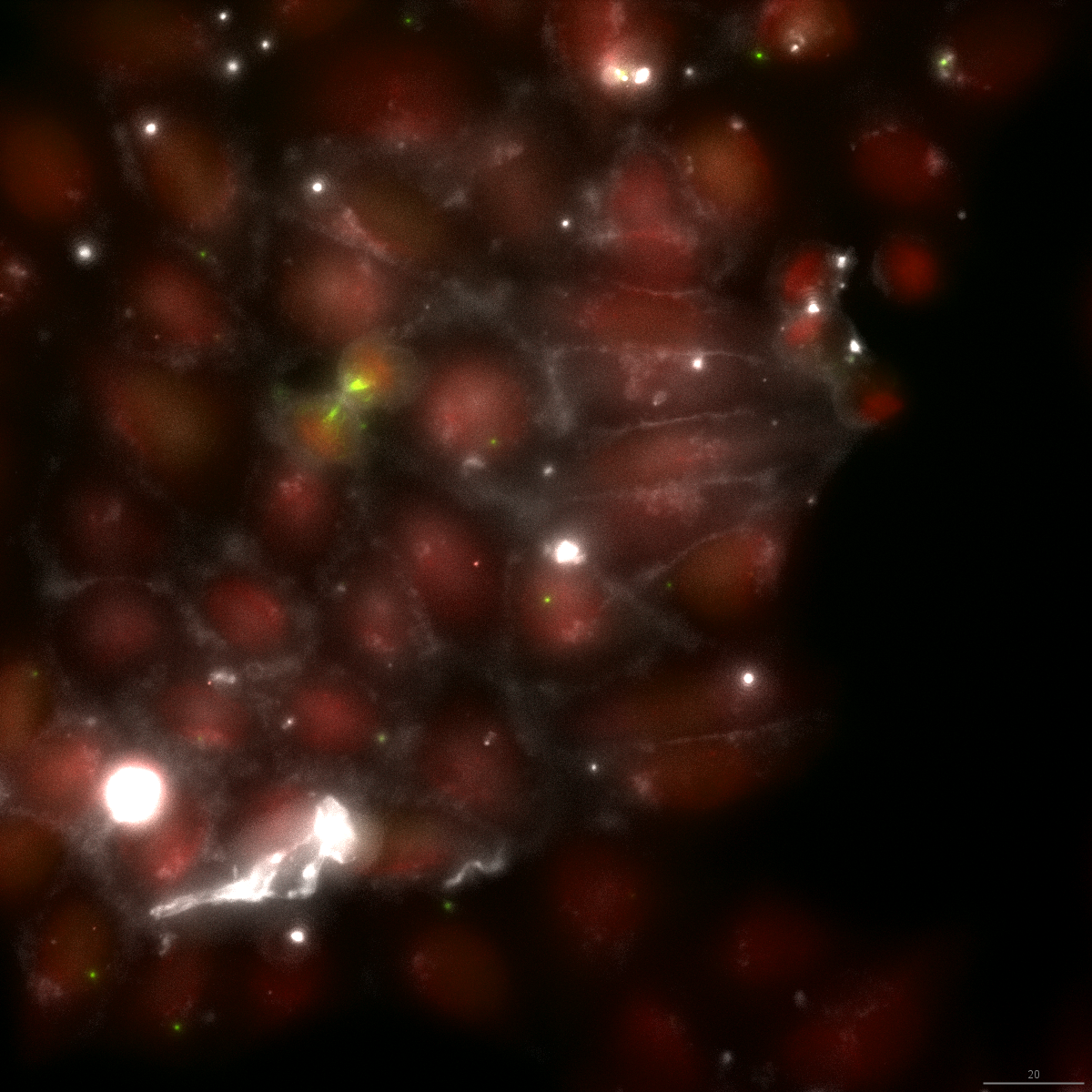

Supplement: Supplementary file 10 — Source Data for Figure 7 [file EMBJ-42-e113647-s009.zip › Figure 7/Figure 7G/uncropped MEE/8 min/PRC1MEEto3Amerge.tif]

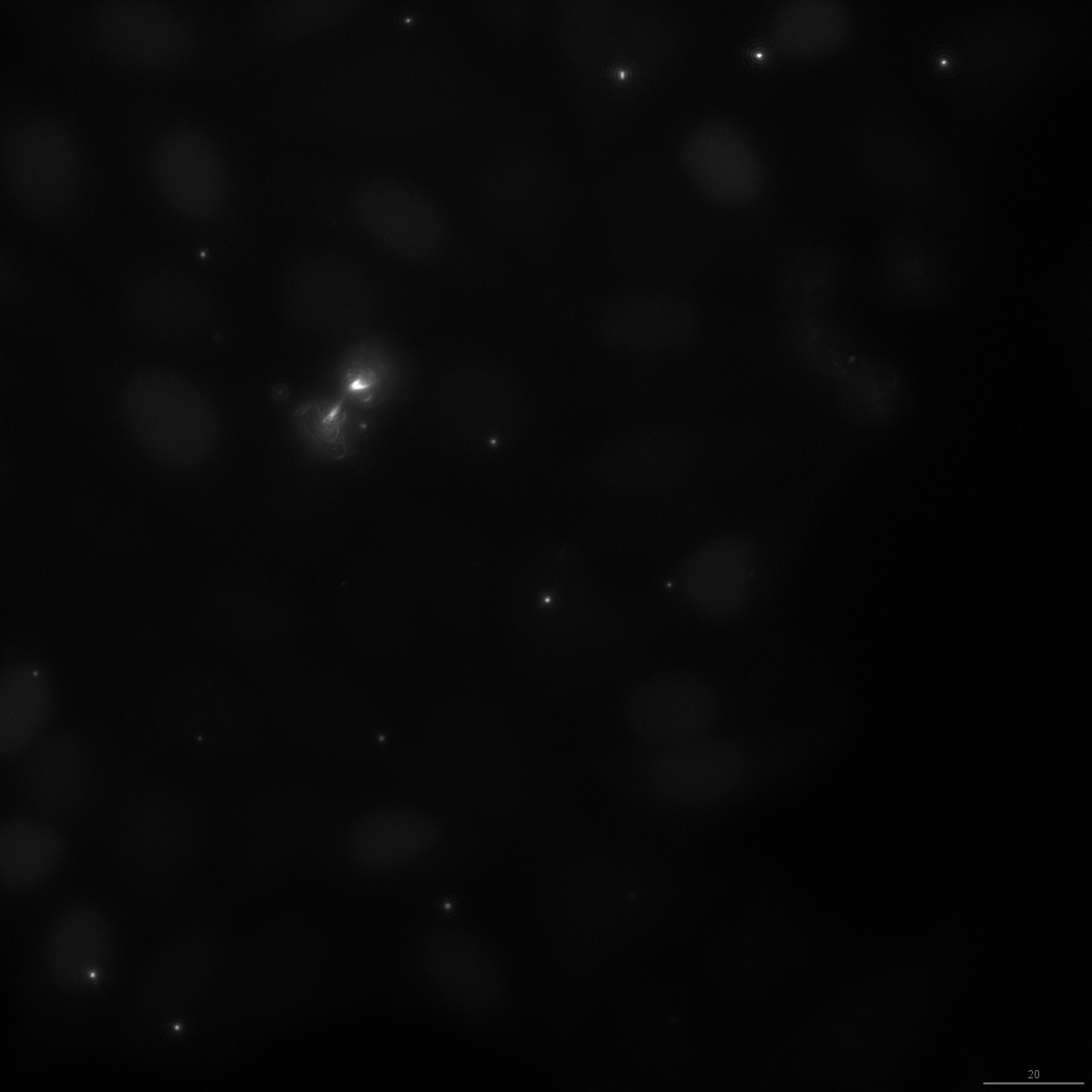

Supplement: Supplementary file 10 — Source Data for Figure 7 [file EMBJ-42-e113647-s009.zip › Figure 7/Figure 7G/uncropped MEE/8 min/PRC1MEEto3AGFP.tif]
