# Supplementary material for: Phosphorylation controls spatial and temporal activities of motor‐PRC1 complexes to complete mitosis
Source: EMBO J. 2023 Aug 18;42(21):e113647. doi: 10.15252/embj.2023113647 (PMC10620760; doi:10.15252/embj.2023113647)
Supplement: Supplementary file 11 [file EMBJ-42-e113647-s005.pdf]

THE EMBO JOURNAL

Phosphorylation controls spatial and temporal activities of motor-PRC1 complexes to complete mitosis

Agata Gluszek-Kustusz, Benjamin Craske, Thibault Legal, Toni McHugh, and Julie Welburn
DOI: 10.15252/embj.2023113647

Corresponding author(s): Julie Welburn (julie.welburn@ed.ac.uk)

Review Timeline:

Submission Date:	31st Jan 23
Editorial Decision:	6th Mar 23
Revision Received:	30th Jun 23
Accepted:	25th Jul 23

Editor: Hartmut Vodermaier

Transaction Report:

(Note: With the exception of the correction of typographical or spelling errors that could be a source of ambiguity, letters and reports are not edited. Depending on transfer agreements, referee reports obtained elsewhere may or may not be included in this compilation. Referee reports are anonymous unless the Referee chooses to sign their reports.)

Dr. Julie Pascale Izilda Welburn
University of Edinburgh
Wellcome Trust Centre for Cell Biology
Swann Building, Kings'Building
Mayfield Road
Edinburgh EH9 3BF
United Kingdom

6th Mar 2023

Re: EMBOJ-2023-113647
Phosphorylation controls spatial and temporal activities of motor-PRC1 complexes to complete mitosis

Dear Julie,

Thank you again for submitting your study on phosphorylation control of motor-PRC1 complexes to The EMBO Journal. Three expert referees have now assessed the manuscript and discussed with each other. Based on their combined feedback, we would be interested in pursuing this work further for publication, pending adequate revisions to address the points raised in the reports copied below.

As you will see, referees 1 and 3 bring up a number of specific queries, while referee 2 also raises some more significant conceptual issues, in particular the concern that PRC1-3A may not represent a true separation-of-function mutant and that the phenotypes in Figure 6 could alternatively be due to an abnormal microtubule-PRC1 interaction - a possibility that referee 1 noted might be tested by using microtubule binding and TIRF assays with the PRC1-3A mutant. Moreover, reviewer 2 outlines various technical/experimental concerns related to the size exclusion chromatographies that would need to be clarified - in this respect, please see also the referee's detailed annotation of the SEC data in the attached image file

Since it is our policy to consider only a single round of major revision, and therefore important to fully answer to all comments at the time of resubmission, I am herewith inviting you to carefully consider the points raised by all referees, and to get back to me with a tentative response letter/revision plan already during the early stages of the revision work - I would subsequently like to discuss this plan and any arising issues via Zoom call. I should add that we could also offer extension of the default three-months revision period if needed, with our 'scooping protection' (meaning that competing work appearing elsewhere in the meantime will not affect our considerations of your study) remaining of course valid also throughout this extension.

Detailed information on preparing, formatting and uploading a revised manuscript can be found below and in our Guide to Authors. Thank you again for the opportunity to consider this work for The EMBO Journal, and I look forward to further discussing with you.

With kind regards,

Hartmut

Hartmut Vodermaier, PhD
Senior Editor, The EMBO Journal
h.vodermaier@embojournal.org

*** PLEASE NOTE: All revised manuscript are subject to initial checks for completeness and adherence to our formatting guidelines. Revisions may be returned to the authors and delayed in their editorial re-evaluation if they fail to comply to the following requirements (see also our Guide to Authors for further information):

1) Every manuscript requires a Data Availability section (even if only stating that no deposited datasets are included). Primary datasets or computer code produced in the current study have to be deposited in appropriate public repositories prior to resubmission, and reviewer access details provided in case that public access is not yet allowed. Further information: embopress.org/page/journal/14602075/authorguide#dataavailability

2) Each figure legend must specify
- size of the scale bars that are mandatory for all micrograph panels
- the statistical test used to generate error bars and P-values
- the type error bars (e.g., S.E.M., S.D.)

- the number (n) and nature (biological or technical replicate) of independent experiments underlying each data point
- Figures may not include error bars for experiments with n<3; scatter plots showing individual data points should be used instead.

3) Revised manuscript text (including main tables, and figure legends for main and EV figures) has to be submitted as editable text file (e.g., .docx format). We encourage highlighting of changes (e.g., via text color) for the referees' reference.

4) Each main and each Expanded View (EV) figure should be uploaded as individual production-quality files (preferably in .eps, .tif, .jpg formats). For suggestions on figure preparation/layout, please refer to our Figure Preparation Guidelines: http://bit.ly/EMBOPressFigurePreparationGuideline

5) Point-by-point response letters should include the original referee comments in full together with your detailed responses to them (and to specific editor requests if applicable), and also be uploaded as editable (e.g., .docx) text files.

6) Please complete our Author Checklist, and make sure that information entered into the checklist is also reflected in the manuscript; the checklist will be available to readers as part of the Review Process File. A download link is found at the top of our Guide to Authors: embopress.org/page/journal/14602075/authorguide

7) All authors listed as (co-)corresponding need to deposit, in their respective author profiles in our submission system, a unique ORCiD identifier linked to their name. Please see our Guide to Authors for detailed instructions.

8) Please note that supplementary information at EMBO Press has been superseded by the 'Expanded View' for inclusion of additional figures, tables, movies or datasets; with up to five EV Figures being typeset and directly accessible in the HTML version of the article. For details and guidance, please refer to:
embopress.org/page/journal/14602075/authorguide#expandedview

9) Digital image enhancement is acceptable practice, as long as it accurately represents the original data and conforms to community standards. If a figure has been subjected to significant electronic manipulation, this must be clearly noted in the figure legend and/or the 'Materials and Methods' section. The editors reserve the right to request original versions of figures and the original images that were used to assemble the figure. Finally, we generally encourage uploading of numerical as well as gel/blot image source data; for details see: embopress.org/page/journal/14602075/authorguide#sourcedata

At EMBO Press, we ask authors to provide source data for the main manuscript figures. Our source data coordinator will contact you to discuss which figure panels we would need source data for and will also provide you with helpful tips on how to upload and organize the files.

Further information is available in our Guide For Authors:
https://www.embopress.org/page/journal/14602075/authorguide

In the interest of ensuring the conceptual advance provided by the work, we recommend submitting a revision within 3 months (4th Jun 2023). Please discuss the revision progress ahead of this time with the editor if you require more time to complete the revisions. Use the link below to submit your revision:

Link Not Available

Referee #1:

This is an interesting and timely study on the function of PRC1 in cell division. PRC1 is one of the most highly conserved cytoskeletal proteins needed for cell division, and has been linked to the function of a number of different kinesin motors in anaphase. Despite that understanding, many questions remain, especially in regard to how PRC1 binds to kinesin motors. This new study from the Wellburn group combines some high-quality biochemistry with microscopy to ask how PRC1 interacts with two important kinesins, CENP-E and KIF4A. This together with an analysis of the role of CENP-E phosphorylation in regulating the interaction with PRC1 and demonstration the interaction is important in cells, convincingly extends previous work in this area, which has mainly focussed on the regulation of PRC1 binding to microtubules by phosphorylation from CDKs and dephosphorylation by PP2A.

Specific points:
Figure S3 is important and I would prefer to see that as a main figure, or added to one of the existing figures. The model really helps understand the mechanism that authors are testing. Also, two suggestions - the PRC1-3A naming isn't particularly

obvious, and a more descriptive name would be helpful. The SEC traces are not really needed in Figure S3, and the gel panels could go across the page under the Alphafold2 model.

Do MKLP1 and KIF14 have motifs related to those described in this work for CENP-E and KIF4A? Have the authors looked at those motors in the PRC1-3A expressing cells depleted of endogenous PRC1?

Page 13: "As chromosomes bi-orient and segregate, CENP-E is dephosphorylated, which increases its affinity for PRC1 and facilitates its recruitment to the central spindle.".
The data show a pattern of CENP-E recruitment in anaphase, not during chromosome bi-orientation. Based on the behaviour of the CENP-E point mutants it is reasonable to conclude CENP-E is phosphorylated and becomes dephosphorylated, however that is not directly shown. This should be reworded: "As chromosomes segregate in anaphase, we conclude CENP-E is dephosphorylated, which increases its affinity for PRC1 and facilitates its recruitment to the central spindle."

Do the authors have any insight into the phosphatase triggering CENP-E dephosphorylation at the right time?

Figure 6H. The cells appear to be binucleate, consistent with the experimental timing (i.e. the cells failed one division). The graph says multinucleate which I think should be changed to binucleate. Although a statistical test for significance is shown, error bars are also missing.

Page 6. "In cell extracts, PRC1 interacts with Kif4A, Kif14, MKLP1 and CENP-E (Kurasawa et al., 2004).". Kurusawa et al showed data for KIF4, MKLP1 and CENP-E but not KIF14. The KIF14 interaction was reported in the Gruneberg et al 2006 citation using IP from cells.

I found the "inverted" images for PRC1 and GFP-GST-CENPE in Fig. 1C a bit difficult to see. The more usual presentation used in Fig. 1E was clearer.

Some microscopy scale bars are labelled (figure 5), whereas others are not (Figure 6). My preference is for a label e.g. "10 µm".

In Figure 6D can labels for the channels be added below or to the side of the image panels.

Referee #2:

Robust cell division relies on morphological dynamics of the mitotic spindle, the microtubule-based machinery for sister chromatid separation, and the regulator of cytokinesis. This highly complicated process is executed by the coordinated action of various microtubule regulators including kinesin-like motor proteins such as KIF4 and CENP-E and non-motor microtubule bundlers such as PRC1. The physical interactions between these different classes of microtubule regulators have been reported and proposed to play key roles in spindle dynamics, especially the formation of the post-anaphase structures such as the central spindle and the midbody. However, in many cases, the mechanistic detail of the interaction and its in vivo roles have been left unclear.

In this manuscript, the authors investigated the interaction between PRC1 and two kinesins, KIF4A and CENP-E. Recently, a motif consisting of two consecutive hydrophobic amino acids in the tail domain of KIF4A (ΦΦ, di-hydrophobic motif) was discovered to be key for its interaction with PRC1. In this manuscript, the authors discovered two hydrophobic motifs in the C-terminal tail of CENP-E as well as the second one on KIF4A. First, the significance of the bipartite ΦΦ motif on CENP-E for CENP-E-PRC1 interaction was examined in vivo and in vitro (SEC and ITC) using a C-terminal fragment of CENP-E without mutations in the two ΦΦ motifs (Fig. 1 and 2). The interaction between the C-terminal KIF4A fragment containing the bipartite ΦΦ motif and the PRC1 was also confirmed (although the biochemistry of the ΦΦ mutants is missing) (Fig. 3). This was followed by in vitro reconstitutions of microtubule bundling by PRC1 and CENP-E (Fig. 4), which confirmed that dimeric CENP-E C-terminal fragment interacts with MTs only via its interaction with PRC1 and demonstrated that the full-length CENP-E can slide microtubules bundled by PRC1. Then, the phosphorylation of CENP-E-PRC1 interaction was investigated with phospho-defective and phospho-memetic mutations at 6 putative phosphorylation sites near the bipartite ΦΦ motif (Fig. 5). Finally, the authors modeled the PRC1-kinesin interactions using AlphaFold2 and designed a mutant PRC1 defective for this (PRC1 3A) and studied the in vivo phenotypes (Fig. 6). The mutant cells showed abnormal PRC1 localization, the central spindle defect, and multinucleation.

The PRC1-kinesin interaction has a long history. A difficulty in testing the biological significance of the interaction is that a phenotype by simple depletion of PRC1 or a kinesin is not sufficient as supportive evidence. We need to test a mutant of PRC1 or a kinesin (ideally both) that specifically affects the interaction without affecting other known functions of them. In this manuscript, the PRC1 3A mutant has been treated as such a mutant. However, the influence of the 3A mutation on the functions of PRC1 other than the interaction with the motors is not sufficient. Although in vivo tests of the ΦΦ motif mutants of CENP-E and KIF4A could have provided complementary evidence, such analysis has not been done. Thus, currently, we can't firmly exclude the possibility that the phenotypes in Figure 6 were caused by an abnormal microtubule-PRC1 interaction.

(Major points)
1. Biochemical characterization of the PRC1 3A mutant
1a Size-exclusion chromatography (SEC)
The only test for the influence of the 3A mutation on the function of PRC1 is "PRC11-168 3A was soluble and behaved similarly to PRC11-168 in size-exclusion chromatography (Fig S3B).". It is unclear how reliable their data is with the size-exclusion chromatography as to the sizes of individual molecules. According to the chromatogram in Fig. 2A, the elution peak of the wild-type PRC1 1-168 fragment appears at 13 mL. This is consistent with the SDS-PAGE gel, which shows a single peak at lanes 8-9 (from left to right, assuming 10 lanes). On the other hand, in Fig. 3C, while the chromatogram shows a single peak of the same PRC1 construct at 13 mL, the PAGE gel shows two peaks at lanes 8 and 10 (left to right assuming 15 lanes). This raises a question of how reproducible the authors' SEC data are. The 3A mutant in S3B showed a peak at 13 mL on the chromatogram, and a single peak on the PAGE gel at lanes 8 and 9 (assuming 10 lanes) similar to the wild type in Fig. 2A. However, this is different from the wild type in Fig. 3C. These indicate that the resolutions of the provided SEC data are sufficient for testing the complex between the fragments of PRC1 and the motors but not for the absolute size of the PRC1 fragment itself.

Zhu 2006 analyzed the PRC1 in a HeLa cell extract by sucrose density gradient (SDG) (Zhu and Jian 2006, PMID:16603632) and found 3 fractions of PRC1, 1) > 230 kDa not phosphorylated at a CDK1 site (T481), 2) between 140 kDa and 66 kDa and T481 phosphorylation, 3) closer to 66 kDa but not phosphorylation. Based on these, they suggested phospho-regulated oligomerization although the interpretation of their SDG data is not so simple since PRC1 can interact with kinesins and both the density and shape of the molecule affect the behavior of molecules in SDG. I don't think that the authors should perform this type of experiment. However, it is worth keeping in mind that PRC1 might take more forms than a dimer without kinesin and a dimer with kinesin. The multiple peaks of wild type PRC1 1-168 wild type alone in Fig. 3C might be due to dissociation of the dimer or further oligomerization.

The phenotypes in Fig. 6 might have been caused by a defect caused by the 3A mutation in a function that can only be detected by a full-length protein. It is essential to perform hydrodynamic characterization (SEC+SDG or SEC+MALS) as well as more functional studies (see below) with full-length wild-type and 3A PRC1 preparations.

By the way, there are additional concerns about the SEC data, the long tails of Fig. 2B, which might be caused by a non-specific interaction between the protein samples and the Superdex gel substratum (ionic strength might be too low), and the peaks at the exclusion volume (8~9 mL) in Fig. S3B (presence of a large aggregate). For these experiments, the way how data are presented is very poor. The size standard is missing. The chromatogram should be displayed with the peak positions of the standards such as https://www.bio-rad.com/webroot/web/pdf/lsr/literature/MSLIT-102E.pdf and https://www.sigmaaldrich.com/NL/en/product/sigma/ge28403842. More than half of the x-range of the presented chromatograms are outside of the range in which the tested fragments or the complex would appear. Zoom in to the range between the exclusion volume (8~9 mL) to the small molecule (15~18 mL?). The lines are too thick, nearly corresponding to one lane of the PAGE gel, limiting the resolution. The lanes on the PAGE gels lack annotations. We can't really compare the chromatogram and the PAGE gel. The fractions should be indicated on both the chromatograms and the PAGE gels. If the fractions were collected in a large volume, multiple peaks might be collected in a single fraction. A consistent fractionation pattern is essential for comparing different experiments.

1b. Functional characterization of the 3A mutant PRC1 as a MT-bundler
Even if the 3A mutation didn't affect dimerization or further oligomerization of PRC1, this doesn't guarantee that this mutation doesn't affect other functions of PRC1. Ideally, the mutant PRC1 should have been tested in the same kind of experiments as in Fig. 4 in parallel with the wild-type PRC1. The phenotypes in Fig. 6 can be explained by the altered preference of PRC1 for the polarity of microtubule bundles. The natural preference of PRC1 for the anti-parallel configuration of microtubule bundles might have been lost by the 3A mutation.
By the way, why weren't the ΦΦ mutants tested at all in Fig. 4?

2. Characterisation of the GFP-PRC1 strains
2-a. Number of strains studied
It is known that just a slight over-expression of PRC1 can cause excess microtubule bundling on the pre-anaphase spindles (Mollinari 2002). Thus, we should be very careful about the expression levels of the WT and 3A GFP-PRC1. However, this point has been left unclear. In addition, in general, a strain per mutation is not sufficient because we can't exclude the possibility that an unknown mutation somewhere in the genome that might have happened during the isolation of the strain might be the cause of the phenotype. Multiple cell lines per mutation should be examined. The expression levels of GFP-PRC1 and its heterogeneities among strains should be assessed by a combination of western blotting for PRC1 and GFP, and by quantifying the GFP intensity. By the way, what was the parental cell line? This should be clarified in the Materials and Methods section.

2-b Phenotypic characterization
It is not clear how many cells were observed in live (Fig. 6 D). How frequently was cytokinesis failed? At which stage the failure happened? Were these consistent with Fig. 6 G and H? No defects in chromosome segregation (elevated premature PRC1-localization on the metaphase spindle might cause a chromosome segregation error such as a chromosome bridge, which would in turn interfere with abscission,)?

3. In vivo test of the ΦΦ motif mutants of kinesins
The ΦΦ motif mutants of the kinesins would have provided evidence for the significance of the PRC-kinesin interaction with the 100% guarantee that observed phenotypes are not primarily due to a non-specific defect of PRC1. For KIF4A, such experiments have been done in Poser 2019 (FF1154AA and FF1220AA). They reported the mislocalization of PRC1 by FF1154AA but not by FF1220AA. The newly identified FF motifs on CENP-E should be tested in vivo in three combinations (FF 1st to AA, FF 2nd to AA, and both) (and ideally, in combination with the KIF4A FF1154AA mutant although this is technically demanding). This is absolutely required to claim "Here we demonstrate that CENP-E promotes microtubule-microtubule sliding later in anaphase, and functions as a complex of CENP-E:PRC1, in a similar manner to Kif4A" (page 12).

By the way "There is a second ΦΦ motif in Kif4A (F1220, F1221) upstream of the published PRC1-binding region (F1154, F1155) (Fig 3B)." sounds odd. FF1220 was published in Poser 2019 and even tested in vivo. In addition, 1220 is usually described as 'downstream' of 1154.

(Minor points)
-(page 8) "CENP-E-driven microtubule sliding was slow, with an average velocity of 10.4 {plus minus} 2.5 nm/s (Fig 4H)" => Fig. 4G. Fig. 4H is not referred from the main text.
- What is the curve in Fig. 4G? A normal distribution? Really? If so, do you think it is because you are not distinguishing the direction of the motility by CENP-E?
- The main text and the Fig. 6A legend mention that both PRC1 CRISPR and PRC1 siRNA were performed. However sufficient detail has not been provided. What was the timing of the induction of the CRISPR-based knockout relative to knockdown by siRNA? What was the concentration of doxycycline?
- In the Fig. 6A legend, the annotations of DNA and microtubules are messed up. What is the difference between the top two rows and the rest?
- The legends for Fig. 6B and 6C are messed up.
- FIg. 6H misses error bars. Does this mean only a single experiment with n=286 and 393 was performed? The percentage of multinucleation in the WT GFP-PRC1 cell line seems to be very low (maybe even lower than the normal HeLa cells). Was this reproducible?
- (page 13) "PRC1 is proposed to act as a break counteracting forces that drive spindle elongation (Janson et al., 2007)". This is the work in yeast. The same role has been proposed and examined in animal cells (PMIDs: 15458647, 26088160). These should be properly discussed.
-(page 14) "Our in vivo data indicate that the resistive or brake forces produced by the microtubule- crosslinking activity of PRC1 are too low to oppose forces that drive spindle elongation (Fig 6)." I don't understand the logic here. For interpreting the data in Fig. 6 in this way in the context of the works by Forth 2004 and Gaska 2020, we need to measure the rates of spindle elongation and the number of microtubules and PRC1 molecules.
- In Table 1, It might be useful for readers if a column is added for the name/abbreviations used in the main text and figure legends (e.g. "MBP-CENP-E2605-2701 ΔΦΦ" for the 4th row).

Referee #3:

In this manuscript the authors utilize a variety of approaches to understand how the kinesin motor protein, CenpE, is recruited to the central spindle during anaphase of mitosis. Overall, the manuscript provides new information and will be of interest to workers in the field.

One aspect of the work is to understand how the PRC1-CenpE interaction is regulated. These experiments are well done and show that conserved residues in the C-terminus of CenpE contribute to binding, and further show that Kif4a requires a similar bipartite motif. On page 6, the subheading "CenpE binds PRC1 with high affinity" might be edited to indicate that the measurements were done with tagged, truncated CENP-E constructs.

The observations regarding microtubule sliding in assays of microtubules, PRC1 and CenpE provide additional insight into how motors contribute to changes in the midzone overlap. It would be of interest to understand how the results might change if Kif4A was also examined, and if dynamic microtubules were assayed. However, these experiments might be the topic for future work.

Throughout the manuscript the authors examine localization of CenpE in cells using overexpression of tagged, truncated constructs. I had some concerns about the degree to overexpression and the extent to which the introduced exogenous constructs compete with the endogenous. This should be mentioned. For figure 2, how many experiments and cells were analyzed?

In fig 4 G, how many experiments for the data on 28 microtubules?

In my experience, fluorescent, live cell probes for tubulin result in alterations in the microtubule arrays; was this encountered using sir-tubulin? Was the dilution 1:40,000 and for how long?

In figure 6 - panels B and C are reversed in the legend.

We thank the reviewers for their constructive comments and feedback. Please find our response to the reviewers'comments documenting our changes to the manuscript.

Referee #1:
Specific points:
Figure S3 is important and I would prefer to see that as a main figure, or added to one of the existing figures. The model really helps understand the mechanism that authors are testing. Also, two suggestions - the PRC1-3A naming isn't particularly obvious, and a more descriptive name would be helpful. The SEC traces are not really needed in Figure S3, and the gel panels could go across the page under the Alphafold2 model.
We agree with the reviewer and have followed this suggestion to include the alphafold model and the gel panels in Figure 6, so that this figure includes the molecular determinants of PRC1-CENP-E interactions.
We have also renamed our PRC1 3A mutant to PRC1-MEE.

Do MKLP1 and KIF14 have motifs related to those described in this work for CENP-E and KIF4A? Have the authors looked at those motors in the PRC1-3A expressing cells depleted of endogenous PRC1?
We have now shown that in the presence of PRC1 MEE, Kif4A is not recruited to microtubules at the site of abscission (Figure 7D, E).
We could not identify from primary protein sequence a PRC1-binding motif in MKLP1, MKLP2 or Kif14. Work in C.elegans has identified a hydrophobic motif in the RhoGAP protein that binds MKLP1 necessary for PRC1 binding. We have included this in the text: "It is possible that the other kinesins such as Kif14, MKLP2, MKLP1 use a ΦΦ motif for PRC1 recruitment to the central spindle. However, we could not identify the PRC1 binding site from the primary sequence. MKLP1 forms a heterotetramer with the RhoGAP Cyk4/MgcRacGAP, which has a highly conserved hydrophobic motif important for PRC1 binding in C.elegans (Lee et al., 2015)."

Page 13: "As chromosomes bi-orient and segregate, CENP-E is dephosphorylated, which increases its affinity for PRC1 and facilitates its recruitment to the central spindle.".
The data show a pattern of CENP-E recruitment in anaphase, not during chromosome bi-orientation. Based on the behaviour of the CENP-E point mutants it is reasonable to conclude CENP-E is phosphorylated and becomes dephosphorylated, however that is not directly shown. This should be reworded: "As chromosomes segregate in anaphase, we conclude CENP-E is dephosphorylated, which increases its affinity for PRC1 and facilitates its recruitment to the central spindle."
We have now amended the sentenced and included also: At the metaphase to anaphase transition, cyclin B degradation leads to a decrease in mitotic kinase activity.

Do the authors have any insight into the phosphatase triggering CENP-E dephosphorylation at the right time?
We currently do not know which phosphatase dephosphorylates CENP-E. PP1 and PP2A are the most likely candidates. CENP-E binds BubR1 at kinetochores, which itself binds PP2A. CENP-E also binds directly PP1 on a site that is dephosphorylated by PP1 in vitro (Kim et al, 2010). This is an interesting but difficult question to address in the future.

Figure 6H. The cells appear to be binucleate, consistent with the experimental timing (i.e. the cells failed one division). The graph says multinucleate which I think should be changed to binucleate. Although a statistical test for significance is shown, error bars are also missing.
We have now included error bars and corrected "multinucleate" to "binucleate".

Page 6. "In cell extracts, PRC1 interacts with Kif4A, Kif14, MKLP1 and CENP-E (Kurasawa et al., 2004).". Kurusawa et al showed data for KIF4, MKLP1 and CENP-E but not KIF14. The KIF14 interaction was reported in the Gruneberg et al 2006 citation using IP from cells.

We apologize for not mentioning this reference on this page. It is now included, as well as previously mentioned on page 4.

I found the "inverted" images for PRC1 and GFP-GST-CENPE in Fig. 1C a bit difficult to see. The more usual presentation used in Fig. 1E was clearer.
We have now changed the images to have a black background.

Some microscopy scale bars are labelled (figure 5), whereas others are not (Figure 6). My preference is for a label e.g. "10 μm".
We have now included scalebars for each image. We apologize for the inconsistencies.

In Figure 6D can labels for the channels be added below or to the side of the image panels.
We have now split the channels.

Referee #2:
(Major points)
1. Biochemical characterization of the PRC1 3A mutant
1a Size-exclusion chromatography (SEC)
The only test for the influence of the 3A mutation on the function of PRC1 is "PRC11-168 3A was soluble and behaved similarly to PRC11-168 in size-exclusion chromatography (Fig S3B).". It is unclear how reliable their data is with the size-exclusion chromatography as to the sizes of individual molecules. According to the chromatogram in Fig. 2A, the elution peak of the wild-type PRC1 1-168 fragment appears at 13 mL. This is consistent with the SDS-PAGE gel, which shows a single peak at lanes 8-9 (from left to right, assuming 10 lanes). On the other hand, in Fig. 3C, while the chromatogram shows a single peak of the same PRC1 construct at 13 mL, the PAGE gel shows two peaks at lanes 8 and 10 (left to right assuming 15 lanes). This raises a question of how reproducible the authors' SEC data are. The 3A mutant in S3B showed a peak at 13 mL on the chromatogram, and a single peak on the PAGE gel at lanes 8 and 9 (assuming 10 lanes) similar to the wild type in Fig. 2A. However, this is different from the wild type in Fig. 3C. These indicate that the resolutions of the provided SEC data are sufficient for testing the complex between the fragments of PRC1 and the motors but not for the absolute size of the PRC1 fragment itself.
We have annotated our gels to indicate the elution volume on the X-axis. SEC is not a good way to estimate protein size and we do not comment on protein size but only complex formation. Because PRC1 is an elongated coiled coil rich protein, it will move faster through a SEC column that standard globular protein the same size. For each figure, we have run all protein alone and together, so all the gels for that experiment are internally consistent. However, they are not to be compared across figures directly. For Figure 3, our conclusion is that GST-Kif4A co-elutes with PRC1, as there is a shift in the elution profile and the proteins are in the same fractions on the gel.

Zhu 2006 analyzed the PRC1 in a HeLa cell extract by sucrose density gradient (SDG) (Zhu and Jian 2006, PMID:16603632) and found 3 fractions of PRC1, 1) > 230 kDa not phosphorylated at a CDK1 site (T481), 2) between 140 kDa and 66 kDa and T481 phosphorylation, 3) closer to 66 kDa but not phosphorylation. Based on these, they suggested phospho-regulated oligomerization although the interpretation of their SDG data is not so simple since PRC1 can interact with kinesins and both the density and shape of the molecule affect the behavior of molecules in SDG. I don't think that the authors should perform this type of experiment. However, it is worth keeping in mind that PRC1 might take more forms than a dimer without kinesin and a dimer with kinesin. The multiple peaks of wild type PRC1 1-168 wild type alone in Fig. 3C might be due to dissociation of the dimer or further oligomerization.
We thank the reviewer for this information. We have performed SEC-MALS analysis on the PRC1 and PRC1-MEE. The results show they are both identical, dimeric and no dissociation is seen.
The phenotypes in Fig. 6 might have been caused by a defect caused by the 3A mutation in a

function that can only be detected by a full-length protein. It is essential to perform hydrodynamic characterization (SEC+SDG or SEC+MALS) as well as more functional studies (see below) with full-length wild-type and 3A PRC1 preparations.

We have performed SEC-MALS analysis on the PRC1$_{1\text{-}168}$ and PRC1$_{1\text{-}168}$ -MEE. The results show they are both identical and dimers. Because the point mutations are in the N terminus and do not perturb the dimerization of PRC1, we believe the results would be the same with full-length protein. We have now tested the microtubule bundling properties of full-length PRC1-MEE and show they are similar to that for full-length PRC1. The data are presented in figure 6.

By the way, there are additional concerns about the SEC data, the long tails of Fig. 2B, which might be caused by a non-specific interaction between the protein samples and the Superdex gel substratum (ionic strength might be too low), and the peaks at the exclusion volume (8~9 mL) in Fig. S3B (presence of a large aggregate). For these experiments, the way how data are presented is very poor. The size standard is missing. The chromatogram should be displayed with the peak positions of the standards such as https://www.bio-rad.com/webroot/web/pdf/lsr/literature/MSLIT-102E.pdf and https://www.sigmaaldrich.com/NL/en/product/sigma/ge28403842. More than half of the x-range of the presented chromatograms are outside of the range in which the tested fragments or the complex would appear. Zoom in to the range between the exclusion volume (8~9 mL) to the small molecule (15~18 mL?). The lines are too thick, nearly corresponding to one lane of the PAGE gel, limiting the resolution. The lanes on the PAGE gels lack annotations. We can't really compare the chromatogram and the PAGE gel. The fractions should be indicated on both the chromatograms and the PAGE gels. If the fractions were collected in a large volume, multiple peaks might be collected in a single fraction. A consistent fractionation pattern is essential for comparing different experiments.

All fractions are 0.5ml, collected separately after a run on a superdex 200 10/30GL.We have now made the lines thinner and zoom in on the elution profiles, all gels in the same panel are internally consistent with same fractions displayed. We have included the fractions selected. We also include here the size standards for our S200 column used in our study. However it would be too crowded to include this on the size exclusion chromatography profile.

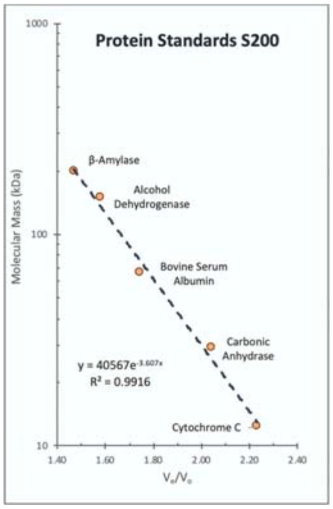

1b. Functional characterization of the 3A mutant PRC1 as a MT-bundler
Even if the 3A mutation didn't affect dimerization or further oligomerization of PRC1, this doesn't guarantee that this mutation doesn't affect other functions of PRC1. Ideally, the mutant PRC1 should have been tested in the same kind of experiments as in Fig. 4 in parallel with the wild-type PRC1. The phenotypes in Fig. 6 can be explained by the altered preference of PRC1 for the polarity of microtubule bundles. The natural preference of PRC1 for the anti-parallel configuration of microtubule bundles might have been lost by the 3A mutation.

A change in polarity of the PRC1 point mutant is virtually impossible. The crystal structure of the N terminus of PRC1 published in (Subramanian et al, 2013) revealed the molecular basis for the dimerization interface and the dimeric organization of PRC1. PRC1 forms a dimer with a 1700A buried area, suggesting a large dimerization interface. The two-fold point symmetry is key to orient the two microtubule binding sites into opposite directions so they can crosslink antiparallel microtubules. It would be very difficult to change the preference of PRC1 for the polarity of microtubules.
We have now performed experiments showing that full-length PRC1-MEE is able to bundle microtubules similarly to full-length PRC1. Therefore the mutations do not disrupt the ability of PRC1 to bundle microtubules. This experiment is shown in figure 6.

By the way, why weren't the ΦΦ mutants tested at all in Fig. 4?
We showed in cells and using SEC and ITC that CENP-E mutants do not bind PRC1. Therefore, it was not necessary to show that CENP-E mutant does not bind to PRC1 using reconstitution assays. For experiment 4F-G, these experiments are very complex to perform, because only a small fraction of full-length CENP-E is active (Craske et al, 2022). Full-length CENP-E is only active after purification from insect cells for a day or so. Therefore we did not do experiments with full-length CENP-E mutant that does not bind to PRC1. Instead in supplementary figure 2B, we show that CENP-E alone does not crosslink microtubules.

2. Characterisation of the GFP-PRC1 strains
2-a. Number of strains studied
It is known that just a slight over-expression of PRC1 can cause excess microtubule bundling on the pre-anaphase spindles (Mollinari 2002). Thus, we should be very careful about the expression levels of the WT and 3A GFP-PRC1. However, this point has been left unclear. In addition, in general, a strain per mutation is not sufficient because we can't exclude the possibility that an unknown mutation somewhere in the genome that might have happened during the isolation of the strain might be the cause of the phenotype. Multiple cell lines per mutation should be examined. The expression levels of GFP-PRC1 and its heterogeneities among strains should be assessed by a combination of western blotting for PRC1 and GFP, and by quantifying the GFP intensity.

It is very unusual to report various strains and selection of clones in papers, even if one screens multiple clones. Our cell lines express constitutively GFP-PRC1, therefore the gene expressed and the level of gene expression does not interfere with cell viability. We screened our cells after FACS sorting clones from 1 96-well plate. We used western blotting to analyze the levels of PRC1 contributed from the transgene and from the endogenous protein (See below). We also inspected the cell line clones visually before selecting 1, that was used for our studies. We have edited the methods section, a detailed protocol is available from Cheeseman and Desai, 2005.

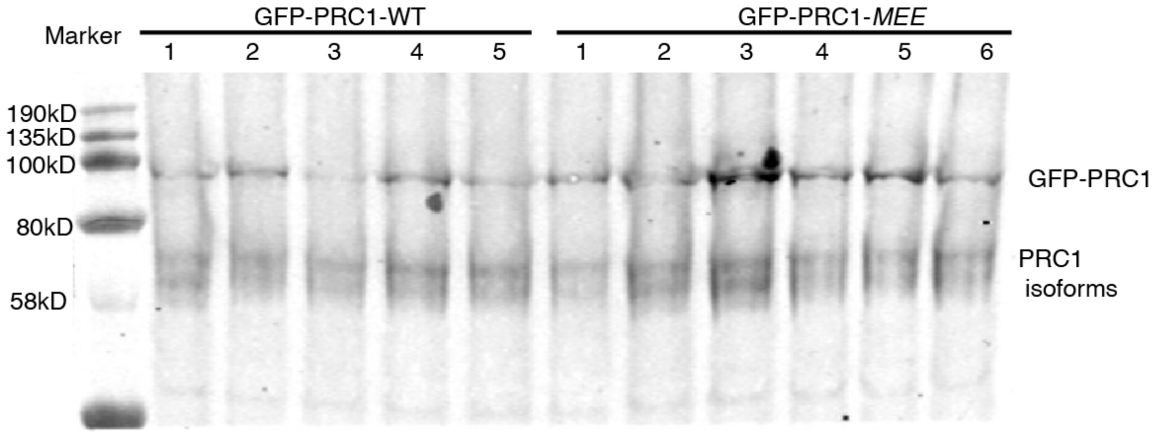

By the way, what was the parental cell line? This should be clarified in the Materials and Methods section.
We have now included the parental cell line in the methods. It is also mentioned in the figure legends.

2-b Phenotypic characterization
It is not clear how many cells were observed in live (Fig. 6 D).
We have now provided this information.

How frequently was cytokinesis failed? At which stage the failure happened? Were these consistent with Fig. 6 G and H?
We did not use live-cell imaging to assess failure of cytokinesis. We used live-cell imaging to examine the GFP-PRC1 bundles and localization during anaphase. Furrow ingression occurs however, it is later that cytokinesis failed. Over our time-lapse experiment, the Cell Mask dye highlighting the cell contour fades away and prevents us from imaging long term cell fate and cells fail cytokinesis after furrow ingression.

No defects in chromosome segregation (elevated premature PRC1-localization on the metaphase spindle might cause a chromosome segregation error such as a chromosome bridge, which would in turn interfere with abscission)?
This is an interesting question. We do not see the PRC1-MEE localizing prematurely to the spindle because this mutant specifically inhibits its interaction with proteins that bind to it through a ΦΦ motif, such as Kif4A and CENP-E. We did not see any major problems with chromosome segregation in our live-cell imaging, however we plan to analyze this in more detail in the future. We believe failure to complete cytokinesis is because the kinesin motors can no longer bind PRC1 and bring Aurora B/Plk1 there (essential for finalizing this step).

3. In vivo test of the ΦΦ motif mutants of kinesins
The newly identified FF motifs on CENP-E should be tested in vivo in three combinations (FF 1st to AA, FF 2nd to AA, and both) (and ideally, in combination with the KIF4A FF1154AA mutant although this is technically demanding). This is absolutely required to claim "Here we demonstrate that CENP-E promotes microtubule-microtubule sliding later in anaphase, and functions as a complex of CENP-E:PRC1, in a similar manner to Kif4A" (page 12).
It is a great suggestion. We have been trying to do these experiments for over 2 years, however they are technically challenging. We have the CENPE-GFP WT and mutant (MEE, SD, SA mutant) cloned into vectors. However three people from my lab have tried multiple strategies to make cell lines and so far we have not managed. We are still trying alternative ways to overcome the issue. In the meantime, we have edited the text to remove this claim.

By the way "There is a second ΦΦ motif in Kif4A (F1220, F1221) upstream of the published PRC1-binding region (F1154, F1155) (Fig 3B)." sounds odd. FF1220 was published in Poser 2019 and even tested in vivo. In addition, 1220 is usually described as 'downstream' of 1154.
We thank the reviewer for spotting this mistake, we have now changed the wording to "downstream". Indeed, this motif (F1220, F1221) was tested by the Barr lab and it was not sufficient to abrogate the interaction. They concluded it was not important for the interaction with PRC1. We show the motif (F1154, F1155) is necessary but not sufficient. In the text we say "Together, these data indicate that both while the previously reported Kif4a ΦΦ motif is necessary to bind PRC1 (Poser et al., 2019), it is not sufficient. "

(Minor points)
-(page 8) "CENP-E-driven microtubule sliding was slow, with an average velocity of 10.4 {plus minus} 2.5 nm/s (Fig 4H)" => Fig. 4G. Fig. 4H is not referred from the main text.
We have now revised the labelling of the panels.

- What is the curve in Fig. 4G? A normal distribution? Really? If so, do you think it is because you are not distinguishing the direction of the motility by CENP-E?
We have removed the curve fitting as it was detracting from the actual data and it was not useful, reporting the sliding velocity of microtubules. We thank the reviewer for pointing this out.

- The main text and the Fig. 6A legend mention that both PRC1 CRISPR and PRC1 siRNA were performed. However sufficient detail has not been provided. What was the timing of the induction of the CRISPR-based knockout relative to knockdown by siRNA? What was the concentration of doxycycline?
We have now added this information in the text.

- In the Fig. 6A legend, the annotations of DNA and microtubules are messed up. What is the difference between the top two rows and the rest?
We have corrected the panels and annotated the top and bottom panels, as they represent 2 stages of late mitosis where CENP-E and PRC1 are present normally.

- The legends for Fig. 6B and 6C are messed up.
We have now correct this.

- Fig. 6H misses error bars. Does this mean only a single experiment with n=286 and 393 was performed? The percentage of multinucleation in the WT GFP-PRC1 cell line seems to be very low (maybe even lower than the normal HeLa cells). Was this reproducible?
We reproduced this experiment 3 times and we have included error bars. We examined whether the cells were binucleate based on the position of the nucleus and the cytoskeleton for both HeLa cells expressing GFP-PRC1 and GFP-PRC1 mutant. It could be a fraction of cells had 2 nuclei but they were not close together and were classified as mononuclear. In that case, binucleated cells would be slightly underrepresented but that would be in both samples, which were quantified in parallel. The ROI for binucleated cells in each image was recorded with the data on Omero.

- (page 13) "PRC1 is proposed to act as a break counteracting forces that drive spindle elongation (Janson et al., 2007)". This is the work in yeast. The same role has been proposed and examined in animal cells (PMIDs: 15458647, 26088160). These should be properly discussed.
We have now discussed these papers: 15458647 on page 3 to report C.elegans PRC1/SPD-1 is essential for central spindle formation, and 26088160 on page 14 alongside Janson et al 2007. 26088160 is also already mentioned page 4.

-(page 14) "Our in vivo data indicate that the resistive or brake forces produced by the microtubule- crosslinking activity of PRC1 are too low to oppose forces that drive spindle elongation (Fig 6)." I don't understand the logic here. For interpreting the data in Fig. 6 in this way in the context of the works by Forth 2004 and Gaska 2020, we need to measure the rates of spindle elongation and the number of microtubules and PRC1 molecules.
We have rewritten this paragraph to dampen our conclusions from our data.

- In Table 1, It might be useful for readers if a column is added for the name/abbreviations used in the main text and figure legends (e.g. "MBP-CENP-E2605-2701 ΔΦΦ" for the 4th row).
We have now included this column.

Referee #3:

In this manuscript the authors utilize a variety of approaches to understand how the kinesin motor protein, CenpE, is recruited to the central spindle during anaphase of mitosis. Overall,

the manuscript provides new information and will be of interest to workers in the field.
One aspect of the work is to understand how the PRC1-CenpE interaction is regulated. These experiments are well done and show that conserved residues in the C-terminus of CenpE contribute to binding, and further show that Kif4a requires a similar bipartite motif. On page 6, the subheading "CenpE binds PRC1 with high affinity" might be edited to indicate that the measurements were done with tagged, truncated CENP-E constructs.
We have edited this subheading.

The observations regarding microtubule sliding in assays of microtubules, PRC1 and CenpE provide additional insight into how motors contribute to changes in the midzone overlap. It would be of interest to understand how the results might change if Kif4A was also examined, and if dynamic microtubules were assayed. However, these experiments might be the topic for future work.
This is an interesting suggestion. The work done with Kif4A and dynamic microtubules by the Kapoor and Surrey labs in vitro is beautiful. However due to technical challenges, it is not possible to do these experiments with CENP-E at the moment, as only a small fraction of CENP-E is active (Craske et al, 2022). Freshly purified CENP-E was used for in vitro sliding assays in Figure 4 due to deterioration in activity after freezing. Understanding the behavior of both Kif4A and CENP-E in vitro on crosslinked microtubules are an interest future experiments.

Throughout the manuscript the authors examine localization of CenpE in cells using overexpression of tagged, truncated constructs. I had some concerns about the degree to overexpression and the extent to which the introduced exogenous constructs compete with the endogenous. This should be mentioned. For figure 2, how many experiments and cells were analyzed?
Figure 2 does not contain any cell biology experiments. We believe the reviewer meant figure 5. We agree that transient transfection and overexpression of cells has some limitations. We only use this approach for testing the l tail domains of Kif4A and CENP-E (2608-2701) which interact with PRC1.
For experiments with full-length PRC1, we used stable monoclonal cell lines and slected for modest expression levels. All our conclusions from transient transfections of the small tails of Kif4A and CENP-E (2608-2701) are confirmed biochemically in vitro by ITC, which is a robust and quantitative method. ITC provides thermodynamic data, affinity measurements and the stoichiometry of binding. We have now moved these data to the main figure to support the cell biology data.

In fig 4 G, how many experiments for the data on 28 microtubules?
The data collected are from two independent experiments.

In my experience, fluorescent, live cell probes for tubulin result in alterations in the microtubule arrays; was this encountered using sir-tubulin? Was the dilution 1:40,000 and for how long?
We have now added more details to the method. We are using SiR at 25 nM, well below the recommended level by Cytoskeleton Inc. We agree with the reviewer that at the higher recommended concentrations, there are sometimes alterations in spindle organization, which is why we optimized our use of this probes. We have previously used it for long-term imaging at low concentration of spindle positioning (McHugh et al, 2018), without seeing any defects in spindle organization or only very occasionally.

In figure 6 - panels B and C are reversed in the legend.
Apologies, we have swapped the figure legends around.

Dr. Julie Pascale Izilda Welburn
University of Edinburgh
Wellcome Trust Centre for Cell Biology
Swann Building, Kings'Building
Mayfield Road
Edinburgh EH9 3BF
United Kingdom

25th Jul 2023

Re: EMBOJ-2023-113647R
Phosphorylation controls spatial and temporal activities of motor-PRC1 complexes to complete mitosis

Dear Dr. Welburn,

Thank you for submitting your final revised manuscript for our consideration. I am pleased to inform you that in light of the positive re-reviews (copied below), we have now accepted it for publication in The EMBO Journal.

Your article will be processed for publication in The EMBO Journal by EMBO Press and Wiley, who will contact you with further information regarding production/publication procedures and license requirements. You will also be provided with page proofs after copy-editing and typesetting of main manuscript and expanded view figure files.

Should you be planning a Press Release on your article, please get in contact with embojournal@wiley.com as early as possible, in order to coordinate publication and release dates.

Thank you again for this contribution to The EMBO Journal and congratulations on a successful publication! Please consider us again in the future for your most exciting work.

Yours sincerely,

Hartmut Vodermaier

Hartmut Vodermaier, PhD
Senior Editor, The EMBO Journal
h.vodermaier@embojournal.org

Referee #1:

The authors of the study have investigated how the microtubule binding protein PRC1 and a cohort of kinesin motor proteins work together at the anaphase spindle to promote cytokinesis. The key question addressed by their work is the mechanism of interaction between the kinesin motors and PRC1, and its functional relevance. The data identify a phi-phi motif present in two kinesins CENP-E and KIF4a which mediates the interaction with PRC1, and use structural modelling to identify a potential binding site on PRC1. This model is tested using a combination of in vitro biochemistry and cell based localisation and functional assays. Those data clear show that CENP-\E and KIF4a use this binding site on PRC1 for anaphase spindle localisation, and show that this property of PRC1 is essential for cytokinesis.

The revised manuscript is greatly improved both adding new and important content, and is improved in structure and general presentation compared to the original submission. The additional data on the PRC1-MEE mutant is crucial since it shows the protein can still bind and bundle microtubules, which is important for supporting the major conclusions of the work.

Overall, the authors have engaged with the reviewer comments and carried out a thorough revision. My only minor comment is there is probably a need for one final round of proofreading of the main text.

Referee #3:

The authors have addressed the concerns that were raised and is now acceptable for publication.

Please note that it is EMBO Journal policy for the transcript of the editorial process (containing referee reports and your response letter) to be published as an online supplement to each paper. If you do NOT want this, you will need to inform the Editorial Office via email immediately. More information is available here: https://www.embopress.org/page/journal/14602075/authorguide#transparentprocess

EMBO Press Author Checklist

Corresponding Author Name: Julie Welburn	
Journal Submitted to: EMBO J	
Manuscript Number: EMBOJ-2023-113647R	

USEFUL LINKS FOR COMPLETING THIS FORM
[The EMBO Journal - Author Guidelines]
[EMBO Reports - Author Guidelines]
[Molecular Systems Biology - Author Guidelines]
[EMBO Molecular Medicine - Author Guidelines]

Reporting Checklist for Life Science Articles (updated January
This checklist is adapted from Materials Design Analysis Reporting (MDAR) Checklist for Authors. MDAR establishes a minimum set of requirements in transparent reporting in the life sciences (see Statement of Task: 10.31222/osf.io/9sm4x). Please follow the journal's guidelines in preparing your manuscript.
Please note that a copy of this checklist will be published alongside your article.

Abridged guidelines for figures
1. Data
The data shown in figures should satisfy the following conditions:
- the data were obtained and processed according to the field's best practice and are presented to reflect the results of the experiments in an accurate and unbiased manner.
- ideally, figure panels should include only measurements that are directly comparable to each other and obtained with the same assay.
- plots include clearly labeled error bars for independent experiments and sample sizes. Unless justified, error bars should not be shown for technical
- if n<5, the individual data points from each experiment should be plotted. Any statistical test employed should be justified.
- Source Data should be included to report the data underlying figures according to the guidelines set out in the authorship guidelines on Data

2. Captions
Each figure caption should contain the following information, for each panel where they are relevant:
- a specification of the experimental system investigated (eg cell line, species name).
- the assay(s) and method(s) used to carry out the reported observations and measurements.
- an explicit mention of the biological and chemical entity(ies) that are being measured.
- an explicit mention of the biological and chemical entity(ies) that are altered/varied/perturbed in a controlled manner.
- the exact sample size (n) for each experimental group/condition, given as a number, not a range;
- a description of the sample collection allowing the reader to understand whether the samples represent technical or biological replicates (including how many animals, litters, cultures, etc.).
- a statement of how many times the experiment shown was independently replicated in the laboratory.
- definitions of statistical methods and measures:
  - common tests, such as t-test (please specify whether paired vs. unpaired), simple χ2 tests, Wilcoxon and Mann-Whitney tests, can be unambiguously identified by name only, but more complex techniques should be described in the methods section;
  - are tests one-sided or two-sided?
  - are there adjustments for multiple comparisons?
  - exact statistical test results, e.g., P values = x but not P values < x;
  - definition of 'center values' as median or average;
  - definition of error bars as s.d. or s.e.m.

Please complete ALL of the questions below.
Select "Not Applicable" only when the requested information is not relevant for your study.

Materials

Newly Created Materials	Information included in the manuscript?	In which section is the information available? (Reagents and Tools Table, Materials and Methods, Figures, Data Availability Section)
New materials and reagents need to be available; do any restrictions apply?	Yes	methods

Antibodies	Information included in the manuscript?	In which section is the information available? (Reagents and Tools Table, Materials and Methods, Figures, Data Availability Section)
For **antibodies** provide the following information:<br>- Commercial antibodies: RRID (if possible) or supplier name, catalogue number and or/clone number<br>- Non-commercial: RRID or citation	Yes	methods

DNA and RNA sequences	Information included in the manuscript?	In which section is the information available? (Reagents and Tools Table, Materials and Methods, Figures, Data Availability Section)
**Short novel DNA or RNA including primers, probes**: provide the sequences.	Yes	All siRNA have been previously characterised and references are included.

Cell materials	Information included in the manuscript?	In which section is the information available? (Reagents and Tools Table, Materials and Methods, Figures, Data Availability Section)
**Cell lines:** Provide species information, strain. Provide accession number in repository **OR** supplier name, catalog number, clone number, and/**OR** RRID.	Yes	
**Primary cultures:** Provide species, strain, sex of origin, genetic modification status.	Not Applicable	
Report if the cell lines were recently **authenticated** (e.g., by STR profiling) and tested for mycoplasma contamination.	Yes	

Experimental animals	Information included in the manuscript?	In which section is the information available? (Reagents and Tools Table, Materials and Methods, Figures, Data Availability Section)
**Laboratory animals or Model organisms:** Provide species, strain, sex, age, genetic modification status. Provide accession number in repository **OR** supplier name, catalog number, clone number, **OR** RRID.	Not Applicable	
**Animal observed in or captured from the field:** Provide species, sex, and age where possible.	Not Applicable	
Please detail **housing and husbandry conditions**.	Not Applicable	

Plants and microbes	Information included in the manuscript?	In which section is the information available? (Reagents and Tools Table, Materials and Methods, Figures, Data Availability Section)
**Plants:** provide species and strain, ecotype and cultivar where relevant, unique accession number if available, and source (including location for collected wild specimens).	Not Applicable	
**Microbes:** provide species and strain, unique accession number if available, and source.	Not Applicable	

Human research participants	Information included in the manuscript?	In which section is the information available? (Reagents and Tools Table, Materials and Methods, Figures, Data Availability Section)
If collected and within the bounds of privacy constraints report on age, sex and gender or ethnicity for all study participants.	Not Applicable	

Core facilities	Information included in the manuscript?	In which section is the information available? (Reagents and Tools Table, Materials and Methods, Figures, Data Availability Section)
If your work benefited from core facilities, was their service mentioned in the acknowledgments section?	Yes	

Design

Study protocol	Information included in the manuscript?	In which section is the information available? (Reagents and Tools Table, Materials and Methods, Figures, Data Availability Section)
If study protocol has been **pre-registered, provide DOI in the manuscript**. For clinical trials, provide the trial registration number **OR** cite DOI.	Not Applicable	
Report the **clinical trial registration number** (at ClinicalTrials.gov or equivalent), where applicable.	Not Applicable	

Laboratory protocol	Information included in the manuscript?	In which section is the information available? (Reagents and Tools Table, Materials and Methods, Figures, Data Availability Section)
Provide DOI OR other citation details if **external detailed step-by-step protocols** are available.	Yes	

Experimental study design and statistics	Information included in the manuscript?	In which section is the information available? (Reagents and Tools Table, Materials and Methods, Figures, Data Availability Section)
Include a statement about **sample size** estimate even if no statistical methods were used.	Yes	methods
Were any steps taken to minimize the effects of subjective bias when allocating animals/samples to treatment (e.g. **randomization procedure**)? If yes, have they been described?	Not Applicable	
Include a statement about **blinding** even if no blinding was done.	Yes	methods
Describe **inclusion/exclusion criteria** if samples or animals were excluded from the analysis. Were the criteria pre-established?<br><br>If sample or data points were omitted from analysis, report if this was due to attrition or intentional exclusion and provide justification.	Yes	methods
For every figure, are **statistical tests** justified as appropriate? Do the data meet the assumptions of the tests (e.g., normal distribution)? Describe any methods used to assess it. Is there an estimate of variation within each group of data? Is the variance similar between the groups that are being statistically compared?	Yes	figure legends

Sample definition and in-laboratory replication	Information included in the manuscript?	In which section is the information available? (Reagents and Tools Table, Materials and Methods, Figures, Data Availability Section)
In the figure legends: state number of times the experiment was **replicated** in laboratory.	Yes	Dtaa included for microscopy and cell biology data figure legend.
In the figure legends: define whether data describe **technical or biological replicates**.	Yes	methods/legends

Ethics

Ethics	Information included in the manuscript?	In which section is the information available? (Reagents and Tools Table, Materials and Methods, Figures, Data Availability Section)
Studies involving **human participants**: State details of **authority granting ethics approval** (IRB or equivalent committee(s), provide reference number for approval.	Not Applicable	
Studies involving **human participants**: Include a statement confirming that **informed consent** was obtained from all subjects and that the experiments conformed to the principles set out in the WMA Declaration of Helsinki and the Department of Health and Human Services Belmont Report.	Not Applicable	
Studies involving **human participants:** For publication of **patient photos**, include a statement confirming that consent to publish was obtained.	Not Applicable	
Studies involving experimental **animals**: State details of **authority granting ethics approval** (IRB or equivalent committee(s), provide reference number for approval. Include a statement of compliance with ethical regulations.	Not Applicable	
Studies involving **specimen and field samples:** State if relevant **permits** obtained, provide details of authority approving study; if none were required, explain why.	Not Applicable	

Dual Use Research of Concern (DURC)	Information included in the manuscript?	In which section is the information available? (Reagents and Tools Table, Materials and Methods, Figures, Data Availability Section)
Could your study fall under dual use research restrictions? Please check biosecurity documents and list of **select agents and toxins** (CDC): https://www.selectagents.gov/sat/list.htm	Not Applicable	
If you used a select agent, is the security level of the lab appropriate and reported in the manuscript?	Not Applicable	
If a study is subject to dual use research of concern regulations, is the name of the **authority granting approval and reference number** for the regulatory approval provided in the manuscript?	Not Applicable	

Reporting

The MDAR framework recommends adoption of discipline-specific guidelines, established and endorsed through community initiatives. Journals have their own policy about requiring specific guidelines and recommendations to complement MDAR.

Adherence to community standards	Information included in the manuscript?	In which section is the information available? (Reagents and Tools Table, Materials and Methods, Figures, Data Availability Section)
State if relevant guidelines or checklists (e.g., **ICMJE, MIBBI, ARRIVE, PRISMA**) have been followed or provided.	Not Applicable	
For **tumor marker prognostic studies,** we recommend that you follow the **REMARK** reporting guidelines (see link list at top right). See author guidelines, under 'Reporting Guidelines'. Please confirm you have followed these guidelines.	Not Applicable	
For **phase II and III randomized controlled trials**, please refer to the **CONSORT** flow diagram (see link list at top right) and submit the CONSORT checklist (see link list at top right) with your submission. See author guidelines, under 'Reporting Guidelines'. Please confirm you have submitted this list.	Not Applicable	

Data Availability

Data availability	Information included in the manuscript?	In which section is the information available? (Reagents and Tools Table, Materials and Methods, Figures, Data Availability Section)
Have **primary datasets** been deposited according to the journal's guidelines (see 'Data Deposition' section) and the respective accession numbers provided in the Data Availability Section?	Not Applicable	
Were **human clinical and genomic datasets** deposited in a public access-controlled repository in accordance to ethical obligations to the patients and to the applicable consent agreement?	Not Applicable	
Are **computational models** that are central and integral to a study available without restrictions in a machine-readable form? Were the relevant accession numbers or links provided?	Not Applicable	
If publicly available data were reused, provide the respective **data citations in the reference list**.	Not Applicable	
